# Extensive Gene Remodeling in the Viral World: New Evidence for Nongradual Evolution in the Mobilome Network

**DOI:** 10.1093/gbe/evu168

**Published:** 2014-08-07

**Authors:** Pierre-Alain Jachiet, Philippe Colson, Philippe Lopez, Eric Bapteste

**Affiliations:** ^1^UMR CNRS 7138 Evolution Paris Seine, IBPS, Université Pierre et Marie Curie, Paris, France; ^2^URMITE UMR CNRS 6236 IRD 198, Facultés de Médecine et de Pharmacie, Université de la Méditerranée, Marseille, France; ^3^Pôle des Maladies Infectieuses et Tropicales Clinique et Biologique, Fédération de Bactériologie-Hygiène-Virologie, Centre Hospitalo-Universitaire Timone, Marseille, France

**Keywords:** evolution, composite genes, virus, network, comparative genomics

## Abstract

Complex nongradual evolutionary processes such as gene remodeling are difficult to model, to visualize, and to investigate systematically. Despite these challenges, the creation of composite (or mosaic) genes by combination of genetic segments from unrelated gene families was established as an important adaptive phenomena in eukaryotic genomes. In contrast, almost no general studies have been conducted to quantify composite genes in viruses. Although viral genome mosaicism has been well-described, the extent of gene mosaicism and its rules of emergence remain largely unexplored. Applying methods from graph theory to inclusive similarity networks, and using data from more than 3,000 complete viral genomes, we provide the first demonstration that composite genes in viruses are 1) functionally biased, 2) involved in key aspects of the arm race between cells and viruses, and 3) can be classified into two distinct types of composite genes in all viral classes. Beyond the quantification of the widespread recombination of genes among different viruses of the same class, we also report a striking sharing of genetic information between viruses of different classes and with different nucleic acid types. This latter discovery provides novel evidence for the existence of a large and complex mobilome network, which appears partly bound by the sharing of genetic information and by the formation of composite genes between mobile entities with different genetic material. Considering that there are around 10E31 viruses on the planet, gene remodeling appears as a hugely significant way of generating and moving novel sequences between different kinds of organisms on Earth.

## Introduction

The assembly of genetic material from different gene families, producing composite genes (Enright et al. 1999; Jachiet et al. 2013), has been mostly described in eukaryotic genomes. Individual studies have shown that the combination of domains (Wang and Caetano-Anollés 2009) and the fusion of genes account for important aspects of biological complexity, from the evolution of distinct signaling systems to possible key evolutionary transitions such as animal multicellularity (Koonin et al. 2002). Genetic fragments common to all cellular beings are combined in specific ways in each domain of life, affecting as many as two-thirds of the proteins in unicellular organisms to over 80% in metazoa (Apic et al. 2001). However, the extent to which composite gene genesis is observed across the viral world is unquantified.

If one considers the mechanisms by which genomes of these major numerous evolutionary players evolve, it can immediately be noted that viruses exploit a vast pool of genes and that viral genomes are structurally and evolutionary highly constrained. Most viral genes are under purifying selection (Holmes 2003; Koonin and Wolf 2010) and intragenomic gene duplication is rare (Liu et al. 2006; Simon-Loriere and Holmes 2013) (with the exception of large and giant DNA viruses [Shackelton and Holmes 2004; Filée 2009]). However, frequent mutations, insertion/deletions, and hyperplastic regions allow viruses to go through their life cycle by escaping their hosts immune systems and defense mechanisms (Arias et al. 2009; Sanjuán et al. 2010). Moreover, many mechanisms could be, in principle at least, involved in the making of composite genes. More precisely, many viral genomes, such as double-stranded (ds) DNA bacteriophages (Casjens 2008; Hatfull 2008) and RNA viruses (Lai 1992; Barr and Fearns 2010; Jackwood et al. 2012), are highly recombinogenic (Lima-Mendez et al. 2008). Viral gene repertoire is thus commonly expanded by strand-switching, the use of incompletely replicated genetic material as a primer for another strain, by crossing-over of nonhomologous segments (Liu et al. 2006; Arias et al. 2009; Savolainen-Kopra and Blomqvist 2010), by genetic reassortment of fragments of genomes (Lei and Shi 2011), by the use of specific proteins enhancing recombination (Martinsohn et al. 2008), by transposition and illegitimate recombination joining pieces of DNA with limited homology (Crawford-Miksza and Schnurr 1996), and by the shuffling of groups of genes (modules) between genomes (Botstein 1980).

These processes may not only generate composite genomes but also composite genes in viruses. However, processes producing composite genes have not been systematically analyzed for these taxa, although an estimated 6–8% of viral genes have been reported to be multidomain (Hatfull 2008; Kristensen, Cai, et al. 2011; Kristensen et al. 2013), as well as few occasional cases of gene fusions between viruses of the same class (involving some tail fiber and replication genes [Highton et al. 1990], and two cyanophage photosynthetic genes [Sharon et al. 2009]). We seek to perform such a systematic analysis of composite genes in viral genomes, and in the process address three complementary questions. First, we tested whether composite genes link genetic material from different kinds of sequences in all viral classes based on three major classifications. Second, we tested whether these composite genes fulfilled central or less essential functions for the completion of the viral life cycle. Third, we investigated whether composite genes may be constituted from genetic segments from viruses belonging to different viral classes (e.g., DNA and RNA viruses), hence from distantly related or even unrelated viruses.

Systematic studies of composite genes are best formulated within the framework of sequence similarity networks (Adai et al. 2004). In these graphs, each node is an individual sequence, connected to others when they share some alignable regions with significant similarity (Atkinson et al. 2009). Composite genes act as detectable bridges that connect sequences harboring segments from unrelated gene families (Adai et al. 2004; Jachiet et al. 2013). Constant progress in sequencing technologies, computing power and memory capacities, network display (Shannon et al. 2003; Bastian et al. 2009) and analyses (Song et al. 2008; Berry et al. 2010; Jachiet et al. 2013) now permit the analysis of the structure of these graphs for data sets of thousands of viral genomes. Here, we mined the genes of 3,008 viral genomes and detected 8–15% composite sequences. These composite genes were found in all viral classes (according to three classifications), mostly encoding important functions for the viral life cycle. The emergence of composite genes operated beyond the frontiers of both viral classes and gene families, meaning that numerous viral adaptations are best understood from a global perspective, because boundaries or viral classes are not forbidding sharing of gene segments from different gene families.

## Materials and Methods

### Data Sets

The viral data set contains 122,392 protein sequences from 3,008 completely sequenced viral genomes, including all of those available at National Center for Biotechnology Information (NCBI) on November 2012, and additional genomes from members of the proposed order Megavirales (viruses in supplementary table S1, Supplementary Material online). The larger comparative data set, used to define important functional classes for viruses, includes protein sequences from completely sequenced plasmids (all available at NCBI) and a phylogenetically balanced selection of cellular organisms from all of life, resulting in a total of 740,842 sequences. Repartitioning of sequences into genetic vectors is summarized in supplementary table S1, Supplementary Material online, and supplementary table S1, Supplementary Material online, details all included genomes. Taxonomical annotation was based on 1) classification of viruses into families by the ICTV (http://talk.ictvonline.org/files/ictv_documents/m/msl/4440.aspx, last accessed August 28, 2014), 2) Baltimore classification that classified viruses according to the nature of their genome and their replicative strategy (Baltimore 1971), and 3) classification into five monophyletic classes of viruses and selfish genetic elements as demonstrated by Koonin et al. (2006).

### Functional Annotations

Sequences were functionally annotated by the category (Tatusov et al. 1997) of their best RPSBLAST match (if *E* value < 10E-5) against COG (baCteria) and KOG (euKaryota) orthologous groups (Tatusov et al. 2003). Sequences with no such significant hit were not considered in functional analyses (74% of viral data set and 50% of larger comparative data set). We did not use POGs (*24*) built on viral genomes, because those have not been grouped into higher functional classes.

### Statistical Test

To determine whether a functional category was significantly enriched in one gene set with respect to another, we performed a two-sided Fisher exact test of this category against the combination of every other category. To account for multiple testing on 25 functional categories, we used the conservative Bonferroni correction and considered significant only those categories for which *P* value < 0.02 = 0.05/25.

### Sequence Similarity Networks Construction and Analyses

We used the result of an all-against-all BLAST+ (Camacho et al. 2009) (softmasking with segmasker) comparison to build a sequence similarity network for this data set, joining pairs of sequences with an *E* value <10E-5. We symmetrized the network and removed multiple edges by keeping the best *E* value hit between each pairs of sequences. We mined this network to detect composite genes using FusedTriplets (Jachiet et al. 2013), with a stringency *E* value of 10E-10. We searched for multicomposite genes by using the same protocol on the subnetwork of previously identified composite genes. We clustered nodes into densely packed groups as determined by the first pass of Louvain community detection algorithm (Blondel et al. 2008). We used NetworkX (Hagberg et al. 2008) Python library to compute several networks metrics: assortativity (Newman 2003) of viral classes in the network, an approximate betweenness (Brandes and Pich 2007) of nodes using *k* = 5,000 random pivots, and a cycle basis of Louvain community network (to find edges participating in cycles). We produced the displays of sequence similarity networks using Cytoscape 2.0 (Shannon et al. 2003) with Force Directed Layout, and the display of Louvain community network Gephi (Bastian et al. 2009) with ForceAtlas2 Layout.

## Results

### Extensive Gene Remodeling in Viruses

We compared 122,392 sequences from 3,008 viruses in a BLAST all-versus-all analysis, searching for sequences with significant similar genetic fragments, called hits. Sequences were included as nodes in sequence similarity networks. Two sequences were connected when at least one of the pairwise BLAST comparisons returned a hit with an *E* value <10E-5. At this stringency threshold, false positive hits between nonhomologous sequences are not expected (Medini et al. 2006; Fokkens et al. 2010), although genuine homology between very divergent sequences can be missed. Using simple linkage, we partitioned the graph into 24,092 singletons and 12,506 clusters or connected components of two sequences or more. Homologous genes that have not diverged beyond recognition by BLAST typically produce such clusters. Composite sequences indirectly bridge several different homologous families in the graph, when distinct regions of composite sequences present similarity with distinct families. Thus composite sequences produce larger connected components, uniting sequences from different gene families (Enright and Ouzounis 2000; Kristensen, Wolf, et al. 2011; Jachiet et al. 2013). The largest connected component present in the network comprised 18,033 sequences (15% of the data set), demonstrating that composite genes involved genetic segments from numerous and diverse homologous families.

The topology of this network was explored to find candidate composite genes, using FusedTriplets (Jachiet et al. 2013). Composite genes fulfill three conditions: i) They fall at the center of a nontransitive triplet of nodes, ii) the hits between a candidate composite sequence and each of its two direct neighbors in such triplets must not overlap by more than 20 amino acids. (These short windows of potential overlap account for BLAST tendency to slightly extend a hit between two similar regions over nonhomologous regions by a few amino acids; this overlap criterion did not affect our results, as they were virtually unchanged when removing it—identifying 9,177 composite genes instead of 9,872 and 2,959 multicomposite genes instead of 3,351, see below.) iii) Along a nontransitive triplet, the edges between sequences with component fragments and the candidate composite sequence must present a similarity above the twilight zone (Rost 1999) (an *E* value of <10E-10 instead of the *E* value of <10E-5 used for network building), so no similarity, however weak, is found between component sequences. This latter condition ensures that nontransitive triplets do not comprise homologous divergent sequences, aligned over distinct regions. There were 423 million triplets to investigate, out of which 123 million were nontransitive (i), 85 million also fulfilled condition (ii) and 53 million fulfilled all three conditions. Within these latter, we counted 9,872 composite genes (8% of the data set, 10% of the sequences present in the network when singletons are excluded from the data set). Without enforcing the stringency condition (iii), 12% of the sequences (15% of the sequences present in the network without the singletons) were diagnosed as composite.

**Fig. 1.—:**
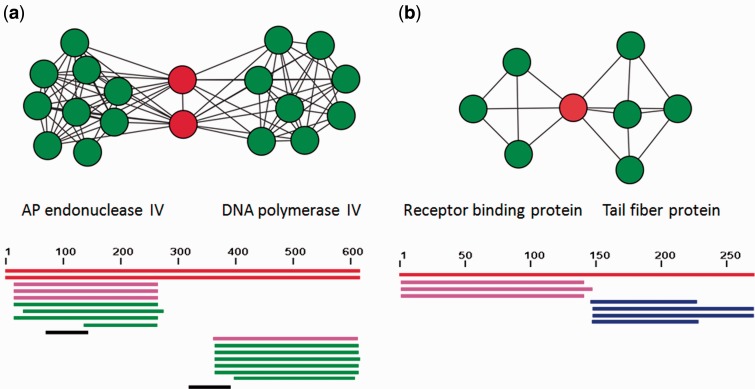
Examples of subgraphs with detected composite genes. Top panel: Subgraphs showing all direct neighbors of composite genes. Nodes (red for composites, green for components) correspond to genes connected by edges when they share a BLAST *E* value <10^−5^. Bottom panel: BLAST alignment showing the overlap between the sequence of a composite for the above gene families and component sequences. A plain segment indicates a region with overlap (significant similarity according to BLAST). Segments are colored to reflect bitscores (black: <40, blue: 40–50, green: 50–80, pink: 80–200, red: >200). Segments in different lines come from different genes. Top red segments correspond to composite genes, other segments to component genes. (*a*) AP endonuclease IV/DNA polymerase IV fusions in *Melanoplus sanguinipes* entomopoxvirus and *Amsacta moorei* entomopoxvirus. Component genes belong to NCLDV. (*b*) Composite receptor-binding protein in *Lactococcus* bacteriophage bIBB29. Sequences in the left component come from *Lactococcus* phages sk1, jj50, and 712; sequences in the right component come from *Streptococcus pyogenes* phage 315.5, *Cronobacter* phage vB_CsaP_GAP52, *Lactococcus* phage 949, and *Vibrio* phage KVP40.

As a proof of concept, our approach identified some formerly known composite genes. Typically, it detected the family of putative DNA polymerase beta/AP endonuclease proteins from the functionally important base excision repair system present in *Melanoplus sanguinipes* entomopoxvirus, consistently with (Afonso et al. 1999). Moreover, our analysis expanded this finding to another composite DNA polymerase beta/AP endonuclease proteins, that of the *Amsacta moorei* entomopoxvirus, when by contrast AP Endonuclease and DNA polymerase beta were observed to exist as physically independent genes in NCLDV viruses. Likewise, our protocol recovered the composite nature of large, multidomain helicase/methyltransferase proteins in *Burkholderia* phage BcepIL02 and in *Burkholderia* phage DC1. These genes, with widely distributed homologs across bacteria, plasmids and IS elements, code antirestriction proteins. Their remarkable size (about 4,650 amino acids, up to 23% of these viruses genomes) strongly suggests that these composite genes benefit to the mobile elements hosting them, likely by providing some protection from the restriction system of cells by DNA methylation (Gill et al. 2011; Lynch et al. 2012, p. 1). Similarly, we identified the composite receptor-binding protein, responsible for the attachment of the virion particle to its host, in the genome of *Lactococcus* bacteriophage bIBB29. Its unusual structure had been described in Hejnowicz et al. (2009), which reported that the N-terminal part of bIBB29 RBP gene is highly conserved among a first group of phages, whereas its C-terminal part demonstrates homology to a gene in another phage, P475, that does not belong to this group. Again, in connection with this first step of infection from cells by phages, we also detected various composite cell wall-associated hydrolases in our viral data set; consistently with previous reports of the modular organization of these invasion-associated genes (Loessner et al. 1997). These precise examples support the notion that a network-approach can successfully identify bona fide composite genes, and that some of these genes are apparently involved in key functions for the viral cycle (from DNA repair functions, important for virus survival, to the invasion of cellular hosts, or to the arm race between phages and cells). However, we immediately wish to add a note of caution. In principle, homologs massively diverging over nonoverlapping regions of their ancestrally derived sequences may also occasionally produce patterns that could be mistaken for that of typical composite genes (fig. 1); therefore, detailed analyses on focal candidate composite genes are to be encouraged, when the goal of the search for these composite is not a general survey as it is the case for this study.

**Fig. 2.—:**
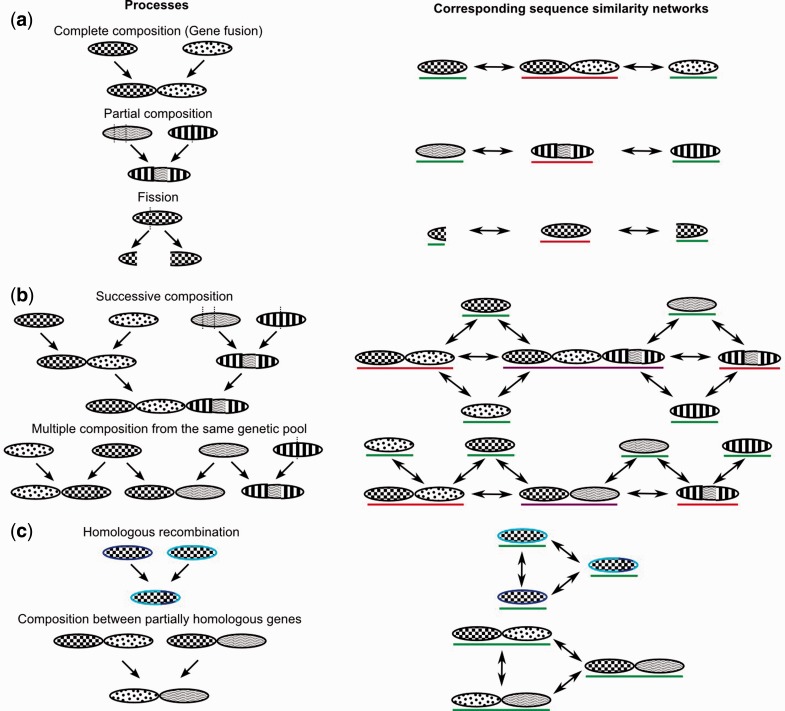
Processes producing composite genes and characteristic similarity patterns. Composite genes result from processes (left) that produce typical similarity networks (right). Shared inner motifs (e.g., wavelets) between genes indicate common ancestry. Underlined in color are genes detected as composite (red), as multicomposite (violet) or not detected as such (green). (*a*) Fusions and fissions lead to the detection of composite genes. Fissioned genes are composite because they combine fragments that exist as independent genes. (*b*) Multiple compositions lead to the detection of multicomposite genes. (*c*) Composition between homologous genes produces transitive similarity relationships and is not detected by this protocol.

Composite genes may be the outcomes of two distinct types of processes occurring in viral genomes, or in their cellular hosts: Fusion events (when components of composite genes originate from different gene families) and fission events (when components of composite genes terminate in different gene families). Here, we did not attempt to distinguish between these two processes (fig. 2). Rather we focused on another observation: All viral classes contained at least one composite gene (table 1). Furthermore, we detected an additional class of composite genes, called multicomposite genes. These multicomposite genes exploit sets of genetic segments found in sequences that were themselves identified as composite by the above protocol. For instance, patterns indicating multicomposite genes occur as a result of two successive steps when genetic fragments from distinct composite genes are subsequently assembled into a new sequence. Moreover, sets of multicomposite genes will also be observed when sequences diagnosed as composite are directly connected in the network, as these sequences evolved from different yet overlapping combinations of a common pool of genetic fragments (fig. 2). We detected these multicomposite genes by applying the three search conditions described above to a subset of the network, retaining only the sequences already identified as composite. We found 3,351 multicomposite viral sequences (3% of the data set, 4% of the sequences in the network without singletons). This is the first report of this class of composite sequences in viral genomes. Again, all viral classes contained at least one multicomposite gene (table 1).

**Table 1:** Composite and Multicomposite Genes in Viral Classes

	Data Set	Composite	Multicomposite
Baltimore classes			
I: dsDNA	109,324	7,488 (6.8)	2,372 (2.2)
II: ssDNA	3,071	732 (23.8)	12 (0.4)
III: dsRNA	819	35 (4.3)	4 (0.5)
IV: +ssRNA	6,000	1,218 (20.3)	763 (12.7)
V: -ssRNA	983	94 (9.6)	31 (3.2)
VI: +ssRNA	394	148 (37.6)	80 (20.3)
DNA intermediate			
VII: dsDNA	283	43 (15.2)	41 (14.5)
RNA intermediate			
Unknown	1,518	114 (7.5)	48 (3.2)
Nucleic acid			
DNA	112,941	8,270 (7.3)	2,431 (2.2)
RNA	8,212	1,495 (18.2)	878 (10.7)
Unknown	1,239	107 (8.6)	42 (3.4)
Monophyletic groups			
1	6,587	1,212 (18.4)	702 (10.7)
2	675	188 (27.9)	118 (17.5)
3	3,241	731 (22.6)	10 (0.3)
4	59,937	3,683 (6.1)	1,330 (2.2)
5	23,765	2,458 (10.3)	652 (2.7)
NA	28,187	1,600 (5.7)	539 (1.9)
Total	122,392	9,872 (8.1)	3,351 (2.7)

Note.—Number and percentages of composite and multicomposite genes in Baltimore and major monophyletic viral classes and by type of nucleic acid.

These proportions of composite sequences indicate that the fixation of composite genes is a general phenomenon in virus evolution. The number of composite genes is likely an underestimate, as some leave undetectable traces in sequence similarity networks (fig. 2). Moreover, we tested for eventual biases in the detection of short hits between component and composite sequences in our approach. Although some short fragments, shared between composite and component sequences, were identified, that is, the minimal ones measuring 25 amino acids, in general hits lengths between composite and component genes were slightly larger than hits lengths in the overall network (supplementary fig. S1, Supplementary Material online). In addition, frequency histograms of −log(*E* values) showed that there were more hits with low scores in the overall network (e.g., for −log(*E* values) ranging from 1 to 5) than there were such hits between composite and component sequences (supplementary fig. S2, Supplementary Material online). As −log10(*E* values) correlates with hits lengths (*r*^2 ^= 0.51/0.54 in the network/for hits between composite and component sequences), our protocol appears conservative: It could miss some short-sized hits between composite and component genes for low *E* values. Therefore, our numbers can be seen as a lower-bound estimate of composite genes in viruses. This minimal estimate of composite genes is consistent, yet provides new information with respect to former analyses of multidomain genes by Kristensen et al. (2013), because composite genes can be built from segments outside the boundaries of protein domains, and because estimates of composite genes for each viral class and functional categories have not been considered previously (see below).

### Remodeling of Genes Essential to the Viral Life Cycle

**Fig. 3.—:**
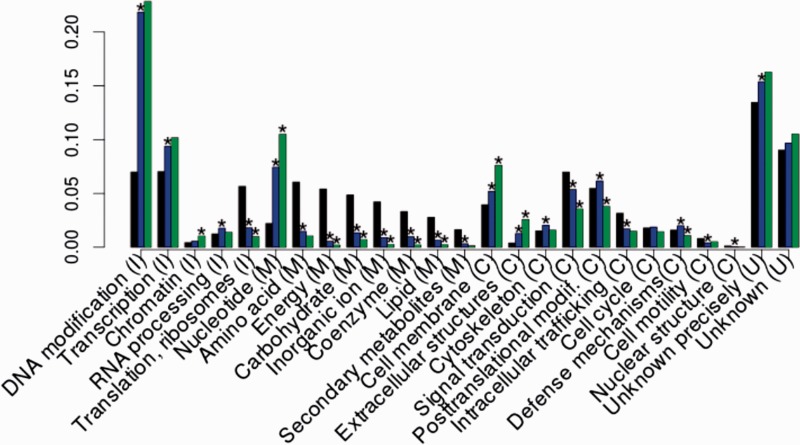
Functional distribution of cellular, viral, and viral composite genes. The proportion of genes in each functional category was plotted for the reference “cellular” data set (black), the viral data set (blue), and the viral composite subset (green). Genes assigned to multiple categories were redistributed evenly into each of the specified categories. Unannotated genes were not considered. Stars highlight functional categories significantly depleted or enriched in the viral data set with respect to the cellular data set, and in viral composite subset with respect to viral data set (Fisher test, overall significance level of 0.05). Final letter indicates broad functional categories: (I) information storage and processing, (C) cellular processes and signaling, (M) metabolism, (U) poorly characterized.

Composite genes were found in all functional categories in different proportions (fig. 3), confirming that they broadly contribute to the range of genetic diversity in viruses. Due to the strong selective pressures acting on viral genomes, one could argue that most of these composite genes are likely adaptative, as viruses have large population sizes these composite genes would be eliminated. One could also argue that some neutral ratchet-like mechanism (a form of constructive neutral evolution) (Gray et al. 2010) is responsible for the fixation of composite genes in viral genomes. One argument in favor of the adaptive interpretation of this extended distribution of composite genes is provided by the fact that these genes are overrepresented in specific functional categories, that is, the fixation is nonrandom. More precisely, we defined functional classes as important for viruses using a larger comparative data set including cellular organisms from all branches of life for a total of 740,842 sequences (supplementary table S1, Supplementary Material online). The comparison with this data set showed functional categories enriched in viruses with respect to cellular organisms. Such categories include replication, recombination and repair (DNA modifications), transcription, RNA processing and modification, chromatin structure and dynamics, posttranslational modification, protein turnover and chaperones, nucleotide transport and metabolism, cytoskeleton, cell wall/membrane/envelope biogenesis, extracellular structures, defense mechanisms, and unknown or precisely unknown functions. Remarkably, most of the categories that are functionally important for viruses were also enriched in viral composite genes (with the exception of posttranslational modification, protein turnover and chaperones, RNA processing and modification, defense mechanisms, and cytoskeleton). This trend of enrichment in viral composite sequences in functional categories important for viruses was most significant (*P* = 0.05) for chromatin structure and dynamics, nucleotide transport and metabolism, cell wall/membrane/envelope biogenesis, and extracellular structures. Therefore, the fixation of composite genes in viruses is biased with respect to functional categories, and composite genes for the most part belong to functions that are essential for the completion of the viral cycle. Noteworthy, several functions particularly enriched in composite genes (e.g., ribonucleotide reductase and thymidylate synthase) are encoded by genomes from large and giant DNA viruses (Boyer et al. 2010). Other composite genes of note encode ankyrin repeat containing proteins that are known to mimic or manipulate various host functions (Al-Khodor et al. 2010).

Indeed, there is a nongradual process of molecular evolution at the origin of such composite genes, because both genetic fission and genetic fusion differ from punctual mutations, and may be responsible of larger, potentially more damaging changes in the sequences. Remarkably, functionally important viral categories presented composite genes, even though changes in such key genes may be generally deleterious for their viral hosts. However, in large viral populations, such changes may be highly adaptive and therefore are relatively frequently observed in extant genomes as shown in our analysis. If composite genes within these functional categories are of benefit at least to some members of the population, for example by enhancing their potential to interact with their cellular hosts, to escape their immune systems and defense mechanisms, then composite genes are important players in that arms race between cells and viruses. These composite genes can be formed through a combinatorial process mixing gene lineages that sustains viral life cycles in all viral classes through (lucky) adaptive changes in key viral genes. If this adaptative interpretation is correct, this result proposes a novel instance of the red queen process in evolution, where intimate genetic transformations involving material beyond the boundaries of the gene family allow for the persistence of a lineage. As a note of caution with respect to this interpretation of the enrichment of composite genes in key viral functions, supplementary figure S1, Supplementary Material online, shows that nonannotated sequences are shorter than annotated sequences, and that annotated sequences enriched in viruses with respect to cellular organisms are larger than average annotated sequences and annotated sequences nonenriched in viruses. As composite genes are larger than noncomposite genes, it is very possible that key viral functions (e.g., annotated sequences enriched in viruses with respect to cellular organisms) are enriched in composite genes simply because genes with known function are longer genes overall.

The quantitative measures of composite genes proposed for each viral class and functional category depend on the quality of sequence annotation of viral genomes, and thus may vary as the annotation improves. We assessed the impact of quality of genomes on our conclusions, by restricting our analyses of the taxonomical and functional distribution of composite genes to a very stringently defined “safest” subset of 6,144 composite genes, using three additional conditions. We removed from our analysis all genes from nontransitive triplets in which components were found embedded in a composite from the same genome (this was to circumvent the issue of overlapping genes). We also removed composite genes found exclusively in one nontransitive triplet where the two component genes came from a single genome (to remove false positives due to genes artefactually split during the annotation process of that genome). Finally, we additionally removed all composite genes that were only found in one host genome, without homologs in any other genome (to reduce the possibility of including genes artefactually “fused” during the annotation process of that genome). The “safest” composite genes are found in all viral classes (following Baltimore classes, major monophyletic classes, or nucleic acid types). We recovered the exact same trends as previously described concerning functional categories (supplementary fig. S3, Supplementary Material online). In addition, 1,920 “safest” multicomposite genes were identified. Consequently, we do not suspect major biases in the trends detected here (although we cannot insulate against overall noise in the data from poorly sequenced genomes or misannotated genes).

### An Informative Network View of Molecular Changes in Viruses

The emergence of composite genes operates on a scale that is broader than gene families. Its study requires a more global perspective. The sequence similarity network, describing the viral sequence space, provides a suitable framework. We analyzed the topological properties of our graph to confirm that the detection of composite genes by means of intransitive triplets had successfully identified composite sequences acting as bridges between unrelated protein families. Indeed, composite sequences have a 17 times higher average betweenness (a centrality estimating the proportion of shortest paths in the network that pass through a node) than noncomposite sequences (2.7E-5 vs. 1.6E-6).

**Fig. 4.—:**
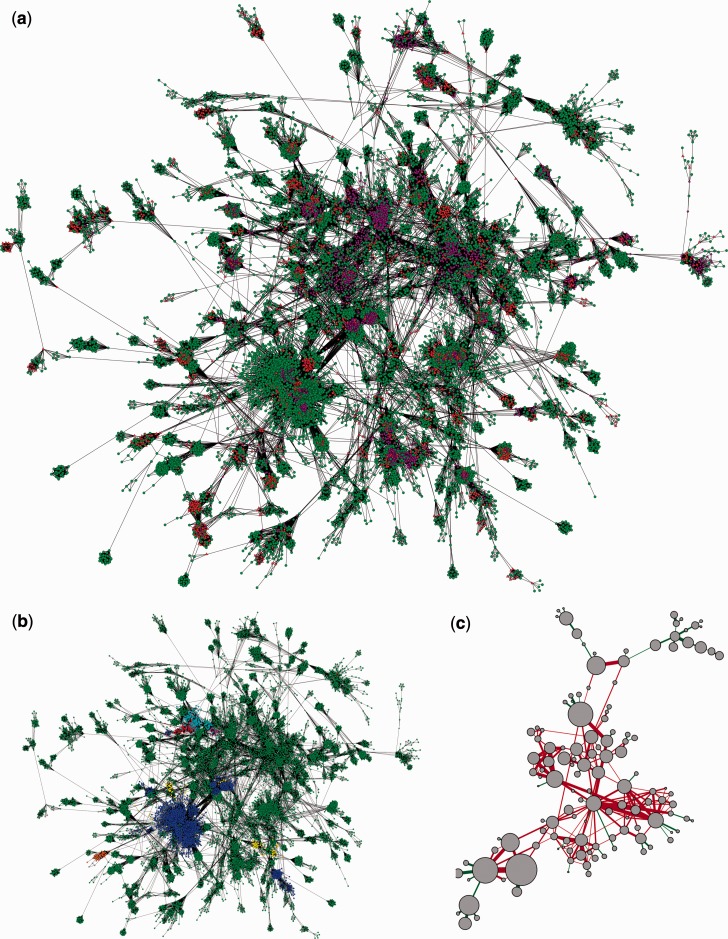
Giant-connected component of the viral gene similarity network. This graph contains 15% of the sequences, held together by composite genes. (*a*) Nodes are individual sequences, edges connect similar sequences (BLAST *E* value < 10E-5). Composite are in red, multicomposite in violet, and other genes in green. (*b*) Same graph with colors corresponding to Baltimore classes (dsDNA: green, ssDNA: orange, dsRNA: yellow, +ssRNA: dark blue, −ssRNA: purple, +ssRNA with DNA intermediate: light blue, dsDNA with RNA intermediate: red). (*c*) Simplification of the graph by pooling together densely connected groups of sequences. Super node area is proportional to community size. Edge width is proportional to 1 + log(number of intercommunity edges). Edges participating to cycles are colored in red.

We showed that these composite sequences bridge many densely connected regions (called graph communities, identified by Louvain community detection algorithm) into a giant-connected component (fig. 4). Moreover, composite genes introduce cycles between these graph communities. Such cycles indicate that sequences in this giant-connected component have not simply diverged from a last common ancestor. Indeed, although sequence divergence lowers the density of connections between homologous sequences in a sequence similarity network, it does not produce cycles. Homologous sequences presenting little conservation (i.e., a lesser sequence similarity across them than the threshold at which the network is constructed) will eventually produce chains of sequences. Instead, we demonstrated that similarities across sequences found in viruses presented cycles, which we visualized by pooling densely connected groups of nodes together in a super node in the graph (fig. 4*c*). These cycles constitute a unique network pattern to diagnose extensive gene remodeling (and nongradual evolutionary processes).

Importantly, other informative patterns of connections between viral sequences are also observed in the graph. A first major observation from the graph is that genes have a high tendency to be similar to genes from the same viral class, as measured by their assortativity (1 means perfect assortativity). The overall assortativity score for the Baltimore classes is 0.992. Thus, Baltimore classes’ overall assortativity is 0.992 (class I: 0.994, class II: 0.998, class III: 0.910, class IV: 0.997, class V: 0.987, class IV: 0.706, class VII: 0.705). Regarding classes VI and VII composed of viruses with different types of nucleic acid but all encoding a reverse transcriptase, their assortativity rises to 0.9 when aggregated. In addition, major monophyletic classes’ overall assortativity is 0.941 (class 1: 0.954, class 2: 0.929, class 3: 1.000, class 4: 0.903, class 5: 0.916). This preferential connection of like with like, for example, genes from the same viral class linked with one another, means that full or partial homologs are not usually readily detected in genomes across the viral classes considered here. The sharing or mixing of genetic material is not the rule for viruses from such distinct groups (which should not be confused with lower level classes such as International Committee on Taxonomy of Viruses [ICTV] families, for example, for which some sharing can be observed).

Although generally viruses from different groups have different genes, composite genes are not limited to associations of genetic material within a given viral class. Indeed, some viruses from different classes harbor sequences that are sufficiently similar to connect together in our graph. Consequently, densely connected sets of sequences from different viral classes or exploiting different nucleic acids fall into the same connected component. Despite the major structural and phylogenetic differences between their members, groups of sequences from viruses from all Baltimore and monophyletic classes (fig. 4*b* and supplementary fig. S4, Supplementary Material online) are indirectly aggregated into the giant-connected component, and in some other connected components. This complex pattern is expected when composite genes associate genetic fragments from different gene families of distinct viral origins into a single composite sequence, or when fragments of a composite sequence are inherited by different gene families from different viral classes. In either case, genetic information present in a given viral class can be effectively remodeled to work into another class of viruses. Figure 4*b* illustrates such cross-combination of genetic material from RNA and DNA viruses.

These results expand our view on the remarkable plasticity of viral genomes: Here illustrated by the combinations of information encoded in genetic material of different types and in unrelated entities (rather than by the more standard acquisition of stand-alone genes from viruses of the same class). Consistently, this holistic network reveals 40 instances of similar sequences distributed across Baltimore viral classes, 20 of them across RNA and DNA viruses, which represents further evidence that information (in particular coding the manipulation of DNA molecules) can be used by multiple members in the viral world, irrespective of biological support (e.g., RNA or DNA) (supplementary figs. S5 and S6, Supplementary Material online). Some large scale gene sharing between very different mobile entities (i.e., viruses and capsidless mobile elements) has recently been described elsewhere (Desnues et al. 2012; Yutin et al. 2013) giving rise to the concept of a mobilome network. Typically, virophages, polintons, some transposable elements, transpovirons, adenoviruses, and some bacteriophages were reported to form a network of evolutionary relationships, held together by overlapping sets of shared genes (Yutin et al. 2013). Our findings on composite genes originating from different viral host lineages provide a fundamentally novel line of evidence for the recognition of the broad scope of the mobilome network, and for the true genetic intricacy and fluidity within it.

## Discussion

Our systematic large scale analysis of composite sequences in viral genomes suggests that the fixation of composite genes is a general fundamental phenomenon in virus evolution. As composite genes were mostly found in functionally important gene categories (this suggests that they play a key role in persistence), in all viral classes. We report the existence of two classes of composite genes, involving genetic components from sequences belonging to distinct, eventually already composite, gene families. These results are relatively unexpected because unlike eukaryotic genomes, viral genomes are not characterized by the presence of intron–exon structure, or junk nucleic acids, that may ease the process of emergence of composite genes. Furthermore, we report composite genes involving information encoded on distant, and even unrelated viral classes, such as RNA viruses and DNA viruses. Viral genomes thus benefit from molecular evolution having occurred in distant lineages, possibly because this information, irrespective of which substrate it is encoded on, allows effective interactions with the machinery of cellular hosts, or alternatively because the functions encoded by some genetic fragments and compatible with any genome type may trace back to a profound connection between some RNA and DNA viruses. Noteworthy, chimeras between RNA and single-stranded (ssDNA) viruses were recently proposed to have resulted from recombination (Diemer and Stedman 2012; Roux et al. 2013).

We propose that the emergence of composite genes, relying on the combination of genetic material from different gene families, and occasionally from dramatically different classes of viruses, may be seen as a nongradual instance of the red queen process. Viral lineages benefit from introgressive combinations of genetic fragments that transform their genes important for their life cycle, allowing these lineages to survive in the cells–viruses arm races. Overall, the recognition of composite genes evolving from the association of genetic material beyond the scale of individual viral gene families and from distinct viral lineages provides further evidence that genome mosaicism is a general feature of viruses (Georgiades and Raoult 2012). This finding encourages the development of increasingly combinatorial models and network-based analyses of viral evolution. Future finer-grained analyses of the rules of combination of domains in viral genes are definitely one such option. Already, considering that there are around 10E31 viruses on the planet, our results indicate that gene remodeling is a hugely significant way of moving novel sequences between different kinds of organisms.

## Supplementary Material

Supplementary tables S1–S3 and figures S1–S6 are available at *Genome Biology and Evolution* online (http://www.gbe.oxfordjournals.org/).

Supplementary Data
