# Supplementary material for: Extensive Gene Remodeling in the Viral World: New Evidence for Nongradual Evolution in the Mobilome Network
Source: Genome Biol Evol. 2014 Aug 7;6(9):2195–205. doi: 10.1093/gbe/evu168 (PMC4202312; doi:10.1093/gbe/evu168)
Supplement: Supplementary Data [file supp_6_9_2195__index.html]

Extensive gene remodeling in the viral world : new evidence for non-gradual evolution in the mobilome network — Extensive Gene Remodeling in the Viral World: New Evidence for Nongradual Evolution in the Mobilome Network — Supplementary Data 

# Extensive Gene Remodeling in the Viral World: New Evidence for Nongradual Evolution in the Mobilome Network

## Supplementary Data

files

**Files in this Data Supplement:**

- Supplementary Data - pdf file
- Supplementary Data - jpg file
- Supplementary Data - docx file
