# Supplementary material for: Extensive Gene Remodeling in the Viral World: New Evidence for Nongradual Evolution in the Mobilome Network
Source: Genome Biol Evol. 2014 Aug 7;6(9):2195–205. doi: 10.1093/gbe/evu168 (PMC4202312; doi:10.1093/gbe/evu168)
Supplement: Supplementary Data [file supp_evu168_bapteste_table_S3.pdf]

| Type | Name | Number of genes | Baltimore class | Nucleic Acid | Monophyletic group |
|---|---|---|---|---|---|
| Archaea | Aeropyrum pernix K1_1 | 1700 | | | |
| Archaea | Archaeoglobus fulgidus DSM 4304_1 | 2420 | | | |
| Archaea | Archaeoglobus profundus DSM 5631_1 | 1819 | | | |
| Archaea | Caldivirga maquilingensis IC-167_1 | 1963 | | | |
| Archaea | Candidatus Korarchaeum cryptofilum OPF8_1 | 1602 | | | |
| Archaea | Candidatus Methanoregula boonei 6A8_1 | 2450 | | | |
| Archaea | Candidatus Methanosphaerula palustris E1-9c_1 | 2655 | | | |
| Archaea | Cenarchaeum symbiosum A_1 | 2017 | | | |
| Archaea | Desulfurococcus kamchatkensis 1221n_1 | 1471 | | | |
| Archaea | Haloarcula marismortui ATCC 43049_1 | 3131 | | | |
| Archaea | Haloarcula marismortui ATCC 43049_2 | 281 | | | |
| Archaea | Halobacterium sp. NRC-1_1 | 2075 | | | |
| Archaea | Halomicrobium mukohataei DSM 12286_1 | 3173 | | | |
| Archaea | Haloquadratum walsbyi DSM 16790_1 | 2610 | | | |
| Archaea | Halorhabdus utahensis DSM 12940_1 | 2998 | | | |
| Archaea | Halorubrum lacusprofundi ATCC 49239_1 | 473 | | | |
| Archaea | Halorubrum lacusprofundi ATCC 49239_2 | 2711 | | | |
| Archaea | Haloterrigena turkmenica DSM 5511_1 | 3739 | | | |
| Archaea | Hyperthermus butylicus DSM 5456_1 | 1602 | | | |
| Archaea | Ignicoccus hospitalis KIN4/I_1 | 1434 | | | |
| Archaea | Metallosphaera sedula DSM 5348_1 | 2256 | | | |
| Archaea | Methanobrevibacter ruminantium M1_1 | 2217 | | | |
| Archaea | Methanocaldococcus fervens AG86_1 | 1620 | | | |
| Archaea | Methanocella paludicola SANAE_1 | 3004 | | | |
| Archaea | Methanococcoides burtonii DSM 6242_1 | 2273 | | | |
| Archaea | Methanococcus aeolicus Nankai-3_1 | 1490 | | | |
| Archaea | Methanococcus maripaludis C6_1 | 1826 | | | |
| Archaea | Methanococcus vannielii SB_1 | 1678 | | | |
| Archaea | Methanocorpusculum labreanum Z_1 | 1739 | | | |
| Archaea | Methanoculleus marisnigri JR1_1 | 2489 | | | |
| Archaea | Methanopyrus kandleri AV19_1 | 1687 | | | |
| Archaea | Methanosaeta thermophila PT_1 | 1696 | | | |
| Archaea | Methanosarcina acetivorans C2A_1 | 4540 | | | |
| Archaea | Methanosarcina barkeri str. Fusaro_1 | 3606 | | | |
| Archaea | Methanosarcina mazei Go1_1 | 3368 | | | |
| Archaea | Methanosphaera stadtmanae DSM 3091_1 | 1534 | | | |
| Archaea | Methanospirillum hungatei JF-1_1 | 3139 | | | |
| Archaea | Nanoarchaeum equitans Kin4-M_1 | 536 | | | |
| Archaea | Natronomonas pharaonis DSM 2160_1 | 2659 | | | |
| Archaea | Nitrosopumilus maritimus SCM1_1 | 1795 | | | |
| Archaea | Picrophilus torridus DSM 9790_1 | 1535 | | | |
| Archaea | Pyrobaculum aerophilum str. IM2_1 | 2603 | | | |
| Archaea | Pyrobaculum arsenaticum DSM 13514_1 | 2299 | | | |
| Archaea | Pyrococcus abyssi GE5_1 | 1780 | | | |
| Archaea | Pyrococcus furiosus DSM 3638_1 | 2125 | | | |
| Archaea | Pyrococcus horikoshii OT3_1 | 1955 | | | |
| Archaea | Staphylothermus marinus F1_1 | 1570 | | | |
| Archaea | Sulfolobus acidocaldarius DSM 639_1 | 2223 | | | |
| Archaea | Sulfolobus solfataricus P2_1 | 2977 | | | |
| Archaea | Thermococcus gammatolerans EJ3_1 | 2156 | | | |
| Archaea | Thermofilum pendens Hrk 5_1 | 1824 | | | |
| Archaea | Thermoplasma acidophilum DSM 1728_1 | 1482 | | | |
| Archaea | Thermoplasma volcanium GSS1_1 | 1499 | | | |
| Archaea | Thermoproteus neutrophilus V24Sta_1 | 1966 | | | |
| Bacteria | Acholeplasma laidlawii PG-8A_1 | 1380 | | | |
| Bacteria | Acidobacterium capsulatum ATCC 51196_1 | 3377 | | | |
| Bacteria | Akkermansia muciniphila ATCC BAA-835_1 | 2138 | | | |
| Bacteria | Alicyclobacillus acidocaldarius subsp. acidocaldarius DSM 446_1 | 2888 | | | |
| Bacteria | Aquifex aeolicus VF5_1 | 1529 | | | |
| Bacteria | Bacillus cereus Q1_1 | 5191 | | | |
| Bacteria | Bacteroides fragilis YCH46_1 | 4578 | | | |
| Bacteria | Bdellovibrio bacteriovorus HD100_1 | 3587 | | | |
| Bacteria | Bordetella pertussis Tohama I_1 | 3436 | | | |
| Bacteria | Campylobacter jejuni subsp. jejuni 81-176_1 | 1653 | | | |
| Bacteria | Candidatus Cloacamonas acidaminovorans_1 | 1815 | | | |
| Bacteria | Candidatus Endomicrobium sp. Rs-D17_1 | 776 | | | |
| Bacteria | Carboxydothermus hydrogenoformans Z-2901_1 | 2620 | | | |
| Bacteria | Chlamydia trachomatis 434/Bu_1 | 874 | | | |
| Bacteria | Chlorobium chlorochromatii CaD3_1 | 2002 | | | |
| Bacteria | Chloroflexus aurantiacus J-10-fl_1 | 3853 | | | |
| Bacteria | Clostridium acetobutylicum ATCC 824_1 | 3671 | | | |
| Bacteria | Clostridium perfringens SM101_1 | 2546 | | | |
| Bacteria | Corynebacterium glutamicum ATCC 13032_1 | 3057 | | | |
| Bacteria | Coxiella burnetii RSA 493_1 | 1817 | | | |
| Bacteria | Cupriavidus taiwanensis_1 | 3135 | | | |
| Bacteria | Cupriavidus taiwanensis_2 | 2242 | | | |
| Bacteria | Cyanothece sp. ATCC 51142_1 | 4761 | | | |
| Bacteria | Cyanothece sp. ATCC 51142_2 | 449 | | | |
| Bacteria | Dehalococcoides ethenogenes 195_1 | 1580 | | | |
| Bacteria | Deinococcus radiodurans R1_1 | 2629 | | | |

| Type | Name | Number of genes | Baltimore class | Nucleic Acid | Monophyletic group |
|---|---|---|---|---|---|
| Bacteria | Deinococcus radiodurans R1_2 | 368 | | | |
| Bacteria | Dictyoglomus thermophilum H-6-12_1 | 1912 | | | |
| Bacteria | Elusimicrobium minutum Pei191_1 | 1529 | | | |
| Bacteria | Flavobacterium psychrophilum JIP02/86_1 | 2412 | | | |
| Bacteria | Fusobacterium nucleatum subsp. nucleatum ATCC 25586_1 | 2063 | | | |
| Bacteria | Gemmata obscuriglobus UQM 2246_1 | 7989 | | | |
| Bacteria | Gemmatimonas aurantiaca T-27_1 | 3935 | | | |
| Bacteria | Gloeobacter violaceus PCC 7421_1 | 4430 | | | |
| Bacteria | Leptospira interrogans serovar Lai str. 56601_1 | 3409 | | | |
| Bacteria | Leptospira interrogans serovar Lai str. 56601_2 | 293 | | | |
| Bacteria | Magnetococcus sp. MC-1_1 | 3716 | | | |
| Bacteria | Nostoc punctiforme PCC 73102_1 | 6086 | | | |
| Bacteria | Opitutus terrae PB90-1_1 | 4612 | | | |
| Bacteria | Pedobacter heparinus DSM 2366_1 | 4252 | | | |
| Bacteria | Pirellula staleyi DSM 6068_1 | 4717 | | | |
| Bacteria | Prochlorococcus marinus str. AS9601_1 | 1920 | | | |
| Bacteria | Psychrobacter arcticus 273-4_1 | 2120 | | | |
| Bacteria | Rhizobium leguminosarum bv. trifolii WSM1325_1 | 4565 | | | |
| Bacteria | Rhodopirellula baltica SH 1_1 | 7325 | | | |
| Bacteria | Rhodospirillum rubrum ATCC 11170_1 | 3791 | | | |
| Bacteria | Rickettsia rickettsii str. Iowa_1 | 1383 | | | |
| Bacteria | Shewanella putrefaciens CN-32_1 | 3972 | | | |
| Bacteria | Solibacter usitatus Ellin6076_1 | 7826 | | | |
| Bacteria | Synechococcus elongatus PCC 6301_1 | 2526 | | | |
| Bacteria | Thermanaerovibrio acidaminovorans DSM 6589_1 | 1738 | | | |
| Bacteria | Thermoanaerobacter tengcongensis MB4_1 | 2588 | | | |
| Bacteria | Thermobaculum terrenum ATCC BAA-798_1 | 1859 | | | |
| Bacteria | Thermobaculum terrenum ATCC BAA-798_2 | 973 | | | |
| Bacteria | Thermodesulfovibrio yellowstonii DSM 11347_1 | 2033 | | | |
| Bacteria | Thermomicrobium roseum DSM 5159_1 | 1917 | | | |
| Bacteria | Thermotoga maritima MSB8_1 | 1858 | | | |
| Bacteria | Thermus thermophilus HB8_1 | 1972 | | | |
| Eukaryota | Arabidopsis thaliana_1 | 27225 | | | |
| Eukaryota | Chlorella variabilis_1 | 9780 | | | |
| Eukaryota | Dictyostelium discoideum AX4 | 13267 | | | |
| Eukaryota | Dictyostelium purpureum | 12399 | | | |
| Eukaryota | Encephalitozoon intestinalis ATCC 50506_1 | 1833 | | | |
| Eukaryota | Entamoeba dispar SAW760 | 8798 | | | |
| Eukaryota | Entamoeba histolytica HM-1:IMSS | 8150 | | | |
| Eukaryota | Giardia lamblia ATCC 50803_1 | 7364 | | | |
| Eukaryota | Homo sapiens_1 | 21973 | | | |
| Eukaryota | Naegleria gruberi_1 | 15711 | | | |
| Eukaryota | Phytophthora infestans T30-4_1 | 17797 | | | |
| Eukaryota | Plasmodium knowlesi strain H_1 | 5102 | | | |
| Eukaryota | Saccharomyces cerevisiae_10 | 357 | | | |
| Eukaryota | Saccharomyces cerevisiae_11 | 312 | | | |
| Eukaryota | Saccharomyces cerevisiae_12 | 508 | | | |
| Eukaryota | Saccharomyces cerevisiae_13 | 460 | | | |
| Eukaryota | Saccharomyces cerevisiae_14 | 393 | | | |
| Eukaryota | Saccharomyces cerevisiae_15 | 536 | | | |
| Eukaryota | Saccharomyces cerevisiae_16 | 464 | | | |
| Eukaryota | Saccharomyces cerevisiae_2 | 406 | | | |
| Eukaryota | Saccharomyces cerevisiae_3 | 160 | | | |
| Eukaryota | Saccharomyces cerevisiae_4 | 755 | | | |
| Eukaryota | Saccharomyces cerevisiae_5 | 276 | | | |
| Eukaryota | Saccharomyces cerevisiae_6 | 126 | | | |
| Eukaryota | Saccharomyces cerevisiae_7 | 526 | | | |
| Eukaryota | Saccharomyces cerevisiae_8 | 281 | | | |
| Eukaryota | Saccharomyces cerevisiae_9 | 207 | | | |
| Eukaryota | Tetrahymena thermophila SB210_1 | 24725 | | | |
| Eukaryota | Trypanosoma cruzi_1 | 19607 | | | |
| Plasmid | 'Nostoc azollae' 0708_1 | 51 | | | |
| Plasmid | 'Nostoc azollae' 0708_2 | 11 | | | |
| Plasmid | Acaryochloris marina MBIC11017_1 | 382 | | | |
| Plasmid | Acaryochloris marina MBIC11017_2 | 408 | | | |
| Plasmid | Acaryochloris marina MBIC11017_3 | 362 | | | |
| Plasmid | Acaryochloris marina MBIC11017_4 | 273 | | | |
| Plasmid | Acaryochloris marina MBIC11017_5 | 222 | | | |
| Plasmid | Acaryochloris marina MBIC11017_6 | 188 | | | |
| Plasmid | Acaryochloris marina MBIC11017_7 | 173 | | | |
| Plasmid | Acaryochloris marina MBIC11017_8 | 117 | | | |
| Plasmid | Acaryochloris marina MBIC11017_9 | 4 | | | |
| Plasmid | Acetobacter pasteurianus IFO 3283-01_1 | 177 | | | |
| Plasmid | Acetobacter pasteurianus IFO 3283-01_2 | 174 | | | |
| Plasmid | Acetobacter pasteurianus IFO 3283-01_3 | 63 | | | |
| Plasmid | Acetobacter pasteurianus IFO 3283-01_4 | 3 | | | |
| Plasmid | Acetobacter pasteurianus IFO 3283-01_5 | 2 | | | |
| Plasmid | Acetobacter pasteurianus IFO 3283-01_6 | 2 | | | |
| Plasmid | Achromobacter xylosoxidans A8_1 | 104 | | | |
| Plasmid | Achromobacter xylosoxidans A8_2 | 252 | | | |

| Type | Name | Number of genes | Baltimore class | Nucleic Acid | Monophyletic group |
|---|---|---|---|---|---|
| Plasmid | Acidiphilium cryptum JF-5_1 | 171 | | | |
| Plasmid | Acidiphilium cryptum JF-5_2 | 169 | | | |
| Plasmid | Acidiphilium cryptum JF-5_3 | 77 | | | |
| Plasmid | Acidiphilium cryptum JF-5_4 | 32 | | | |
| Plasmid | Acidiphilium cryptum JF-5_5 | 28 | | | |
| Plasmid | Acidiphilium cryptum JF-5_6 | 9 | | | |
| Plasmid | Acidiphilium cryptum JF-5_7 | 5 | | | |
| Plasmid | Acidiphilium cryptum JF-5_8 | 5 | | | |
| Plasmid | Acidiphilium multivorum AIU301_1 | 284 | | | |
| Plasmid | Acidiphilium multivorum AIU301_2 | 61 | | | |
| Plasmid | Acidiphilium multivorum AIU301_3 | 19 | | | |
| Plasmid | Acidiphilium multivorum AIU301_4 | 14 | | | |
| Plasmid | Acidiphilium multivorum AIU301_5 | 1 | | | |
| Plasmid | Acidiphilium multivorum AIU301_6 | 69 | | | |
| Plasmid | Acidiphilium multivorum AIU301_7 | 44 | | | |
| Plasmid | Acidiphilium multivorum AIU301_8 | 8 | | | |
| Plasmid | Acidobacterium sp. MP5ACTX9_1 | 286 | | | |
| Plasmid | Acidobacterium sp. MP5ACTX9_2 | 166 | | | |
| Plasmid | Acidobacterium sp. MP5ACTX9_3 | 91 | | | |
| Plasmid | Acidobacterium sp. MP5ACTX9_4 | 110 | | | |
| Plasmid | Acidobacterium sp. MP5ACTX9_5 | 191 | | | |
| Plasmid | Acidovorax sp. JS42_1 | 84 | | | |
| Plasmid | Acidovorax sp. JS42_2 | 64 | | | |
| Plasmid | Acinetobacter baumannii AB0057_1 | 11 | | | |
| Plasmid | Acinetobacter baumannii ACICU_1 | 28 | | | |
| Plasmid | Acinetobacter baumannii ACICU_2 | 64 | | | |
| Plasmid | Acinetobacter baumannii ATCC 17978_1 | 11 | | | |
| Plasmid | Acinetobacter baumannii ATCC 17978_2 | 5 | | | |
| Plasmid | Acinetobacter baumannii AYE_1 | 7 | | | |
| Plasmid | Acinetobacter baumannii AYE_2 | 11 | | | |
| Plasmid | Acinetobacter baumannii AYE_3 | 5 | | | |
| Plasmid | Acinetobacter baumannii AYE_4 | 82 | | | |
| Plasmid | Acinetobacter baumannii SDF_1 | 8 | | | |
| Plasmid | Acinetobacter baumannii SDF_2 | 30 | | | |
| Plasmid | Acinetobacter baumannii SDF_3 | 24 | | | |
| Plasmid | Actinobacillus pleuropneumoniae serovar 7 str. AP76_1 | 2 | | | |
| Plasmid | Actinobacillus pleuropneumoniae serovar 7 str. AP76_2 | 4 | | | |
| Plasmid | Actinobacillus pleuropneumoniae serovar 7 str. AP76_3 | 5 | | | |
| Plasmid | Aeromonas salmonicida subsp. salmonicida A449_1 | 8 | | | |
| Plasmid | Aeromonas salmonicida subsp. salmonicida A449_2 | 9 | | | |
| Plasmid | Aeromonas salmonicida subsp. salmonicida A449_3 | 7 | | | |
| Plasmid | Aeromonas salmonicida subsp. salmonicida A449_4 | 173 | | | |
| Plasmid | Aeromonas salmonicida subsp. salmonicida A449_5 | 154 | | | |
| Plasmid | Aggregatibacter actinomycetemcomitans D11S-1_1 | 41 | | | |
| Plasmid | Aggregatibacter actinomycetemcomitans_1 | 43 | | | |
| Plasmid | Agrobacterium radiobacter K84_1 | 366 | | | |
| Plasmid | Agrobacterium radiobacter K84_2 | 176 | | | |
| Plasmid | Agrobacterium radiobacter K84_3 | 35 | | | |
| Plasmid | Agrobacterium sp. H13-3_1 | 651 | | | |
| Plasmid | Agrobacterium tumefaciens str. C58_1 | 542 | | | |
| Plasmid | Agrobacterium tumefaciens str. C58_2 | 197 | | | |
| Plasmid | Agrobacterium vitis S4_1 | 529 | | | |
| Plasmid | Agrobacterium vitis S4_2 | 205 | | | |
| Plasmid | Agrobacterium vitis S4_3 | 178 | | | |
| Plasmid | Agrobacterium vitis S4_4 | 82 | | | |
| Plasmid | Agrobacterium vitis S4_5 | 107 | | | |
| Plasmid | Alicycliphilus denitrificans BC_1 | 125 | | | |
| Plasmid | Alicycliphilus denitrificans BC_2 | 87 | | | |
| Plasmid | Alicycliphilus denitrificans K601_1 | 89 | | | |
| Plasmid | Alicyclobacillus acidocaldarius subsp. acidocaldarius DSM 446_1 | 98 | | | |
| Plasmid | Alicyclobacillus acidocaldarius subsp. acidocaldarius DSM 446_2 | 92 | | | |
| Plasmid | Alicyclobacillus acidocaldarius subsp. acidocaldarius DSM 446_3 | 6 | | | |
| Plasmid | Aliivibrio salmonicida LFI1238_1 | 72 | | | |
| Plasmid | Aliivibrio salmonicida LFI1238_2 | 29 | | | |
| Plasmid | Aliivibrio salmonicida LFI1238_3 | 3 | | | |
| Plasmid | Aliivibrio salmonicida LFI1238_4 | 3 | | | |
| Plasmid | Allochromatium vinosum DSM 180_1 | 119 | | | |
| Plasmid | Allochromatium vinosum DSM 180_2 | 48 | | | |
| Plasmid | Ammonifex degensii KC4_1 | 28 | | | |
| Plasmid | Amycolicicoccus subflavus DQS3-9A1_1 | 27 | | | |
| Plasmid | Amycolicicoccus subflavus DQS3-9A1_2 | 121 | | | |
| Plasmid | Anabaena variabilis ATCC 29413_1 | 344 | | | |
| Plasmid | Anabaena variabilis ATCC 29413_2 | 31 | | | |
| Plasmid | Anabaena variabilis ATCC 29413_3 | 243 | | | |
| Plasmid | Anaerococcus prevotii DSM 20548_1 | 104 | | | |
| Plasmid | Aquifex aeolicus VF5_1 | 31 | | | |
| Plasmid | Archaeoglobus profundus DSM 5631_1 | 4 | | | |
| Plasmid | Arthrobacter arilaitensis Re117_1 | 13 | | | |
| Plasmid | Arthrobacter arilaitensis Re117_2 | 47 | | | |
| Plasmid | Arthrobacter aurescens TC1_1 | 292 | | | |

| Type | Name | Number of genes | Baltimore class | Nucleic Acid | Monophyletic group |
|---|---|---|---|---|---|
| Plasmid | Arthrobacter aurescens TC1_2 | 254 | | | |
| Plasmid | Arthrobacter chlorophenolicus A6_1 | 554 | | | |
| Plasmid | Arthrobacter chlorophenolicus A6_2 | 151 | | | |
| Plasmid | Arthrobacter phenanthrenivorans Sphe3_1 | 189 | | | |
| Plasmid | Arthrobacter phenanthrenivorans Sphe3_2 | 99 | | | |
| Plasmid | Arthrobacter sp. FB24_1 | 158 | | | |
| Plasmid | Arthrobacter sp. FB24_2 | 110 | | | |
| Plasmid | Arthrobacter sp. FB24_3 | 96 | | | |
| Plasmid | Aster yellows witches'-broom phytoplasma AYWB_1 | 5 | | | |
| Plasmid | Aster yellows witches'-broom phytoplasma AYWB_2 | 4 | | | |
| Plasmid | Aster yellows witches'-broom phytoplasma AYWB_3 | 7 | | | |
| Plasmid | Aster yellows witches'-broom phytoplasma AYWB_4 | 6 | | | |
| Plasmid | Asticcacaulis excentricus CB 48_1 | 172 | | | |
| Plasmid | Asticcacaulis excentricus CB 48_2 | 140 | | | |
| Plasmid | Azoarcus sp. EbN1_1 | 272 | | | |
| Plasmid | Azoarcus sp. EbN1_2 | 194 | | | |
| Plasmid | Azospirillum sp. B510_1 | 1131 | | | |
| Plasmid | Azospirillum sp. B510_2 | 631 | | | |
| Plasmid | Azospirillum sp. B510_3 | 533 | | | |
| Plasmid | Azospirillum sp. B510_4 | 519 | | | |
| Plasmid | Azospirillum sp. B510_5 | 415 | | | |
| Plasmid | Azospirillum sp. B510_6 | 187 | | | |
| Plasmid | Bacillus anthracis CI_1 | 210 | | | |
| Plasmid | Bacillus anthracis CI_2 | 109 | | | |
| Plasmid | Bacillus anthracis CI_3 | 18 | | | |
| Plasmid | Bacillus anthracis str. 'Ames Ancestor'_1 | 178 | | | |
| Plasmid | Bacillus anthracis str. 'Ames Ancestor'_2 | 98 | | | |
| Plasmid | Bacillus anthracis str. A0248_1 | 89 | | | |
| Plasmid | Bacillus anthracis str. A0248_2 | 162 | | | |
| Plasmid | Bacillus anthracis str. CDC 684_1 | 117 | | | |
| Plasmid | Bacillus anthracis str. CDC 684_2 | 206 | | | |
| Plasmid | Bacillus cereus 03BB102_1 | 208 | | | |
| Plasmid | Bacillus cereus AH187_1 | 22 | | | |
| Plasmid | Bacillus cereus AH187_2 | 273 | | | |
| Plasmid | Bacillus cereus AH187_3 | 59 | | | |
| Plasmid | Bacillus cereus AH187_4 | 5 | | | |
| Plasmid | Bacillus cereus AH820_1 | 11 | | | |
| Plasmid | Bacillus cereus AH820_2 | 5 | | | |
| Plasmid | Bacillus cereus AH820_3 | 320 | | | |
| Plasmid | Bacillus cereus ATCC 10987_1 | 241 | | | |
| Plasmid | Bacillus cereus ATCC 14579_1 | 21 | | | |
| Plasmid | Bacillus cereus E33L_1 | 430 | | | |
| Plasmid | Bacillus cereus E33L_2 | 5 | | | |
| Plasmid | Bacillus cereus E33L_3 | 54 | | | |
| Plasmid | Bacillus cereus E33L_4 | 8 | | | |
| Plasmid | Bacillus cereus E33L_5 | 10 | | | |
| Plasmid | Bacillus cereus G9842_1 | 118 | | | |
| Plasmid | Bacillus cereus G9842_2 | 251 | | | |
| Plasmid | Bacillus cereus Q1_1 | 69 | | | |
| Plasmid | Bacillus cereus Q1_2 | 228 | | | |
| Plasmid | Bacillus cereus subsp. cytotoxis NVH 391-98_1 | 11 | | | |
| Plasmid | Bacillus megaterium QM B1551_1 | 60 | | | |
| Plasmid | Bacillus megaterium QM B1551_2 | 8 | | | |
| Plasmid | Bacillus megaterium QM B1551_3 | 13 | | | |
| Plasmid | Bacillus megaterium QM B1551_4 | 38 | | | |
| Plasmid | Bacillus megaterium QM B1551_5 | 174 | | | |
| Plasmid | Bacillus megaterium QM B1551_6 | 94 | | | |
| Plasmid | Bacillus megaterium QM B1551_7 | 109 | | | |
| Plasmid | Bacillus pseudofirmus OF4_1 | 292 | | | |
| Plasmid | Bacillus pseudofirmus OF4_2 | 122 | | | |
| Plasmid | Bacillus thuringiensis BMB171_1 | 271 | | | |
| Plasmid | Bacillus thuringiensis serovar konkukian str. 97-27_1 | 80 | | | |
| Plasmid | Bacillus thuringiensis str. Al Hakam_1 | 62 | | | |
| Plasmid | Bacillus weihenstephanensis KBAB4_1 | 289 | | | |
| Plasmid | Bacillus weihenstephanensis KBAB4_2 | 63 | | | |
| Plasmid | Bacillus weihenstephanensis KBAB4_3 | 75 | | | |
| Plasmid | Bacillus weihenstephanensis KBAB4_4 | 71 | | | |
| Plasmid | Bacteroides fragilis NCTC 9343_1 | 47 | | | |
| Plasmid | Bacteroides fragilis YCH46_1 | 47 | | | |
| Plasmid | Bacteroides salanitronis DSM 18170_1 | 56 | | | |
| Plasmid | Bacteroides salanitronis DSM 18170_2 | 8 | | | |
| Plasmid | Bacteroides salanitronis DSM 18170_3 | 24 | | | |
| Plasmid | Bacteroides thetaiotaomicron VPI-5482_1 | 38 | | | |
| Plasmid | Bartonella grahamii as4aup_1 | 31 | | | |
| Plasmid | Bartonella tribocorum CIP 105476_1 | 18 | | | |
| Plasmid | Beijerinckia indica subsp. indica ATCC 9039_1 | 39 | | | |
| Plasmid | Beijerinckia indica subsp. indica ATCC 9039_2 | 176 | | | |
| Plasmid | Bifidobacterium longum DJO10A_1 | 10 | | | |
| Plasmid | Bifidobacterium longum DJO10A_2 | 3 | | | |
| Plasmid | Bifidobacterium longum NCC2705_1 | 2 | | | |

| Type | Name | Number of genes | Baltimore class | Nucleic Acid | Monophyletic group |
|---|---|---|---|---|---|
| Plasmid | Bifidobacterium longum subsp. infantis 157F_1 | 5 | | | |
| Plasmid | Bifidobacterium longum subsp. infantis 157F_2 | 3 | | | |
| Plasmid | Blattabacterium sp. (Periplaneta americana) str. BPLAN_1 | 4 | | | |
| Plasmid | Borrelia afzelii PKo_1 | 43 | | | |
| Plasmid | Borrelia afzelii PKo_2 | 28 | | | |
| Plasmid | Borrelia afzelii PKo_3 | 75 | | | |
| Plasmid | Borrelia afzelii PKo_4 | 59 | | | |
| Plasmid | Borrelia afzelii PKo_5 | 36 | | | |
| Plasmid | Borrelia afzelii PKo_6 | 55 | | | |
| Plasmid | Borrelia afzelii PKo_7 | 34 | | | |
| Plasmid | Borrelia afzelii PKo_8 | 29 | | | |
| Plasmid | Borrelia burgdorferi B31_1 | 42 | | | |
| Plasmid | Borrelia burgdorferi B31_10 | 6 | | | |
| Plasmid | Borrelia burgdorferi B31_11 | 23 | | | |
| Plasmid | Borrelia burgdorferi B31_12 | 29 | | | |
| Plasmid | Borrelia burgdorferi B31_13 | 26 | | | |
| Plasmid | Borrelia burgdorferi B31_14 | 32 | | | |
| Plasmid | Borrelia burgdorferi B31_15 | 37 | | | |
| Plasmid | Borrelia burgdorferi B31_16 | 43 | | | |
| Plasmid | Borrelia burgdorferi B31_17 | 51 | | | |
| Plasmid | Borrelia burgdorferi B31_18 | 48 | | | |
| Plasmid | Borrelia burgdorferi B31_19 | 76 | | | |
| Plasmid | Borrelia burgdorferi B31_2 | 45 | | | |
| Plasmid | Borrelia burgdorferi B31_20 | 29 | | | |
| Plasmid | Borrelia burgdorferi B31_21 | 11 | | | |
| Plasmid | Borrelia burgdorferi B31_3 | 44 | | | |
| Plasmid | Borrelia burgdorferi B31_4 | 42 | | | |
| Plasmid | Borrelia burgdorferi B31_5 | 44 | | | |
| Plasmid | Borrelia burgdorferi B31_6 | 43 | | | |
| Plasmid | Borrelia burgdorferi B31_7 | 35 | | | |
| Plasmid | Borrelia burgdorferi B31_8 | 11 | | | |
| Plasmid | Borrelia burgdorferi B31_9 | 72 | | | |
| Plasmid | Borrelia burgdorferi ZS7_1 | 60 | | | |
| Plasmid | Borrelia burgdorferi ZS7_10 | 22 | | | |
| Plasmid | Borrelia burgdorferi ZS7_11 | 14 | | | |
| Plasmid | Borrelia burgdorferi ZS7_12 | 17 | | | |
| Plasmid | Borrelia burgdorferi ZS7_13 | 55 | | | |
| Plasmid | Borrelia burgdorferi ZS7_14 | 21 | | | |
| Plasmid | Borrelia burgdorferi ZS7_2 | 35 | | | |
| Plasmid | Borrelia burgdorferi ZS7_3 | 25 | | | |
| Plasmid | Borrelia burgdorferi ZS7_4 | 36 | | | |
| Plasmid | Borrelia burgdorferi ZS7_5 | 39 | | | |
| Plasmid | Borrelia burgdorferi ZS7_6 | 40 | | | |
| Plasmid | Borrelia burgdorferi ZS7_7 | 23 | | | |
| Plasmid | Borrelia burgdorferi ZS7_8 | 31 | | | |
| Plasmid | Borrelia burgdorferi ZS7_9 | 13 | | | |
| Plasmid | Borrelia duttonii Ly_1 | 8 | | | |
| Plasmid | Borrelia duttonii Ly_10 | 45 | | | |
| Plasmid | Borrelia duttonii Ly_11 | 21 | | | |
| Plasmid | Borrelia duttonii Ly_12 | 12 | | | |
| Plasmid | Borrelia duttonii Ly_13 | 34 | | | |
| Plasmid | Borrelia duttonii Ly_14 | 20 | | | |
| Plasmid | Borrelia duttonii Ly_15 | 22 | | | |
| Plasmid | Borrelia duttonii Ly_16 | 35 | | | |
| Plasmid | Borrelia duttonii Ly_2 | 13 | | | |
| Plasmid | Borrelia duttonii Ly_3 | 36 | | | |
| Plasmid | Borrelia duttonii Ly_4 | 134 | | | |
| Plasmid | Borrelia duttonii Ly_5 | 19 | | | |
| Plasmid | Borrelia duttonii Ly_6 | 20 | | | |
| Plasmid | Borrelia duttonii Ly_7 | 18 | | | |
| Plasmid | Borrelia duttonii Ly_8 | 28 | | | |
| Plasmid | Borrelia duttonii Ly_9 | 20 | | | |
| Plasmid | Borrelia garinii PBi_1 | 26 | | | |
| Plasmid | Borrelia garinii PBi_2 | 74 | | | |
| Plasmid | Borrelia garinii PBi_3 | 338 | | | |
| Plasmid | Borrelia recurrentis A1_1 | 91 | | | |
| Plasmid | Borrelia recurrentis A1_2 | 17 | | | |
| Plasmid | Borrelia recurrentis A1_3 | 17 | | | |
| Plasmid | Borrelia recurrentis A1_4 | 15 | | | |
| Plasmid | Borrelia recurrentis A1_5 | 19 | | | |
| Plasmid | Borrelia recurrentis A1_6 | 28 | | | |
| Plasmid | Borrelia recurrentis A1_7 | 3 | | | |
| Plasmid | Brachyspira hyodysenteriae WA1_1 | 31 | | | |
| Plasmid | Bradyrhizobium sp. BTAi1_1 | 228 | | | |
| Plasmid | Buchnera aphidicola (Cinara cedri)_1 | 5 | | | |
| Plasmid | Buchnera aphidicola str. APS (Acyrthosiphon pisum)_1 | 3 | | | |
| Plasmid | Buchnera aphidicola str. APS (Acyrthosiphon pisum)_2 | 7 | | | |
| Plasmid | Buchnera aphidicola str. Bp (Baizongia pistaciae)_1 | 3 | | | |
| Plasmid | Burkholderia ambifaria AMMD_1 | 45 | | | |
| Plasmid | Burkholderia ambifaria MC40-6_1 | 274 | | | |

| Type | Name | Number of genes | Baltimore class | Nucleic Acid | Monophyletic group |
|---|---|---|---|---|---|
| Plasmid | Burkholderia cenocepacia HI2424_1 | 156 | | | |
| Plasmid | Burkholderia cenocepacia J2315_1 | 93 | | | |
| Plasmid | Burkholderia gladioli BSR3_1 | 104 | | | |
| Plasmid | Burkholderia gladioli BSR3_2 | 94 | | | |
| Plasmid | Burkholderia gladioli BSR3_3 | 189 | | | |
| Plasmid | Burkholderia gladioli BSR3_4 | 319 | | | |
| Plasmid | Burkholderia glumae BGR1_1 | 97 | | | |
| Plasmid | Burkholderia glumae BGR1_2 | 106 | | | |
| Plasmid | Burkholderia glumae BGR1_3 | 102 | | | |
| Plasmid | Burkholderia glumae BGR1_4 | 102 | | | |
| Plasmid | Burkholderia multivorans ATCC 17616_1 | 138 | | | |
| Plasmid | Burkholderia multivorans ATCC 17616_2 | 131 | | | |
| Plasmid | Burkholderia phymatum STM815_1 | 1626 | | | |
| Plasmid | Burkholderia phymatum STM815_2 | 449 | | | |
| Plasmid | Burkholderia phytofirmans PsJN_1 | 167 | | | |
| Plasmid | Burkholderia rhizoxinica HKI 454_1 | 203 | | | |
| Plasmid | Burkholderia sp. CCGE1002_1 | 406 | | | |
| Plasmid | Burkholderia vietnamiensis G4_1 | 111 | | | |
| Plasmid | Burkholderia vietnamiensis G4_2 | 263 | | | |
| Plasmid | Burkholderia vietnamiensis G4_3 | 107 | | | |
| Plasmid | Burkholderia vietnamiensis G4_4 | 249 | | | |
| Plasmid | Burkholderia vietnamiensis G4_5 | 403 | | | |
| Plasmid | Butyrivibrio proteoclasticus B316_1 | 424 | | | |
| Plasmid | Butyrivibrio proteoclasticus B316_2 | 198 | | | |
| Plasmid | Caldicellulosiruptor bescii DSM 6725_1 | 8 | | | |
| Plasmid | Caldicellulosiruptor bescii DSM 6725_2 | 4 | | | |
| Plasmid | Caldicellulosiruptor kristjanssonii 177R1B_1 | 17 | | | |
| Plasmid | Calditerrivibrio nitroreducens DSM 19672_1 | 55 | | | |
| Plasmid | Campylobacter concisus 13826_1 | 33 | | | |
| Plasmid | Campylobacter concisus 13826_2 | 23 | | | |
| Plasmid | Campylobacter hominis ATCC BAA-381_1 | 5 | | | |
| Plasmid | Campylobacter jejuni subsp. jejuni 81-176_1 | 53 | | | |
| Plasmid | Campylobacter jejuni subsp. jejuni 81-176_2 | 52 | | | |
| Plasmid | Campylobacter jejuni subsp. jejuni ICDCCJ07001_1 | 37 | | | |
| Plasmid | Campylobacter lari RM2100_1 | 42 | | | |
| Plasmid | Candidatus Accumulibacter phosphatis clade IIA str. UW-1_1 | 40 | | | |
| Plasmid | Candidatus Accumulibacter phosphatis clade IIA str. UW-1_2 | 40 | | | |
| Plasmid | Candidatus Accumulibacter phosphatis clade IIA str. UW-1_3 | 156 | | | |
| Plasmid | Candidatus Azobacteroides pseudotrichonymphae genomovar. CFP2_1 | 28 | | | |
| Plasmid | Candidatus Azobacteroides pseudotrichonymphae genomovar. CFP2_2 | 25 | | | |
| Plasmid | Candidatus Azobacteroides pseudotrichonymphae genomovar. CFP2_3 | 4 | | | |
| Plasmid | Candidatus Azobacteroides pseudotrichonymphae genomovar. CFP2_4 | 37 | | | |
| Plasmid | Candidatus Hamiltonella defensa 5AT (Acyrthosiphon pisum)_1 | 54 | | | |
| Plasmid | Candidatus Riesia pediculicola USDA_1 | 12 | | | |
| Plasmid | Carnobacterium sp. 17-4_1 | 54 | | | |
| Plasmid | Caulobacter sp. K31_1 | 165 | | | |
| Plasmid | Caulobacter sp. K31_2 | 212 | | | |
| Plasmid | Chlamydia muridarum Nigg_1 | 7 | | | |
| Plasmid | Chlamydia trachomatis A/HAR-13_1 | 8 | | | |
| Plasmid | Chlamydophila caviae GPIC_1 | 7 | | | |
| Plasmid | Chlamydophila felis Fe/C-56_1 | 8 | | | |
| Plasmid | Chlamydophila psittaci 6BC_1 | 8 | | | |
| Plasmid | Citrobacter koseri ATCC BAA-895_1 | 15 | | | |
| Plasmid | Citrobacter koseri ATCC BAA-895_2 | 13 | | | |
| Plasmid | Citrobacter rodentium ICC168_1 | 55 | | | |
| Plasmid | Citrobacter rodentium ICC168_2 | 55 | | | |
| Plasmid | Citrobacter rodentium ICC168_3 | 3 | | | |
| Plasmid | Clavibacter michiganensis subsp. michiganensis NCPPB 382_1 | 28 | | | |
| Plasmid | Clavibacter michiganensis subsp. michiganensis NCPPB 382_2 | 67 | | | |
| Plasmid | Clavibacter michiganensis subsp. sepedonicus_1 | 64 | | | |
| Plasmid | Clavibacter michiganensis subsp. sepedonicus_2 | 112 | | | |
| Plasmid | Clostridium acetobutylicum ATCC 824_1 | 176 | | | |
| Plasmid | Clostridium botulinum A str. ATCC 3502_1 | 18 | | | |
| Plasmid | Clostridium botulinum A3 str. Loch Maree_1 | 329 | | | |
| Plasmid | Clostridium botulinum B str. Eklund 17B_1 | 50 | | | |
| Plasmid | Clostridium botulinum B1 str. Okra_1 | 194 | | | |
| Plasmid | Clostridium botulinum Ba4 str. 657_1 | 306 | | | |
| Plasmid | Clostridium botulinum Ba4 str. 657_2 | 6 | | | |
| Plasmid | Clostridium botulinum BKT015925_1 | 221 | | | |
| Plasmid | Clostridium botulinum BKT015925_2 | 86 | | | |
| Plasmid | Clostridium botulinum BKT015925_3 | 13 | | | |
| Plasmid | Clostridium botulinum BKT015925_4 | 103 | | | |
| Plasmid | Clostridium botulinum BKT015925_5 | 53 | | | |
| Plasmid | Clostridium botulinum F str. Langeland_1 | 24 | | | |
| Plasmid | Clostridium difficile 630_1 | 11 | | | |
| Plasmid | Clostridium kluyveri DSM 555_1 | 75 | | | |
| Plasmid | Clostridium kluyveri NBRC 12016_1 | 55 | | | |
| Plasmid | Clostridium perfringens str. 13_1 | 63 | | | |
| Plasmid | Clostridium tetani E88_1 | 59 | | | |
| Plasmid | Comamonas testosteroni CNB-1_1 | 92 | | | |

| Type | Name | Number of genes | Baltimore class | Nucleic Acid | Monophyletic group |
|---|---|---|---|---|---|
| Plasmid | Corynebacterium aurimucosum ATCC 700975_1 | 20 | | | |
| Plasmid | Corynebacterium efficiens YS-314_1 | 15 | | | |
| Plasmid | Corynebacterium efficiens YS-314_2 | 41 | | | |
| Plasmid | Corynebacterium glutamicum R_1 | 28 | | | |
| Plasmid | Corynebacterium jeikeium K411_1 | 16 | | | |
| Plasmid | Coxiella burnetii CbuK_Q154_1 | 42 | | | |
| Plasmid | Coxiella burnetii Dugway 5J108-111_1 | 52 | | | |
| Plasmid | Coxiella burnetii RSA 331_1 | 45 | | | |
| Plasmid | Coxiella burnetii RSA 493_1 | 30 | | | |
| Plasmid | Cronobacter sakazakii ATCC BAA-894_1 | 38 | | | |
| Plasmid | Cronobacter sakazakii ATCC BAA-894_2 | 127 | | | |
| Plasmid | Cronobacter turicensis z3032_1 | 136 | | | |
| Plasmid | Cronobacter turicensis z3032_2 | 32 | | | |
| Plasmid | Cronobacter turicensis z3032_3 | 74 | | | |
| Plasmid | Cupriavidus metallidurans CH34_1 | 200 | | | |
| Plasmid | Cupriavidus metallidurans CH34_2 | 165 | | | |
| Plasmid | Cupriavidus metallidurans CH34_3 | 2386 | | | |
| Plasmid | Cupriavidus taiwanensis_1 | 519 | | | |
| Plasmid | Cyanothece sp. ATCC 51142_1 | 38 | | | |
| Plasmid | Cyanothece sp. ATCC 51142_2 | 25 | | | |
| Plasmid | Cyanothece sp. ATCC 51142_3 | 19 | | | |
| Plasmid | Cyanothece sp. ATCC 51142_4 | 11 | | | |
| Plasmid | Cyanothece sp. PCC 7424_1 | 26 | | | |
| Plasmid | Cyanothece sp. PCC 7424_2 | 19 | | | |
| Plasmid | Cyanothece sp. PCC 7424_3 | 16 | | | |
| Plasmid | Cyanothece sp. PCC 7424_4 | 11 | | | |
| Plasmid | Cyanothece sp. PCC 7424_5 | 175 | | | |
| Plasmid | Cyanothece sp. PCC 7424_6 | 236 | | | |
| Plasmid | Cyanothece sp. PCC 7425_1 | 160 | | | |
| Plasmid | Cyanothece sp. PCC 7425_2 | 29 | | | |
| Plasmid | Cyanothece sp. PCC 7425_3 | 172 | | | |
| Plasmid | Cyanothece sp. PCC 7822_1 | 236 | | | |
| Plasmid | Cyanothece sp. PCC 7822_2 | 28 | | | |
| Plasmid | Cyanothece sp. PCC 7822_3 | 33 | | | |
| Plasmid | Cyanothece sp. PCC 7822_4 | 546 | | | |
| Plasmid | Cyanothece sp. PCC 7822_5 | 364 | | | |
| Plasmid | Cyanothece sp. PCC 7822_6 | 13 | | | |
| Plasmid | Cyanothece sp. PCC 8801_1 | 52 | | | |
| Plasmid | Cyanothece sp. PCC 8801_2 | 44 | | | |
| Plasmid | Cyanothece sp. PCC 8801_3 | 11 | | | |
| Plasmid | Cyanothece sp. PCC 8802_1 | 58 | | | |
| Plasmid | Cyanothece sp. PCC 8802_2 | 24 | | | |
| Plasmid | Cyanothece sp. PCC 8802_3 | 29 | | | |
| Plasmid | Cyanothece sp. PCC 8802_4 | 13 | | | |
| Plasmid | Deferribacter desulfuricans SSM1_1 | 257 | | | |
| Plasmid | Deinococcus deserti VCD115_1 | 261 | | | |
| Plasmid | Deinococcus deserti VCD115_2 | 347 | | | |
| Plasmid | Deinococcus deserti VCD115_3 | 250 | | | |
| Plasmid | Deinococcus geothermalis DSM 11300_1 | 519 | | | |
| Plasmid | Deinococcus geothermalis DSM 11300_2 | 205 | | | |
| Plasmid | Deinococcus proteolyticus MRP_1 | 225 | | | |
| Plasmid | Deinococcus proteolyticus MRP_2 | 93 | | | |
| Plasmid | Deinococcus proteolyticus MRP_3 | 254 | | | |
| Plasmid | Deinococcus proteolyticus MRP_4 | 109 | | | |
| Plasmid | Deinococcus radiodurans R1_1 | 131 | | | |
| Plasmid | Deinococcus radiodurans R1_2 | 39 | | | |
| Plasmid | Desulfobacterium autotrophicum HRM2_1 | 76 | | | |
| Plasmid | Desulfohalobium retbaense DSM 5692_1 | 47 | | | |
| Plasmid | Desulfotalea psychrophila LSv54_1 | 101 | | | |
| Plasmid | Desulfotalea psychrophila LSv54_2 | 17 | | | |
| Plasmid | Desulfovibrio magneticus RS-1_1 | 10 | | | |
| Plasmid | Desulfovibrio magneticus RS-1_2 | 65 | | | |
| Plasmid | Desulfovibrio vulgaris str. Hildenborough_1 | 156 | | | |
| Plasmid | Desulfovibrio vulgaris subsp. vulgaris DP4_1 | 150 | | | |
| Plasmid | Dinoroseobacter shibae DFL 12_1 | 195 | | | |
| Plasmid | Dinoroseobacter shibae DFL 12_2 | 137 | | | |
| Plasmid | Dinoroseobacter shibae DFL 12_3 | 135 | | | |
| Plasmid | Dinoroseobacter shibae DFL 12_4 | 68 | | | |
| Plasmid | Dinoroseobacter shibae DFL 12_5 | 73 | | | |
| Plasmid | Edwardsiella tarda EIB202_1 | 53 | | | |
| Plasmid | Enterobacter cloacae subsp. cloacae ATCC 13047_1 | 274 | | | |
| Plasmid | Enterobacter cloacae subsp. cloacae ATCC 13047_2 | 124 | | | |
| Plasmid | Enterobacter sp. 638_1 | 125 | | | |
| Plasmid | Enterococcus faecalis V583_1 | 72 | | | |
| Plasmid | Enterococcus faecalis V583_2 | 18 | | | |
| Plasmid | Enterococcus faecalis V583_3 | 62 | | | |
| Plasmid | Erwinia amylovora ATCC 49946_1 | 41 | | | |
| Plasmid | Erwinia amylovora ATCC 49946_2 | 87 | | | |
| Plasmid | Erwinia amylovora CFBP1430_1 | 29 | | | |
| Plasmid | Erwinia billingiae Eb661_1 | 220 | | | |

| Type | Name | Number of genes | Baltimore class | Nucleic Acid | Monophyletic group |
|---|---|---|---|---|---|
| Plasmid | Erwinia pyrifoliae Ep1/96_1 | 37 | | | |
| Plasmid | Erwinia pyrifoliae Ep1/96_2 | 4 | | | |
| Plasmid | Erwinia pyrifoliae Ep1/96_3 | 5 | | | |
| Plasmid | Erwinia pyrifoliae Ep1/96_4 | 6 | | | |
| Plasmid | Erwinia tasmaniensis Et1/99_1 | 39 | | | |
| Plasmid | Erwinia tasmaniensis Et1/99_2 | 7 | | | |
| Plasmid | Erwinia tasmaniensis Et1/99_3 | 42 | | | |
| Plasmid | Erwinia tasmaniensis Et1/99_4 | 61 | | | |
| Plasmid | Erwinia tasmaniensis Et1/99_5 | 46 | | | |
| Plasmid | Escherichia coli APEC O1_1 | 199 | | | |
| Plasmid | Escherichia coli APEC O1_2 | 224 | | | |
| Plasmid | Escherichia coli E24377A_1 | 68 | | | |
| Plasmid | Escherichia coli E24377A_2 | 29 | | | |
| Plasmid | Escherichia coli E24377A_3 | 68 | | | |
| Plasmid | Escherichia coli E24377A_4 | 5 | | | |
| Plasmid | Escherichia coli E24377A_5 | 69 | | | |
| Plasmid | Escherichia coli E24377A_6 | 3 | | | |
| Plasmid | Escherichia coli O103:H2 str. 12009_1 | 67 | | | |
| Plasmid | Escherichia coli O111:H- str. 11128_1 | 222 | | | |
| Plasmid | Escherichia coli O111:H- str. 11128_2 | 72 | | | |
| Plasmid | Escherichia coli O111:H- str. 11128_3 | 10 | | | |
| Plasmid | Escherichia coli O111:H- str. 11128_4 | 10 | | | |
| Plasmid | Escherichia coli O111:H- str. 11128_5 | 121 | | | |
| Plasmid | Escherichia coli O127:H6 str. E2348/69_1 | 9 | | | |
| Plasmid | Escherichia coli O127:H6 str. E2348/69_2 | 90 | | | |
| Plasmid | Escherichia coli O157:H7 EDL933_1 | 99 | | | |
| Plasmid | Escherichia coli O157:H7 str. EC4115_1 | 108 | | | |
| Plasmid | Escherichia coli O157:H7 str. EC4115_2 | 54 | | | |
| Plasmid | Escherichia coli O157:H7 str. Sakai_1 | 3 | | | |
| Plasmid | Escherichia coli O157:H7 str. Sakai_2 | 85 | | | |
| Plasmid | Escherichia coli O157:H7 str. TW14359_1 | 110 | | | |
| Plasmid | Escherichia coli O26:H11 str. 11368_1 | 81 | | | |
| Plasmid | Escherichia coli O26:H11 str. 11368_2 | 6 | | | |
| Plasmid | Escherichia coli O26:H11 str. 11368_3 | 65 | | | |
| Plasmid | Escherichia coli O26:H11 str. 11368_4 | 3 | | | |
| Plasmid | Escherichia coli O55:H7 str. CB9615_1 | 107 | | | |
| Plasmid | Escherichia coli S88_1 | 135 | | | |
| Plasmid | Escherichia coli SE11_1 | 10 | | | |
| Plasmid | Escherichia coli SE11_2 | 7 | | | |
| Plasmid | Escherichia coli SE11_3 | 3 | | | |
| Plasmid | Escherichia coli SE11_4 | 112 | | | |
| Plasmid | Escherichia coli SE11_5 | 67 | | | |
| Plasmid | Escherichia coli SE11_6 | 124 | | | |
| Plasmid | Escherichia coli SMS-3-5_1 | 10 | | | |
| Plasmid | Escherichia coli SMS-3-5_2 | 4 | | | |
| Plasmid | Escherichia coli SMS-3-5_3 | 3 | | | |
| Plasmid | Escherichia coli SMS-3-5_4 | 153 | | | |
| Plasmid | Escherichia coli UMN026_1 | 49 | | | |
| Plasmid | Escherichia coli UMN026_2 | 142 | | | |
| Plasmid | Escherichia coli UTI89_1 | 145 | | | |
| Plasmid | Escherichia fergusonii ATCC 35469_1 | 55 | | | |
| Plasmid | Eubacterium eligens ATCC 27750_1 | 664 | | | |
| Plasmid | Eubacterium eligens ATCC 27750_2 | 97 | | | |
| Plasmid | Exiguobacterium sibiricum 255-15_1 | 5 | | | |
| Plasmid | Exiguobacterium sibiricum 255-15_2 | 3 | | | |
| Plasmid | Finegoldia magna ATCC 29328_1 | 182 | | | |
| Plasmid | Francisella philomiragia subsp. philomiragia ATCC 25017_1 | 4 | | | |
| Plasmid | Gallibacterium anatis UMN179_1 | 6 | | | |
| Plasmid | Geobacillus kaustophilus HTA426_1 | 42 | | | |
| Plasmid | Geobacillus sp. WCH70_1 | 9 | | | |
| Plasmid | Geobacillus sp. WCH70_2 | 31 | | | |
| Plasmid | Geobacillus sp. Y4.1MC1_1 | 64 | | | |
| Plasmid | Geobacillus sp. Y412MC52_1 | 39 | | | |
| Plasmid | Geobacillus sp. Y412MC61_1 | 39 | | | |
| Plasmid | Geobacillus thermodenitrificans NG80-2_1 | 53 | | | |
| Plasmid | Geobacter lovleyi SZ_1 | 79 | | | |
| Plasmid | Geobacter metallireducens GS-15_1 | 14 | | | |
| Plasmid | Glaciecola agarilytica 4H-3-7+YE-5_1 | 314 | | | |
| Plasmid | Gluconacetobacter diazotrophicus PAl 5_1 | 21 | | | |
| Plasmid | Gluconacetobacter diazotrophicus PAl 5_2 | 53 | | | |
| Plasmid | Gluconacetobacter diazotrophicus PAl 5_3 | 29 | | | |
| Plasmid | Gluconobacter oxydans 621H_1 | 163 | | | |
| Plasmid | Gluconobacter oxydans 621H_2 | 29 | | | |
| Plasmid | Gluconobacter oxydans 621H_3 | 18 | | | |
| Plasmid | Gluconobacter oxydans 621H_4 | 18 | | | |
| Plasmid | Gluconobacter oxydans 621H_5 | 4 | | | |
| Plasmid | Gordonia bronchialis DSM 43247_1 | 80 | | | |
| Plasmid | Haemophilus somnus 129PT_1 | 6 | | | |
| Plasmid | Halalkalicoccus jeotgali B3_1 | 362 | | | |
| Plasmid | Halalkalicoccus jeotgali B3_2 | 362 | | | |

| Type | Name | Number of genes | Baltimore class | Nucleic Acid | Monophyletic group |
|---|---|---|---|---|---|
| Plasmid | Halalkalicoccus jeotgali B3_3 | 41 | | | |
| Plasmid | Halalkalicoccus jeotgali B3_4 | 41 | | | |
| Plasmid | Halalkalicoccus jeotgali B3_5 | 24 | | | |
| Plasmid | Halalkalicoccus jeotgali B3_6 | 8 | | | |
| Plasmid | Haliscomenobacter hydrossis DSM 1100_1 | 121 | | | |
| Plasmid | Haliscomenobacter hydrossis DSM 1100_2 | 108 | | | |
| Plasmid | Haliscomenobacter hydrossis DSM 1100_3 | 72 | | | |
| Plasmid | Haloarcula marismortui ATCC 43049_1 | 36 | | | |
| Plasmid | Haloarcula marismortui ATCC 43049_2 | 42 | | | |
| Plasmid | Haloarcula marismortui ATCC 43049_3 | 40 | | | |
| Plasmid | Haloarcula marismortui ATCC 43049_4 | 51 | | | |
| Plasmid | Haloarcula marismortui ATCC 43049_5 | 131 | | | |
| Plasmid | Haloarcula marismortui ATCC 43049_6 | 166 | | | |
| Plasmid | Haloarcula marismortui ATCC 43049_7 | 362 | | | |
| Plasmid | Halobacterium salinarum R1_1 | 152 | | | |
| Plasmid | Halobacterium salinarum R1_2 | 39 | | | |
| Plasmid | Halobacterium salinarum R1_3 | 248 | | | |
| Plasmid | Halobacterium salinarum R1_4 | 200 | | | |
| Plasmid | Halobacterium sp. NRC-1_1 | 176 | | | |
| Plasmid | Halobacterium sp. NRC-1_2 | 371 | | | |
| Plasmid | Haloferax volcanii DS2_1 | 376 | | | |
| Plasmid | Haloferax volcanii DS2_2 | 5 | | | |
| Plasmid | Haloferax volcanii DS2_3 | 611 | | | |
| Plasmid | Haloferax volcanii DS2_4 | 78 | | | |
| Plasmid | Halogeometricum borinquense DSM 11551_1 | 305 | | | |
| Plasmid | Halogeometricum borinquense DSM 11551_2 | 179 | | | |
| Plasmid | Halogeometricum borinquense DSM 11551_3 | 309 | | | |
| Plasmid | Halogeometricum borinquense DSM 11551_4 | 189 | | | |
| Plasmid | Halogeometricum borinquense DSM 11551_5 | 23 | | | |
| Plasmid | Halomicrobium mukohataei DSM 12286_1 | 176 | | | |
| Plasmid | Haloquadratum walsbyi DSM 16790_1 | 36 | | | |
| Plasmid | Halorubrum lacusprofundi ATCC 49239_1 | 376 | | | |
| Plasmid | Haloterrigena turkmenica DSM 5511_1 | 593 | | | |
| Plasmid | Haloterrigena turkmenica DSM 5511_2 | 329 | | | |
| Plasmid | Haloterrigena turkmenica DSM 5511_3 | 175 | | | |
| Plasmid | Haloterrigena turkmenica DSM 5511_4 | 159 | | | |
| Plasmid | Haloterrigena turkmenica DSM 5511_5 | 97 | | | |
| Plasmid | Haloterrigena turkmenica DSM 5511_6 | 21 | | | |
| Plasmid | Helicobacter acinonychis str. Sheeba_1 | 6 | | | |
| Plasmid | Helicobacter pylori G27_1 | 11 | | | |
| Plasmid | Helicobacter pylori HPAG1_1 | 8 | | | |
| Plasmid | Helicobacter pylori P12_1 | 10 | | | |
| Plasmid | Helicobacter pylori PeCan4_1 | 8 | | | |
| Plasmid | Helicobacter pylori_1 | 5 | | | |
| Plasmid | Herpetosiphon aurantiacus ATCC 23779_1 | 231 | | | |
| Plasmid | Herpetosiphon aurantiacus ATCC 23779_2 | 71 | | | |
| Plasmid | Hirschia baltica ATCC 49814_1 | 68 | | | |
| Plasmid | Ilyobacter polytropus DSM 2926_1 | 882 | | | |
| Plasmid | Ilyobacter polytropus DSM 2926_2 | 109 | | | |
| Plasmid | Isosphaera pallida ATCC 43644_1 | 32 | | | |
| Plasmid | Jannaschia sp. CCS1_1 | 71 | | | |
| Plasmid | Ketogulonicigenium vulgare Y25_1 | 249 | | | |
| Plasmid | Ketogulonicigenium vulgare Y25_2 | 220 | | | |
| Plasmid | Kineococcus radiotolerans SRS30216_1 | 17 | | | |
| Plasmid | Kineococcus radiotolerans SRS30216_2 | 184 | | | |
| Plasmid | Klebsiella pneumoniae 342_1 | 113 | | | |
| Plasmid | Klebsiella pneumoniae 342_2 | 230 | | | |
| Plasmid | Klebsiella pneumoniae NTUH-K2044_1 | 270 | | | |
| Plasmid | Klebsiella pneumoniae subsp. pneumoniae MGH 78578_1 | 178 | | | |
| Plasmid | Klebsiella pneumoniae subsp. pneumoniae MGH 78578_2 | 123 | | | |
| Plasmid | Klebsiella pneumoniae subsp. pneumoniae MGH 78578_3 | 98 | | | |
| Plasmid | Klebsiella pneumoniae subsp. pneumoniae MGH 78578_4 | 5 | | | |
| Plasmid | Klebsiella pneumoniae subsp. pneumoniae MGH 78578_5 | 5 | | | |
| Plasmid | Lactobacillus acidophilus 30SC_1 | 6 | | | |
| Plasmid | Lactobacillus acidophilus 30SC_2 | 16 | | | |
| Plasmid | Lactobacillus amylovorus GRL 1112_1 | 40 | | | |
| Plasmid | Lactobacillus amylovorus GRL 1112_2 | 27 | | | |
| Plasmid | Lactobacillus brevis ATCC 367_1 | 11 | | | |
| Plasmid | Lactobacillus brevis ATCC 367_2 | 22 | | | |
| Plasmid | Lactobacillus buchneri NRRL B-30929_1 | 46 | | | |
| Plasmid | Lactobacillus buchneri NRRL B-30929_2 | 14 | | | |
| Plasmid | Lactobacillus buchneri NRRL B-30929_3 | 18 | | | |
| Plasmid | Lactobacillus casei ATCC 334_1 | 20 | | | |
| Plasmid | Lactobacillus casei str. Zhang_1 | 44 | | | |
| Plasmid | Lactobacillus delbrueckii subsp. bulgaricus ND02_1 | 6 | | | |
| Plasmid | Lactobacillus johnsonii FI9785_1 | 2 | | | |
| Plasmid | Lactobacillus johnsonii FI9785_2 | 23 | | | |
| Plasmid | Lactobacillus plantarum subsp. plantarum ST-III_1 | 42 | | | |
| Plasmid | Lactobacillus plantarum WCFS1_1 | 3 | | | |
| Plasmid | Lactobacillus plantarum WCFS1_2 | 4 | | | |

| Type | Name | Number of genes | Baltimore class | Nucleic Acid | Monophyletic group |
|---|---|---|---|---|---|
| Plasmid | Lactobacillus plantarum WCFS1_3 | 43 | | | |
| Plasmid | Lactobacillus rhamnosus Lc 705_1 | 79 | | | |
| Plasmid | Lactobacillus salivarius UCC118_1 | 27 | | | |
| Plasmid | Lactobacillus salivarius UCC118_2 | 47 | | | |
| Plasmid | Lactobacillus salivarius UCC118_3 | 222 | | | |
| Plasmid | Lactococcus lactis subsp. cremoris SK11_1 | 10 | | | |
| Plasmid | Lactococcus lactis subsp. cremoris SK11_2 | 6 | | | |
| Plasmid | Lactococcus lactis subsp. cremoris SK11_3 | 61 | | | |
| Plasmid | Lactococcus lactis subsp. cremoris SK11_4 | 35 | | | |
| Plasmid | Lactococcus lactis subsp. cremoris SK11_5 | 8 | | | |
| Plasmid | Lactococcus lactis subsp. lactis KF147_1 | 29 | | | |
| Plasmid | Lawsonia intracellularis PHE/MN1-00_1 | 29 | | | |
| Plasmid | Lawsonia intracellularis PHE/MN1-00_2 | 24 | | | |
| Plasmid | Lawsonia intracellularis PHE/MN1-00_3 | 104 | | | |
| Plasmid | Legionella longbeachae NSW150_1 | 67 | | | |
| Plasmid | Legionella pneumophila str. Lens_1 | 56 | | | |
| Plasmid | Legionella pneumophila str. Paris_1 | 139 | | | |
| Plasmid | Leptospira biflexa serovar Patoc strain 'Patoc 1 (Ames)'_1 | 57 | | | |
| Plasmid | Leptospira biflexa serovar Patoc strain 'Patoc 1 (Paris)'_1 | 59 | | | |
| Plasmid | Leuconostoc citreum KM20_1 | 36 | | | |
| Plasmid | Leuconostoc citreum KM20_2 | 20 | | | |
| Plasmid | Leuconostoc citreum KM20_3 | 13 | | | |
| Plasmid | Leuconostoc citreum KM20_4 | 49 | | | |
| Plasmid | Leuconostoc kimchii IMSNU 11154_1 | 21 | | | |
| Plasmid | Leuconostoc kimchii IMSNU 11154_2 | 21 | | | |
| Plasmid | Leuconostoc kimchii IMSNU 11154_3 | 21 | | | |
| Plasmid | Leuconostoc kimchii IMSNU 11154_4 | 36 | | | |
| Plasmid | Leuconostoc kimchii IMSNU 11154_5 | 4 | | | |
| Plasmid | Leuconostoc mesenteroides subsp. mesenteroides ATCC 8293_1 | 35 | | | |
| Plasmid | Listeria innocua Clip11262_1 | 75 | | | |
| Plasmid | Listeria monocytogenes 08-5578_1 | 78 | | | |
| Plasmid | Lysinibacillus sphaericus C3-41_1 | 187 | | | |
| Plasmid | Macrococcus caseolyticus JCSC5402_1 | 14 | | | |
| Plasmid | Macrococcus caseolyticus JCSC5402_2 | 69 | | | |
| Plasmid | Macrococcus caseolyticus JCSC5402_3 | 1 | | | |
| Plasmid | Macrococcus caseolyticus JCSC5402_4 | 2 | | | |
| Plasmid | Macrococcus caseolyticus JCSC5402_5 | 4 | | | |
| Plasmid | Macrococcus caseolyticus JCSC5402_6 | 4 | | | |
| Plasmid | Macrococcus caseolyticus JCSC5402_7 | 5 | | | |
| Plasmid | Macrococcus caseolyticus JCSC5402_8 | 3 | | | |
| Plasmid | Marinobacter aquaeolei VT8_1 | 213 | | | |
| Plasmid | Marinobacter aquaeolei VT8_2 | 201 | | | |
| Plasmid | Marivirga tractuosa DSM 4126_1 | 8 | | | |
| Plasmid | Meiothermus silvanus DSM 9946_1 | 306 | | | |
| Plasmid | Meiothermus silvanus DSM 9946_2 | 127 | | | |
| Plasmid | Melissococcus plutonius ATCC 35311_1 | 143 | | | |
| Plasmid | Mesorhizobium ciceri biovar biserrulae WSM1271_1 | 394 | | | |
| Plasmid | Mesorhizobium loti MAFF303099_1 | 320 | | | |
| Plasmid | Mesorhizobium loti MAFF303099_2 | 209 | | | |
| Plasmid | Mesorhizobium sp. BNC1_1 | 317 | | | |
| Plasmid | Mesorhizobium sp. BNC1_2 | 113 | | | |
| Plasmid | Mesorhizobium sp. BNC1_3 | 49 | | | |
| Plasmid | Methanocaldococcus fervens AG86_1 | 35 | | | |
| Plasmid | Methanocaldococcus jannaschii DSM 2661_1 | 45 | | | |
| Plasmid | Methanocaldococcus jannaschii DSM 2661_2 | 12 | | | |
| Plasmid | Methanocaldococcus sp. FS406-22_1 | 12 | | | |
| Plasmid | Methanocaldococcus vulcanius M7_1 | 13 | | | |
| Plasmid | Methanocaldococcus vulcanius M7_2 | 2 | | | |
| Plasmid | Methanococcus maripaludis C5_1 | 9 | | | |
| Plasmid | Methanohalobium evestigatum Z-7303_1 | 106 | | | |
| Plasmid | Methanosaeta concilii GP-6_1 | 21 | | | |
| Plasmid | Methanosarcina barkeri str. Fusaro_1 | 18 | | | |
| Plasmid | Methanothermobacter marburgensis str. Marburg_1 | 4 | | | |
| Plasmid | Methylibium petroleiphilum PM1_1 | 630 | | | |
| Plasmid | Methylobacterium chloromethanicum CM4_1 | 306 | | | |
| Plasmid | Methylobacterium chloromethanicum CM4_2 | 37 | | | |
| Plasmid | Methylobacterium extorquens AM1_1 | 33 | | | |
| Plasmid | Methylobacterium extorquens AM1_2 | 34 | | | |
| Plasmid | Methylobacterium extorquens AM1_3 | 30 | | | |
| Plasmid | Methylobacterium extorquens AM1_4 | 1162 | | | |
| Plasmid | Methylobacterium extorquens DM4_1 | 105 | | | |
| Plasmid | Methylobacterium extorquens DM4_2 | 37 | | | |
| Plasmid | Methylobacterium nodulans ORS 2060_1 | 409 | | | |
| Plasmid | Methylobacterium nodulans ORS 2060_2 | 21 | | | |
| Plasmid | Methylobacterium nodulans ORS 2060_3 | 13 | | | |
| Plasmid | Methylobacterium nodulans ORS 2060_4 | 7 | | | |
| Plasmid | Methylobacterium nodulans ORS 2060_5 | 402 | | | |
| Plasmid | Methylobacterium nodulans ORS 2060_6 | 53 | | | |
| Plasmid | Methylobacterium nodulans ORS 2060_7 | 48 | | | |
| Plasmid | Methylobacterium populi BJ001_1 | 26 | | | |

| Type | Name | Number of genes | Baltimore class | Nucleic Acid | Monophyletic group |
|---|---|---|---|---|---|
| Plasmid | Methylobacterium populi BJ001_2 | 25 | | | |
| Plasmid | Methylobacterium radiotolerans JCM 2831_1 | 23 | | | |
| Plasmid | Methylobacterium radiotolerans JCM 2831_2 | 21 | | | |
| Plasmid | Methylobacterium radiotolerans JCM 2831_3 | 27 | | | |
| Plasmid | Methylobacterium radiotolerans JCM 2831_4 | 52 | | | |
| Plasmid | Methylobacterium radiotolerans JCM 2831_5 | 507 | | | |
| Plasmid | Methylobacterium radiotolerans JCM 2831_6 | 43 | | | |
| Plasmid | Methylobacterium radiotolerans JCM 2831_7 | 41 | | | |
| Plasmid | Methylobacterium radiotolerans JCM 2831_8 | 31 | | | |
| Plasmid | Methylobacterium sp. 4-46_1 | 62 | | | |
| Plasmid | Methylobacterium sp. 4-46_2 | 21 | | | |
| Plasmid | Methylovorus sp. SIP3-4_1 | 79 | | | |
| Plasmid | Methylovorus sp. SIP3-4_2 | 13 | | | |
| Plasmid | Mycobacterium abscessus ATCC 19977_1 | 21 | | | |
| Plasmid | Mycobacterium gilvum PYR-GCK_1 | 298 | | | |
| Plasmid | Mycobacterium gilvum PYR-GCK_2 | 24 | | | |
| Plasmid | Mycobacterium gilvum PYR-GCK_3 | 16 | | | |
| Plasmid | Mycobacterium marinum M_1 | 29 | | | |
| Plasmid | Mycobacterium sp. KMS_1 | 283 | | | |
| Plasmid | Mycobacterium sp. KMS_2 | 232 | | | |
| Plasmid | Mycobacterium sp. MCS_1 | 224 | | | |
| Plasmid | Mycobacterium sp. Spyr1_1 | 196 | | | |
| Plasmid | Mycobacterium sp. Spyr1_2 | 23 | | | |
| Plasmid | Mycobacterium ulcerans Agy99_1 | 81 | | | |
| Plasmid | Natranaerobius thermophilus JW/NM-WN-LF_1 | 14 | | | |
| Plasmid | Natranaerobius thermophilus JW/NM-WN-LF_2 | 10 | | | |
| Plasmid | Natrialba magadii ATCC 43099_1 | 340 | | | |
| Plasmid | Natrialba magadii ATCC 43099_2 | 219 | | | |
| Plasmid | Natrialba magadii ATCC 43099_3 | 94 | | | |
| Plasmid | Natronomonas pharaonis DSM 2160_1 | 125 | | | |
| Plasmid | Natronomonas pharaonis DSM 2160_2 | 36 | | | |
| Plasmid | Neisseria gonorrhoeae NCCP11945_1 | 12 | | | |
| Plasmid | Nitrobacter hamburgensis X14_1 | 239 | | | |
| Plasmid | Nitrobacter hamburgensis X14_2 | 172 | | | |
| Plasmid | Nitrobacter hamburgensis X14_3 | 111 | | | |
| Plasmid | Nitrosococcus halophilus Nc4_1 | 68 | | | |
| Plasmid | Nitrosococcus oceani ATCC 19707_1 | 43 | | | |
| Plasmid | Nitrosococcus watsoni C-113_1 | 41 | | | |
| Plasmid | Nitrosococcus watsoni C-113_2 | 6 | | | |
| Plasmid | Nitrosomonas eutropha C91_1 | 55 | | | |
| Plasmid | Nitrosomonas eutropha C91_2 | 52 | | | |
| Plasmid | Nitrosomonas sp. AL212_1 | 65 | | | |
| Plasmid | Nitrosomonas sp. AL212_2 | 86 | | | |
| Plasmid | Nitrosospira multiformis ATCC 25196_1 | 17 | | | |
| Plasmid | Nitrosospira multiformis ATCC 25196_2 | 16 | | | |
| Plasmid | Nitrosospira multiformis ATCC 25196_3 | 15 | | | |
| Plasmid | Nocardia farcinica IFM 10152_1 | 160 | | | |
| Plasmid | Nocardia farcinica IFM 10152_2 | 93 | | | |
| Plasmid | Nocardioides sp. JS614_1 | 264 | | | |
| Plasmid | Nocardiopsis dassonvillei subsp. dassonvillei DSM 43111_1 | 699 | | | |
| Plasmid | Nostoc punctiforme PCC 73102_1 | 25 | | | |
| Plasmid | Nostoc punctiforme PCC 73102_2 | 73 | | | |
| Plasmid | Nostoc punctiforme PCC 73102_3 | 260 | | | |
| Plasmid | Nostoc punctiforme PCC 73102_4 | 197 | | | |
| Plasmid | Nostoc punctiforme PCC 73102_5 | 48 | | | |
| Plasmid | Nostoc sp. PCC 7120_1 | 186 | | | |
| Plasmid | Nostoc sp. PCC 7120_2 | 5 | | | |
| Plasmid | Nostoc sp. PCC 7120_3 | 90 | | | |
| Plasmid | Nostoc sp. PCC 7120_4 | 31 | | | |
| Plasmid | Nostoc sp. PCC 7120_5 | 66 | | | |
| Plasmid | Nostoc sp. PCC 7120_6 | 386 | | | |
| Plasmid | Novosphingobium aromaticivorans DSM 12444_1 | 182 | | | |
| Plasmid | Novosphingobium aromaticivorans DSM 12444_2 | 431 | | | |
| Plasmid | Novosphingobium sp. PP1Y_1 | 199 | | | |
| Plasmid | Novosphingobium sp. PP1Y_2 | 54 | | | |
| Plasmid | Novosphingobium sp. PP1Y_3 | 969 | | | |
| Plasmid | Oceanithermus profundus DSM 14977_1 | 144 | | | |
| Plasmid | Ochrobactrum anthropi ATCC 49188_1 | 161 | | | |
| Plasmid | Ochrobactrum anthropi ATCC 49188_2 | 91 | | | |
| Plasmid | Ochrobactrum anthropi ATCC 49188_3 | 86 | | | |
| Plasmid | Ochrobactrum anthropi ATCC 49188_4 | 37 | | | |
| Plasmid | Paenibacillus polymyxa SC2_1 | 626 | | | |
| Plasmid | Pantoea sp. At-9b_1 | 768 | | | |
| Plasmid | Pantoea sp. At-9b_2 | 340 | | | |
| Plasmid | Pantoea sp. At-9b_3 | 292 | | | |
| Plasmid | Pantoea sp. At-9b_4 | 294 | | | |
| Plasmid | Pantoea sp. At-9b_5 | 117 | | | |
| Plasmid | Pantoea vagans C9-1_1 | 535 | | | |
| Plasmid | Pantoea vagans C9-1_2 | 162 | | | |
| Plasmid | Pantoea vagans C9-1_3 | 229 | | | |

| Type | Name | Number of genes | Baltimore class | Nucleic Acid | Monophyletic group |
|---|---|---|---|---|---|
| Plasmid | Paracoccus denitrificans PD1222_1 | 616 | | | |
| Plasmid | Pelobacter propionicus DSM 2379_1 | 195 | | | |
| Plasmid | Pelobacter propionicus DSM 2379_2 | 33 | | | |
| Plasmid | Persephonella marina EX-H1_1 | 70 | | | |
| Plasmid | Phenylobacterium zucineum HLK1_1 | 325 | | | |
| Plasmid | Photobacterium profundum SS9_1 | 67 | | | |
| Plasmid | Photorhabdus asymbiotica_1 | 27 | | | |
| Plasmid | Planctomyces limnophilus DSM 3776_1 | 60 | | | |
| Plasmid | Polaromonas naphthalenivorans CJ2_1 | 305 | | | |
| Plasmid | Polaromonas naphthalenivorans CJ2_2 | 161 | | | |
| Plasmid | Polaromonas naphthalenivorans CJ2_3 | 155 | | | |
| Plasmid | Polaromonas naphthalenivorans CJ2_4 | 136 | | | |
| Plasmid | Polaromonas naphthalenivorans CJ2_5 | 52 | | | |
| Plasmid | Polaromonas naphthalenivorans CJ2_6 | 20 | | | |
| Plasmid | Polaromonas naphthalenivorans CJ2_7 | 11 | | | |
| Plasmid | Polaromonas naphthalenivorans CJ2_8 | 5 | | | |
| Plasmid | Polaromonas sp. JS666_1 | 326 | | | |
| Plasmid | Polaromonas sp. JS666_2 | 310 | | | |
| Plasmid | Polymorphum gilvum SL003B-26A1_1 | 69 | | | |
| Plasmid | Prosthecochloris aestuarii DSM 271_1 | 64 | | | |
| Plasmid | Proteus mirabilis_1 | 55 | | | |
| Plasmid | Pseudomonas fluorescens SBW25_1 | 474 | | | |
| Plasmid | Pseudomonas syringae pv. phaseolicola 1448A_1 | 127 | | | |
| Plasmid | Pseudomonas syringae pv. phaseolicola 1448A_2 | 60 | | | |
| Plasmid | Pseudomonas syringae pv. tomato str. DC3000_1 | 70 | | | |
| Plasmid | Pseudomonas syringae pv. tomato str. DC3000_2 | 68 | | | |
| Plasmid | Pseudonocardia dioxanivorans CB1190_1 | 14 | | | |
| Plasmid | Pseudonocardia dioxanivorans CB1190_2 | 172 | | | |
| Plasmid | Psychrobacter cryohalolentis K5_1 | 44 | | | |
| Plasmid | Psychrobacter sp. PRwf-1_1 | 14 | | | |
| Plasmid | Psychrobacter sp. PRwf-1_2 | 1 | | | |
| Plasmid | Pusillimonas sp. T7-7_1 | 77 | | | |
| Plasmid | Pyrococcus abyssi GE5_1 | 2 | | | |
| Plasmid | Rahnella sp. Y9602_1 | 595 | | | |
| Plasmid | Rahnella sp. Y9602_2 | 124 | | | |
| Plasmid | Ralstonia eutropha H16_1 | 420 | | | |
| Plasmid | Ralstonia eutropha JMP134_1 | 512 | | | |
| Plasmid | Ralstonia eutropha JMP134_2 | 88 | | | |
| Plasmid | Ralstonia pickettii 12D_1 | 276 | | | |
| Plasmid | Ralstonia pickettii 12D_2 | 67 | | | |
| Plasmid | Ralstonia pickettii 12D_3 | 427 | | | |
| Plasmid | Ralstonia pickettii 12J_1 | 90 | | | |
| Plasmid | Ralstonia solanacearum GMI1000_1 | 1676 | | | |
| Plasmid | Ralstonia solanacearum PSI07_1 | 1694 | | | |
| Plasmid | Rhizobium etli CFN 42_1 | 336 | | | |
| Plasmid | Rhizobium etli CFN 42_2 | 175 | | | |
| Plasmid | Rhizobium etli CFN 42_3 | 163 | | | |
| Plasmid | Rhizobium etli CFN 42_4 | 232 | | | |
| Plasmid | Rhizobium etli CFN 42_5 | 455 | | | |
| Plasmid | Rhizobium etli CFN 42_6 | 567 | | | |
| Plasmid | Rhizobium etli CIAT 652_1 | 356 | | | |
| Plasmid | Rhizobium etli CIAT 652_2 | 976 | | | |
| Plasmid | Rhizobium etli CIAT 652_3 | 381 | | | |
| Plasmid | Rhizobium leguminosarum bv. trifolii WSM1325_1 | 737 | | | |
| Plasmid | Rhizobium leguminosarum bv. trifolii WSM1325_2 | 306 | | | |
| Plasmid | Rhizobium leguminosarum bv. trifolii WSM1325_3 | 494 | | | |
| Plasmid | Rhizobium leguminosarum bv. trifolii WSM1325_4 | 275 | | | |
| Plasmid | Rhizobium leguminosarum bv. trifolii WSM1325_5 | 624 | | | |
| Plasmid | Rhizobium leguminosarum bv. trifolii WSM2304_1 | 450 | | | |
| Plasmid | Rhizobium leguminosarum bv. trifolii WSM2304_2 | 1161 | | | |
| Plasmid | Rhizobium leguminosarum bv. trifolii WSM2304_3 | 256 | | | |
| Plasmid | Rhizobium leguminosarum bv. trifolii WSM2304_4 | 223 | | | |
| Plasmid | Rhizobium leguminosarum bv. viciae 3841_1 | 780 | | | |
| Plasmid | Rhizobium leguminosarum bv. viciae 3841_2 | 305 | | | |
| Plasmid | Rhizobium leguminosarum bv. viciae 3841_3 | 442 | | | |
| Plasmid | Rhizobium leguminosarum bv. viciae 3841_4 | 156 | | | |
| Plasmid | Rhizobium leguminosarum bv. viciae 3841_5 | 132 | | | |
| Plasmid | Rhizobium leguminosarum bv. viciae 3841_6 | 634 | | | |
| Plasmid | Rhodobacter capsulatus SB 1003_1 | 149 | | | |
| Plasmid | Rhodobacter sphaeroides 2.4.1_1 | 100 | | | |
| Plasmid | Rhodobacter sphaeroides 2.4.1_2 | 82 | | | |
| Plasmid | Rhodobacter sphaeroides 2.4.1_3 | 87 | | | |
| Plasmid | Rhodobacter sphaeroides 2.4.1_4 | 87 | | | |
| Plasmid | Rhodobacter sphaeroides 2.4.1_5 | 29 | | | |
| Plasmid | Rhodobacter sphaeroides ATCC 17025_1 | 802 | | | |
| Plasmid | Rhodobacter sphaeroides ATCC 17025_2 | 268 | | | |
| Plasmid | Rhodobacter sphaeroides ATCC 17025_3 | 109 | | | |
| Plasmid | Rhodobacter sphaeroides ATCC 17025_4 | 31 | | | |
| Plasmid | Rhodobacter sphaeroides ATCC 17025_5 | 12 | | | |
| Plasmid | Rhodobacter sphaeroides ATCC 17029_1 | 107 | | | |

| Type | Name | Number of genes | Baltimore class | Nucleic Acid | Monophyletic group |
|---|---|---|---|---|---|
| Plasmid | Rhodobacter sphaeroides KD131_1 | 102 | | | |
| Plasmid | Rhodobacter sphaeroides KD131_2 | 142 | | | |
| Plasmid | Rhodococcus erythropolis PR4_1 | 102 | | | |
| Plasmid | Rhodococcus erythropolis PR4_2 | 3 | | | |
| Plasmid | Rhodococcus erythropolis PR4_3 | 298 | | | |
| Plasmid | Rhodococcus jostii RHA1_1 | 1146 | | | |
| Plasmid | Rhodococcus jostii RHA1_2 | 454 | | | |
| Plasmid | Rhodococcus jostii RHA1_3 | 334 | | | |
| Plasmid | Rhodococcus opacus B4_1 | 6 | | | |
| Plasmid | Rhodococcus opacus B4_2 | 2 | | | |
| Plasmid | Rhodococcus opacus B4_3 | 593 | | | |
| Plasmid | Rhodococcus opacus B4_4 | 248 | | | |
| Plasmid | Rhodococcus opacus B4_5 | 102 | | | |
| Plasmid | Rhodoferax ferrireducens T118_1 | 248 | | | |
| Plasmid | Rhodopseudomonas palustris CGA009_1 | 7 | | | |
| Plasmid | Rhodospirillum rubrum ATCC 11170_1 | 50 | | | |
| Plasmid | Rhodothermus marinus DSM 4252_1 | 97 | | | |
| Plasmid | Rickettsia africae ESF-5_1 | 11 | | | |
| Plasmid | Rickettsia felis URRWXCal2_1 | 68 | | | |
| Plasmid | Rickettsia felis URRWXCal2_2 | 44 | | | |
| Plasmid | Rickettsia massiliae MTU5_1 | 12 | | | |
| Plasmid | Rickettsia peacockii str. Rustic_1 | 20 | | | |
| Plasmid | Roseobacter denitrificans OCh 114_1 | 105 | | | |
| Plasmid | Roseobacter denitrificans OCh 114_2 | 56 | | | |
| Plasmid | Roseobacter denitrificans OCh 114_3 | 16 | | | |
| Plasmid | Roseobacter denitrificans OCh 114_4 | 6 | | | |
| Plasmid | Ruegeria pomeroyi DSS-3_1 | 442 | | | |
| Plasmid | Ruegeria sp. TM1040_1 | 106 | | | |
| Plasmid | Ruegeria sp. TM1040_2 | 728 | | | |
| Plasmid | Ruminococcus albus 7_1 | 376 | | | |
| Plasmid | Ruminococcus albus 7_2 | 290 | | | |
| Plasmid | Ruminococcus albus 7_3 | 18 | | | |
| Plasmid | Ruminococcus albus 7_4 | 8 | | | |
| Plasmid | Salinibacter ruber DSM 13855_1 | 32 | | | |
| Plasmid | Salinibacter ruber M8_1 | 13 | | | |
| Plasmid | Salinibacter ruber M8_2 | 38 | | | |
| Plasmid | Salinibacter ruber M8_3 | 50 | | | |
| Plasmid | Salmonella enterica subsp. enterica serovar Agona str. SL483_1 | 52 | | | |
| Plasmid | Salmonella enterica subsp. enterica serovar Choleraesuis str. SC-B67_1 | 51 | | | |
| Plasmid | Salmonella enterica subsp. enterica serovar Choleraesuis str. SC-B67_2 | 170 | | | |
| Plasmid | Salmonella enterica subsp. enterica serovar Dublin str. CT_02021853_1 | 103 | | | |
| Plasmid | Salmonella enterica subsp. enterica serovar Heidelberg str. SL476_1 | 125 | | | |
| Plasmid | Salmonella enterica subsp. enterica serovar Heidelberg str. SL476_2 | 4 | | | |
| Plasmid | Salmonella enterica subsp. enterica serovar Newport str. SL254_1 | 190 | | | |
| Plasmid | Salmonella enterica subsp. enterica serovar Newport str. SL254_2 | 3 | | | |
| Plasmid | Salmonella enterica subsp. enterica serovar Paratyphi C strain RKS4594_1 | 62 | | | |
| Plasmid | Salmonella enterica subsp. enterica serovar Schwarzengrund str. CVM19633_1 | 122 | | | |
| Plasmid | Salmonella enterica subsp. enterica serovar Schwarzengrund str. CVM19633_2 | 5 | | | |
| Plasmid | Salmonella enterica subsp. enterica serovar Typhi str. CT18_1 | 235 | | | |
| Plasmid | Salmonella enterica subsp. enterica serovar Typhi str. CT18_2 | 128 | | | |
| Plasmid | Salmonella typhimurium LT2_1 | 102 | | | |
| Plasmid | Sebaldella termitidis ATCC 33386_1 | 51 | | | |
| Plasmid | Sebaldella termitidis ATCC 33386_2 | 16 | | | |
| Plasmid | Serratia proteamaculans 568_1 | 51 | | | |
| Plasmid | Shewanella baltica OS155_1 | 92 | | | |
| Plasmid | Shewanella baltica OS155_2 | 71 | | | |
| Plasmid | Shewanella baltica OS155_3 | 12 | | | |
| Plasmid | Shewanella baltica OS155_4 | 7 | | | |
| Plasmid | Shewanella baltica OS185_1 | 71 | | | |
| Plasmid | Shewanella baltica OS195_1 | 67 | | | |
| Plasmid | Shewanella baltica OS195_2 | 73 | | | |
| Plasmid | Shewanella baltica OS195_3 | 49 | | | |
| Plasmid | Shewanella baltica OS223_1 | 79 | | | |
| Plasmid | Shewanella baltica OS223_2 | 51 | | | |
| Plasmid | Shewanella baltica OS223_3 | 61 | | | |
| Plasmid | Shewanella oneidensis MR-1_1 | 149 | | | |
| Plasmid | Shewanella sp. ANA-3_1 | 249 | | | |
| Plasmid | Shewanella sp. MR-7_1 | 8 | | | |
| Plasmid | Shigella boydii CDC 3083-94_1 | 3 | | | |
| Plasmid | Shigella boydii CDC 3083-94_2 | 52 | | | |
| Plasmid | Shigella boydii CDC 3083-94_3 | 4 | | | |
| Plasmid | Shigella boydii CDC 3083-94_4 | 242 | | | |
| Plasmid | Shigella boydii CDC 3083-94_5 | 10 | | | |
| Plasmid | Shigella boydii Sb227_1 | 148 | | | |
| Plasmid | Shigella dysenteriae Sd197_1 | 223 | | | |
| Plasmid | Shigella dysenteriae Sd197_2 | 8 | | | |
| Plasmid | Shigella flexneri 2a str. 301_1 | 263 | | | |
| Plasmid | Shigella sonnei Ss046_1 | 238 | | | |
| Plasmid | Shigella sonnei Ss046_2 | 5 | | | |
| Plasmid | Shigella sonnei Ss046_3 | 8 | | | |

| Type | Name | Number of genes | Baltimore class | Nucleic Acid | Monophyletic group |
|---|---|---|---|---|---|
| Plasmid | Shigella sonnei Ss046_4 | 1 | | | |
| Plasmid | Sinorhizobium fredii NGR234_1 | 405 | | | |
| Plasmid | Sinorhizobium fredii NGR234_2 | 2328 | | | |
| Plasmid | Sinorhizobium medicae WSM419_1 | 1441 | | | |
| Plasmid | Sinorhizobium medicae WSM419_2 | 1094 | | | |
| Plasmid | Sinorhizobium medicae WSM419_3 | 149 | | | |
| Plasmid | Sinorhizobium meliloti 1021_1 | 1290 | | | |
| Plasmid | Sinorhizobium meliloti 1021_2 | 1569 | | | |
| Plasmid | Sodalis glossinidius str. 'morsitans'_1 | 54 | | | |
| Plasmid | Sodalis glossinidius str. 'morsitans'_2 | 23 | | | |
| Plasmid | Sodalis glossinidius str. 'morsitans'_3 | 7 | | | |
| Plasmid | Sphingobium japonicum UT26S_1 | 44 | | | |
| Plasmid | Sphingobium japonicum UT26S_2 | 224 | | | |
| Plasmid | Sphingobium japonicum UT26S_3 | 8 | | | |
| Plasmid | Sphingomonas wittichii RW1_1 | 285 | | | |
| Plasmid | Sphingomonas wittichii RW1_2 | 210 | | | |
| Plasmid | Sphingopyxis alaskensis RB2256_1 | 30 | | | |
| Plasmid | Spirosoma linguale DSM 74_1 | 177 | | | |
| Plasmid | Spirosoma linguale DSM 74_2 | 142 | | | |
| Plasmid | Spirosoma linguale DSM 74_3 | 40 | | | |
| Plasmid | Spirosoma linguale DSM 74_4 | 14 | | | |
| Plasmid | Spirosoma linguale DSM 74_5 | 12 | | | |
| Plasmid | Spirosoma linguale DSM 74_6 | 10 | | | |
| Plasmid | Spirosoma linguale DSM 74_7 | 11 | | | |
| Plasmid | Spirosoma linguale DSM 74_8 | 8 | | | |
| Plasmid | Staphylococcus aureus subsp. aureus COL_1 | 3 | | | |
| Plasmid | Staphylococcus aureus subsp. aureus ED98_1 | 2 | | | |
| Plasmid | Staphylococcus aureus subsp. aureus ED98_2 | 3 | | | |
| Plasmid | Staphylococcus aureus subsp. aureus ED98_3 | 23 | | | |
| Plasmid | Staphylococcus aureus subsp. aureus JH1_1 | 33 | | | |
| Plasmid | Staphylococcus aureus subsp. aureus JH9_1 | 29 | | | |
| Plasmid | Staphylococcus aureus subsp. aureus MSSA476_1 | 19 | | | |
| Plasmid | Staphylococcus aureus subsp. aureus Mu50_1 | 34 | | | |
| Plasmid | Staphylococcus aureus subsp. aureus N315_1 | 31 | | | |
| Plasmid | Staphylococcus aureus subsp. aureus USA300_FPR3757_1 | 5 | | | |
| Plasmid | Staphylococcus aureus subsp. aureus USA300_FPR3757_2 | 3 | | | |
| Plasmid | Staphylococcus aureus subsp. aureus USA300_FPR3757_3 | 36 | | | |
| Plasmid | Staphylococcus aureus subsp. aureus USA300_TCH1516_1 | 26 | | | |
| Plasmid | Staphylococcus aureus subsp. aureus USA300_TCH1516_2 | 6 | | | |
| Plasmid | Staphylococcus epidermidis ATCC 12228_1 | 11 | | | |
| Plasmid | Staphylococcus epidermidis ATCC 12228_2 | 22 | | | |
| Plasmid | Staphylococcus epidermidis ATCC 12228_3 | 16 | | | |
| Plasmid | Staphylococcus epidermidis ATCC 12228_4 | 8 | | | |
| Plasmid | Staphylococcus epidermidis ATCC 12228_5 | 6 | | | |
| Plasmid | Staphylococcus epidermidis ATCC 12228_6 | 3 | | | |
| Plasmid | Staphylococcus epidermidis RP62A_1 | 32 | | | |
| Plasmid | Staphylococcus haemolyticus JCSC1435_1 | 3 | | | |
| Plasmid | Staphylococcus haemolyticus JCSC1435_2 | 2 | | | |
| Plasmid | Staphylococcus haemolyticus JCSC1435_3 | 11 | | | |
| Plasmid | Staphylococcus saprophyticus subsp. saprophyticus ATCC 15305_1 | 45 | | | |
| Plasmid | Staphylococcus saprophyticus subsp. saprophyticus ATCC 15305_2 | 23 | | | |
| Plasmid | Streptobacillus moniliformis DSM 12112_1 | 8 | | | |
| Plasmid | Streptococcus gallolyticus subsp. gallolyticus ATCC BAA-2069_1 | 20 | | | |
| Plasmid | Streptococcus suis BM407_1 | 15 | | | |
| Plasmid | Streptococcus thermophilus LMD-9_1 | 4 | | | |
| Plasmid | Streptococcus thermophilus LMD-9_2 | 2 | | | |
| Plasmid | Streptomyces avermitilis MA-4680_1 | 96 | | | |
| Plasmid | Streptomyces coelicolor A3(2)_1 | 351 | | | |
| Plasmid | Streptomyces coelicolor A3(2)_2 | 34 | | | |
| Plasmid | Streptosporangium roseum DSM 43021_1 | 30 | | | |
| Plasmid | Sulfolobus islandicus L.D.8.5_1 | 32 | | | |
| Plasmid | Sulfolobus islandicus Y.N.15.51_1 | 50 | | | |
| Plasmid | Sulfuricurvum kujiense DSM 16994_1 | 112 | | | |
| Plasmid | Sulfuricurvum kujiense DSM 16994_2 | 86 | | | |
| Plasmid | Sulfuricurvum kujiense DSM 16994_3 | 58 | | | |
| Plasmid | Sulfuricurvum kujiense DSM 16994_4 | 4 | | | |
| Plasmid | Synechococcus elongatus PCC 7942_1 | 50 | | | |
| Plasmid | Synechococcus sp. PCC 7002_1 | 165 | | | |
| Plasmid | Synechococcus sp. PCC 7002_2 | 3 | | | |
| Plasmid | Synechococcus sp. PCC 7002_3 | 17 | | | |
| Plasmid | Synechococcus sp. PCC 7002_4 | 30 | | | |
| Plasmid | Synechococcus sp. PCC 7002_5 | 39 | | | |
| Plasmid | Synechococcus sp. PCC 7002_6 | 109 | | | |
| Plasmid | Synechocystis sp. PCC 6803_1 | 132 | | | |
| Plasmid | Synechocystis sp. PCC 6803_2 | 105 | | | |
| Plasmid | Synechocystis sp. PCC 6803_3 | 49 | | | |
| Plasmid | Synechocystis sp. PCC 6803_4 | 110 | | | |
| Plasmid | Thauera sp. MZ1T_1 | 75 | | | |
| Plasmid | Thermococcus barophilus MP_1 | 58 | | | |
| Plasmid | Thermofilum pendens Hrk 5_1 | 52 | | | |

| Type | Name | Number of genes | Baltimore class | Nucleic Acid | Monophyletic group |
|---|---|---|---|---|---|
| Plasmid | Thermomicrobium roseum DSM 5159_1 | 937 | | | |
| Plasmid | Thermovibrio ammonificans HB-1_1 | 102 | | | |
| Plasmid | Thermus scotoductus SA-01_1 | 12 | | | |
| Plasmid | Thermus thermophilus HB27_1 | 228 | | | |
| Plasmid | Thermus thermophilus HB8_1 | 251 | | | |
| Plasmid | Thermus thermophilus HB8_2 | 14 | | | |
| Plasmid | Thioalkalivibrio sp. K90mix_1 | 280 | | | |
| Plasmid | Thiomonas intermedia K12_1 | 43 | | | |
| Plasmid | Thiomonas intermedia K12_2 | 23 | | | |
| Plasmid | Treponema succinifaciens DSM 2489_1 | 156 | | | |
| Plasmid | Tsukamurella paurometabola DSM 20162_1 | 85 | | | |
| Plasmid | uncultured Termite group 1 bacterium phylotype Rs-D17_1 | 9 | | | |
| Plasmid | uncultured Termite group 1 bacterium phylotype Rs-D17_2 | 3 | | | |
| Plasmid | uncultured Termite group 1 bacterium phylotype Rs-D17_3 | 3 | | | |
| Plasmid | Verminephrobacter eiseniae EF01-2_1 | 39 | | | |
| Plasmid | Verrucosispora maris AB-18-032_1 | 53 | | | |
| Plasmid | Vibrio fischeri ES114_1 | 57 | | | |
| Plasmid | Vibrio fischeri MJ11_1 | 195 | | | |
| Plasmid | Vibrio harveyi ATCC BAA-1116_1 | 120 | | | |
| Plasmid | Vibrio vulnificus YJ016_1 | 69 | | | |
| Plasmid | Waddlia chondrophila WSU 86-1044_1 | 22 | | | |
| Plasmid | Wigglesworthia glossinidia endosymbiont of Glossina brevipalpis_1 | 6 | | | |
| Plasmid | Xanthobacter autotrophicus Py2_1 | 289 | | | |
| Plasmid | Xanthomonas axonopodis pv. citri str. 306_1 | 42 | | | |
| Plasmid | Xanthomonas axonopodis pv. citri str. 306_2 | 73 | | | |
| Plasmid | Xanthomonas campestris pv. vesicatoria str. 85-10_1 | 2 | | | |
| Plasmid | Xanthomonas campestris pv. vesicatoria str. 85-10_2 | 22 | | | |
| Plasmid | Xanthomonas campestris pv. vesicatoria str. 85-10_3 | 43 | | | |
| Plasmid | Xanthomonas campestris pv. vesicatoria str. 85-10_4 | 172 | | | |
| Plasmid | Xenorhabdus nematophila ATCC 19061_1 | 175 | | | |
| Plasmid | Xylanimonas cellulosilytica DSM 15894_1 | 106 | | | |
| Plasmid | Xylella fastidiosa 9a5c_1 | 2 | | | |
| Plasmid | Xylella fastidiosa 9a5c_2 | 64 | | | |
| Plasmid | Xylella fastidiosa M23_1 | 40 | | | |
| Plasmid | Xylella fastidiosa Temecula1_1 | 2 | | | |
| Plasmid | Yersinia enterocolitica subsp. enterocolitica 8081_1 | 72 | | | |
| Plasmid | Yersinia enterocolitica subsp. palearctica 105.5R(r)_1 | 86 | | | |
| Plasmid | Yersinia pestis Angola_1 | 88 | | | |
| Plasmid | Yersinia pestis Angola_2 | 120 | | | |
| Plasmid | Yersinia pestis Antiqua_1 | 99 | | | |
| Plasmid | Yersinia pestis Antiqua_2 | 9 | | | |
| Plasmid | Yersinia pestis Antiqua_3 | 89 | | | |
| Plasmid | Yersinia pestis biovar Microtus str. 91001_1 | 85 | | | |
| Plasmid | Yersinia pestis biovar Microtus str. 91001_2 | 30 | | | |
| Plasmid | Yersinia pestis biovar Microtus str. 91001_3 | 122 | | | |
| Plasmid | Yersinia pestis biovar Microtus str. 91001_4 | 10 | | | |
| Plasmid | Yersinia pestis CO92_1 | 71 | | | |
| Plasmid | Yersinia pestis CO92_2 | 9 | | | |
| Plasmid | Yersinia pestis CO92_3 | 101 | | | |
| Plasmid | Yersinia pestis KIM_1 | 116 | | | |
| Plasmid | Yersinia pestis Nepal516_1 | 104 | | | |
| Plasmid | Yersinia pestis Nepal516_2 | 9 | | | |
| Plasmid | Yersinia pestis Pestoides F_1 | 88 | | | |
| Plasmid | Yersinia pestis Pestoides F_2 | 131 | | | |
| Plasmid | Yersinia pestis Z176003_1 | 67 | | | |
| Plasmid | Yersinia pestis Z176003_2 | 76 | | | |
| Plasmid | Yersinia pestis Z176003_3 | 8 | | | |
| Plasmid | Yersinia pseudotuberculosis IP 31758_1 | 64 | | | |
| Plasmid | Yersinia pseudotuberculosis IP 31758_2 | 136 | | | |
| Plasmid | Yersinia pseudotuberculosis IP 32953_1 | 95 | | | |
| Plasmid | Yersinia pseudotuberculosis IP 32953_2 | 42 | | | |
| Plasmid | Yersinia pseudotuberculosis PB1/+_1 | 87 | | | |
| Plasmid | Zymomonas mobilis subsp. mobilis NCIB 11163_1 | 46 | | | |
| Plasmid | Zymomonas mobilis subsp. mobilis NCIB 11163_2 | 32 | | | |
| Plasmid | Zymomonas mobilis subsp. mobilis NCIB 11163_3 | 6 | | | |
| Virus | Abaca bunchy top virus | 5 | 2 | DNA | NA |
| Virus | Abalone herpesvirus Taiwan/2004 | 14 | 1 | DNA | NA |
| Virus | Abalone herpesvirus Victoria/AUS/2009 | 118 | 1 | DNA | NA |
| Virus | Abalone shriveling syndrome-associated virus | 31 | 1 | DNA | NA |
| Virus | Abelson murine leukemia virus | 6 | 6 | RNA | 2 |
| Virus | Abutilon Brazil virus | 7 | 2 | DNA | 3 |
| Virus | Abutilon mosaic Bolivia virus | 7 | 2 | DNA | 3 |
| Virus | Abutilon mosaic Brazil virus | 7 | 2 | DNA | 3 |
| Virus | Abutilon mosaic virus | 9 | 2 | DNA | 3 |
| Virus | Acanthamoeba polyphaga mimivirus | 979 | 1 | DNA | 5 |
| Virus | Acanthocystis turfacea Chlorella virus 1 | 860 | 1 | DNA | 5 |
| Virus | Acheta domestica densovirus | 5 | 2 | DNA | 3 |
| Virus | Acholeplasma phage L2 | 16 | 1 | DNA | NA |
| Virus | Acholeplasma phage MV-L1 | 4 | 2 | DNA | NA |
| Virus | Acidianus bottle-shaped virus | 57 | 1 | DNA | NA |

| Type | Name | Number of genes | Baltimore class | Nucleic Acid | Monophyletic group |
|---|---|---|---|---|---|
| Virus | Acidianus filamentous virus 1 | 40 | 1 | DNA | NA |
| Virus | Acidianus filamentous virus 2 | 52 | 1 | DNA | NA |
| Virus | Acidianus filamentous virus 3 | 68 | 1 | DNA | NA |
| Virus | Acidianus filamentous virus 6 | 66 | 1 | DNA | NA |
| Virus | Acidianus filamentous virus 7 | 57 | 1 | DNA | NA |
| Virus | Acidianus filamentous virus 8 | 61 | 1 | DNA | NA |
| Virus | Acidianus filamentous virus 9 | 73 | 1 | DNA | NA |
| Virus | Acidianus rod-shaped virus 1 | 41 | 1 | DNA | NA |
| Virus | Acidianus spindle-shaped virus 1 | 38 | 1 | DNA | NA |
| Virus | Acidianus two-tailed virus | 72 | 1 | DNA | NA |
| Virus | Acinetobacter bacteriophage AP22 | 89 | 1 | DNA | 1 |
| Virus | Acinetobacter phage 133 | 257 | 1 | DNA | 4 |
| Virus | Acinetobacter phage Ac42 | 255 | 1 | DNA | 1 |
| Virus | Acinetobacter phage Acj61 | 241 | 1 | DNA | 1 |
| Virus | Acinetobacter phage Acj9 | 253 | 1 | DNA | 1 |
| Virus | Acinetobacter phage AP205 | 4 | 4 | RNA | 1 |
| Virus | Acinetobacter phage ZZ1 | 402 | 1 | DNA | 1 |
| Virus | Aconitum latent virus | 6 | 4 | RNA | 1 |
| Virus | Actinidia virus B | 5 | 4 | RNA | 1 |
| Virus | Actinomyces phage Av-1 | 22 | 1 | DNA | 4 |
| Virus | Actinoplanes phage phiAsp2 | 76 | 1 | DNA | 4 |
| Virus | Acute bee paralysis virus | 3 | 4 | RNA | 1 |
| Virus | Acyrthosiphon pisum bacteriophage APSE-1 | 54 | 1 | DNA | 4 |
| Virus | Acyrthosiphon pisum virus | 2 | 4 | RNA | NA |
| Virus | Adeno-associated virus - 1 | 2 | 2 | DNA | 3 |
| Virus | Adeno-associated virus - 2 | 8 | 2 | DNA | 3 |
| Virus | Adeno-associated virus - 3 | 2 | 2 | DNA | 3 |
| Virus | Adeno-associated virus - 4 | 2 | 2 | DNA | 3 |
| Virus | Adeno-associated virus - 5 | 2 | 2 | DNA | 3 |
| Virus | Adeno-associated virus - 7 | 2 | 2 | DNA | 3 |
| Virus | Adeno-associated virus - 8 | 2 | 2 | DNA | 3 |
| Virus | Adoxophyes honmai NPV | 125 | 1 | DNA | NA |
| Virus | Adoxophyes orana granulovirus | 119 | 1 | DNA | NA |
| Virus | Adoxophyes orana nucleopolyhedrovirus | 121 | 1 | DNA | NA |
| Virus | Adult diarrheal rotavirus strain J19 | 11 | 3 | RNA | NA |
| Virus | Aedes aegypti densovirus | 3 | 2 | DNA | 3 |
| Virus | Aedes albopictus densovirus | 3 | 2 | DNA | 3 |
| Virus | Aedes flavivirus | 14 | 4 | RNA | 1 |
| Virus | Aedes pseudoscutellaris reovirus | 9 | 3 | RNA | NA |
| Virus | Aeromonas phage 25 | 242 | 1 | DNA | 4 |
| Virus | Aeromonas phage 31 | 247 | 1 | DNA | 4 |
| Virus | Aeromonas phage 44RR2.8t | 252 | 1 | DNA | 4 |
| Virus | Aeromonas phage 65 | 437 | 1 | DNA | 4 |
| Virus | Aeromonas phage Aeh1 | 352 | 1 | DNA | 4 |
| Virus | Aeromonas phage phiAS4 | 271 | 1 | DNA | 4 |
| Virus | Aeromonas phage phiAS5 | 343 | 1 | DNA | 4 |
| Virus | Aeromonas phage phiO18P | 45 | 1 | DNA | 4 |
| Virus | African cassava mosaic virus | 9 | 2 | DNA | 3 |
| Virus | African green monkey polyomavirus | 5 | 1 | DNA | 3 |
| Virus | African green monkey simian foamy virus | 5 | 6 | RNA | 2 |
| Virus | African horsesickness virus | 11 | 3 | RNA | NA |
| Virus | African oil palm ringspot virus | 5 | 4 | RNA | 1 |
| Virus | African swine fever virus | 160 | 1 | DNA | 5 |
| Virus | Ageratum enation virus | 6 | 2 | DNA | 3 |
| Virus | Ageratum leaf Cameroon betasatellite | 1 | 2 | DNA | 3 |
| Virus | Ageratum leaf curl Buea betasatellite | 1 | 2 | DNA | 3 |
| Virus | Ageratum leaf curl Cameroon alphasatellite | 3 | 2 | DNA | 3 |
| Virus | Ageratum leaf curl Cameroon virus | 6 | 2 | DNA | 3 |
| Virus | Ageratum leaf curl disease associated satellite DNA beta | 1 | 2 | DNA | 3 |
| Virus | Ageratum yellow vein China betasatellite | 1 | 2 | DNA | 3 |
| Virus | Ageratum yellow vein China virus | 6 | 2 | DNA | 3 |
| Virus | Ageratum yellow vein Hualian virus | 6 | 2 | DNA | 3 |
| Virus | Ageratum yellow vein Singapore alphasatellite | 1 | 2 | DNA | 3 |
| Virus | Ageratum yellow vein Sri Lanka virus | 6 | 2 | DNA | 3 |
| Virus | Ageratum yellow vein Taiwan virus | 6 | 2 | DNA | 3 |
| Virus | Ageratum yellow vein virus | 7 | 2 | DNA | 3 |
| Virus | Ageratum yellow vein virus satellite DNA beta | 1 | 2 | DNA | 3 |
| Virus | Aggregatibacter phage S1249 | 66 | 1 | DNA | 4 |
| Virus | Agropyron mosaic virus | 12 | 4 | RNA | 1 |
| Virus | Agrotis ipsilon multiple nucleopolyhedrovirus | 163 | 1 | DNA | NA |
| Virus | Agrotis segetum granulovirus | 132 | 1 | DNA | NA |
| Virus | Agrotis segetum nucleopolyhedrovirus | 153 | 1 | DNA | NA |
| Virus | Aguacate virus | 4 | 5 | RNA | NA |
| Virus | Aichi virus | 11 | 4 | RNA | 1 |
| Virus | Aino virus | 4 | 5 | RNA | NA |
| Virus | Akabane virus | 4 | 5 | RNA | NA |
| Virus | Alcelaphine herpesvirus 1 | 71 | 1 | DNA | NA |
| Virus | Aleutian mink disease virus | 4 | 2 | DNA | 3 |
| Virus | Alfalfa mosaic virus | 4 | 4 | RNA | 1 |
| Virus | Algerian watermelon mosaic virus | 12 | 4 | RNA | 1 |

| Type | Name | Number of genes | Baltimore class | Nucleic Acid | Monophyletic group |
|---|---|---|---|---|---|
| Virus | Alkhumra hemorrhagic fever virus | 14 | 4 | RNA | 1 |
| Virus | Allamanda leaf curl virus | 6 | 2 | DNA | 3 |
| Virus | Allium virus X | 5 | 4 | RNA | 1 |
| Virus | Allpahuayo virus | 4 | 5 | RNA | NA |
| Virus | Alphapapillomavirus 12 | 8 | 1 | DNA | 3 |
| Virus | Alphapapillomavirus 13 | 7 | 1 | DNA | 3 |
| Virus | Alphapapillomavirus 3 | 7 | 1 | DNA | 3 |
| Virus | Alstroemeria virus x | 5 | 4 | RNA | 1 |
| Virus | Alternanthera mosaic virus | 5 | 4 | RNA | 1 |
| Virus | Alternanthera yellow vein betasatellite | 1 | 2 | DNA | 3 |
| Virus | Alternanthera yellow vein virus | 6 | 2 | DNA | 3 |
| Virus | Alternaria alternata virus 1 | 4 | 3 | RNA | NA |
| Virus | Amapari virus | 4 | 5 | RNA | NA |
| Virus | Amasya cherry disease associated chrysovirus | 4 | 3 | RNA | NA |
| Virus | Amasya cherry disease-associated mycovirus | 2 | 3 | RNA | 1 |
| Virus | Amazon lily mild mottle virus | 4 | 4 | RNA | NA |
| Virus | Ambystoma tigrinum virus | 95 | 1 | DNA | 5 |
| Virus | American grass carp reovirus | 12 | 3 | RNA | NA |
| Virus | American hop latent virus | 6 | 4 | RNA | 1 |
| Virus | American plum line pattern virus | 4 | 4 | RNA | 1 |
| Virus | Amsacta moorei entomopoxvirus 'L' | 294 | 1 | DNA | 5 |
| Virus | Anagyris vein yellowing virus | 3 | 4 | RNA | 1 |
| Virus | Anatid herpesvirus 1 | 77 | 1 | DNA | NA |
| Virus | Andes virus | 4 | 5 | RNA | NA |
| Virus | Angelonia flower break virus | 6 | 4 | RNA | 1 |
| Virus | Anguillid herpesvirus 1 | 134 | 1 | DNA | NA |
| Virus | Anopheles gambiae densonucleosis virus | 3 | 2 | DNA | 3 |
| Virus | Antheraea pernyi nucleopolyhedrovirus | 147 | 1 | DNA | NA |
| Virus | Anticarsia gemmatalis nucleopolyhedrovirus | 152 | 1 | DNA | NA |
| Virus | Aotine herpesvirus 1 | 153 | 1 | DNA | NA |
| Virus | Aphid lethal paralysis virus | 2 | 4 | RNA | 1 |
| Virus | Apium virus Y | 12 | 4 | RNA | 1 |
| Virus | Apocheima cinerarium nucleopolyhedrovirus | 117 | 1 | DNA | NA |
| Virus | Apoi virus | 14 | 4 | RNA | 1 |
| Virus | Apple chlorotic leaf spot virus | 3 | 4 | RNA | 1 |
| Virus | Apple latent spherical virus | 11 | 4 | RNA | 1 |
| Virus | Apple mosaic virus | 4 | 4 | RNA | 1 |
| Virus | Apple stem grooving virus | 2 | 4 | RNA | 1 |
| Virus | Apple stem pitting virus | 5 | 4 | RNA | 1 |
| Virus | Apricot latent virus | 5 | 4 | RNA | 1 |
| Virus | Apricot pseudo-chlorotic leaf spot virus | 3 | 4 | RNA | 1 |
| Virus | Aquareovirus A | 9 | 3 | RNA | NA |
| Virus | Aquareovirus C | 12 | 3 | RNA | NA |
| Virus | Arabis mosaic virus | 10 | 4 | RNA | 1 |
| Virus | Arabis mosaic virus large satellite RNA | 1 | NA | RNA | NA |
| Virus | Archaeal BJ1 virus | 70 | 1 | DNA | 4 |
| Virus | Armigeres subalbatus virus SaX06-AK20 | 2 | 3 | RNA | NA |
| Virus | Aroa virus | 1 | 4 | RNA | 1 |
| Virus | Arracacha mottle virus | 1 | 4 | RNA | 1 |
| Virus | Artemisia virus A | 4 | 4 | RNA | 1 |
| Virus | Artibeus jamaicensis parvovirus 1 | 2 | 2 | DNA | NA |
| Virus | Artichoke mottled crinkle virus | 5 | 4 | RNA | 1 |
| Virus | Asclepias asymptomatic virus | 3 | 4 | RNA | 1 |
| Virus | Asparagus virus 2 | 5 | 4 | RNA | 1 |
| Virus | Asparagus virus 3 | 5 | 4 | RNA | 1 |
| Virus | Astrovirus MLB1 | 3 | 4 | RNA | 1 |
| Virus | Astrovirus MLB1 HK05 | 3 | 4 | RNA | 1 |
| Virus | Astrovirus MLB2 | 3 | 4 | RNA | 1 |
| Virus | Astrovirus MLB3 | 3 | 4 | RNA | 1 |
| Virus | Astrovirus VA1 | 3 | 4 | RNA | 1 |
| Virus | Astrovirus VA2 | 3 | 4 | RNA | 1 |
| Virus | Astrovirus VA3 | 3 | 4 | RNA | 1 |
| Virus | Astrovirus VA4 | 3 | 4 | RNA | 1 |
| Virus | Astrovirus wild boar/WBAstV-1/2011/HUN | 3 | 4 | RNA | 1 |
| Virus | Asystasia begomovirus 1 | 8 | 2 | DNA | 3 |
| Virus | Ateline herpesvirus 3 | 73 | 1 | DNA | NA |
| Virus | Atkinsonella hypoxylon partitivirus | 2 | 3 | RNA | 1 |
| Virus | Atlantic salmon swim bladder sarcoma virus | 3 | 6 | RNA | 2 |
| Virus | Aura virus | 14 | 4 | RNA | 1 |
| Virus | Australian bat lyssavirus | 5 | 5 | RNA | NA |
| Virus | Autographa californica nucleopolyhedrovirus | 156 | 1 | DNA | NA |
| Virus | Avian adeno-associated virus ATCC VR-865 | 2 | 2 | DNA | 3 |
| Virus | Avian adeno-associated virus strain DA-1 | 2 | 2 | DNA | 3 |
| Virus | Avian carcinoma virus | 1 | 6 | RNA | 2 |
| Virus | Avian encephalomyelitis virus | 12 | 4 | RNA | 1 |
| Virus | Avian endogenous retrovirus EAV-HP | 1 | 6 | RNA | 2 |
| Virus | Avian gyrovirus 2 | 3 | 2 | DNA | 3 |
| Virus | Avian leukemia virus | 3 | 6 | RNA | 2 |
| Virus | Avian leukosis virus | 3 | 6 | RNA | 2 |
| Virus | Avian metapneumovirus | 9 | 5 | RNA | NA |

| Type | Name | Number of genes | Baltimore class | Nucleic Acid | Monophyletic group |
|---|---|---|---|---|---|
| Virus | Avian myelocytomatosis virus | 5 | 6 | RNA | 2 |
| Virus | Avian orthoreovirus | 10 | 3 | RNA | NA |
| Virus | Avian paramyxovirus 6 | 7 | 5 | RNA | NA |
| Virus | Avian sapelovirus | 13 | 4 | RNA | 1 |
| Virus | Azospirillum phage Cd | 95 | 1 | DNA | 4 |
| Virus | Baboon orthoreovirus | 11 | 3 | RNA | NA |
| Virus | Bacillus phage 0305phi8-36 | 246 | 1 | DNA | 4 |
| Virus | Bacillus phage AP50 | 31 | 1 | DNA | NA |
| Virus | Bacillus phage B103 | 17 | 1 | DNA | 4 |
| Virus | Bacillus phage B4 | 277 | 1 | DNA | NA |
| Virus | Bacillus phage Bam35c | 32 | 1 | DNA | NA |
| Virus | Bacillus phage Bastille | 267 | 1 | DNA | NA |
| Virus | Bacillus phage BCJA1c | 58 | 1 | DNA | 4 |
| Virus | Bacillus phage BCP78 | 227 | 1 | DNA | NA |
| Virus | Bacillus phage BPS13 | 268 | 1 | DNA | NA |
| Virus | Bacillus phage BtCS33 | 57 | 1 | DNA | NA |
| Virus | Bacillus phage Cherry | 51 | 1 | DNA | 4 |
| Virus | Bacillus phage Fah | 50 | 1 | DNA | 4 |
| Virus | Bacillus phage GA-1 | 35 | 1 | DNA | 4 |
| Virus | Bacillus phage Gamma | 53 | 1 | DNA | 4 |
| Virus | Bacillus phage IEBH | 86 | 1 | DNA | 4 |
| Virus | Bacillus phage PBC1 | 50 | 1 | DNA | NA |
| Virus | Bacillus phage phBC6A51 | 75 | 1 | DNA | NA |
| Virus | Bacillus phage phBC6A52 | 49 | 1 | DNA | NA |
| Virus | Bacillus phage phi105 | 51 | 1 | DNA | 4 |
| Virus | Bacillus phage phi29 | 27 | 1 | DNA | 4 |
| Virus | Bacillus phage SPBc2 | 185 | 1 | DNA | NA |
| Virus | Bacillus phage SPO1 | 206 | 1 | DNA | 4 |
| Virus | Bacillus phage SPP1 | 101 | 1 | DNA | 4 |
| Virus | Bacillus phage TP21-L | 56 | 1 | DNA | 4 |
| Virus | Bacillus phage W.Ph. | 258 | 1 | DNA | NA |
| Virus | Bacillus phage WBeta | 53 | 1 | DNA | 4 |
| Virus | Bacillus thuringiensis phage GIL16c | 31 | 1 | DNA | NA |
| Virus | Bacillus virus 1 | 54 | 1 | DNA | NA |
| Virus | Bacteriophage APSE-2 | 41 | 1 | DNA | 4 |
| Virus | Bacteroides phage B124-14 | 68 | 1 | DNA | 4 |
| Virus | Bacteroides phage B40-8 | 46 | 1 | DNA | 4 |
| Virus | Bagaza virus | 1 | 4 | RNA | 1 |
| Virus | Bamboo mosaic virus | 6 | 4 | RNA | 1 |
| Virus | Bamboo mosaic virus satellite RNA | 1 | NA | RNA | NA |
| Virus | Banana bract mosaic virus | 12 | 4 | RNA | 1 |
| Virus | Banana bunchy top virus | 5 | 2 | DNA | NA |
| Virus | Banana mild mosaic virus | 5 | 4 | RNA | 1 |
| Virus | Banana streak CA virus | 3 | 7 | DNA | 2 |
| Virus | Banana streak GF virus | 3 | 7 | DNA | 2 |
| Virus | Banana streak IM virus | 3 | 7 | DNA | 2 |
| Virus | Banana streak Mysore virus | 3 | 7 | DNA | 2 |
| Virus | Banana streak OL virus | 3 | 7 | DNA | 2 |
| Virus | Banana streak UA virus | 3 | 7 | DNA | 2 |
| Virus | Banana streak UI virus | 3 | 7 | DNA | 2 |
| Virus | Banana streak UL virus | 3 | 7 | DNA | 2 |
| Virus | Banana streak UM virus | 3 | 7 | DNA | 2 |
| Virus | Banana streak virus | 3 | 7 | DNA | 2 |
| Virus | Banana streak virus strain Acuminata Vietnam | 3 | 7 | DNA | 2 |
| Virus | Bandicoot papillomatosis carcinomatosis virus type 1 | 3 | 1 | DNA | 3 |
| Virus | Bandicoot papillomatosis carcinomatosis virus type 2 | 3 | 1 | DNA | 3 |
| Virus | Banna virus | 12 | 3 | RNA | NA |
| Virus | Barbel circovirus | 2 | 2 | DNA | 3 |
| Virus | Barfin flounder nervous necrosis virus | 3 | 4 | RNA | 1 |
| Virus | Barfin flounder virus BF93Hok | 3 | 4 | RNA | 1 |
| Virus | Barley dwarf virus | 4 | 2 | DNA | 3 |
| Virus | Barley mild mosaic virus | 13 | 4 | RNA | 1 |
| Virus | Barley stripe mosaic virus | 7 | 4 | RNA | 1 |
| Virus | Barley yellow dwarf virus - MAV | 6 | 4 | RNA | 1 |
| Virus | Barley yellow dwarf virus-GAV | 6 | 4 | RNA | 1 |
| Virus | Barley yellow dwarf virus-PAS | 6 | 4 | RNA | 1 |
| Virus | Barley yellow dwarf virus-PAV | 7 | 4 | RNA | 1 |
| Virus | Barley yellow mosaic virus | 13 | 4 | RNA | 1 |
| Virus | Barmah Forest virus | 13 | 4 | RNA | 1 |
| Virus | Basella rugose mosaic virus | 12 | 4 | RNA | 1 |
| Virus | Bat adeno-associated virus YNM | 2 | 2 | DNA | 3 |
| Virus | Bat adenovirus 2 | 30 | 1 | DNA | NA |
| Virus | Bat adenovirus TJM | 30 | 1 | DNA | NA |
| Virus | Bat coronavirus (BtCoV/133/2005) | 8 | 4 | RNA | NA |
| Virus | Bat coronavirus 1A | 7 | 4 | RNA | NA |
| Virus | Bat coronavirus 1B | 7 | 4 | RNA | NA |
| Virus | Bat coronavirus BM48-31/BGR/2008 | 9 | 4 | RNA | NA |
| Virus | Bat coronavirus HKU2 | 8 | 4 | RNA | NA |
| Virus | Bat coronavirus HKU4 | 9 | 4 | RNA | NA |
| Virus | Bat coronavirus HKU5 | 9 | 4 | RNA | NA |

| Type | Name | Number of genes | Baltimore class | Nucleic Acid | Monophyletic group |
|---|---|---|---|---|---|
| Virus | Bat coronavirus HKU8 | 8 | 4 | RNA | NA |
| Virus | Bat coronavirus HKU9 | 8 | 4 | RNA | NA |
| Virus | Bat hepevirus | 3 | 4 | RNA | 1 |
| Virus | Bat picornavirus 1 | 15 | 4 | RNA | 1 |
| Virus | Bat picornavirus 2 | 15 | 4 | RNA | 1 |
| Virus | Bat picornavirus 3 | 13 | 4 | RNA | 1 |
| Virus | Bat sapovirus TLC58/HK | 2 | 4 | RNA | 1 |
| Virus | Bathycoccus sp. RCC1105 virus BpV1 | 203 | 1 | DNA | 5 |
| Virus | Bdellovibrio phage phi1402 | 42 | 1 | DNA | 4 |
| Virus | Bdellovibrio phage phiMH2K | 11 | 2 | DNA | NA |
| Virus | Beak and feather disease virus | 3 | 2 | DNA | 3 |
| Virus | Bean calico mosaic virus | 7 | 2 | DNA | 3 |
| Virus | Bean common mosaic necrosis virus | 12 | 4 | RNA | 1 |
| Virus | Bean common mosaic virus | 12 | 4 | RNA | 1 |
| Virus | Bean dwarf mosaic virus | 6 | 2 | DNA | 3 |
| Virus | Bean golden mosaic virus | 6 | 2 | DNA | 3 |
| Virus | Bean golden yellow mosaic virus | 8 | 2 | DNA | 3 |
| Virus | Bean leafroll virus | 5 | 4 | RNA | 1 |
| Virus | Bean necrotic mosaic virus | 5 | 5 | RNA | NA |
| Virus | Bean pod mottle virus | 11 | 4 | RNA | 1 |
| Virus | Bean yellow disorder virus | 15 | 4 | RNA | 1 |
| Virus | Bean yellow dwarf virus | 4 | 2 | DNA | 3 |
| Virus | Bean yellow mosaic Mexico virus | 4 | 2 | DNA | 3 |
| Virus | Bean yellow mosaic virus | 12 | 4 | RNA | 1 |
| Virus | Bear Canyon virus | 4 | 5 | RNA | NA |
| Virus | Bebaru virus | 2 | 4 | RNA | 1 |
| Virus | Beet black scorch virus | 6 | 4 | RNA | 1 |
| Virus | Beet chlorosis virus | 5 | 4 | RNA | 1 |
| Virus | Beet cryptic virus 1 | 2 | 3 | RNA | 1 |
| Virus | Beet curly top Iran virus | 5 | 2 | DNA | 3 |
| Virus | Beet curly top virus | 6 | 2 | DNA | 3 |
| Virus | Beet mild curly top virus | 7 | 2 | DNA | 3 |
| Virus | Beet mild yellowing virus | 6 | 4 | RNA | 1 |
| Virus | Beet mosaic virus | 12 | 4 | RNA | 1 |
| Virus | Beet necrotic yellow vein virus | 12 | 4 | RNA | 1 |
| Virus | Beet pseudo-yellows virus | 12 | 4 | RNA | 1 |
| Virus | Beet ringspot virus | 9 | 4 | RNA | 1 |
| Virus | Beet severe curly top virus | 8 | 2 | DNA | 3 |
| Virus | Beet soil-borne mosaic virus | 10 | 4 | RNA | 1 |
| Virus | Beet soil-borne virus | 7 | 4 | RNA | 1 |
| Virus | Beet virus Q | 9 | 4 | RNA | 1 |
| Virus | Beet western yellows ST9 associated virus | 4 | 4 | RNA | NA |
| Virus | Beet western yellows virus | 6 | 4 | RNA | 1 |
| Virus | Beet yellows virus | 10 | 4 | RNA | 1 |
| Virus | Beilong virus | 11 | 5 | RNA | NA |
| Virus | Bell pepper endornavirus | 1 | 3 | RNA | NA |
| Virus | Bell pepper mottle virus | 5 | 4 | RNA | 1 |
| Virus | Beluga Whale coronavirus SW1 | 14 | 4 | RNA | NA |
| Virus | Bettongia penicillata papillomavirus 1 | 7 | 1 | DNA | 3 |
| Virus | Bhendi yellow vein Bhubhaneswar virus | 7 | 2 | DNA | 3 |
| Virus | Bhendi yellow vein mosaic virus | 7 | 2 | DNA | 3 |
| Virus | Bhendi yellow vein mosaic virus satellite DNA beta | 1 | NA | NA | NA |
| Virus | Bhendi yellow vein mosaic virus-associated alphasatellite | 1 | NA | NA | NA |
| Virus | Bidens mottle virus | 12 | 4 | RNA | 1 |
| Virus | Bitter gourd leaf curl betasatellite | 1 | 2 | DNA | NA |
| Virus | BK polyomavirus | 6 | 1 | DNA | 3 |
| Virus | Black beetle virus | 5 | 4 | RNA | 1 |
| Virus | Black queen cell virus | 2 | 4 | RNA | 1 |
| Virus | Black raspberry necrosis virus | 10 | 4 | RNA | 1 |
| Virus | Black raspberry virus F | 2 | 3 | RNA | NA |
| Virus | Blackberry chlorotic ringspot virus | 5 | 4 | RNA | 1 |
| Virus | Blackberry virus E | 5 | 4 | RNA | 1 |
| Virus | Blackberry virus Y | 12 | 4 | RNA | 1 |
| Virus | Blackberry yellow vein-associated virus | 13 | 4 | RNA | 1 |
| Virus | Blackcurrant reversion virus | 9 | 4 | RNA | 1 |
| Virus | Blackcurrant reversion virus satellite RNA | 1 | NA | RNA | NA |
| Virus | Blainvillea yellow spot virus | 7 | 2 | DNA | 3 |
| Virus | Blattella germanica densovirus | 5 | 2 | DNA | 3 |
| Virus | Blechum interveinal chlorosis virus | 7 | 2 | DNA | 3 |
| Virus | Blotched snakehead virus | 11 | 3 | RNA | NA |
| Virus | Blue squill virus A | 11 | 4 | RNA | 1 |
| Virus | Blueberry latent virus | 2 | 3 | RNA | NA |
| Virus | Blueberry necrotic ring blotch virus | 7 | 4 | RNA | NA |
| Virus | Blueberry red ringspot virus | 8 | 7 | DNA | 2 |
| Virus | Blueberry scorch virus | 6 | 4 | RNA | 1 |
| Virus | Blueberry virus A | 11 | 4 | RNA | 1 |
| Virus | Bluegill picornavirus | 12 | 4 | RNA | 1 |
| Virus | Bluetongue virus | 11 | 3 | RNA | NA |
| Virus | Bocavirus gorilla/GBoV1/2009 | 4 | 2 | DNA | 3 |
| Virus | Bombyx mandarina nucleopolyhedrovirus | 141 | 1 | DNA | NA |

| Type | Name | Number of genes | Baltimore class | Nucleic Acid | Monophyletic group |
|---|---|---|---|---|---|
| Virus | Bombyx mori densovirus 5 | 6 | 2 | DNA | 3 |
| Virus | Bombyx mori Macula-like virus | 3 | 4 | RNA | NA |
| Virus | Bombyx mori NPV | 143 | 1 | DNA | NA |
| Virus | Boolarra virus | 3 | 4 | RNA | 1 |
| Virus | Border disease virus | 14 | 4 | RNA | 1 |
| Virus | Bordetella phage BIP-1 | 48 | 1 | DNA | 4 |
| Virus | Bordetella phage BMP-1 | 47 | 1 | DNA | 4 |
| Virus | Bordetella phage BPP-1 | 49 | 1 | DNA | 4 |
| Virus | Borna disease virus | 6 | 5 | RNA | NA |
| Virus | Botryotinia fuckeliana partitivirus 1 | 3 | 3 | RNA | 1 |
| Virus | Botryotinia fuckeliana totivirus 1 | 2 | 3 | RNA | NA |
| Virus | Botrytis cinerea debilitation-related virus | 1 | 4 | RNA | 1 |
| Virus | Botrytis porri RNA virus 1 | 2 | 3 | RNA | NA |
| Virus | Botrytis virus F | 2 | 4 | RNA | 1 |
| Virus | Botrytis virus X | 5 | 4 | RNA | 1 |
| Virus | Bougainvillea spectabilis chlorotic vein-banding virus | 4 | 7 | DNA | 2 |
| Virus | Bovine adeno-associated virus | 2 | 2 | DNA | 3 |
| Virus | Bovine adenovirus A | 29 | 1 | DNA | NA |
| Virus | Bovine adenovirus B | 52 | 1 | DNA | NA |
| Virus | Bovine adenovirus D | 36 | 1 | DNA | NA |
| Virus | Bovine coronavirus | 27 | 4 | RNA | NA |
| Virus | Bovine enterovirus | 12 | 4 | RNA | 1 |
| Virus | Bovine ephemeral fever virus | 12 | 5 | RNA | NA |
| Virus | Bovine foamy virus | 5 | 6 | RNA | 2 |
| Virus | Bovine herpesvirus 1 | 70 | 1 | DNA | NA |
| Virus | Bovine herpesvirus 4 | 79 | 1 | DNA | NA |
| Virus | Bovine herpesvirus 5 | 70 | 1 | DNA | NA |
| Virus | Bovine hungarovirus | 13 | 4 | RNA | 1 |
| Virus | Bovine immunodeficiency virus | 5 | 6 | RNA | 2 |
| Virus | Bovine kobuvirus | 12 | 4 | RNA | 1 |
| Virus | Bovine leukemia virus | 14 | 6 | RNA | 2 |
| Virus | Bovine papillomavirus - 1 | 8 | 1 | DNA | 3 |
| Virus | Bovine papillomavirus - 5 | 6 | 1 | DNA | 3 |
| Virus | Bovine papillomavirus 7 | 7 | 1 | DNA | 3 |
| Virus | Bovine papillomavirus 8 | 8 | 1 | DNA | 3 |
| Virus | Bovine papular stomatitis virus | 131 | 1 | DNA | 5 |
| Virus | Bovine parainfluenza virus 3 | 6 | 5 | RNA | NA |
| Virus | Bovine parvovirus | 4 | 2 | DNA | 3 |
| Virus | Bovine parvovirus - 2 | 2 | 2 | DNA | 3 |
| Virus | Bovine polyomavirus | 6 | 1 | DNA | 3 |
| Virus | Bovine respiratory coronavirus AH187 | 12 | 4 | RNA | NA |
| Virus | Bovine respiratory coronavirus bovine/US/OH-440-TC/1996 | 11 | 4 | RNA | NA |
| Virus | Bovine respiratory syncytial virus | 11 | 5 | RNA | NA |
| Virus | Bovine rhinitis B virus | 13 | 4 | RNA | 1 |
| Virus | Bovine viral diarrhea virus 1 | 14 | 4 | RNA | 1 |
| Virus | Bovine viral diarrhea virus 2 | 14 | 4 | RNA | 1 |
| Virus | Bovine viral diarrhea virus 3 Th/04_KhonKaen | 1 | 4 | RNA | 1 |
| Virus | Brassica yellows virus | 6 | 4 | RNA | 1 |
| Virus | Breda virus | 6 | 4 | RNA | NA |
| Virus | Brevicoryne brassicae picorna-like virus | 1 | 4 | RNA | 1 |
| Virus | Broad bean mottle virus | 4 | 4 | RNA | 1 |
| Virus | Broad bean necrosis virus | 8 | 4 | RNA | 1 |
| Virus | Broad bean wilt virus 1 | 10 | 4 | RNA | 1 |
| Virus | Broad bean wilt virus 2 | 10 | 4 | RNA | 1 |
| Virus | Brochothrix phage A9 | 199 | 1 | DNA | 4 |
| Virus | Brochothrix phage BL3 | 67 | 1 | DNA | 4 |
| Virus | Brochothrix phage NF5 | 58 | 1 | DNA | 4 |
| Virus | Brome mosaic virus | 4 | 4 | RNA | 1 |
| Virus | Brome streak mosaic virus | 12 | 4 | RNA | 1 |
| Virus | Bromus catharticus striate mosaic virus | 4 | 2 | DNA | 3 |
| Virus | Broome virus | 11 | 3 | RNA | NA |
| Virus | Brugmansia mild mottle virus | 4 | 4 | RNA | 1 |
| Virus | Brugmansia suaveolens mottle virus | 12 | 4 | RNA | 1 |
| Virus | Budgerigar fledgling disease virus - 1 | 9 | 1 | DNA | NA |
| Virus | Bundibugyo ebolavirus | 9 | 5 | RNA | NA |
| Virus | Bunyamwera virus | 4 | 5 | RNA | NA |
| Virus | Burkholderia phage AH2 | 78 | 1 | DNA | 4 |
| Virus | Burkholderia phage Bcep1 | 71 | 1 | DNA | 4 |
| Virus | Burkholderia phage Bcep176 | 81 | 1 | DNA | 4 |
| Virus | Burkholderia phage Bcep22 | 81 | 1 | DNA | 4 |
| Virus | Burkholderia phage Bcep43 | 65 | 1 | DNA | 4 |
| Virus | Burkholderia phage Bcep781 | 66 | 1 | DNA | 4 |
| Virus | Burkholderia phage BcepB1A | 73 | 1 | DNA | 4 |
| Virus | Burkholderia phage BcepC6B | 46 | 1 | DNA | 4 |
| Virus | Burkholderia phage BcepF1 | 127 | 1 | DNA | 4 |
| Virus | Burkholderia phage BcepGomr | 75 | 1 | DNA | 4 |
| Virus | Burkholderia phage BcepIL02 | 76 | 1 | DNA | 4 |
| Virus | Burkholderia phage BcepMu | 53 | 1 | DNA | 4 |
| Virus | Burkholderia phage BcepNazgul | 73 | 1 | DNA | 4 |
| Virus | Burkholderia phage BcepNY3 | 70 | 1 | DNA | 4 |

| Type | Name | Number of genes | Baltimore class | Nucleic Acid | Monophyletic group |
|---|---|---|---|---|---|
| Virus | Burkholderia phage DC1 | 73 | 1 | DNA | 4 |
| Virus | Burkholderia phage KL1 | 55 | 1 | DNA | 4 |
| Virus | Burkholderia phage KL3 | 52 | 1 | DNA | 4 |
| Virus | Burkholderia phage KS10 | 49 | 1 | DNA | 4 |
| Virus | Burkholderia phage KS14 | 44 | 1 | DNA | 4 |
| Virus | Burkholderia phage KS5 | 46 | 1 | DNA | 4 |
| Virus | Burkholderia phage KS9 | 50 | 1 | DNA | 4 |
| Virus | Burkholderia phage phi1026b | 83 | 1 | DNA | 4 |
| Virus | Burkholderia phage phi52237 | 47 | 1 | DNA | 4 |
| Virus | Burkholderia phage phi644-2 | 71 | 1 | DNA | 4 |
| Virus | Burkholderia phage phiE12-2 | 50 | 1 | DNA | 4 |
| Virus | Burkholderia phage phiE125 | 71 | 1 | DNA | 4 |
| Virus | Burkholderia phage phiE202 | 48 | 1 | DNA | 4 |
| Virus | Burkholderia phage phiE255 | 55 | 1 | DNA | 4 |
| Virus | Butterbur mosaic virus | 6 | 4 | RNA | 1 |
| Virus | Cabbage leaf curl virus | 7 | 2 | DNA | 3 |
| Virus | Cacao swollen shoot virus | 5 | 7 | DNA | 2 |
| Virus | Cactus mild mottle virus | 4 | 4 | RNA | 1 |
| Virus | Cactus virus X | 7 | 4 | RNA | 1 |
| Virus | Cafeteria roenbergensis virus BV-PW1 | 552 | 1 | DNA | 5 |
| Virus | Caladenia virus A | 11 | 4 | RNA | 1 |
| Virus | Calicivirus isolate TCG | 2 | 4 | RNA | 1 |
| Virus | Calicivirus pig/AB90/CAN | 2 | 4 | RNA | 1 |
| Virus | Calicivirus strain NB | 2 | 4 | RNA | 1 |
| Virus | California sea lion anellovirus | 3 | 2 | DNA | NA |
| Virus | California sea lion polyomavirus 1 | 6 | 1 | DNA | 3 |
| Virus | Callitrichine herpesvirus 3 | 72 | 1 | DNA | NA |
| Virus | Camelpox virus | 211 | 1 | DNA | 5 |
| Virus | Camelus dromedarius papillomavirus type 1 | 7 | 1 | DNA | 3 |
| Virus | Camelus dromedarius papillomavirus type 2 | 7 | 1 | DNA | 3 |
| Virus | Campoletis sonorensis ichnovirus | 5 | 1 | DNA | NA |
| Virus | Campylobacter phage CP30A | 162 | NA | NA | NA |
| Virus | Campylobacter phage CPX | 149 | 1 | DNA | 4 |
| Virus | Campylobacter phage NCTC12673 | 166 | 1 | DNA | 4 |
| Virus | Canary circovirus | 2 | 2 | DNA | 3 |
| Virus | Canary polyomavirus | 5 | 1 | DNA | 3 |
| Virus | Canarypox virus | 328 | 1 | DNA | 5 |
| Virus | Candiru virus | 4 | 5 | RNA | NA |
| Virus | Canine adenovirus 1 | 31 | 1 | DNA | NA |
| Virus | Canine adenovirus 2 | 31 | 1 | DNA | NA |
| Virus | Canine adenovirus A | 30 | 1 | DNA | NA |
| Virus | Canine calicivirus | 12 | 4 | RNA | 1 |
| Virus | Canine distemper virus | 7 | 5 | RNA | NA |
| Virus | Canine minute virus | 4 | 2 | DNA | 3 |
| Virus | Canine papillomavirus 10 | 7 | 1 | DNA | 3 |
| Virus | Canine papillomavirus 11 | 8 | 1 | DNA | 3 |
| Virus | Canine papillomavirus 3 | 6 | 1 | DNA | 3 |
| Virus | Canine papillomavirus 4 | 6 | 1 | DNA | 3 |
| Virus | Canine papillomavirus 5 | 7 | 1 | DNA | 3 |
| Virus | Canine papillomavirus 8 | 7 | 1 | DNA | 3 |
| Virus | Canine papillomavirus 9 | 7 | 1 | DNA | 3 |
| Virus | Canine parvovirus | 5 | 2 | DNA | 3 |
| Virus | Canine picornavirus | 13 | 4 | RNA | 1 |
| Virus | Canis familiaris papillomavirus 2 | 8 | 1 | DNA | 3 |
| Virus | Canna Yellow Streak Virus | 12 | 4 | RNA | 1 |
| Virus | Capra hircus papillomavirus 1 | 7 | 1 | DNA | 3 |
| Virus | Capreolus capreolus papillomavirus 1 | 8 | 1 | DNA | 3 |
| Virus | Caprine arthritis-encephalitis virus | 6 | 6 | RNA | 2 |
| Virus | Capsicum chlorosis virus | 5 | 5 | RNA | NA |
| Virus | Cardamine chlorotic fleck virus | 4 | 4 | RNA | 1 |
| Virus | Cardiospermum yellow leaf curl betasatellite | 1 | 2 | DNA | 3 |
| Virus | Caretta caretta papillomavirus 1 | 7 | 1 | DNA | 3 |
| Virus | Carnation etched ring virus | 6 | 7 | DNA | 2 |
| Virus | Carnation Italian ringspot virus | 5 | 4 | RNA | 1 |
| Virus | Carnation mottle virus | 6 | 4 | RNA | 1 |
| Virus | Carnation ringspot virus | 4 | 4 | RNA | 1 |
| Virus | Carrot mottle mimic virus | 4 | 4 | RNA | 1 |
| Virus | Carrot mottle virus | 3 | 4 | RNA | 1 |
| Virus | Carrot red leaf luteovirus associated RNA | 3 | 4 | RNA | NA |
| Virus | Carrot red leaf virus | 6 | 4 | RNA | 1 |
| Virus | Carrot yellow leaf virus | 10 | 4 | RNA | 1 |
| Virus | CAS virus | 4 | 5 | RNA | NA |
| Virus | Casphalia extranea densovirus | 3 | 2 | DNA | 3 |
| Virus | Cassava brown streak virus | 12 | 4 | RNA | 1 |
| Virus | Cassava common mosaic virus | 5 | 4 | RNA | 1 |
| Virus | Cassava mosaic Madagascar alphasatellite | 1 | NA | NA | NA |
| Virus | Cassava mosaic Madagascar virus | 8 | 2 | DNA | 3 |
| Virus | Cassava vein mosaic virus | 5 | 7 | DNA | 2 |
| Virus | Cassava virus C | 3 | 4 | RNA | 1 |
| Virus | Cassia yellow blotch virus | 4 | 4 | RNA | 1 |

| Type | Name | Number of genes | Baltimore class | Nucleic Acid | Monophyletic group |
|---|---|---|---|---|---|
| Virus | Cauliflower mosaic virus | 7 | 7 | DNA | 2 |
| Virus | Caulobacter phage CcrColossus | 452 | 1 | DNA | 4 |
| Virus | Caulobacter phage CcrKarma | 357 | 1 | DNA | 4 |
| Virus | Caulobacter phage CcrMagneto | 351 | 1 | DNA | 4 |
| Virus | Caulobacter phage CcrRogue | 354 | 1 | DNA | 4 |
| Virus | Caulobacter phage CcrSwift | 347 | 1 | DNA | 4 |
| Virus | Caulobacter phage phiCbK | 342 | 1 | DNA | 4 |
| Virus | Cavally virus | 7 | 4 | RNA | 1 |
| Virus | Caviid herpesvirus 2 | 106 | 1 | DNA | NA |
| Virus | Celeribacter phage P12053L | 56 | 1 | DNA | NA |
| Virus | Celery mosaic virus | 12 | 4 | RNA | 1 |
| Virus | Cell fusing agent virus | 14 | 4 | RNA | 1 |
| Virus | Centrosema yellow spot virus | 5 | 2 | DNA | 3 |
| Virus | Ceratocystis polonica partitivirus | 2 | 3 | RNA | 1 |
| Virus | Ceratocystis resinifera partitivirus | 2 | 3 | RNA | 1 |
| Virus | Cercopithecine herpesvirus 2 | 75 | 1 | DNA | NA |
| Virus | Cercopithecine herpesvirus 5 | 183 | 1 | DNA | NA |
| Virus | Cercopithecine herpesvirus 9 | 74 | 1 | DNA | NA |
| Virus | Cereal yellow dwarf virus - RPV | 6 | 4 | RNA | 1 |
| Virus | Cereal yellow dwarf virus-RPS | 6 | 4 | RNA | 1 |
| Virus | Cestrum yellow leaf curling virus | 7 | 7 | DNA | 2 |
| Virus | Chaetoceros lorenzianus DNA Virus | 3 | NA | DNA | NA |
| Virus | Chaetoceros salsugineum DNA virus | 6 | NA | DNA | NA |
| Virus | Chaetoceros socialis f. radians RNA virus 01 | 2 | 4 | RNA | 1 |
| Virus | Chaetoceros tenuissimus DNA virus | 3 | NA | DNA | NA |
| Virus | Chalara elegans RNA Virus 1 | 2 | 3 | RNA | NA |
| Virus | Chaoyang virus | 1 | 4 | RNA | 1 |
| Virus | Chapare virus | 4 | 5 | RNA | NA |
| Virus | Chayote mosaic virus | 3 | 4 | RNA | 1 |
| Virus | Chayote yellow mosaic virus | 5 | 2 | DNA | 3 |
| Virus | Chelonia mydas papillomavirus 1 | 7 | 1 | DNA | 3 |
| Virus | Cherry green ring mottle virus | 3 | 4 | RNA | 1 |
| Virus | Cherry leaf roll virus | 2 | 4 | RNA | 1 |
| Virus | Cherry mottle leaf virus | 4 | 4 | RNA | 1 |
| Virus | Cherry necrotic rusty mottle virus | 7 | 4 | RNA | 1 |
| Virus | Cherry rasp leaf virus | 11 | 4 | RNA | 1 |
| Virus | Cherry virus A | 2 | 4 | RNA | 1 |
| Virus | Chicken anemia virus | 4 | 2 | DNA | 3 |
| Virus | Chicken astrovirus | 2 | 4 | RNA | 1 |
| Virus | Chickpea chlorosis virus-A | 4 | 2 | DNA | 3 |
| Virus | Chickpea chlorotic dwarf Sudan virus | 4 | 2 | DNA | 3 |
| Virus | Chickpea chlorotic dwarf virus | 4 | 2 | DNA | 3 |
| Virus | Chickpea chlorotic stunt virus | 6 | 4 | RNA | 1 |
| Virus | Chickpea redleaf virus | 4 | 2 | DNA | 3 |
| Virus | Chicory yellow mottle virus large satellite RNA | 1 | NA | RNA | NA |
| Virus | Chicory yellow mottle virus satellite RNA | 1 | NA | RNA | NA |
| Virus | Chicory yellow mottle virus satellite RNA L1 | 1 | NA | NA | NA |
| Virus | Chikungunya virus | 4 | 4 | RNA | 1 |
| Virus | Chilli leaf curl Multan alphasatellite | 1 | 2 | DNA | NA |
| Virus | Chilli leaf curl virus | 6 | 2 | DNA | 3 |
| Virus | Chilli leaf curl virus satellite DNA beta | 1 | 2 | DNA | 3 |
| Virus | Chilli ringspot virus | 12 | 4 | RNA | 1 |
| Virus | Chilli veinal mottle virus | 12 | 4 | RNA | 1 |
| Virus | Chiltepin yellow mosaic virus | 3 | 4 | RNA | 1 |
| Virus | Chimpanzee adenovirus Y25 | 31 | 1 | DNA | NA |
| Virus | Chimpanzee polyomavirus | 5 | 1 | DNA | 3 |
| Virus | Chinese wheat mosaic virus | 7 | 4 | RNA | 1 |
| Virus | Chinese yam necrotic mosaic virus | 10 | 4 | RNA | 1 |
| Virus | Chino del tomate virus | 7 | 2 | DNA | 3 |
| Virus | Chlamydia phage 3 | 8 | 2 | DNA | NA |
| Virus | Chlamydia phage 4 | 8 | 2 | DNA | NA |
| Virus | Chlamydia phage Chp1 | 12 | 2 | DNA | NA |
| Virus | Chlamydia phage Chp2 | 8 | 2 | DNA | NA |
| Virus | Chlamydia phage CPAR39 | 7 | 2 | DNA | NA |
| Virus | Chlamydia phage phiCPG1 | 9 | 2 | DNA | NA |
| Virus | Chloris striate mosaic virus | 5 | 2 | DNA | 3 |
| Virus | Chocolate lily virus A | 9 | 4 | RNA | 1 |
| Virus | Choristoneura fumiferana DEF MNPV | 149 | 1 | DNA | NA |
| Virus | Choristoneura fumiferana MNPV | 146 | 1 | DNA | NA |
| Virus | Choristoneura occidentalis granulovirus | 116 | 1 | DNA | NA |
| Virus | Chronic bee paralysis virus | 7 | 4 | RNA | NA |
| Virus | Chrysanthemum virus B | 6 | 4 | RNA | 1 |
| Virus | Chrysodeixis chalcites nucleopolyhedrovirus | 151 | 1 | DNA | NA |
| Virus | Circovirus-like genome BBC-A | 2 | 2 | DNA | NA |
| Virus | Circovirus-like genome CB-A | 2 | 2 | DNA | NA |
| Virus | Circovirus-like genome CB-B | 3 | 2 | DNA | NA |
| Virus | Circovirus-like genome RW-A | 3 | 2 | DNA | NA |
| Virus | Circovirus-like genome RW-B | 2 | 2 | DNA | NA |
| Virus | Circovirus-like genome RW-C | 2 | 2 | DNA | NA |
| Virus | Circovirus-like genome RW-D | 2 | 2 | DNA | NA |

| Type | Name | Number of genes | Baltimore class | Nucleic Acid | Monophyletic group |
|---|---|---|---|---|---|
| Virus | Circovirus-like genome RW-E | 2 | 2 | DNA | NA |
| Virus | Circovirus-like genome SAR-A | 2 | 2 | DNA | NA |
| Virus | Circovirus-like genome SAR-B | 2 | 2 | DNA | NA |
| Virus | Circulifer tenellus virus 1 | 2 | 3 | RNA | NA |
| Virus | Citrus chlorotic dwarf associated virus | 5 | 2 | DNA | 3 |
| Virus | Citrus leaf blotch virus | 3 | 4 | RNA | 1 |
| Virus | Citrus leaf rugose virus | 5 | 4 | RNA | 1 |
| Virus | Citrus leprosis virus C | 10 | 4 | RNA | 1 |
| Virus | Citrus psorosis virus | 4 | 5 | RNA | NA |
| Virus | Citrus sudden death-associated virus | 6 | 4 | RNA | 1 |
| Virus | Citrus tristeza virus | 14 | 4 | RNA | 1 |
| Virus | Citrus variegation virus | 5 | 4 | RNA | 1 |
| Virus | Citrus yellow mosaic virus | 6 | 7 | DNA | 2 |
| Virus | Clanis bilineata nucleopolyhedrosis virus | 129 | 1 | DNA | NA |
| Virus | Classical swine fever virus | 14 | 4 | RNA | 1 |
| Virus | Clavibacter phage CMP1 | 74 | 1 | DNA | 4 |
| Virus | Cleome golden mosaic virus | 3 | 2 | DNA | 3 |
| Virus | Cleome leaf crumple virus | 7 | 2 | DNA | 3 |
| Virus | Cleome leaf crumple virus associated DNA 1 | 1 | NA | NA | NA |
| Virus | Clerodendron yellow mosaic virus | 6 | 2 | DNA | 3 |
| Virus | Clerodendrum golden mosaic China virus | 8 | 2 | DNA | 3 |
| Virus | Clerodendrum golden mosaic virus | 8 | 2 | DNA | 3 |
| Virus | Clitocybe odora virus | 1 | 4 | RNA | 1 |
| Virus | Clitoria yellow mottle virus | 4 | 4 | RNA | 1 |
| Virus | Clostera anachoreta granulovirus | 123 | 1 | DNA | NA |
| Virus | Clostridium phage c-st | 198 | 1 | DNA | 4 |
| Virus | Clostridium phage D-1873 | 145 | NA | DNA | NA |
| Virus | Clostridium phage phi3626 | 51 | 1 | DNA | 4 |
| Virus | Clostridium phage phiC2 | 82 | 1 | DNA | 4 |
| Virus | Clostridium phage phiCD119 | 79 | 1 | DNA | 4 |
| Virus | Clostridium phage phiCD27 | 75 | 1 | DNA | 4 |
| Virus | Clostridium phage phiCD38-2 | 55 | 1 | DNA | 4 |
| Virus | Clostridium phage phiCD6356 | 59 | 1 | DNA | 4 |
| Virus | Clostridium phage phiCP39-O | 62 | 1 | DNA | 4 |
| Virus | Clostridium phage phiCP7R | 26 | 1 | DNA | 4 |
| Virus | Clostridium phage phiCPV4 | 28 | 1 | DNA | 4 |
| Virus | Clostridium phage phiCTP1 | 86 | 1 | DNA | 4 |
| Virus | Clostridium phage phiMMP02 | 76 | 1 | DNA | 4 |
| Virus | Clostridium phage phiMMP04 | 51 | 1 | DNA | 4 |
| Virus | Clostridium phage PhiS63 | 43 | 1 | DNA | 4 |
| Virus | Clostridium phage phiSM101 | 53 | 1 | DNA | NA |
| Virus | Clostridium phage phiZP2 | 27 | 1 | DNA | 4 |
| Virus | Clover yellow mosaic virus | 5 | 4 | RNA | 1 |
| Virus | Clover yellow vein virus | 12 | 4 | RNA | 1 |
| Virus | Cocksfoot mild mosaic virus | 6 | 4 | RNA | 1 |
| Virus | Cocksfoot mottle virus | 7 | 4 | RNA | 1 |
| Virus | Cocksfoot streak virus | 12 | 4 | RNA | 1 |
| Virus | Coconut foliar decay virus | 6 | 2 | DNA | NA |
| Virus | Coleus vein necrosis virus | 6 | 4 | RNA | 1 |
| Virus | Colobus guereza papillomavirus type 2 | 6 | 1 | DNA | 3 |
| Virus | Colorado tick fever virus | 13 | 3 | RNA | NA |
| Virus | Columbid circovirus | 5 | 2 | DNA | 3 |
| Virus | Colwellia phage 9A | 149 | 1 | DNA | 4 |
| Virus | Commelina yellow mottle virus | 3 | 7 | DNA | 2 |
| Virus | Common chimpanzee papillomavirus 1 | 8 | 1 | DNA | 3 |
| Virus | Common-moorhen coronavirus HKU21 | 9 | 4 | RNA | NA |
| Virus | Coniothyrium minitans RNA virus | 2 | 3 | RNA | NA |
| Virus | Corchorus golden mosaic virus | 7 | 2 | DNA | 3 |
| Virus | Corchorus yellow spot virus | 6 | 2 | DNA | 3 |
| Virus | Corynebacterium phage BFK20 | 56 | 1 | DNA | 4 |
| Virus | Corynebacterium phage P1201 | 97 | 1 | DNA | 4 |
| Virus | Cotesia congregata bracovirus | 155 | 1 | DNA | NA |
| Virus | Cotia virus SPAn232 | 185 | 1 | DNA | 5 |
| Virus | Cotton leaf crumple virus | 6 | 2 | DNA | 3 |
| Virus | Cotton leaf curl Alabad virus | 6 | 2 | DNA | 3 |
| Virus | Cotton leaf curl Bangalore betasatellite | 1 | 2 | DNA | NA |
| Virus | Cotton leaf curl Bangalore virus | 6 | 2 | DNA | 3 |
| Virus | Cotton leaf curl betasatellite | 1 | 2 | DNA | NA |
| Virus | Cotton leaf curl Burewala alphasatellite | 1 | 2 | DNA | NA |
| Virus | Cotton leaf curl Burewala betasatellite | 1 | 2 | DNA | NA |
| Virus | Cotton leaf curl Burewala virus | 5 | 2 | DNA | 3 |
| Virus | Cotton leaf curl Gezira alphasatellite | 1 | 2 | DNA | NA |
| Virus | Cotton leaf curl Gezira beta | 1 | 2 | DNA | NA |
| Virus | Cotton leaf curl Gezira betasatellite | 2 | 2 | DNA | 3 |
| Virus | Cotton leaf curl Gezira virus | 6 | 2 | DNA | 3 |
| Virus | Cotton leaf curl Kokhran virus | 6 | 2 | DNA | 3 |
| Virus | Cotton leaf curl Multan alphasatellite | 1 | NA | NA | NA |
| Virus | Cotton leaf curl Multan betasatellite | 1 | 2 | DNA | 3 |
| Virus | Cotton leaf curl Multan virus | 6 | 2 | DNA | 3 |
| Virus | Cotton leaf curl Rajasthan virus | 6 | 2 | DNA | 3 |

| Type | Name | Number of genes | Baltimore class | Nucleic Acid | Monophyletic group |
|---|---|---|---|---|---|
| Virus | Cotton leaf curl virus | 7 | 2 | DNA | 3 |
| Virus | Cotton leaf curl virus associated DNA 1 isolate Lucknow | 1 | NA | NA | NA |
| Virus | Cotton leaf curl virus betasatellite | 1 | NA | NA | NA |
| Virus | Cotton leafroll dwarf virus | 6 | 4 | RNA | 1 |
| Virus | Courdo7 | 1337 | 1 | DNA | 5 |
| Virus | Cowpea aphid-borne mosaic virus | 12 | 4 | RNA | 1 |
| Virus | Cowpea chlorotic mottle virus | 4 | 4 | RNA | 1 |
| Virus | Cowpea mild mottle virus | 6 | 4 | RNA | 1 |
| Virus | Cowpea mosaic virus | 12 | 4 | RNA | 1 |
| Virus | Cowpea mottle virus | 6 | 4 | RNA | 1 |
| Virus | Cowpea severe leaf curl-associated DNA beta | 1 | 2 | DNA | 3 |
| Virus | Cowpea severe mosaic virus | 10 | 4 | RNA | 1 |
| Virus | Cowpox virus | 233 | 1 | DNA | 5 |
| Virus | Crassocephalum yellow vein virus - Jinghong | 6 | 2 | DNA | 3 |
| Virus | Cricket paralysis virus | 2 | 4 | RNA | 1 |
| Virus | Crimean-Congo hemorrhagic fever virus | 3 | 5 | RNA | NA |
| Virus | Croceibacter phage P2559S | 68 | 1 | DNA | NA |
| Virus | Crocodilepox virus | 173 | 1 | DNA | 5 |
| Virus | Crocuta crocuta papillomavirus 1 | 7 | 1 | DNA | 3 |
| Virus | Cronobacter phage CR3 | 265 | 1 | DNA | 4 |
| Virus | Cronobacter phage phiES15 | 52 | 1 | DNA | 4 |
| Virus | Cronobacter phage vB_CsaM_GAP161 | 275 | 1 | DNA | 4 |
| Virus | Cronobacter phage vB_CsaM_GAP31 | 269 | 1 | DNA | 4 |
| Virus | Cronobacter phage vB_CsaM_GAP32 | 545 | 1 | DNA | 4 |
| Virus | Cronobacter phage vB_CsaP_GAP52 | 115 | 1 | DNA | 4 |
| Virus | Croton yellow vein mosaic alphasatellite | 1 | 2 | DNA | NA |
| Virus | Croton yellow vein mosaic betasatellite | 1 | 2 | DNA | 3 |
| Virus | Croton yellow vein mosaic virus | 7 | 2 | DNA | 3 |
| Virus | Croton yellow vein virus | 6 | 2 | DNA | 3 |
| Virus | Crow polyomavirus | 6 | 1 | DNA | 3 |
| Virus | Cryphonectria hypovirus 1 | 2 | 3 | RNA | NA |
| Virus | Cryphonectria hypovirus 2 | 2 | 3 | RNA | NA |
| Virus | Cryphonectria hypovirus 3 | 1 | 3 | RNA | NA |
| Virus | Cryphonectria hypovirus 4 | 1 | 3 | RNA | NA |
| Virus | Cryphonectria parasitica mitovirus 1-NB631 | 1 | 4 | RNA | 1 |
| Virus | Cryptophlebia leucotreta granulovirus | 128 | 1 | DNA | NA |
| Virus | Cucumber Bulgarian latent virus | 5 | 4 | RNA | 1 |
| Virus | Cucumber fruit mottle mosaic virus | 3 | 4 | RNA | 1 |
| Virus | Cucumber green mottle mosaic virus | 4 | 4 | RNA | 1 |
| Virus | Cucumber leaf spot virus | 4 | 4 | RNA | 1 |
| Virus | Cucumber mosaic virus | 5 | 4 | RNA | 1 |
| Virus | Cucumber mottle virus | 4 | 4 | RNA | 1 |
| Virus | Cucumber necrosis virus | 5 | 4 | RNA | 1 |
| Virus | Cucumber vein yellowing virus | 11 | 4 | RNA | 1 |
| Virus | Cucurbit aphid-borne yellows virus | 6 | 4 | RNA | 1 |
| Virus | Cucurbit chlorotic yellows virus | 13 | 4 | RNA | 1 |
| Virus | Cucurbit leaf crumple virus | 6 | 2 | DNA | 3 |
| Virus | Cucurbit yellow stunting disorder virus | 14 | 4 | RNA | 1 |
| Virus | Culex flavivirus | 14 | 4 | RNA | 1 |
| Virus | Culex nigripalpus NPV | 109 | 1 | DNA | NA |
| Virus | Culex originated Tymoviridae-like virus | 2 | 4 | RNA | 1 |
| Virus | Culex pipiens densovirus | 4 | 2 | DNA | 3 |
| Virus | Cupixi virus | 4 | 5 | RNA | NA |
| Virus | Curvularia thermal tolerance virus | 4 | 3 | RNA | NA |
| Virus | Cutthroat trout virus | 7 | 4 | RNA | 1 |
| Virus | Cyanophage 9515-10a | 55 | 1 | DNA | 4 |
| Virus | Cyanophage NATL1A-7 | 65 | 1 | DNA | 4 |
| Virus | Cyanophage NATL2A-133 | 62 | 1 | DNA | 4 |
| Virus | Cyanophage P-SSP2 | 56 | 1 | DNA | NA |
| Virus | Cyanophage Pf-WMP4 | 45 | 1 | DNA | 4 |
| Virus | Cyanophage PSS2 | 131 | 1 | DNA | 4 |
| Virus | Cyanophage Syn5 | 61 | 1 | DNA | 4 |
| Virus | Cycad leaf necrosis virus | 3 | 7 | DNA | 2 |
| Virus | Cycas necrotic stunt virus | 9 | 4 | RNA | 1 |
| Virus | Cyclovirus bat/USA/2009 | 2 | 2 | DNA | NA |
| Virus | Cyclovirus NGchicken15/NGA/2009 | 2 | 2 | DNA | 3 |
| Virus | Cyclovirus PKgoat11/PAK/2009 | 2 | 2 | DNA | 3 |
| Virus | Cyclovirus PKgoat21/PAK/2009 | 2 | 2 | DNA | 3 |
| Virus | Cydia pomonella granulovirus | 143 | 1 | DNA | NA |
| Virus | Cymbidium mosaic virus | 5 | 4 | RNA | 1 |
| Virus | Cymbidium ringspot virus | 5 | 4 | RNA | 1 |
| Virus | Cymbidium ringspot virus satellite RNA | 1 | NA | RNA | NA |
| Virus | Cynomolgus macaque cytomegalovirus strain Ottawa | 274 | 1 | DNA | NA |
| Virus | Cypovirus 1 | 10 | 3 | RNA | NA |
| Virus | Cypovirus 14 | 11 | 3 | RNA | NA |
| Virus | Cypovirus 5 | 6 | 3 | RNA | NA |
| Virus | Cyprinid herpesvirus 3 | 163 | 1 | DNA | NA |
| Virus | Dahlia mosaic virus | 8 | 7 | DNA | 2 |
| Virus | Dalechampia chlorotic mosaic virus | 7 | 2 | DNA | 3 |
| Virus | Daphne mosaic virus | 12 | 4 | RNA | 1 |

| Type | Name | Number of genes | Baltimore class | Nucleic Acid | Monophyletic group |
|---|---|---|---|---|---|
| Virus | Daphne virus S | 6 | 4 | RNA | 1 |
| Virus | Dasheen mosaic virus | 12 | 4 | RNA | 1 |
| Virus | Datura leaf distortion virus | 7 | 2 | DNA | 3 |
| Virus | Deerpox virus W-1170-84 | 170 | 1 | DNA | 5 |
| Virus | Deerpox virus W-848-83 | 169 | 1 | DNA | 5 |
| Virus | Deformed wing virus | 1 | 4 | RNA | 1 |
| Virus | Deftia phage phiW-14 | 236 | 1 | DNA | 4 |
| Virus | Deltapapillomavirus 1 | 15 | 1 | DNA | 3 |
| Virus | Deltapapillomavirus 2 | 9 | 1 | DNA | 3 |
| Virus | Dendrolimus punctatus densovirus | 3 | 2 | DNA | 3 |
| Virus | Dendrolimus punctatus tetravirus | 3 | 4 | RNA | 1 |
| Virus | Dengue virus 1 | 14 | 4 | RNA | 1 |
| Virus | Dengue virus 2 | 14 | 4 | RNA | 1 |
| Virus | Dengue virus 3 | 14 | 4 | RNA | 1 |
| Virus | Dengue virus 4 | 14 | 4 | RNA | 1 |
| Virus | Desmodium leaf distortion virus | 6 | 2 | DNA | 3 |
| Virus | Diaporthe ambigua RNA virus 1 | 2 | 4 | RNA | NA |
| Virus | Diascia yellow mottle virus | 3 | 4 | RNA | 1 |
| Virus | Diatraea saccharalis densovirus | 7 | 2 | DNA | 3 |
| Virus | Dicliptera yellow mottle virus | 6 | 2 | DNA | 3 |
| Virus | Digitaria ciliaris striate mosaic virus | 4 | 2 | DNA | 3 |
| Virus | Digitaria didactyla striate mosaic virus | 4 | 2 | DNA | 3 |
| Virus | Digitaria streak virus | 4 | 2 | DNA | 3 |
| Virus | Dioscorea bacilliform virus | 3 | 7 | DNA | 2 |
| Virus | Diplodia scrobiculata RNA virus 1 | 2 | 3 | RNA | NA |
| Virus | Discula destructiva virus 1 | 2 | 3 | RNA | 1 |
| Virus | Discula destructiva virus 2 | 2 | 3 | RNA | 1 |
| Virus | Diuris virus A | 7 | 4 | RNA | 1 |
| Virus | Diuris virus B | 7 | 4 | RNA | NA |
| Virus | Dobrava-Belgrade virus | 3 | 5 | RNA | NA |
| Virus | Dolichos yellow mosaic virus | 6 | 2 | DNA | 3 |
| Virus | Dolphin morbillivirus | 7 | 5 | RNA | NA |
| Virus | Donggang virus | 1 | 4 | RNA | 1 |
| Virus | Dracaena mottle virus | 7 | 7 | DNA | 2 |
| Virus | Dragonfly-associated microphage 1 | 5 | 2 | DNA | NA |
| Virus | Drosophila A virus | 2 | 3 | RNA | NA |
| Virus | Drosophila C virus | 2 | 4 | RNA | 1 |
| Virus | Drosophila melanogaster sigma virus AP30 | 6 | 5 | RNA | NA |
| Virus | Drosophila melanogaster totivirus SW-2009a | 2 | 3 | RNA | NA |
| Virus | Drosophila x virus | 2 | 3 | RNA | NA |
| Virus | Duck adenovirus A | 60 | 1 | DNA | NA |
| Virus | Duck astrovirus C-NGB | 3 | 4 | RNA | 1 |
| Virus | Duck circovirus | 4 | 2 | DNA | 3 |
| Virus | Duck egg-drop syndrome virus | 1 | 4 | RNA | 1 |
| Virus | Duck flavivirus TA | 1 | 4 | RNA | 1 |
| Virus | Duck hepatitis A virus 1 | 1 | 4 | RNA | 1 |
| Virus | Duck hepatitis A virus 3 | 1 | 4 | RNA | 1 |
| Virus | Duck hepatitis B virus | 7 | 7 | DNA | 2 |
| Virus | Dugbe virus | 4 | 5 | RNA | NA |
| Virus | Dulcamara mottle virus | 3 | 4 | RNA | 1 |
| Virus | East African cassava mosaic Cameroon virus | 8 | 2 | DNA | 3 |
| Virus | East African cassava mosaic Kenya virus | 8 | 2 | DNA | 3 |
| Virus | East African cassava mosaic virus | 9 | 2 | DNA | 3 |
| Virus | East African cassava mosaic Zanzibar virus | 8 | 2 | DNA | 3 |
| Virus | East Asian Passiflora virus | 12 | 4 | RNA | 1 |
| Virus | Eastern equine encephalitis virus | 19 | 4 | RNA | 1 |
| Virus | Ebola virus - Mayinga | Zaire | 1976 | | RNA |
| Virus | Ectocarpus siliculosus virus 1 | 240 | 1 | DNA | 5 |
| Virus | Ectromelia virus | 173 | 1 | DNA | 5 |
| Virus | Ectropis obliqua NPV | 126 | 1 | DNA | NA |
| Virus | Ectropis obliqua picorna-like virus | 1 | 4 | RNA | 1 |
| Virus | Edwardsiella phage KF-1 | 48 | 1 | DNA | 4 |
| Virus | Eggplant mosaic virus | 3 | 4 | RNA | 1 |
| Virus | Eidolon helvum parvovirus 1 | 4 | 2 | DNA | 3 |
| Virus | Eimeria brunetti RNA virus 1 | 2 | 3 | RNA | NA |
| Virus | Eliat virus | 3 | 4 | RNA | 1 |
| Virus | Elm mottle virus | 5 | 4 | RNA | 1 |
| Virus | Emilia yellow vein virus-associated DNA beta | 1 | 2 | DNA | NA |
| Virus | Emiliania huxleyi virus 86 | 597 | 1 | DNA | 5 |
| Virus | Encephalomyocarditis virus | 13 | 4 | RNA | 1 |
| Virus | Entebbe bat virus | 1 | 4 | RNA | 1 |
| Virus | Enterobacter phage IME11 | 91 | 1 | DNA | 4 |
| Virus | Enterobacteria phage 13a | 55 | 1 | DNA | 4 |
| Virus | Enterobacteria phage 285P | 47 | 1 | DNA | 4 |
| Virus | Enterobacteria phage 933W | 80 | 1 | DNA | 4 |
| Virus | Enterobacteria phage alpha3 | 10 | 2 | DNA | NA |
| Virus | Enterobacteria phage BA14 | 52 | 1 | DNA | 4 |
| Virus | Enterobacteria phage BZ13 | 4 | 4 | RNA | 1 |
| Virus | Enterobacteria phage CC31 | 279 | 1 | DNA | 4 |
| Virus | Enterobacteria phage EcoDS1 | 53 | 1 | DNA | 4 |

| Type | Name | Number of genes | Baltimore class | Nucleic Acid | Monophyletic group |
|---|---|---|---|---|---|
| Virus | Enterobacteria phage EPS7 | 170 | 1 | DNA | 4 |
| Virus | Enterobacteria phage epsilon15 | 51 | 1 | DNA | NA |
| Virus | Enterobacteria phage ES18 | 79 | 1 | DNA | 4 |
| Virus | Enterobacteria phage FI sensu lato | 4 | 4 | RNA | 1 |
| Virus | Enterobacteria phage G4 sensu lato | 11 | 2 | DNA | NA |
| Virus | Enterobacteria phage HK022 | 57 | 1 | DNA | 4 |
| Virus | Enterobacteria phage HK620 | 58 | 1 | DNA | NA |
| Virus | Enterobacteria phage HK97 | 61 | 1 | DNA | 4 |
| Virus | Enterobacteria phage HX01 | 62 | 1 | DNA | 4 |
| Virus | Enterobacteria phage I2-2 | 9 | 2 | DNA | NA |
| Virus | Enterobacteria phage ID18 sensu lato | 11 | 2 | DNA | NA |
| Virus | Enterobacteria phage ID2 Moscow/ID/2001 | 11 | 2 | DNA | NA |
| Virus | Enterobacteria phage If1 | 10 | 2 | DNA | NA |
| Virus | Enterobacteria phage Ike | 12 | 2 | DNA | NA |
| Virus | Enterobacteria phage IME08 | 253 | 1 | DNA | 4 |
| Virus | Enterobacteria phage JK06 | 82 | 1 | DNA | 4 |
| Virus | Enterobacteria phage JL1 | 56 | 1 | DNA | 4 |
| Virus | Enterobacteria phage JS10 | 265 | 1 | DNA | 4 |
| Virus | Enterobacteria phage JS98 | 266 | 1 | DNA | 4 |
| Virus | Enterobacteria phage JSE | 277 | 1 | DNA | 4 |
| Virus | Enterobacteria phage K1-5 | 52 | 1 | DNA | 4 |
| Virus | Enterobacteria phage K1E | 62 | 1 | DNA | 4 |
| Virus | Enterobacteria phage K1F | 43 | 1 | DNA | 4 |
| Virus | Enterobacteria phage K30 | 49 | 1 | DNA | 4 |
| Virus | Enterobacteria phage lambda | 74 | 1 | DNA | 4 |
| Virus | Enterobacteria phage LKA1 | 56 | 1 | DNA | 4 |
| Virus | Enterobacteria phage M13 | 10 | 2 | DNA | NA |
| Virus | Enterobacteria phage Min27 | 83 | 1 | DNA | 4 |
| Virus | Enterobacteria phage MS2 | 4 | 4 | RNA | 1 |
| Virus | Enterobacteria phage Mu | 55 | 1 | DNA | 4 |
| Virus | Enterobacteria phage N15 | 60 | 1 | DNA | 4 |
| Virus | Enterobacteria phage NJ01 | 109 | 1 | DNA | 4 |
| Virus | Enterobacteria phage P1 | 110 | 1 | DNA | 4 |
| Virus | Enterobacteria phage P2 | 43 | 1 | DNA | 4 |
| Virus | Enterobacteria phage P22 | 72 | 1 | DNA | 4 |
| Virus | Enterobacteria phage P4 | 14 | 1 | DNA | 4 |
| Virus | Enterobacteria phage Phi1 | 276 | 1 | DNA | 4 |
| Virus | Enterobacteria phage phiEco32 | 128 | 1 | DNA | 4 |
| Virus | Enterobacteria phage phiEcoM-GJ1 | 75 | 1 | DNA | 4 |
| Virus | Enterobacteria phage phiP27 | 58 | 1 | DNA | 4 |
| Virus | Enterobacteria phage phiX174 sensu lato | 11 | 2 | DNA | NA |
| Virus | Enterobacteria phage PRD1 | 31 | 1 | DNA | NA |
| Virus | Enterobacteria phage PsP3 | 42 | 1 | DNA | 4 |
| Virus | Enterobacteria phage Qbeta | 4 | 4 | RNA | 1 |
| Virus | Enterobacteria phage RB14 | 274 | 1 | DNA | 4 |
| Virus | Enterobacteria phage RB16 | 270 | 1 | DNA | 4 |
| Virus | Enterobacteria phage RB32 | 270 | 1 | DNA | 4 |
| Virus | Enterobacteria phage RB43 | 292 | 1 | DNA | 4 |
| Virus | Enterobacteria phage RB49 | 279 | 1 | DNA | 4 |
| Virus | Enterobacteria phage RB51 | 273 | 1 | DNA | 4 |
| Virus | Enterobacteria phage RB69 | 273 | 1 | DNA | 4 |
| Virus | Enterobacteria phage RTP | 75 | 1 | DNA | 4 |
| Virus | Enterobacteria phage Sf6 | 66 | 1 | DNA | NA |
| Virus | Enterobacteria phage SfV | 53 | 1 | DNA | 4 |
| Virus | Enterobacteria phage SP6 | 52 | 1 | DNA | 4 |
| Virus | Enterobacteria phage SPC35 | 145 | 1 | DNA | 4 |
| Virus | Enterobacteria phage SSL-2009a | 52 | 1 | DNA | 4 |
| Virus | Enterobacteria phage St-1 | 11 | 2 | DNA | NA |
| Virus | Enterobacteria phage ST104 | 63 | 1 | DNA | 4 |
| Virus | Enterobacteria phage ST64T | 65 | 1 | DNA | NA |
| Virus | Enterobacteria phage T1 | 78 | 1 | DNA | 4 |
| Virus | Enterobacteria phage T3 | 55 | 1 | DNA | 4 |
| Virus | Enterobacteria phage T4 | 278 | 1 | DNA | 4 |
| Virus | Enterobacteria phage T5 | 163 | 1 | DNA | 4 |
| Virus | Enterobacteria phage T7 | 60 | 1 | DNA | 4 |
| Virus | Enterobacteria phage TLS | 87 | 1 | DNA | 4 |
| Virus | Enterobacteria phage vB_EcoM_ACG-C40 | 282 | 1 | DNA | 4 |
| Virus | Enterobacteria phage vB_EcoM-VR7 | 293 | 1 | DNA | 4 |
| Virus | Enterobacteria phage vB_EcoP_ACG-C91 | 56 | 1 | DNA | 4 |
| Virus | Enterobacteria phage vB_EcoS_ACG-M12 | 78 | 1 | DNA | 4 |
| Virus | Enterobacteria phage VT2-Sakai | 83 | 1 | DNA | 4 |
| Virus | Enterobacteria phage WA13 sensu lato | 10 | 2 | DNA | NA |
| Virus | Enterobacteria phage YYZ-2008 | 75 | NA | NA | NA |
| Virus | Enterococcus phage BC-611 | 57 | 1 | DNA | 4 |
| Virus | Enterococcus phage EF62phi | 48 | NA | NA | NA |
| Virus | Enterococcus phage EFAP-1 | 24 | 1 | DNA | 4 |
| Virus | Enterococcus phage EFRM31 | 23 | 1 | DNA | 4 |
| Virus | Enterococcus phage phiEf11 | 65 | 1 | DNA | 4 |
| Virus | Enterococcus phage phiEF24C | 221 | 1 | DNA | 4 |
| Virus | Enterococcus phage phiFL1A | 61 | 1 | DNA | 4 |

| Type | Name | Number of genes | Baltimore class | Nucleic Acid | Monophyletic group |
|---|---|---|---|---|---|
| Virus | Enterococcus phage phiFL2A | 63 | 1 | DNA | 4 |
| Virus | Enterococcus phage phiFL3A | 64 | 1 | DNA | 4 |
| Virus | Enterococcus phage phiFL4A | 55 | 1 | DNA | 4 |
| Virus | Enzootic nasal tumour virus of goats | 4 | 6 | RNA | 2 |
| Virus | Epinephelus tauvina nervous necrosis virus | 3 | 4 | RNA | 1 |
| Virus | Epinotia aporema granulovirus | 132 | 1 | DNA | NA |
| Virus | Epiphyas postvittana NPV | 136 | 1 | DNA | NA |
| Virus | Epirus cherry virus | 3 | 4 | RNA | 1 |
| Virus | Epizootic hemorrhagic disease virus (serotype 1 / strain New Jersey) | 11 | 3 | RNA | NA |
| Virus | Equid herpesvirus 1 | 80 | 1 | DNA | NA |
| Virus | Equid herpesvirus 2 | 79 | 1 | DNA | NA |
| Virus | Equid herpesvirus 4 | 79 | 1 | DNA | NA |
| Virus | Equid herpesvirus 8 | 81 | 1 | DNA | NA |
| Virus | Equid herpesvirus 9 | 80 | 1 | DNA | NA |
| Virus | Equine arteritis virus | 22 | 4 | RNA | NA |
| Virus | Equine coronavirus | 11 | 4 | RNA | NA |
| Virus | equine foamy virus | 5 | 6 | RNA | 2 |
| Virus | Equine infectious anemia virus | 4 | 6 | RNA | 2 |
| Virus | Equine papillomavirus 2 | 6 | 1 | DNA | 3 |
| Virus | Equine papillomavirus 3 | 7 | 1 | DNA | 3 |
| Virus | Equine polyomavirus | 5 | 1 | DNA | 3 |
| Virus | Equine rhinitis A virus | 14 | 4 | RNA | 1 |
| Virus | Equine rhinitis B virus 1 | 13 | 4 | RNA | 1 |
| Virus | Equine rhinitis B virus 2 | 13 | 4 | RNA | 1 |
| Virus | Equinus papillomavirus | 6 | 1 | DNA | 3 |
| Virus | Equus caballus papillomavirus 1 | 7 | 1 | DNA | 3 |
| Virus | Eragrostis curvula streak virus | 3 | 2 | DNA | 3 |
| Virus | Eragrostis minor streak virus | 4 | 2 | DNA | 3 |
| Virus | Eragrostis streak virus | 3 | 2 | DNA | 3 |
| Virus | Erectites yellow mosaic virus | 6 | 2 | DNA | 3 |
| Virus | Erectites yellow mosaic virus satellite DNA beta | 1 | 2 | DNA | 3 |
| Virus | Erethizon dorsatum papillomavirus 1 | 7 | 1 | DNA | 3 |
| Virus | Erinaceus europaeus papillomavirus | 7 | 1 | DNA | 3 |
| Virus | Erwinia amylovora phage Era103 | 53 | 1 | DNA | 4 |
| Virus | Erwinia phage PEp14 | 64 | 1 | DNA | 4 |
| Virus | Erwinia phage phiEa104 | 118 | 1 | DNA | 4 |
| Virus | Erwinia phage phiEa21-4 | 118 | 1 | DNA | 4 |
| Virus | Erwinia phage phiEt88 | 68 | 1 | DNA | 4 |
| Virus | Erysimum latent virus | 3 | 4 | RNA | 1 |
| Virus | Escherichia phage bV_EcoS_AKFV33 | 160 | 1 | DNA | 4 |
| Virus | Escherichia phage D108 | 57 | 1 | DNA | 4 |
| Virus | Escherichia phage HK639 | 76 | 1 | DNA | 4 |
| Virus | Escherichia phage HK75 | 58 | 1 | DNA | 4 |
| Virus | Escherichia phage KBNP135 | 120 | NA | NA | NA |
| Virus | Escherichia phage KBNP21 | 82 | 1 | DNA | 4 |
| Virus | Escherichia phage N4 | 72 | 1 | DNA | 4 |
| Virus | Escherichia phage P13374 | 79 | 1 | DNA | 4 |
| Virus | Escherichia phage phiV10 | 55 | 1 | DNA | 4 |
| Virus | Escherichia phage rv5 | 233 | 1 | DNA | 4 |
| Virus | Escherichia phage vB_EcoM_CBA120 | 204 | 1 | DNA | 4 |
| Virus | Escherichia phage vB_EcoP_G7C | 79 | 1 | DNA | 4 |
| Virus | Escherichia phage wV8 | 140 | 1 | DNA | 4 |
| Virus | Espirito Santo virus | 6 | 3 | RNA | NA |
| Virus | Etapapillomavirus 1 | 6 | 1 | DNA | 3 |
| Virus | Eupatorium vein clearing virus | 6 | 7 | DNA | 2 |
| Virus | Eupatorium yellow vein betasatellite | 1 | 2 | DNA | 3 |
| Virus | Eupatorium yellow vein virus | 6 | 2 | DNA | 3 |
| Virus | Euphorbia leaf curl virus | 6 | 2 | DNA | 3 |
| Virus | Euphorbia mosaic virus | 6 | 2 | DNA | 3 |
| Virus | Euphorbia mosaic virus associated DNA 1 | 1 | NA | NA | NA |
| Virus | Euphorbia yellow mosaic virus | 6 | 2 | DNA | 3 |
| Virus | Euproctis pseudoconspersa nucleopolyhedrovirus | 139 | 1 | DNA | NA |
| Virus | Euprosterna elaeasa virus | 5 | 4 | RNA | 1 |
| Virus | European bat lyssavirus 1 | 5 | 5 | RNA | NA |
| Virus | European bat lyssavirus 2 | 5 | 5 | RNA | NA |
| Virus | European brown hare syndrome virus | 9 | 4 | RNA | 1 |
| Virus | European catfish virus | 136 | 1 | DNA | 5 |
| Virus | European mountain ash ringspot-associated virus | 4 | 4 | RNA | 1 |
| Virus | Eyach virus | 13 | 3 | RNA | NA |
| Virus | Faba bean necrotic stunt virus | 8 | 2 | DNA | NA |
| Virus | Faba bean necrotic yellows virus | 10 | 2 | DNA | NA |
| Virus | Feldmannia species virus | 150 | 1 | DNA | 5 |
| Virus | Felid herpesvirus 1 | 77 | 1 | DNA | NA |
| Virus | Feline bocavirus | 4 | 2 | DNA | 3 |
| Virus | Feline calicivirus | 13 | 4 | RNA | 1 |
| Virus | Feline foamy virus | 4 | 6 | RNA | 2 |
| Virus | Feline immunodeficiency virus | 7 | 6 | RNA | 2 |
| Virus | Feline infectious peritonitis virus | 9 | 4 | RNA | NA |
| Virus | Feline leukemia virus | 13 | 6 | RNA | 2 |
| Virus | Feline picornavirus | 13 | 4 | RNA | 1 |

| Type | Name | Number of genes | Baltimore class | Nucleic Acid | Monophyletic group |
|---|---|---|---|---|---|
| Virus | Felis domesticus papillomavirus type 1 | 7 | 1 | DNA | 3 |
| Virus | Fenneropenaeus chinensis hepatopancreatic densovirus | 3 | 2 | DNA | 3 |
| Virus | Fer-de-lance virus | 8 | 5 | RNA | NA |
| Virus | Fig badnavirus 1 | 3 | 7 | DNA | 2 |
| Virus | Fig cryptic virus | 2 | 3 | RNA | 1 |
| Virus | Fig fleck-associated virus | 7 | 4 | RNA | 1 |
| Virus | Figwort mosaic virus | 7 | 7 | DNA | 2 |
| Virus | Fiji disease virus | 12 | 3 | RNA | NA |
| Virus | Finch circovirus | 3 | 2 | DNA | 3 |
| Virus | Finch polyomavirus | 6 | 1 | DNA | 3 |
| Virus | Flavobacterium phage 11b | 65 | 1 | DNA | 4 |
| Virus | Flexal virus | 4 | 5 | RNA | NA |
| Virus | Flock house virus | 4 | 4 | RNA | 1 |
| Virus | Foot-and-mouth disease virus - type A | 1 | 4 | RNA | 1 |
| Virus | Foot-and-mouth disease virus - type Asia 1 | 8 | 4 | RNA | 1 |
| Virus | Foot-and-mouth disease virus - type C | 15 | 4 | RNA | 1 |
| Virus | Foot-and-mouth disease virus - type O | 13 | 4 | RNA | 1 |
| Virus | Foot-and-mouth disease virus - type SAT 1 | 1 | 4 | RNA | 1 |
| Virus | Foot-and-mouth disease virus - type SAT 2 | 1 | 4 | RNA | 1 |
| Virus | Foot-and-mouth disease virus - type SAT 3 | 1 | 4 | RNA | 1 |
| Virus | Fort Morgan virus | 13 | 4 | RNA | 1 |
| Virus | Fowl adenovirus A | 77 | 1 | DNA | NA |
| Virus | Fowl adenovirus C | 46 | 1 | DNA | NA |
| Virus | Fowl adenovirus D | 63 | 1 | DNA | NA |
| Virus | Fowl adenovirus E | 46 | 1 | DNA | NA |
| Virus | Fowlpox virus | 261 | 1 | DNA | 5 |
| Virus | Foxtail mosaic virus | 5 | 4 | RNA | 1 |
| Virus | Fragaria chiloensis cryptic virus | 3 | 3 | RNA | 1 |
| Virus | Fragaria chiloensis latent virus | 6 | 4 | RNA | 1 |
| Virus | Francolinus leucoscepus papillomavirus 1 | 8 | 1 | DNA | 3 |
| Virus | Frangipani mosaic virus | 4 | 4 | RNA | 1 |
| Virus | Freesia mosaic virus | 12 | 4 | RNA | 1 |
| Virus | French bean leaf curl betasatellite-Kanpur | 1 | NA | NA | NA |
| Virus | French bean leaf curl virus-Kanpur | 7 | 2 | DNA | 3 |
| Virus | French bean severe leaf curl virus | 2 | 2 | DNA | 3 |
| Virus | Friend murine leukemia virus | 10 | 6 | RNA | 2 |
| Virus | Fritillary virus Y | 12 | 4 | RNA | 1 |
| Virus | Frog adenovirus 1 | 23 | 1 | DNA | NA |
| Virus | Frog virus 3 | 99 | 1 | DNA | 5 |
| Virus | Fujinami sarcoma virus | 5 | 6 | RNA | 2 |
| Virus | Fusarium graminearum dsRNA mycovirus-1 | 4 | 3 | RNA | NA |
| Virus | Fusarium graminearum dsRNA mycovirus-3 | 2 | 3 | RNA | NA |
| Virus | Fusarium graminearum dsRNA mycovirus-4 | 3 | 3 | RNA | NA |
| Virus | Fusarium poae virus 1 | 2 | 3 | RNA | 1 |
| Virus | Galinsoga mosaic virus | 5 | 4 | RNA | 1 |
| Virus | Galleria mellonella densovirus | 7 | 2 | DNA | 3 |
| Virus | Gallid herpesvirus 1 | 79 | 1 | DNA | NA |
| Virus | Gallid herpesvirus 2 | 85 | 1 | DNA | NA |
| Virus | Gallid herpesvirus 3 | 76 | 1 | DNA | NA |
| Virus | Gammapapillomavirus HPV127 | 7 | 1 | DNA | 3 |
| Virus | Garlic common latent virus | 6 | 4 | RNA | 1 |
| Virus | Garlic latent virus | 6 | 4 | RNA | 1 |
| Virus | Garlic virus A | 6 | 4 | RNA | 1 |
| Virus | Garlic virus C | 6 | 4 | RNA | 1 |
| Virus | Garlic virus E | 8 | 4 | RNA | 1 |
| Virus | Garlic virus X | 6 | 4 | RNA | 1 |
| Virus | Gayfeather mild mottle virus | 5 | 4 | RNA | 1 |
| Virus | GB virus A | 9 | 4 | RNA | 1 |
| Virus | GB virus C | 9 | 4 | RNA | 1 |
| Virus | Geobacillus phage GBSV1 | 54 | 1 | DNA | NA |
| Virus | Geobacillus virus E2 | 71 | 1 | DNA | NA |
| Virus | Getah virus | 4 | 4 | RNA | 1 |
| Virus | Giardia lamblia virus | 2 | 3 | RNA | NA |
| Virus | Gibbon ape leukemia virus | 12 | 6 | RNA | 2 |
| Virus | Gill-associated virus | 5 | 4 | RNA | NA |
| Virus | Glossina pallidipes salivary gland hypertrophy virus | 160 | 1 | DNA | NA |
| Virus | Glypta fumiferanae ichnovirus | 103 | 1 | DNA | NA |
| Virus | Goatpox virus Pellor | 150 | 1 | DNA | 5 |
| Virus | Golden Gate virus | 4 | 5 | RNA | NA |
| Virus | Goose adenovirus 4 | 34 | 1 | DNA | NA |
| Virus | Goose circovirus | 4 | 2 | DNA | 3 |
| Virus | Goose hemorrhagic polyomavirus | 6 | 1 | DNA | 3 |
| Virus | Goose paramyxovirus SF02 | 6 | 5 | RNA | NA |
| Virus | Goose parvovirus | 4 | 2 | DNA | 3 |
| Virus | Gooseberry vein banding virus | 3 | 7 | DNA | 2 |
| Virus | Gordonia phage GRU1 | 94 | 1 | DNA | 4 |
| Virus | Gordonia phage GTE2 | 57 | 1 | DNA | 4 |
| Virus | Gordonia phage GTE5 | 92 | 1 | DNA | 4 |
| Virus | Gordonia phage GTE7 | 103 | 1 | DNA | 4 |
| Virus | Gossypium darwinii symptomless alphasatellite | 1 | 2 | DNA | NA |

| Type | Name | Number of genes | Baltimore class | Nucleic Acid | Monophyletic group |
|---|---|---|---|---|---|
| Virus | Gossypium darwinii symptomless virus | 6 | 2 | DNA | 3 |
| Virus | Gossypium davidsonii symptomless alphasatellite | 1 | 2 | DNA | NA |
| Virus | Gossypium mustilinum symptomless alphasatellite | 1 | 2 | DNA | NA |
| Virus | Gossypium punctatum mild leaf curl virus | 8 | 2 | DNA | 3 |
| Virus | Grapevine Algerian latent virus | 5 | 4 | RNA | 1 |
| Virus | Grapevine Anatolian ringspot virus | 2 | 4 | RNA | 1 |
| Virus | Grapevine berry inner necrosis virus | 3 | 4 | RNA | 1 |
| Virus | Grapevine Bulgarian latent virus | 10 | 4 | RNA | 1 |
| Virus | grapevine chrome mosaic virus | 2 | 4 | RNA | 1 |
| Virus | Grapevine chrome mosaic virus | 7 | 4 | RNA | 1 |
| Virus | Grapevine deformation virus | 7 | 4 | RNA | 1 |
| Virus | Grapevine fanleaf virus | 10 | 4 | RNA | 1 |
| Virus | Grapevine fanleaf virus satellite RNA | 1 | NA | RNA | NA |
| Virus | Grapevine fleck virus | 4 | 4 | RNA | 1 |
| Virus | Grapevine geminivirus | 1 | 2 | DNA | 3 |
| Virus | Grapevine leafroll-associated virus 1 | 10 | 4 | RNA | 1 |
| Virus | Grapevine leafroll-associated virus 10 | 7 | 4 | RNA | 1 |
| Virus | Grapevine leafroll-associated virus 12 | 10 | 4 | RNA | 1 |
| Virus | Grapevine leafroll-associated virus 2 | 9 | 4 | RNA | 1 |
| Virus | Grapevine leafroll-associated virus 3 | 12 | 4 | RNA | 1 |
| Virus | Grapevine leafroll-associated virus 4 | 6 | 4 | RNA | 1 |
| Virus | Grapevine leafroll-associated virus 5 | 7 | 4 | RNA | 1 |
| Virus | Grapevine leafroll-associated virus 6 | 6 | 4 | RNA | 1 |
| Virus | Grapevine leafroll-associated virus 7 | 10 | 4 | RNA | 1 |
| Virus | Grapevine Pinot gris virus | 3 | 4 | RNA | 1 |
| Virus | Grapevine rootstock stem lesion associated virus | 11 | 4 | RNA | 1 |
| Virus | Grapevine Syrah virus 1 | 5 | 4 | RNA | 1 |
| Virus | Grapevine vein-clearing virus | 4 | NA | DNA | 2 |
| Virus | Grapevine virus A | 5 | 4 | RNA | 1 |
| Virus | Grapevine virus B | 5 | 4 | RNA | 1 |
| Virus | Grapevine virus E | 5 | 4 | RNA | 1 |
| Virus | Grapevine virus F | 5 | 4 | RNA | NA |
| Virus | Great Island virus | 11 | 3 | RNA | NA |
| Virus | Gremmeniella abietina mitochondrial RNA virus S2 | 1 | 4 | RNA | 1 |
| Virus | Gremmeniella abietina RNA virus L1 | 2 | 3 | RNA | NA |
| Virus | Gremmeniella abietina RNA virus L2 | 2 | 3 | RNA | NA |
| Virus | Gremmeniella abietina RNA virus MS1 | 3 | 3 | RNA | 1 |
| Virus | Gremmeniella abietina RNA virus MS2 | 3 | 3 | RNA | 1 |
| Virus | Gremmeniella abietina type B RNA virus XL1 | 1 | 3 | RNA | NA |
| Virus | Ground squirrel hepatitis virus | 5 | 7 | DNA | 2 |
| Virus | Groundnut bud necrosis virus | 5 | 5 | RNA | NA |
| Virus | Groundnut ringspot and Tomato chlorotic spot virus reassortant | 5 | 5 | RNA | NA |
| Virus | Groundnut rosette virus | 4 | 4 | RNA | 1 |
| Virus | Gryllus bimaculatus nudivirus | 98 | 1 | DNA | NA |
| Virus | Guanarito virus | 4 | 5 | RNA | NA |
| Virus | Gull circovirus | 3 | 2 | DNA | 3 |
| Virus | Gyrovirus 4 | 2 | 2 | DNA | 3 |
| Virus | Gyrovirus GyV3 | 3 | 2 | DNA | 3 |
| Virus | Haemophilus phage Aaphi23 | 66 | 1 | DNA | 4 |
| Virus | Haemophilus phage HP1 | 42 | 1 | DNA | 4 |
| Virus | Haemophilus phage HP2 | 37 | 1 | DNA | 4 |
| Virus | Halastavi arva RNA virus | 2 | 4 | RNA | NA |
| Virus | Haloarcula hispanica pleomorphic virus 1 | 9 | 1 | DNA | NA |
| Virus | Haloarcula phage SH1 | 56 | 1 | DNA | NA |
| Virus | Halocynthia phage JM-2012 | 173 | 1 | DNA | 4 |
| Virus | Halogeometricum pleomorphic virus 1 | 16 | 2 | DNA | NA |
| Virus | Halomonas phage phiHAP-1 | 46 | 1 | DNA | 4 |
| Virus | Halorubrum phage HF2 | 114 | 1 | DNA | NA |
| Virus | Halorubrum pleomorphic virus 1 | 10 | 2 | DNA | NA |
| Virus | Halorubrum pleomorphic virus 2 | 16 | 2 | DNA | NA |
| Virus | Halorubrum pleomorphic virus 3 | 13 | 2 | DNA | 3 |
| Virus | Halorubrum pleomorphic virus 6 | 11 | 2 | DNA | NA |
| Virus | Halovirus HF1 | 102 | 1 | DNA | NA |
| Virus | Hamster polyomavirus | 6 | 1 | DNA | 3 |
| Virus | Hantaan virus | 5 | 5 | RNA | NA |
| Virus | Hantavirus Z10 | 3 | 5 | RNA | NA |
| Virus | Hardenbergia mosaic virus | 12 | 4 | RNA | 1 |
| Virus | Hardenbergia virus A | 2 | 4 | RNA | 1 |
| Virus | Helicobacter phage phiHP33 | 27 | 1 | DNA | 4 |
| Virus | Helicobasidium mompa endornavirus 1 | 1 | 3 | RNA | NA |
| Virus | Helicobasidium mompa No.17 dsRNA virus | 3 | 3 | RNA | NA |
| Virus | Helicoverpa armigera densovirus | 3 | 2 | DNA | 3 |
| Virus | Helicoverpa armigera granulovirus | 179 | 1 | DNA | NA |
| Virus | Helicoverpa armigera multiple nucleopolyhedrovirus | 162 | 1 | DNA | NA |
| Virus | Helicoverpa armigera NPV | 137 | 1 | DNA | NA |
| Virus | Helicoverpa armigera NPV NNg1 | 143 | 1 | DNA | NA |
| Virus | Helicoverpa armigera nucleopolyhedrovirus G4 | 135 | 1 | DNA | NA |
| Virus | Helicoverpa armigera stunt virus | 3 | 4 | RNA | 1 |
| Virus | Helicoverpa zea nudivirus 2 | 113 | 1 | DNA | NA |
| Virus | Helicoverpa zea SNPV | 139 | 1 | DNA | NA |

| Type | Name | Number of genes | Baltimore class | Nucleic Acid | Monophyletic group |
|---|---|---|---|---|---|
| Virus | Heliothis armigera cypovirus 5 | 4 | 3 | RNA | NA |
| Virus | Heliothis virescens ascovirus 3e | 180 | 1 | DNA | 5 |
| Virus | Helleborus net necrosis virus | 6 | 4 | RNA | 1 |
| Virus | Helminthosporium victoriae 145S virus | 4 | 3 | RNA | NA |
| Virus | Helminthosporium victoriae virus 190S | 2 | 3 | RNA | NA |
| Virus | Hendra virus | 8 | 5 | RNA | NA |
| Virus | Hepatitis A virus | 14 | 4 | RNA | 1 |
| Virus | Hepatitis B virus | 7 | 7 | DNA | 2 |
| Virus | Hepatitis C virus | 12 | 4 | RNA | 1 |
| Virus | Hepatitis C virus genotype 2 | 1 | 4 | RNA | 1 |
| Virus | Hepatitis C virus genotype 3 | 10 | 4 | RNA | 1 |
| Virus | Hepatitis C virus genotype 4 | 1 | 4 | RNA | 1 |
| Virus | Hepatitis C virus genotype 5 | 1 | 4 | RNA | 1 |
| Virus | Hepatitis C virus genotype 6 | 1 | 4 | RNA | 1 |
| Virus | Hepatitis delta virus | 2 | 5 | RNA | NA |
| Virus | Hepatitis E virus | 3 | 4 | RNA | 1 |
| Virus | Hepatitis GB virus B | 11 | 4 | RNA | 1 |
| Virus | Heron hepatitis B virus | 4 | 7 | DNA | 2 |
| Virus | Heterocapsa circularisquama RNA virus | 2 | 4 | RNA | 1 |
| Virus | Heterosigma akashiwo RNA virus | 1 | 4 | RNA | 1 |
| Virus | Hibiscus chlorotic ringspot virus | 7 | 4 | RNA | 1 |
| Virus | Hibiscus green spot virus | 8 | 4 | RNA | NA |
| Virus | Hibiscus latent Singapore virus | 4 | 4 | RNA | 1 |
| Virus | Highlands J virus | 13 | 4 | RNA | 1 |
| Virus | Himetobi P virus | 2 | 4 | RNA | 1 |
| Virus | Hippeastrum latent virus | 6 | 4 | RNA | 1 |
| Virus | Hippeastrum mosaic virus | 11 | 4 | RNA | 1 |
| Virus | Hirame rhabdovirus | 6 | 5 | RNA | NA |
| Virus | His1 virus | 35 | 1 | DNA | NA |
| Virus | His2 virus | 35 | 1 | DNA | NA |
| Virus | HMO Astrovirus A | 3 | 4 | RNA | 1 |
| Virus | Hollyhock leaf crumple virus | 6 | 2 | DNA | 3 |
| Virus | Hollyhock yellow vein mosaic virus | 6 | 2 | DNA | 3 |
| Virus | Homalodisca coagulata virus-1 | 2 | 4 | RNA | 1 |
| Virus | Homalodisca vitripennis reovirus | 13 | 3 | RNA | NA |
| Virus | Honeysuckle ringspot virus | 6 | 4 | RNA | 1 |
| Virus | Honeysuckle yellow vein betasatellite | 1 | 2 | DNA | 3 |
| Virus | Honeysuckle yellow vein mosaic disease associated satellite DNA beta | 1 | 2 | DNA | NA |
| Virus | Honeysuckle yellow vein mosaic virus | 6 | 2 | DNA | 3 |
| Virus | Honeysuckle yellow vein virus | 6 | 2 | DNA | 3 |
| Virus | Hop latent virus | 6 | 4 | RNA | 1 |
| Virus | Hop mosaic virus | 6 | 4 | RNA | 1 |
| Virus | Hordeum mosaic virus | 12 | 4 | RNA | 1 |
| Virus | Horsegram yellow mosaic virus | 8 | 2 | DNA | 3 |
| Virus | Horseradish curly top virus | 6 | 2 | DNA | 3 |
| Virus | Horseradish latent virus | 8 | 7 | DNA | 2 |
| Virus | Hosta virus X | 5 | 4 | RNA | 1 |
| Virus | Human adenovirus 1 | 36 | 1 | DNA | NA |
| Virus | Human adenovirus 2 | 36 | 1 | DNA | NA |
| Virus | Human adenovirus 35 | 37 | 1 | DNA | NA |
| Virus | Human adenovirus 5 | 36 | 1 | DNA | NA |
| Virus | Human adenovirus 54 | 36 | 1 | DNA | NA |
| Virus | Human adenovirus 7 | 37 | 1 | DNA | NA |
| Virus | Human adenovirus A | 71 | 1 | DNA | NA |
| Virus | Human adenovirus B | 77 | 1 | DNA | NA |
| Virus | Human adenovirus C | 38 | 1 | DNA | NA |
| Virus | Human adenovirus D | 57 | 1 | DNA | NA |
| Virus | Human adenovirus E | 38 | 1 | DNA | NA |
| Virus | Human adenovirus F | 34 | 1 | DNA | NA |
| Virus | Human bocavirus | 4 | 2 | DNA | 3 |
| Virus | Human bocavirus 2 | 4 | 2 | DNA | 3 |
| Virus | Human bocavirus 3 | 4 | 2 | DNA | 3 |
| Virus | Human bocavirus 4 | 5 | 2 | DNA | 3 |
| Virus | Human coronavirus 229E | 8 | 4 | RNA | NA |
| Virus | Human coronavirus HKU1 | 23 | 4 | RNA | NA |
| Virus | Human coronavirus NL63 | 6 | 4 | RNA | NA |
| Virus | Human coronavirus OC43 | 9 | 4 | RNA | NA |
| Virus | Human cosavirus A | 12 | 4 | RNA | 1 |
| Virus | Human cosavirus B | 12 | 4 | RNA | 1 |
| Virus | Human cosavirus D | 12 | 4 | RNA | 1 |
| Virus | Human cosavirus E | 12 | 4 | RNA | 1 |
| Virus | Human enteric coronavirus strain 4408 | 12 | 4 | RNA | NA |
| Virus | Human enterovirus 100 | 12 | 4 | RNA | 1 |
| Virus | Human enterovirus 107 | 12 | 4 | RNA | 1 |
| Virus | Human enterovirus 98 | 12 | 4 | RNA | 1 |
| Virus | Human enterovirus A | 12 | 4 | RNA | 1 |
| Virus | Human enterovirus B | 12 | 4 | RNA | 1 |
| Virus | Human enterovirus C | 12 | 4 | RNA | 1 |
| Virus | Human enterovirus C109 | 12 | 4 | RNA | 1 |
| Virus | Human enterovirus D | 17 | 4 | RNA | 1 |

| Type | Name | Number of genes | Baltimore class | Nucleic Acid | Monophyletic group |
|---|---|---|---|---|---|
| Virus | Human erythrovirus V9 | 6 | 2 | DNA | 3 |
| Virus | Human herpesvirus 1 | 77 | 1 | DNA | NA |
| Virus | Human herpesvirus 2 | 77 | 1 | DNA | NA |
| Virus | Human herpesvirus 3 | 73 | 1 | DNA | NA |
| Virus | Human herpesvirus 4 | 94 | 1 | DNA | NA |
| Virus | Human herpesvirus 4 type 2 | 80 | 1 | DNA | NA |
| Virus | Human herpesvirus 5 | 168 | 1 | DNA | NA |
| Virus | Human herpesvirus 6 | 192 | 1 | DNA | NA |
| Virus | Human herpesvirus 7 | 86 | 1 | DNA | NA |
| Virus | Human herpesvirus 8 | 86 | 1 | DNA | NA |
| Virus | Human immunodeficiency virus 1 | 27 | 6 | RNA | 2 |
| Virus | Human immunodeficiency virus 2 | 9 | 6 | RNA | 2 |
| Virus | Human klassevirus 1 | 1 | 4 | RNA | 1 |
| Virus | Human metapneumovirus | 9 | 5 | RNA | NA |
| Virus | Human papillomavirus | 6 | 1 | DNA | 3 |
| Virus | Human papillomavirus - 1 | 7 | 1 | DNA | 3 |
| Virus | Human papillomavirus - 18 | 8 | 1 | DNA | 3 |
| Virus | Human papillomavirus - 2 | 7 | 1 | DNA | 3 |
| Virus | Human papillomavirus 109 | 7 | 1 | DNA | 3 |
| Virus | Human papillomavirus 112 | 7 | 1 | DNA | 3 |
| Virus | Human papillomavirus 116 | 7 | 1 | DNA | 3 |
| Virus | Human papillomavirus 121 | 7 | 1 | DNA | 3 |
| Virus | Human papillomavirus type 10 | 7 | 1 | DNA | 3 |
| Virus | Human papillomavirus type 101 | 6 | 1 | DNA | 3 |
| Virus | Human papillomavirus type 103 | 6 | 1 | DNA | 3 |
| Virus | Human papillomavirus type 108 | 5 | 1 | DNA | 3 |
| Virus | Human papillomavirus type 126 | 7 | 1 | DNA | 3 |
| Virus | Human papillomavirus type 128 | 7 | 1 | DNA | 3 |
| Virus | Human papillomavirus type 129 | 7 | 1 | DNA | 3 |
| Virus | Human papillomavirus type 131 | 7 | 1 | DNA | 3 |
| Virus | Human papillomavirus type 132 | 7 | 1 | DNA | 3 |
| Virus | Human papillomavirus type 134 | 7 | 1 | DNA | 3 |
| Virus | Human papillomavirus type 135 | 7 | 1 | DNA | 3 |
| Virus | Human papillomavirus type 136 | 7 | 1 | DNA | 3 |
| Virus | Human papillomavirus type 137 | 7 | 1 | DNA | 3 |
| Virus | Human papillomavirus type 140 | 7 | 1 | DNA | 3 |
| Virus | Human papillomavirus type 144 | 7 | 1 | DNA | 3 |
| Virus | Human papillomavirus type 16 | 8 | 1 | DNA | 3 |
| Virus | Human papillomavirus type 26 | 6 | 1 | DNA | 3 |
| Virus | Human papillomavirus type 32 | 6 | 1 | DNA | 3 |
| Virus | Human papillomavirus type 34 | 6 | 1 | DNA | 3 |
| Virus | Human papillomavirus type 4 | 7 | 1 | DNA | 3 |
| Virus | Human papillomavirus type 41 | 11 | 1 | DNA | 3 |
| Virus | Human papillomavirus type 48 | 7 | 1 | DNA | 3 |
| Virus | Human papillomavirus type 49 | 6 | 1 | DNA | 3 |
| Virus | Human papillomavirus type 5 | 8 | 1 | DNA | 3 |
| Virus | Human papillomavirus type 50 | 7 | 1 | DNA | 3 |
| Virus | Human papillomavirus type 53 | 7 | 1 | DNA | 3 |
| Virus | Human papillomavirus type 60 | 7 | 1 | DNA | 3 |
| Virus | Human papillomavirus type 63 | 7 | 1 | DNA | 3 |
| Virus | Human papillomavirus type 6b | 9 | 1 | DNA | 3 |
| Virus | Human papillomavirus type 7 | 6 | 1 | DNA | 3 |
| Virus | Human papillomavirus type 88 | 7 | 1 | DNA | 3 |
| Virus | Human papillomavirus type 9 | 7 | 1 | DNA | 3 |
| Virus | Human papillomavirus type 90 | 7 | 1 | DNA | 3 |
| Virus | Human papillomavirus type 92 | 7 | 1 | DNA | 3 |
| Virus | Human papillomavirus type 96 | 7 | 1 | DNA | 3 |
| Virus | Human parainfluenza virus 1 | 10 | 5 | RNA | NA |
| Virus | Human parainfluenza virus 2 | 7 | 5 | RNA | NA |
| Virus | Human parainfluenza virus 3 | 8 | 5 | RNA | NA |
| Virus | Human parechovirus | 11 | 4 | RNA | 1 |
| Virus | Human parvovirus 4 | 2 | 2 | DNA | 3 |
| Virus | Human parvovirus B19 | 6 | 2 | DNA | 3 |
| Virus | Human picobirnavirus | 3 | 1 | DNA | 1 |
| Virus | Human polyomavirus 9 | 5 | 1 | DNA | 3 |
| Virus | Human respiratory syncytial virus | 11 | 5 | RNA | NA |
| Virus | Human rhinovirus A | 12 | 4 | RNA | 1 |
| Virus | Human rhinovirus B14 | 12 | 4 | RNA | 1 |
| Virus | Human rhinovirus C | 12 | 4 | RNA | 1 |
| Virus | Human T-lymphotropic virus 1 | 11 | 6 | RNA | 2 |
| Virus | Human T-lymphotropic virus 2 | 10 | 6 | RNA | 2 |
| Virus | Human T-lymphotropic virus 4 | 6 | 6 | RNA | 2 |
| Virus | Human TMEV-like cardiovirus | 13 | 4 | RNA | 1 |
| Virus | Humulus japonicus latent virus | 4 | 4 | RNA | 1 |
| Virus | Hydrangea chlorotic mottle virus | 6 | 4 | RNA | 1 |
| Virus | Hydrangea ringspot virus | 6 | 4 | RNA | 1 |
| Virus | Hyperthermophilic Archaeal Virus 1 | 40 | 1 | DNA | NA |
| Virus | Hyperthermophilic Archaeal Virus 2 | 15 | NA | NA | NA |
| Virus | Hyphantria cunea nucleopolyhedrovirus | 148 | 1 | DNA | NA |
| Virus | Hyposoter fugitivus ichnovirus | 143 | 1 | DNA | NA |

| Type | Name | Number of genes | Baltimore class | Nucleic Acid | Monophyletic group |
|---|---|---|---|---|---|
| Virus | Ictalurid herpesvirus 1 | 90 | 1 | DNA | NA |
| Virus | Ikoma lyssavirus | 5 | 5 | RNA | NA |
| Virus | Ilheus virus | 1 | 4 | RNA | 1 |
| Virus | Impatiens necrotic spot virus | 5 | 5 | RNA | NA |
| Virus | Imperata yellow mottle virus | 4 | 4 | RNA | 1 |
| Virus | Indian cassava mosaic virus | 10 | 2 | DNA | 3 |
| Virus | Indian citrus ringspot virus | 6 | 4 | RNA | 1 |
| Virus | Indian peanut clump virus | 8 | 4 | RNA | 1 |
| Virus | Infectious bronchitis virus | 24 | 4 | RNA | NA |
| Virus | Infectious bursal disease virus | 3 | 3 | RNA | NA |
| Virus | Infectious flacherie virus | 1 | 4 | RNA | 1 |
| Virus | Infectious hematopoietic necrosis virus | 6 | 5 | RNA | NA |
| Virus | Infectious hypodermal and hematopoietic necrosis virus | 3 | 2 | DNA | 3 |
| Virus | Infectious pancreatic necrosis virus | 3 | 3 | RNA | NA |
| Virus | Infectious salmon anemia virus | 10 | 5 | RNA | NA |
| Virus | Infectious spleen and kidney necrosis virus | 125 | 1 | DNA | 5 |
| Virus | Influenza A virus (A/Goose/Guangdong/1/96(H5N1)) | 14 | 5 | RNA | NA |
| Virus | Influenza A virus (A/Hong Kong/1073/99(H9N2)) | 14 | 5 | RNA | NA |
| Virus | Influenza A virus (A/Korea/426/1968(H2N2)) | 3 | 5 | RNA | NA |
| Virus | Influenza A virus (A/Korea/426/68(H2N2)) | 11 | 5 | RNA | NA |
| Virus | Influenza A virus (A/New York/392/2004(H3N2)) | 14 | 5 | RNA | NA |
| Virus | Influenza A virus (A/Puerto Rico/8/1934(H1N1)) | 2 | 5 | RNA | NA |
| Virus | Influenza A virus (A/Puerto Rico/8/34(H1N1)) | 12 | 5 | RNA | NA |
| Virus | Influenza B virus | 11 | 5 | RNA | NA |
| Virus | Influenza C virus (C/Ann Arbor/1/50) | 9 | 5 | RNA | NA |
| Virus | Invertebrate iridescent virus 3 | 126 | 1 | DNA | 5 |
| Virus | Invertebrate iridescent virus 6 | 468 | 1 | DNA | 5 |
| Virus | Iodobacteriophage phiPLPE | 84 | 1 | DNA | 4 |
| Virus | Iotapapillomavirus 1 | 6 | 1 | DNA | 3 |
| Virus | Ipomoea yellow vein virus | 6 | 2 | DNA | 3 |
| Virus | Ippy virus | 4 | 5 | RNA | NA |
| Virus | Iranian johnsongrass mosaic virus | 1 | 4 | RNA | 1 |
| Virus | Israeli acute paralysis virus | 3 | 4 | RNA | 1 |
| Virus | J-virus | 11 | 5 | RNA | NA |
| Virus | Jaagsiekte sheep retrovirus | 5 | 6 | RNA | 2 |
| Virus | Japanese eel endothelial cells-infecting virus | 1 | 1 | DNA | NA |
| Virus | Japanese encephalitis virus | 16 | 4 | RNA | 1 |
| Virus | Japanese holly fern mottle virus | 5 | 3 | RNA | NA |
| Virus | Japanese iris necrotic ring virus | 6 | 4 | RNA | 1 |
| Virus | Japanese yam mosaic virus | 12 | 4 | RNA | 1 |
| Virus | Jatropha leaf curl virus | 6 | 2 | DNA | 3 |
| Virus | Jatropha mosaic Nigerian virus | 6 | 2 | DNA | 3 |
| Virus | Jatropha yellow mosaic India virus | 6 | 2 | DNA | 3 |
| Virus | JC polyomavirus | 6 | 1 | DNA | 3 |
| Virus | Johnsongrass chlorotic stripe mosaic virus | 5 | 4 | RNA | 1 |
| Virus | Johnsongrass mosaic virus | 12 | 4 | RNA | 1 |
| Virus | Junin virus | 6 | 5 | RNA | NA |
| Virus | Junonia coenia densovirus | 4 | 2 | DNA | 3 |
| Virus | Kadipiro virus | 12 | 3 | RNA | NA |
| Virus | Kakugo virus | 1 | 4 | RNA | 1 |
| Virus | Kalanchoe latent virus | 7 | 4 | RNA | 1 |
| Virus | Kalanchoe top-spotting virus | 3 | 7 | DNA | 2 |
| Virus | Kamiti River virus | 14 | 4 | RNA | 1 |
| Virus | Kappapapillomavirus 1 | 9 | 1 | DNA | 3 |
| Virus | Kappapapillomavirus 2 | 10 | 1 | DNA | 3 |
| Virus | Karshi virus | 1 | 4 | RNA | 1 |
| Virus | Kashmir bee virus | 7 | 4 | RNA | 1 |
| Virus | Kedougou virus | 1 | 4 | RNA | 1 |
| Virus | Kelp fly virus | 1 | 4 | RNA | NA |
| Virus | Kennedya yellow mosaic virus | 3 | 4 | RNA | 1 |
| Virus | Keunjorong mosaic virus | 12 | 4 | RNA | 1 |
| Virus | KI polyomavirus | 5 | 1 | DNA | 3 |
| Virus | Klebsiella phage K11 | 51 | 1 | DNA | 4 |
| Virus | Klebsiella phage KP15 | 258 | 1 | DNA | 4 |
| Virus | Klebsiella phage KP32 | 44 | 1 | DNA | 4 |
| Virus | Klebsiella phage KP34 | 57 | 1 | DNA | 4 |
| Virus | Klebsiella phage phiKO2 | 64 | 1 | DNA | 4 |
| Virus | Kluyvera phage Kvp1 | 47 | 1 | DNA | 4 |
| Virus | Kokobera virus | 1 | 4 | RNA | 1 |
| Virus | Konjac mosaic virus | 12 | 4 | RNA | 1 |
| Virus | Kotonkan virus | 11 | 5 | RNA | NA |
| Virus | Kudzu mosaic virus | 8 | 2 | DNA | 3 |
| Virus | Kyuri green mottle mosaic virus | 4 | 4 | RNA | 1 |
| Virus | La Crosse virus | 4 | 5 | RNA | NA |
| Virus | Lactate dehydrogenase-elevating virus | 12 | 4 | RNA | NA |
| Virus | Lactobacillus johnsonii prophage Lj771 | 56 | NA | NA | NA |
| Virus | Lactobacillus phage A2 | 62 | 1 | DNA | 4 |
| Virus | Lactobacillus phage KC5a | 61 | 1 | DNA | 4 |
| Virus | Lactobacillus phage Lb338-1 | 199 | 1 | DNA | 4 |
| Virus | Lactobacillus phage Lc-Nu | 51 | 1 | DNA | 4 |

| Type | Name | Number of genes | Baltimore class | Nucleic Acid | Monophyletic group |
|---|---|---|---|---|---|
| Virus | Lactobacillus phage LL-H | 51 | 1 | DNA | 4 |
| Virus | Lactobacillus phage LP65 | 165 | 1 | DNA | 4 |
| Virus | Lactobacillus phage Lrm1 | 54 | 1 | DNA | 4 |
| Virus | Lactobacillus phage Lv-1 | 47 | 1 | DNA | 4 |
| Virus | Lactobacillus phage phiadh | 63 | 1 | DNA | 4 |
| Virus | Lactobacillus phage phiAT3 | 55 | 1 | DNA | 4 |
| Virus | Lactobacillus phage phig1e | 60 | 1 | DNA | 4 |
| Virus | Lactobacillus phage phiJL-1 | 46 | 1 | DNA | 4 |
| Virus | Lactobacillus prophage Lj928 | 50 | 1 | DNA | 4 |
| Virus | Lactobacillus prophage Lj965 | 46 | 1 | DNA | 4 |
| Virus | Lactococcus phage 1706 | 76 | 1 | DNA | 4 |
| Virus | Lactococcus phage 712 | 55 | 1 | DNA | 4 |
| Virus | Lactococcus phage 949 | 154 | 1 | DNA | 4 |
| Virus | Lactococcus phage asccphi28 | 28 | 1 | DNA | 4 |
| Virus | Lactococcus phage bIBB29 | 54 | 1 | DNA | 4 |
| Virus | Lactococcus phage bIL285 | 62 | 1 | DNA | 4 |
| Virus | Lactococcus phage bIL286 | 61 | 1 | DNA | 4 |
| Virus | Lactococcus phage bIL309 | 56 | 1 | DNA | 4 |
| Virus | Lactococcus phage bIL310 | 29 | 1 | DNA | 4 |
| Virus | Lactococcus phage bIL311 | 22 | 1 | DNA | 4 |
| Virus | Lactococcus phage bIL312 | 27 | 1 | DNA | 4 |
| Virus | Lactococcus phage bIL67 | 37 | 1 | DNA | 4 |
| Virus | Lactococcus phage BK5-T | 63 | 1 | DNA | 4 |
| Virus | Lactococcus phage c2 | 39 | 1 | DNA | 4 |
| Virus | Lactococcus phage jj50 | 49 | 1 | DNA | 4 |
| Virus | Lactococcus phage KSY1 | 130 | 1 | DNA | 4 |
| Virus | Lactococcus phage P087 | 88 | 1 | DNA | 4 |
| Virus | Lactococcus phage P335 sensu lato | 49 | 1 | DNA | 4 |
| Virus | Lactococcus phage phiLC3 | 51 | 1 | DNA | 4 |
| Virus | Lactococcus phage Q54 | 47 | 1 | DNA | 4 |
| Virus | Lactococcus phage r1t | 50 | 1 | DNA | 4 |
| Virus | Lactococcus phage sk1 | 56 | 1 | DNA | 4 |
| Virus | Lactococcus phage TP901-1 | 56 | 1 | DNA | 4 |
| Virus | Lactococcus phage Tuc2009 | 56 | 1 | DNA | 4 |
| Virus | Lactococcus phage ul36 | 61 | 1 | DNA | 4 |
| Virus | Lambdapapillomavirus 2 | 7 | 1 | DNA | 3 |
| Virus | Lambdapapillomavirus 3 | 7 | 1 | DNA | 3 |
| Virus | Lamium leaf distortion virus | 6 | 7 | DNA | 2 |
| Virus | Langat virus | 11 | 4 | RNA | 1 |
| Virus | Lassa virus | 4 | 5 | RNA | NA |
| Virus | Latino virus | 4 | 5 | RNA | NA |
| Virus | Lausannevirus | 444 | 1 | DNA | 5 |
| Virus | Leek white stripe virus | 5 | 4 | RNA | 1 |
| Virus | Leek yellow stripe virus | 12 | 4 | RNA | 1 |
| Virus | Leishmania RNA virus 1 - 1 | 3 | 3 | RNA | NA |
| Virus | Leishmania RNA virus 1 - 4 | 2 | 3 | RNA | NA |
| Virus | Leishmania RNA virus 2 - 1 | 3 | 3 | RNA | NA |
| Virus | Lettuce big-vein associated virus | 6 | 4 | RNA | 1 |
| Virus | Lettuce chlorosis virus | 14 | 4 | RNA | 1 |
| Virus | Lettuce infectious yellows virus | 9 | 4 | RNA | 1 |
| Virus | Lettuce mosaic virus | 12 | 4 | RNA | 1 |
| Virus | Lettuce necrotic stunt virus | 6 | 4 | RNA | 1 |
| Virus | Lettuce necrotic yellows virus | 6 | 5 | RNA | NA |
| Virus | Lettuce ring necrosis virus | 5 | 5 | RNA | NA |
| Virus | Lettuce virus X | 5 | 4 | RNA | 1 |
| Virus | Lettuce yellow mottle virus | 6 | 5 | RNA | NA |
| Virus | Leucania separata nuclear polyhedrosis virus | 169 | 1 | DNA | NA |
| Virus | Leucas zeylanica yellow vein virus satellite DNA beta | 1 | 2 | DNA | 3 |
| Virus | Leuconostoc phage Lmd1 | 40 | 1 | DNA | 4 |
| Virus | Liao ning virus | 12 | 3 | RNA | NA |
| Virus | Ligustrum necrotic ringspot virus | 6 | 4 | RNA | 1 |
| Virus | Lily mottle virus | 12 | 4 | RNA | 1 |
| Virus | Lily symptomless virus | 6 | 4 | RNA | 1 |
| Virus | Lily virus X | 5 | 4 | RNA | 1 |
| Virus | Lindernia anagallis yellow vein virus | 6 | 2 | DNA | 3 |
| Virus | Lindernia anagallis yellow vein virus satellite DNA beta | 1 | 2 | DNA | 3 |
| Virus | Lisianthus necrosis virus | 5 | 4 | RNA | 1 |
| Virus | Listeria phage 2389 | 59 | 1 | DNA | 4 |
| Virus | Listeria phage A006 | 64 | 1 | DNA | 4 |
| Virus | Listeria phage A118 | 72 | 1 | DNA | 4 |
| Virus | Listeria phage A500 | 63 | 1 | DNA | 4 |
| Virus | Listeria phage A511 | 201 | 1 | DNA | 4 |
| Virus | Listeria phage B025 | 66 | 1 | DNA | 4 |
| Virus | Listeria phage B054 | 80 | 1 | DNA | 4 |
| Virus | Listeria phage P35 | 56 | 1 | DNA | 4 |
| Virus | Listeria phage P40 | 62 | 1 | DNA | 4 |
| Virus | Listeria phage P70 | 119 | 1 | DNA | 4 |
| Virus | Listonella phage phiHSIC | 47 | 1 | DNA | 4 |
| Virus | Little cherry virus 1 | 10 | 4 | RNA | 1 |
| Virus | Little cherry virus 2 | 10 | 4 | RNA | 1 |

| Type | Name | Number of genes | Baltimore class | Nucleic Acid | Monophyletic group |
|---|---|---|---|---|---|
| Virus | Ljungan virus | 12 | 4 | RNA | 1 |
| Virus | Lloviu virus | 9 | 5 | RNA | NA |
| Virus | Lolium latent virus | 6 | 4 | RNA | 1 |
| Virus | Louping ill virus | 14 | 4 | RNA | 1 |
| Virus | Lucerne transient streak virus | 8 | 4 | RNA | 1 |
| Virus | Lucky bamboo bacilliform virus | 7 | 7 | DNA | 2 |
| Virus | Ludwigia yellow vein virus | 6 | 2 | DNA | 3 |
| Virus | Ludwigia yellow vein virus-associated DNA beta | 1 | 2 | DNA | NA |
| Virus | Luffa begomovirus associated DNA beta | 1 | 2 | DNA | 3 |
| Virus | Luffa puckering and leaf distortion-associated DNA beta | 1 | 2 | DNA | 3 |
| Virus | Luffa yellow mosaic virus | 8 | 2 | DNA | 3 |
| Virus | LuIII virus | 2 | 2 | DNA | 3 |
| Virus | Lujo virus | 4 | 5 | RNA | NA |
| Virus | Lumpy skin disease virus NI-2490 | 156 | 1 | DNA | 5 |
| Virus | Luna virus | 4 | 5 | RNA | NA |
| Virus | Lunk virus NKS-1 | 4 | 5 | RNA | NA |
| Virus | Lupine mosaic virus | 12 | 4 | RNA | 1 |
| Virus | Lymantria dispar MNPV | 164 | 1 | DNA | NA |
| Virus | Lymantria xylina MNPV | 157 | 1 | DNA | NA |
| Virus | Lymphocystis disease virus - isolate China | 239 | 1 | DNA | 5 |
| Virus | Lymphocystis disease virus 1 | 110 | 1 | DNA | 5 |
| Virus | Lymphocytic choriomeningitis virus | 4 | 5 | RNA | NA |
| Virus | Macaca fascicularis papillomavirus 2 | 7 | 1 | DNA | 3 |
| Virus | Macacine herpesvirus 1 | 75 | 1 | DNA | NA |
| Virus | Macacine herpesvirus 3 | 223 | 1 | DNA | NA |
| Virus | Macacine herpesvirus 4 | 80 | 1 | DNA | NA |
| Virus | Macacine herpesvirus 5 | 89 | 1 | DNA | NA |
| Virus | Macaque simian foamy virus | 5 | 6 | RNA | 2 |
| Virus | Machupo virus | 4 | 5 | RNA | NA |
| Virus | Macrobrachium rosenbergii nodavirus | 3 | 4 | RNA | 1 |
| Virus | Macrobrachium rosenbergii Taihu virus | 2 | 4 | RNA | NA |
| Virus | Macroptilium golden mosaic virus | 6 | 2 | DNA | 3 |
| Virus | Macroptilium mosaic Puerto Rico virus | 7 | 2 | DNA | 3 |
| Virus | Macroptilium yellow mosaic Florida virus | 7 | 2 | DNA | 3 |
| Virus | Macroptilium yellow mosaic virus | 7 | 2 | DNA | 3 |
| Virus | Macroptilium yellow net virus | 7 | 2 | DNA | 3 |
| Virus | Macroptilium yellow spot virus | 5 | 2 | DNA | 3 |
| Virus | Macroptilium yellow vein virus | 5 | 2 | DNA | 3 |
| Virus | Magnaporthe oryzae chrysovirus 1 | 4 | 3 | RNA | NA |
| Virus | Magnaporthe oryzae virus 1 | 2 | 3 | RNA | NA |
| Virus | Magnaporthe oryzae virus 2 | 2 | 3 | RNA | NA |
| Virus | Magpie-robin coronavirus HKU18 | 9 | 4 | RNA | NA |
| Virus | Maize chlorotic dwarf virus | 8 | 4 | RNA | 1 |
| Virus | Maize chlorotic mottle virus | 6 | 4 | RNA | 1 |
| Virus | Maize dwarf mosaic virus | 11 | 4 | RNA | 1 |
| Virus | Maize fine streak virus | 7 | 5 | RNA | NA |
| Virus | Maize Iranian mosaic virus | 6 | 5 | RNA | NA |
| Virus | Maize mosaic virus | 6 | 5 | RNA | NA |
| Virus | Maize necrotic streak virus | 5 | 4 | RNA | 1 |
| Virus | maize rayado fino virus | 3 | 4 | RNA | 1 |
| Virus | Maize rayado fino virus | 2 | 4 | RNA | 1 |
| Virus | Maize streak Reunion virus | 4 | 2 | DNA | 3 |
| Virus | Maize streak virus | 5 | 2 | DNA | 3 |
| Virus | Maize white line mosaic virus | 5 | 4 | RNA | 1 |
| Virus | Mal de Rio Cuarto virus | 12 | 3 | RNA | NA |
| Virus | Malachra yellow vein mosaic virus-associated satellite DNA beta | 1 | 2 | DNA | 3 |
| Virus | Malva mosaic virus | 5 | 4 | RNA | 1 |
| Virus | Malvastrum leaf curl betasatellite | 1 | 2 | DNA | 3 |
| Virus | Malvastrum leaf curl Guangdong virus | 6 | 2 | DNA | 3 |
| Virus | Malvastrum leaf curl virus | 6 | 2 | DNA | 3 |
| Virus | Malvastrum yellow mosaic alphasatellite | 1 | NA | NA | NA |
| Virus | Malvastrum yellow mosaic Cameroon alphasatellite | 1 | NA | NA | NA |
| Virus | Malvastrum yellow mosaic virus | 6 | 2 | DNA | 3 |
| Virus | Malvastrum yellow mosaic virus satellite DNA beta | 1 | 2 | DNA | 3 |
| Virus | Malvastrum yellow vein Baoshan virus | 6 | 2 | DNA | 3 |
| Virus | Malvastrum yellow vein betasatellite | 1 | 2 | DNA | 3 |
| Virus | Malvastrum yellow vein Changa Manga virus | 6 | 2 | DNA | 3 |
| Virus | Malvastrum yellow vein virus | 6 | 2 | DNA | 3 |
| Virus | Malvastrum yellow vein Yunnan virus | 6 | 2 | DNA | 3 |
| Virus | Malvastrum yellow vein Yunnan virus satellite DNA beta | 1 | 2 | DNA | 3 |
| Virus | Mama_proteome_aa_ | 1023 | 1 | DNA | 5 |
| Virus | Mamastrovirus 1 | 10 | 4 | RNA | 1 |
| Virus | Mamastrovirus 10 | 3 | 4 | RNA | 1 |
| Virus | Mamastrovirus 13 | 3 | 4 | RNA | 1 |
| Virus | Mamestra configurata NPV-A | 169 | 1 | DNA | NA |
| Virus | Mamestra configurata NPV-B | 168 | 1 | DNA | NA |
| Virus | Mammalian orthoreovirus 3 | 24 | 3 | RNA | NA |
| Virus | Mannheimia phage phiMHaA1 | 49 | 1 | DNA | 4 |
| Virus | Mapuera virus | 9 | 5 | RNA | NA |
| Virus | Maracuja mosaic virus | 4 | 4 | RNA | 1 |

| Type | Name | Number of genes | Baltimore class | Nucleic Acid | Monophyletic group |
|---|---|---|---|---|---|
| Virus | Marburg marburgvirus | 7 | 5 | RNA | NA |
| Virus | Marine birnavirus | 3 | 3 | RNA | NA |
| Virus | Marine RNA virus JP-A | 2 | 4 | RNA | 1 |
| Virus | Marine RNA virus JP-B | 2 | 4 | RNA | NA |
| Virus | Marine RNA virus SOG | 3 | 4 | RNA | NA |
| Virus | Marinomonas phage P12026 | 54 | 1 | DNA | NA |
| Virus | Mars_Cplete_proteome_aa | 457 | 1 | DNA | 5 |
| Virus | Marseillevirus | 428 | 1 | DNA | 5 |
| Virus | Maruca vitrata MNPV | 126 | 1 | DNA | NA |
| Virus | Mason-Pfizer monkey virus | 14 | 6 | RNA | 2 |
| Virus | Mastomys coucha papillomavirus 2 | 6 | 1 | DNA | 3 |
| Virus | Mavirus | 20 | 1 | DNA | Unconsidered |
| Virus | Mayaro virus | 14 | 4 | RNA | 1 |
| Virus | Measles virus | 8 | 5 | RNA | NA |
| Virus | Megavirus chiliensis | 1120 | 1 | DNA | 5 |
| Virus | Melandrium yellow fleck virus | 4 | 4 | RNA | 1 |
| Virus | Melanoplus sanguinipes entomopoxvirus | 267 | 1 | DNA | 5 |
| Virus | Meleagrid herpesvirus 1 | 79 | 1 | DNA | NA |
| Virus | Melon aphid-borne yellows virus | 6 | 4 | RNA | 1 |
| Virus | Melon chlorotic mosaic virus | 7 | 2 | DNA | 3 |
| Virus | Melon chlorotic mosaic virus-associated alphasatellite | 2 | 2 | DNA | NA |
| Virus | Melon necrotic spot virus | 6 | 4 | RNA | 1 |
| Virus | Melon yellow spot virus | 5 | 5 | RNA | NA |
| Virus | Menangle virus | 7 | 5 | RNA | NA |
| Virus | Merkel cell polyomavirus | 5 | 1 | DNA | 3 |
| Virus | Merremia mosaic Puerto Rico virus | 6 | 2 | DNA | 3 |
| Virus | Merremia mosaic virus | 6 | 2 | DNA | 3 |
| Virus | Mesta yellow vein mosaic Bahraich virus | 7 | 2 | DNA | 3 |
| Virus | Mesta yellow vein mosaic virus | 6 | 2 | DNA | 3 |
| Virus | Mesta yellow vein mosaic virus-associated alphasatellite | 1 | NA | NA | NA |
| Virus | Mesta yellow vein mosaic virus-associated DNA beta | 1 | 2 | DNA | NA |
| Virus | Methanobacterium phage psiM2 | 32 | 1 | DNA | 4 |
| Virus | Methanothermobacter phage psiM100 | 35 | 1 | DNA | 4 |
| Virus | Microbacterium phage Min1 | 77 | 1 | DNA | 4 |
| Virus | Microcystis phage Ma-LMM01 | 184 | 1 | DNA | 4 |
| Virus | Micromonas pusilla reovirus | 11 | 3 | RNA | NA |
| Virus | Micromonas sp. RCC1109 virus MpV1 | 244 | 1 | DNA | 5 |
| Virus | Micromys minutus papillomavirus 1 | 7 | 1 | DNA | 3 |
| Virus | Microplitis demolitor bracovirus | 53 | 1 | DNA | NA |
| Virus | Microviridae phi-CA82 | 10 | 2 | DNA | NA |
| Virus | Midway virus | 6 | 5 | RNA | NA |
| Virus | Mikania micrantha mosaic virus | 9 | 4 | RNA | 1 |
| Virus | Milk vetch dwarf virus | 11 | 2 | DNA | NA |
| Virus | Mimosa yellow leaf curl virus | 6 | 2 | DNA | 3 |
| Virus | Mimosa yellow leaf curl virus satellite DNA beta | 1 | 2 | DNA | 3 |
| Virus | Mimosa yellow leaf curl virus-associated DNA 1 | 1 | 2 | DNA | 3 |
| Virus | Mint virus 1 | 11 | 4 | RNA | 1 |
| Virus | Mint virus X | 5 | 4 | RNA | 1 |
| Virus | Minute virus of mice | 7 | 2 | DNA | 3 |
| Virus | Mirabilis jalapa mottle virus | 6 | 4 | RNA | 1 |
| Virus | Mirabilis mosaic virus | 7 | 7 | DNA | 2 |
| Virus | Mirafiore lettuce virus | 7 | 5 | RNA | NA |
| Virus | Miscanthus streak virus | 5 | 2 | DNA | 3 |
| Virus | Mobala virus | 4 | 5 | RNA | NA |
| Virus | Modoc virus | 14 | 4 | RNA | 1 |
| Virus | Mokola virus | 5 | 5 | RNA | NA |
| Virus | Molluscum contagiosum virus subtype 1 | 163 | 1 | DNA | 5 |
| Virus | Moloney murine leukemia virus | 14 | 6 | RNA | 2 |
| Virus | Moloney murine sarcoma virus | 8 | 6 | RNA | 2 |
| Virus | Monkeypox virus Zaire-96-I-16 | 191 | 1 | DNA | 5 |
| Virus | Montana myotis leukoencephalitis virus | 14 | 4 | RNA | 1 |
| Virus | Monve_proteome | 1150 | 1 | DNA | 5 |
| Virus | Mopeia Lassa reassortant 29 | 4 | 5 | RNA | NA |
| Virus | Mopeia virus AN20410 | 4 | 5 | RNA | NA |
| Virus | Morelia spilota papillomavirus 1 | 7 | 1 | DNA | 3 |
| Virus | Morganella phage MmP1 | 49 | 1 | DNA | 4 |
| Virus | Moroccan watermelon mosaic virus | 12 | 4 | RNA | 1 |
| Virus | Morogoro virus | 4 | 5 | RNA | NA |
| Virus | Mosquito densovirus BR/07 | 3 | 2 | DNA | 3 |
| Virus | Mossman virus | 8 | 5 | RNA | NA |
| Virus | Moumou_proteome_aa | 930 | 1 | DNA | 5 |
| Virus | Mouse astrovirus M-52/USA/2008 | 2 | 4 | RNA | 1 |
| Virus | Mouse kobuvirus M-5/USA/2010 | 1 | 4 | RNA | 1 |
| Virus | Mouse mammary tumor virus | 14 | 6 | RNA | 2 |
| Virus | Mouse parvovirus 1 | 2 | 2 | DNA | 3 |
| Virus | Mouse parvovirus 2 | 6 | 2 | DNA | 3 |
| Virus | Mouse parvovirus 3 | 6 | 2 | DNA | 3 |
| Virus | Mouse parvovirus 4a | 6 | 2 | DNA | 3 |
| Virus | Mouse parvovirus 5a | 6 | 2 | DNA | 3 |
| Virus | Mud crab dicistrovirus | 2 | 4 | RNA | 1 |

| Type | Name | Number of genes | Baltimore class | Nucleic Acid | Monophyletic group |
|---|---|---|---|---|---|
| Virus | Mulard duck circovirus | 2 | 2 | DNA | 3 |
| Virus | Mumps virus | 8 | 5 | RNA | NA |
| Virus | Mungbean yellow mosaic India virus | 9 | 2 | DNA | 3 |
| Virus | Mungbean yellow mosaic virus | 10 | 2 | DNA | 3 |
| Virus | Munia coronavirus HKU13-3514 | 9 | 4 | RNA | NA |
| Virus | Murid herpesvirus 1 | 161 | 1 | DNA | NA |
| Virus | Murid herpesvirus 2 | 167 | 1 | DNA | NA |
| Virus | Murid herpesvirus 4 | 74 | 1 | DNA | NA |
| Virus | Murine adenovirus 2 | 27 | 1 | DNA | NA |
| Virus | Murine adenovirus 3 | 28 | 1 | DNA | NA |
| Virus | Murine adenovirus A | 55 | 1 | DNA | NA |
| Virus | Murine astrovirus | 3 | 4 | RNA | 1 |
| Virus | Murine hepatitis virus strain A59 | 27 | 4 | RNA | NA |
| Virus | Murine hepatitis virus strain JHM | 27 | 4 | RNA | NA |
| Virus | Murine norovirus 1 | 10 | 4 | RNA | 1 |
| Virus | Murine osteosarcoma virus | 5 | 6 | RNA | 2 |
| Virus | Murine pneumotropic virus | 5 | 1 | DNA | 3 |
| Virus | Murine polyomavirus | 6 | 1 | DNA | 3 |
| Virus | Murine type C retrovirus | 2 | 6 | RNA | 2 |
| Virus | Murray Valley encephalitis virus | 16 | 4 | RNA | 1 |
| Virus | Mus musculus papillomavirus type 1 | 7 | 1 | DNA | 3 |
| Virus | Musca domestica salivary gland hypertrophy virus | 108 | 1 | DNA | NA |
| Virus | Muscovy duck circovirus | 4 | 2 | DNA | 3 |
| Virus | Muscovy duck parvovirus | 4 | 2 | DNA | 3 |
| Virus | Mushroom bacilliform virus | 4 | 4 | RNA | 1 |
| Virus | MW polyomavirus | 5 | 1 | DNA | 3 |
| Virus | Mycobacterium phage 244 | 142 | 1 | DNA | 4 |
| Virus | Mycobacterium phage Adjutor | 86 | 1 | DNA | 4 |
| Virus | Mycobacterium phage Angel | 61 | 1 | DNA | 4 |
| Virus | Mycobacterium phage Angelica | 94 | 1 | DNA | 4 |
| Virus | Mycobacterium phage Ardmore | 87 | 1 | DNA | NA |
| Virus | Mycobacterium phage Barnyard | 109 | 1 | DNA | 4 |
| Virus | Mycobacterium phage Bethlehem | 87 | 1 | DNA | 4 |
| Virus | Mycobacterium phage Boomer | 105 | 1 | DNA | 4 |
| Virus | Mycobacterium phage BPs | 63 | 1 | DNA | 4 |
| Virus | Mycobacterium phage Brujita | 74 | 1 | DNA | 4 |
| Virus | Mycobacterium phage Butterscotch | 86 | 1 | DNA | 4 |
| Virus | Mycobacterium phage Bxb1 | 86 | 1 | DNA | 4 |
| Virus | Mycobacterium phage Bxz1 | 225 | 1 | DNA | 4 |
| Virus | Mycobacterium phage Bxz2 | 86 | 1 | DNA | 4 |
| Virus | Mycobacterium phage Cali | 222 | 1 | DNA | 4 |
| Virus | Mycobacterium phage Catera | 218 | 1 | DNA | 4 |
| Virus | Mycobacterium phage Chah | 104 | 1 | DNA | 4 |
| Virus | Mycobacterium phage Che12 | 98 | 1 | DNA | 4 |
| Virus | Mycobacterium phage Che8 | 112 | 1 | DNA | 4 |
| Virus | Mycobacterium phage Che9c | 84 | 1 | DNA | 4 |
| Virus | Mycobacterium phage Che9d | 111 | 1 | DNA | 4 |
| Virus | Mycobacterium phage Cjw1 | 141 | 1 | DNA | 4 |
| Virus | Mycobacterium phage Cooper | 99 | 1 | DNA | 4 |
| Virus | Mycobacterium phage Corndog | 122 | 1 | DNA | 4 |
| Virus | Mycobacterium phage CrimD | 95 | 1 | DNA | 4 |
| Virus | Mycobacterium phage D29 | 79 | 1 | DNA | 4 |
| Virus | Mycobacterium phage DD5 | 87 | 1 | DNA | 4 |
| Virus | Mycobacterium phage ET08 | 218 | 1 | DNA | 4 |
| Virus | Mycobacterium phage Faith1 | 129 | 1 | DNA | 4 |
| Virus | Mycobacterium phage Fruitloop | 102 | 1 | DNA | 4 |
| Virus | Mycobacterium phage Giles | 78 | 1 | DNA | 4 |
| Virus | Mycobacterium phage Gumball | 88 | 1 | DNA | 4 |
| Virus | Mycobacterium phage Halo | 65 | 1 | DNA | 4 |
| Virus | Mycobacterium phage Jasper | 94 | 1 | DNA | 4 |
| Virus | Mycobacterium phage KBG | 89 | 1 | DNA | 4 |
| Virus | Mycobacterium phage Konstantine | 95 | 1 | DNA | 4 |
| Virus | Mycobacterium phage Kostya | 143 | 1 | DNA | 4 |
| Virus | Mycobacterium phage L5 | 85 | 1 | DNA | 4 |
| Virus | Mycobacterium phage LeBron | 123 | 1 | DNA | 4 |
| Virus | Mycobacterium phage Llij | 100 | 1 | DNA | 4 |
| Virus | Mycobacterium phage Lockley | 90 | 1 | DNA | 4 |
| Virus | Mycobacterium phage Myrna | 229 | 1 | DNA | 4 |
| Virus | Mycobacterium phage Nigel | 94 | 1 | DNA | 4 |
| Virus | Mycobacterium phage Omega | 237 | 1 | DNA | 4 |
| Virus | Mycobacterium phage Orion | 100 | 1 | DNA | 4 |
| Virus | Mycobacterium phage Pacc40 | 101 | 1 | DNA | 4 |
| Virus | Mycobacterium phage PBI1 | 81 | 1 | DNA | 4 |
| Virus | Mycobacterium phage Peaches | 86 | 1 | DNA | 4 |
| Virus | Mycobacterium phage PG1 | 100 | 1 | DNA | 4 |
| Virus | Mycobacterium phage Phaedrus | 98 | 1 | DNA | 4 |
| Virus | Mycobacterium phage Phlyer | 105 | 1 | DNA | 4 |
| Virus | Mycobacterium phage Pipefish | 102 | 1 | DNA | 4 |
| Virus | Mycobacterium phage PLot | 89 | 1 | DNA | 4 |
| Virus | Mycobacterium phage PMC | 104 | 1 | DNA | 4 |

| Type | Name | Number of genes | Baltimore class | Nucleic Acid | Monophyletic group |
|---|---|---|---|---|---|
| Virus | Mycobacterium phage Porky | 147 | 1 | DNA | 4 |
| Virus | Mycobacterium phage Predator | 92 | 1 | DNA | 4 |
| Virus | Mycobacterium phage Pukovnik | 88 | 1 | DNA | 4 |
| Virus | Mycobacterium phage Qyrzula | 81 | 1 | DNA | 4 |
| Virus | Mycobacterium phage Ramsey | 108 | 1 | DNA | 4 |
| Virus | Mycobacterium phage Rizal | 220 | 1 | DNA | 4 |
| Virus | Mycobacterium phage Rosebush | 90 | 1 | DNA | 4 |
| Virus | Mycobacterium phage ScottMcG | 221 | 1 | DNA | 4 |
| Virus | Mycobacterium phage Solon | 86 | 1 | DNA | 4 |
| Virus | Mycobacterium phage Spud | 222 | 1 | DNA | 4 |
| Virus | Mycobacterium phage SWU1 | 94 | 1 | DNA | 4 |
| Virus | Mycobacterium phage TM4 | 89 | 1 | DNA | 4 |
| Virus | Mycobacterium phage Troll4 | 84 | 1 | DNA | 4 |
| Virus | Mycobacterium phage Tweety | 109 | 1 | DNA | 4 |
| Virus | Mycobacterium phage U2 | 81 | 1 | DNA | 4 |
| Virus | Mycobacterium phage Wee | 108 | 1 | DNA | 4 |
| Virus | Mycobacterium phage Wildcat | 148 | 1 | DNA | 4 |
| Virus | Mycoplasma phage MAV1 | 15 | 1 | DNA | NA |
| Virus | Mycoplasma phage P1 | 11 | 1 | DNA | 4 |
| Virus | Mycoplasma phage phiMFV1 | 15 | NA | DNA | NA |
| Virus | Mycoreovirus 1 | 11 | 3 | RNA | NA |
| Virus | Mycoreovirus 3 | 12 | 3 | RNA | NA |
| Virus | Mycovirus FusoV | 2 | 3 | RNA | 1 |
| Virus | Myotis polyomavirus VM-2008 | 6 | 1 | DNA | 3 |
| Virus | Mythimna loreyi densovirus | 7 | 2 | DNA | 3 |
| Virus | Myxococcus phage Mx8 | 86 | 1 | DNA | 4 |
| Virus | Myxoma virus | 170 | 1 | DNA | 5 |
| Virus | Myzus persicae densovirus | 5 | 2 | DNA | 3 |
| Virus | Nam Dinh virus | 6 | 4 | RNA | NA |
| Virus | Nanovirus-like particle | 1 | 2 | DNA | NA |
| Virus | Narcissus common latent virus | 6 | 4 | RNA | 1 |
| Virus | Narcissus degeneration virus | 12 | 4 | RNA | 1 |
| Virus | Narcissus mosaic virus | 6 | 4 | RNA | 1 |
| Virus | Narcissus symptomless virus | 6 | 4 | RNA | 1 |
| Virus | Narcissus yellow stripe virus | 12 | 4 | RNA | 1 |
| Virus | Nariva virus | 8 | 5 | RNA | NA |
| Virus | Natrialba phage PhiCh1 | 98 | 1 | DNA | 4 |
| Virus | Ndumu virus | 4 | 4 | RNA | 1 |
| Virus | Nemesia ring necrosis virus | 3 | 4 | RNA | 1 |
| Virus | Neodiprion abietis NPV | 93 | 1 | DNA | NA |
| Virus | Neodiprion lecontei NPV | 89 | 1 | DNA | NA |
| Virus | Neodiprion sertifer NPV | 90 | 1 | DNA | NA |
| Virus | Nerine virus X | 5 | 4 | RNA | 1 |
| Virus | Newbury agent 1 | 4 | 4 | RNA | 1 |
| Virus | Newcastle disease virus B1 | 6 | 5 | RNA | NA |
| Virus | Ngaingan virus | 15 | 5 | RNA | NA |
| Virus | Night-heron coronavirus HKU19 | 8 | 4 | RNA | NA |
| Virus | Nilaparvata lugens reovirus | 11 | 3 | RNA | NA |
| Virus | Nipah virus | 8 | 5 | RNA | NA |
| Virus | Nocardia phage NBR1 | 68 | 1 | DNA | 4 |
| Virus | Nodamura virus | 5 | 4 | RNA | 1 |
| Virus | Nootka lupine vein clearing virus | 5 | 4 | RNA | 1 |
| Virus | Nora virus | 7 | 4 | RNA | NA |
| Virus | Northern cereal mosaic virus | 9 | 5 | RNA | NA |
| Virus | Norwalk virus | 9 | 4 | RNA | 1 |
| Virus | Ntaya virus | 1 | 4 | RNA | 1 |
| Virus | Nudaurelia capensis beta virus | 2 | 4 | RNA | 1 |
| Virus | Nyamanini virus | 6 | 5 | RNA | NA |
| Virus | O'nyong-nyong virus | 13 | 4 | RNA | 1 |
| Virus | oat blue dwarf virus | 3 | 4 | RNA | 1 |
| Virus | Oat blue dwarf virus | 2 | 4 | RNA | 1 |
| Virus | Oat chlorotic stunt virus | 4 | 4 | RNA | 1 |
| Virus | Oat dwarf virus | 4 | 2 | DNA | 3 |
| Virus | Oat golden stripe virus | 6 | 4 | RNA | 1 |
| Virus | Oat mosaic virus | 13 | 4 | RNA | 1 |
| Virus | Oat necrotic mottle virus | 12 | 4 | RNA | 1 |
| Virus | Obodhiang virus | 9 | 5 | RNA | NA |
| Virus | Obuda pepper virus | 4 | 4 | RNA | 1 |
| Virus | Odontoglossum ringspot virus | 5 | 4 | RNA | 1 |
| Virus | Okra leaf curl alphasatellite | 1 | 2 | DNA | NA |
| Virus | Okra leaf curl Cameroon virus | 6 | 2 | DNA | 3 |
| Virus | Okra leaf curl Mali virus satellite DNA beta | 1 | 2 | DNA | 3 |
| Virus | Okra leaf curl virus | 6 | 2 | DNA | 3 |
| Virus | Okra leaf curl virus satellite DNA beta | 1 | NA | NA | NA |
| Virus | Okra mosaic virus | 3 | 4 | RNA | 1 |
| Virus | Okra mottle virus | 6 | 2 | DNA | 3 |
| Virus | Okra yellow crinkle virus | 6 | 2 | DNA | 3 |
| Virus | Okra yellow mosaic Mexico virus | 7 | 2 | DNA | 3 |
| Virus | Okra yellow vein disease associated sequence | 1 | 2 | DNA | NA |
| Virus | Okra yellow vein mosaic virus | 6 | 2 | DNA | 3 |

| Type | Name | Number of genes | Baltimore class | Nucleic Acid | Monophyletic group |
|---|---|---|---|---|---|
| Virus | Olive latent virus 1 | 5 | 4 | RNA | 1 |
| Virus | Olive latent virus 2 | 4 | 4 | RNA | 1 |
| Virus | Olive latent virus 3 | 4 | 4 | RNA | 1 |
| Virus | Olive mild mosaic virus | 6 | 4 | RNA | 1 |
| Virus | Oliveros virus | 4 | 5 | RNA | NA |
| Virus | Omikronpapillomavirus 1 | 8 | 1 | DNA | 3 |
| Virus | Omsk hemorrhagic fever virus | 1 | 4 | RNA | 1 |
| Virus | Onion yellow dwarf virus | 12 | 4 | RNA | 1 |
| Virus | Ononis yellow mosaic virus | 3 | 4 | RNA | 1 |
| Virus | Operophtera brumata reovirus | 10 | 3 | RNA | NA |
| Virus | Ophiostoma mitovirus 3a | 1 | 4 | RNA | 1 |
| Virus | Ophiostoma mitovirus 4 | 1 | 4 | RNA | 1 |
| Virus | Ophiostoma mitovirus 5 | 1 | 4 | RNA | 1 |
| Virus | Ophiostoma mitovirus 6 | 1 | 4 | RNA | 1 |
| Virus | Opuntia virus X | 5 | 4 | RNA | 1 |
| Virus | Orangutan polyomavirus | 5 | 1 | DNA | 3 |
| Virus | Orchid fleck virus | 6 | 5 | RNA | NA |
| Virus | Orf virus | 130 | 1 | DNA | 5 |
| Virus | Organic Lake phycodnavirus isolate OLPV-scf7180000030512 | 48 | 1 | DNA | 5 |
| Virus | Organic Lake phycodnavirus isolate OLPV-scf7180000030567 | 31 | 1 | DNA | 5 |
| Virus | Organic Lake phycodnavirus isolate OLPV-scf7180000031019 | 26 | 1 | DNA | 5 |
| Virus | Organic Lake phycodnavirus isolate OLPV-scf7180000031023 | 38 | 1 | DNA | 5 |
| Virus | Organic Lake phycodnavirus isolate OLPV-scf7180000031026 | 38 | 1 | DNA | 5 |
| Virus | Organic_Lake_phycodnavirus_1 | 401 | 1 | DNA | 5 |
| Virus | Organic_Lake_phycodnavirus_2 | 326 | 1 | DNA | 5 |
| Virus | Organic_Lake_virophage | 24 | 1 | DNA | Unconsidered |
| Virus | Orgyia leucostigma NPV | 135 | 1 | DNA | NA |
| Virus | Orgyia pseudotsugata MNPV | 152 | 1 | DNA | NA |
| Virus | Ornithogalum mosaic virus | 11 | 4 | RNA | 1 |
| Virus | Oropouche virus | 4 | 5 | RNA | NA |
| Virus | Oryctes rhinoceros virus | 139 | 1 | DNA | NA |
| Virus | Oryza rufipogon endornavirus | 1 | 3 | RNA | NA |
| Virus | Oryza sativa endornavirus | 1 | 3 | RNA | NA |
| Virus | Ostreid herpesvirus 1 | 127 | 1 | DNA | NA |
| Virus | Ostreococcus lucimarinus virus OlV1 | 250 | 1 | DNA | 5 |
| Virus | Ostreococcus tauri virus 1 | 230 | 1 | DNA | 5 |
| Virus | Ostreococcus tauri virus 2 | 237 | 1 | DNA | 5 |
| Virus | Ostreococcus virus OsV5 | 264 | 1 | DNA | 5 |
| Virus | Ourmia melon virus | 3 | 4 | RNA | 1 |
| Virus | Ovine adenovirus A | 41 | 1 | DNA | NA |
| Virus | Ovine adenovirus D | 32 | 1 | DNA | NA |
| Virus | Ovine enzootic nasal tumour virus | 6 | 6 | RNA | NA |
| Virus | Ovine herpesvirus 2 | 73 | 1 | DNA | NA |
| Virus | Ovine lentivirus | 8 | 6 | RNA | 2 |
| Virus | Ovine papillomavirus - 1 | 5 | 1 | DNA | 3 |
| Virus | Oyster mushroom spherical virus | 7 | 4 | RNA | NA |
| Virus | Palyam virus | 11 | 3 | RNA | NA |
| Virus | Panax virus Y | 12 | 4 | RNA | 1 |
| Virus | Panicum mosaic satellite virus | 2 | NA | RNA | NA |
| Virus | Panicum mosaic virus | 6 | 4 | RNA | 1 |
| Virus | Panicum streak virus | 4 | 2 | DNA | 3 |
| Virus | Panine herpesvirus 2 | 169 | 1 | DNA | NA |
| Virus | Pantoea phage LIMEzero | 57 | 1 | DNA | 4 |
| Virus | Papaya leaf curl China virus | 6 | 2 | DNA | 3 |
| Virus | Papaya leaf curl China virus satellite DNA beta | 1 | 2 | DNA | 3 |
| Virus | Papaya leaf curl Guandong virus | 6 | 2 | DNA | 3 |
| Virus | Papaya leaf curl virus | 7 | 2 | DNA | 3 |
| Virus | Papaya leaf curl virus-associated DNA beta | 1 | 2 | DNA | 3 |
| Virus | Papaya leaf distortion mosaic virus | 12 | 4 | RNA | 1 |
| Virus | Papaya lethal yellowing virus | 4 | 4 | RNA | NA |
| Virus | Papaya mosaic virus | 5 | 4 | RNA | 1 |
| Virus | Papaya ringspot virus | 12 | 4 | RNA | 1 |
| Virus | Papiine herpesvirus 2 | 75 | 1 | DNA | NA |
| Virus | Papilio polyxenes densovirus | 3 | 2 | DNA | 3 |
| Virus | Papio hamadryas papillomavirus type 1 | 8 | 1 | DNA | 3 |
| Virus | Paprika mild mottle virus | 4 | 4 | RNA | 1 |
| Virus | Parainfluenza virus 5 | 8 | 5 | RNA | NA |
| Virus | Paralichthys olivaceus birnavirus | 3 | 3 | RNA | NA |
| Virus | Paramecium bursaria Chlorella virus 1 | 802 | 1 | DNA | 5 |
| Virus | Paramecium bursaria Chlorella virus AR158 | 814 | 1 | DNA | 5 |
| Virus | Paramecium bursaria Chlorella virus FR483 | 849 | 1 | DNA | 5 |
| Virus | Paramecium bursaria Chlorella virus NY2A | 886 | 1 | DNA | 5 |
| Virus | Parana virus | 4 | 5 | RNA | NA |
| Virus | Pariacato virus | 5 | 4 | RNA | 1 |
| Virus | Parietaria mottle virus | 5 | 4 | RNA | 1 |
| Virus | Parrot hepatitis B virus | 5 | 7 | DNA | 2 |
| Virus | Parsnip yellow fleck virus | 8 | 4 | RNA | 1 |
| Virus | Parvovirus H1 | 3 | 2 | DNA | 3 |
| Virus | Paspalum dilatatum striate mosaic virus | 4 | 2 | DNA | 3 |
| Virus | Paspalum striate mosaic virus | 4 | 2 | DNA | 3 |

| Type | Name | Number of genes | Baltimore class | Nucleic Acid | Monophyletic group |
|---|---|---|---|---|---|
| Virus | Passiflora latent carlavirus | 6 | 4 | RNA | 1 |
| Virus | Passion fruit mosaic virus | 4 | 4 | RNA | 1 |
| Virus | Passion fruit woodiness virus | 12 | 4 | RNA | 1 |
| Virus | Passionfruit severe leaf distortion virus | 7 | 2 | DNA | 3 |
| Virus | Pasteurella phage F108 | 44 | 1 | DNA | 4 |
| Virus | Patchouli mild mosaic virus | 10 | 4 | RNA | 1 |
| Virus | Pea early-browning virus | 7 | 4 | RNA | 1 |
| Virus | Pea enation mosaic virus-1 | 5 | 4 | RNA | 1 |
| Virus | Pea enation mosaic virus-2 | 4 | 4 | RNA | 1 |
| Virus | Pea seed-borne mosaic virus | 12 | 4 | RNA | 1 |
| Virus | Pea stem necrosis virus | 5 | 4 | RNA | 1 |
| Virus | Peach chlorotic mottle virus | 5 | 4 | RNA | 1 |
| Virus | Peach mosaic virus | 4 | 4 | RNA | 1 |
| Virus | Peanut chlorotic streak virus | 4 | 7 | DNA | 2 |
| Virus | Peanut clump virus | 8 | 4 | RNA | 1 |
| Virus | Peanut mottle virus | 12 | 4 | RNA | 1 |
| Virus | Peanut stunt virus | 5 | 4 | RNA | 1 |
| Virus | Pear latent virus | 5 | 4 | RNA | 1 |
| Virus | Pectobacterium phage My1 | 149 | 1 | DNA | 4 |
| Virus | Pedilanthus leaf curl virus | 6 | 2 | DNA | 3 |
| Virus | Pediococcus phage clP1 | 57 | 1 | DNA | 4 |
| Virus | Pelargonium chlorotic ring pattern virus | 6 | 4 | RNA | 1 |
| Virus | Pelargonium flower break virus | 6 | 4 | RNA | 1 |
| Virus | Pelargonium line pattern virus | 7 | 4 | RNA | 1 |
| Virus | Pelargonium necrotic spot virus | 5 | 4 | RNA | 1 |
| Virus | Pelargonium vein banding virus | 3 | 7 | DNA | 2 |
| Virus | Pelargonium zonate spot virus | 4 | 4 | RNA | 1 |
| Virus | Penaeid shrimp infectious myonecrosis virus | 2 | 3 | RNA | NA |
| Virus | Penaeus merguiensis densovirus | 3 | 2 | DNA | 3 |
| Virus | Penaeus monodon hepatopancreatic parvovirus | 3 | 2 | DNA | 3 |
| Virus | Penaeus vannamei nodavirus | 3 | 4 | RNA | NA |
| Virus | Penicillium chrysogenum virus | 4 | 3 | RNA | NA |
| Virus | Penicillium stoloniferum virus F | 2 | 3 | RNA | 1 |
| Virus | Penicillium stoloniferum virus S | 2 | 3 | RNA | 1 |
| Virus | Pennisetum mosaic virus | 12 | 4 | RNA | 1 |
| Virus | Pepino mosaic virus | 5 | 4 | RNA | 1 |
| Virus | Pepper curly top virus | 7 | 2 | DNA | 3 |
| Virus | Pepper golden mosaic virus | 6 | 2 | DNA | 3 |
| Virus | Pepper huasteco yellow vein virus | 7 | 2 | DNA | 3 |
| Virus | Pepper leaf curl Bangladesh virus | 6 | 2 | DNA | 3 |
| Virus | Pepper leaf curl virus | 6 | 2 | DNA | 3 |
| Virus | Pepper leaf curl virus satellite DNA beta | 1 | 2 | DNA | 3 |
| Virus | Pepper leaf curl Yunnan virus satellite DNA beta | 1 | 2 | DNA | 3 |
| Virus | Pepper mild mottle virus | 4 | 4 | RNA | 1 |
| Virus | Pepper mottle virus | 12 | 4 | RNA | 1 |
| Virus | Pepper ringspot virus | 5 | 4 | RNA | 1 |
| Virus | Pepper severe mosaic virus | 12 | 4 | RNA | 1 |
| Virus | Pepper vein yellows virus | 6 | 4 | RNA | 1 |
| Virus | Pepper veinal mottle virus | 12 | 4 | RNA | 1 |
| Virus | Pepper yellow dwarf virus - New Mexico | 7 | 2 | DNA | 3 |
| Virus | Pepper yellow leaf curl Indonesia virus | 7 | 2 | DNA | 3 |
| Virus | Pepper yellow mosaic virus | 12 | 4 | RNA | 1 |
| Virus | Pepper yellow vein Mali virus | 6 | 2 | DNA | 3 |
| Virus | Perina nuda virus | 1 | 4 | RNA | 1 |
| Virus | Periplaneta fuliginosa densovirus | 8 | 2 | DNA | 3 |
| Virus | Peristrophe mosaic virus | 2 | 2 | DNA | 3 |
| Virus | Persea americana endornavirus | 1 | 3 | RNA | NA |
| Virus | Persicivirga phage P12024L | 58 | 1 | DNA | NA |
| Virus | Persicivirga phage P12024S | 59 | 1 | DNA | NA |
| Virus | Persimmon cryptic virus | 2 | 3 | RNA | 1 |
| Virus | Peru tomato mosaic virus | 11 | 4 | RNA | 1 |
| Virus | Peruvian horse sickness virus | 11 | 3 | RNA | NA |
| Virus | Peste-des-petits-ruminants virus | 8 | 5 | RNA | NA |
| Virus | Pestivirus Giraffe-1 | 14 | 4 | RNA | 1 |
| Virus | Pestivirus strain Aydin/04-TR | 1 | 4 | RNA | 1 |
| Virus | Petunia vein clearing virus | 1 | 7 | DNA | 2 |
| Virus | Phage BP-4795 | 85 | 1 | DNA | 4 |
| Virus | Phage cdtI | 60 | 1 | DNA | 4 |
| Virus | Phage Gifsy-1 | 58 | NA | NA | NA |
| Virus | Phage Gifsy-2 | 55 | NA | NA | NA |
| Virus | Phage MP22 | 51 | 1 | DNA | 4 |
| Virus | Phage phiJL001 | 90 | 1 | DNA | 4 |
| Virus | Phage phiSMA9 | 7 | NA | DNA | NA |
| Virus | Phaius virus X | 5 | 4 | RNA | 1 |
| Virus | Phlebiopsis gigantea mycovirus dsRNA 1 | 2 | 3 | RNA | NA |
| Virus | Phlox Virus B | 6 | 4 | RNA | 1 |
| Virus | Phlox virus S | 6 | 4 | RNA | 1 |
| Virus | Phocoena phocoena papillomavirus 1 | 6 | 1 | DNA | 3 |
| Virus | Phocoena phocoena papillomavirus 2 | 6 | 1 | DNA | 3 |
| Virus | Phocoena phocoena papillomavirus 4 | 6 | 1 | DNA | 3 |

| Type | Name | Number of genes | Baltimore class | Nucleic Acid | Monophyletic group |
|---|---|---|---|---|---|
| Virus | Phormidium phage Pf-WMP3 | 41 | 1 | DNA | 4 |
| Virus | Phthorimaea operculella granulovirus | 130 | 1 | DNA | NA |
| Virus | Physalis mottle virus | 3 | 4 | RNA | 1 |
| Virus | Phytophthora endornavirus 1 | 1 | 3 | RNA | NA |
| Virus | Phytophthora infestans RNA virus 1 | 3 | 4 | RNA | NA |
| Virus | Pichinde virus | 4 | 5 | RNA | NA |
| Virus | Pieris rapae granulovirus | 120 | 1 | DNA | NA |
| Virus | Pig stool associated circular ssDNA virus GER2011 | 5 | 2 | DNA | NA |
| Virus | Pigeon picornavirus B | 13 | 4 | RNA | 1 |
| Virus | Pineapple bacilliform comosus virus | 3 | 7 | DNA | 2 |
| Virus | Pineapple mealybug wilt-associated virus 1 | 7 | 4 | RNA | 1 |
| Virus | Pirital virus | 4 | 5 | RNA | NA |
| Virus | Piscine myocarditis virus AL V-708 | 3 | 3 | RNA | NA |
| Virus | Planaria asexual strain-specific virus-like element type 1 | 6 | NA | NA | NA |
| Virus | Planktothrix phage PaV-LD | 142 | 1 | DNA | 4 |
| Virus | Planococcus citri densovirus | 4 | 2 | DNA | 3 |
| Virus | Plantago asiatica mosaic virus | 5 | 4 | RNA | 1 |
| Virus | Plantago mottle virus | 3 | 4 | RNA | 1 |
| Virus | Plautia stali intestine virus | 2 | 4 | RNA | 1 |
| Virus | Pleurotus ostreatus virus 1 | 2 | 3 | RNA | 1 |
| Virus | Plum bark necrosis and stem pitting-associated virus | 7 | 4 | RNA | 1 |
| Virus | Plum pox virus | 12 | 4 | RNA | 1 |
| Virus | Plutella xylostella granulovirus | 120 | 1 | DNA | NA |
| Virus | Plutella xylostella multiple nucleopolyhedrovirus | 152 | 1 | DNA | NA |
| Virus | Pneumonia virus of mice J3666 | 12 | 5 | RNA | NA |
| Virus | Poinsettia cryptic virus | 4 | 4 | RNA | 1 |
| Virus | Poinsettia mosaic virus | 5 | 4 | RNA | 1 |
| Virus | Pokeweed mosaic virus | 1 | 4 | RNA | 1 |
| Virus | Poliovirus | 12 | 4 | RNA | 1 |
| Virus | Polyomavirus HPyV6 | 5 | 1 | DNA | 3 |
| Virus | Polyomavirus HPyV7 | 5 | 1 | DNA | 3 |
| Virus | Poplar mosaic virus | 6 | 4 | RNA | 1 |
| Virus | Porcine adenovirus A | 29 | 1 | DNA | NA |
| Virus | Porcine adenovirus C | 58 | 1 | DNA | NA |
| Virus | Porcine associated stool circular virus | 2 | 2 | DNA | NA |
| Virus | Porcine bocavirus 3 | 4 | 2 | DNA | 3 |
| Virus | Porcine bocavirus 4-1 | 4 | 2 | DNA | 3 |
| Virus | Porcine bocavirus 5/JS677 | 4 | 2 | DNA | 3 |
| Virus | Porcine circovirus 1 | 2 | 2 | DNA | 3 |
| Virus | Porcine circovirus 2 | 3 | 2 | DNA | 3 |
| Virus | Porcine circovirus type 1/2a | 2 | 2 | DNA | 3 |
| Virus | Porcine coronavirus HKU15 | 7 | 4 | RNA | NA |
| Virus | Porcine enteric sapovirus | 2 | 4 | RNA | 1 |
| Virus | Porcine enterovirus B | 12 | 4 | RNA | 1 |
| Virus | Porcine epidemic diarrhea virus | 19 | 4 | RNA | NA |
| Virus | Porcine hemagglutinating encephalomyelitis virus | 10 | 4 | RNA | NA |
| Virus | Porcine kobuvirus SH-W-CHN/2010/China | 1 | 4 | RNA | 1 |
| Virus | Porcine kobuvirus swine/S-1-HUN/2007/Hungary | 12 | 4 | RNA | 1 |
| Virus | Porcine parvovirus | 6 | 2 | DNA | 3 |
| Virus | Porcine parvovirus 4 | 3 | 2 | DNA | 3 |
| Virus | Porcine reproductive and respiratory syndrome virus | 24 | 4 | RNA | NA |
| Virus | Porcine rubulavirus | 9 | 5 | RNA | NA |
| Virus | Porcine sapelovirus 1 | 12 | 4 | RNA | 1 |
| Virus | Porcine teschovirus | 13 | 4 | RNA | 1 |
| Virus | Potato apical leaf curl disease-associated satellite DNA beta | 1 | 2 | DNA | 3 |
| Virus | Potato aucuba mosaic virus | 6 | 4 | RNA | 1 |
| Virus | Potato latent virus | 6 | 4 | RNA | 1 |
| Virus | Potato leafroll virus | 7 | 4 | RNA | 1 |
| Virus | Potato mop-top virus | 8 | 4 | RNA | 1 |
| Virus | Potato virus A | 12 | 4 | RNA | 1 |
| Virus | Potato virus H | 6 | 4 | RNA | NA |
| Virus | Potato virus M | 8 | 4 | RNA | 1 |
| Virus | Potato Virus P | 6 | 4 | RNA | 1 |
| Virus | Potato virus S | 7 | 4 | RNA | 1 |
| Virus | Potato virus T | 3 | 4 | RNA | 1 |
| Virus | Potato virus V | 12 | 4 | RNA | 1 |
| Virus | Potato virus X | 5 | 4 | RNA | 1 |
| Virus | Potato virus Y | 12 | 4 | RNA | 1 |
| Virus | Potato yellow dwarf virus | 7 | 5 | RNA | NA |
| Virus | Potato yellow mosaic Panama virus | 7 | 2 | DNA | 3 |
| Virus | Potato yellow mosaic Trinidad virus | 7 | 2 | DNA | 3 |
| Virus | Potato yellow mosaic virus | 6 | 2 | DNA | 3 |
| Virus | Potato yellow vein virus | 13 | 4 | RNA | 1 |
| Virus | Pothos latent virus | 5 | 4 | RNA | 1 |
| Virus | Powassan virus | 14 | 4 | RNA | 1 |
| Virus | PreXMRV-1 | 2 | 6 | RNA | 2 |
| Virus | Primula malacoides virus China/Mar2007 | 2 | 3 | RNA | 1 |
| Virus | Prochlorococcus phage P-HM1 | 241 | 1 | DNA | 4 |
| Virus | Prochlorococcus phage P-HM2 | 242 | 1 | DNA | 4 |
| Virus | Prochlorococcus phage P-RSM4 | 239 | 1 | DNA | 4 |

| Type | Name | Number of genes | Baltimore class | Nucleic Acid | Monophyletic group |
|---|---|---|---|---|---|
| Virus | Prochlorococcus phage P-SSM2 | 334 | 1 | DNA | 4 |
| Virus | Prochlorococcus phage P-SSM4 | 221 | 1 | DNA | 4 |
| Virus | Prochlorococcus phage P-SSM7 | 237 | 1 | DNA | 4 |
| Virus | Prochlorococcus phage P-SSP7 | 58 | 1 | DNA | 4 |
| Virus | Prochlorococcus phage Syn1 | 234 | 1 | DNA | NA |
| Virus | Prochlorococcus phage Syn33 | 227 | 1 | DNA | NA |
| Virus | Procyon lotor papillomavirus 1 | 7 | 1 | DNA | 3 |
| Virus | Propionibacterium phage ATCC29399B_C | 46 | 1 | DNA | 4 |
| Virus | Propionibacterium phage ATCC29399B_T | 46 | 1 | DNA | 4 |
| Virus | Propionibacterium phage B5 | 10 | 2 | DNA | NA |
| Virus | Propionibacterium phage P1.1 | 45 | 1 | DNA | 4 |
| Virus | Propionibacterium phage P100_1 | 47 | 1 | DNA | 4 |
| Virus | Propionibacterium phage P100_A | 45 | 1 | DNA | 4 |
| Virus | Propionibacterium phage P100D | 47 | 1 | DNA | 4 |
| Virus | Propionibacterium phage P101A | 47 | 1 | DNA | 4 |
| Virus | Propionibacterium phage P104A | 45 | 1 | DNA | 4 |
| Virus | Propionibacterium phage P105 | 45 | 1 | DNA | 4 |
| Virus | Propionibacterium phage P14.4 | 47 | 1 | DNA | 4 |
| Virus | Propionibacterium phage P9.1 | 45 | 1 | DNA | 4 |
| Virus | Propionibacterium phage PA6 | 48 | 1 | DNA | 4 |
| Virus | Propionibacterium phage PAD20 | 45 | 1 | DNA | 4 |
| Virus | Propionibacterium phage PAS50 | 46 | 1 | DNA | 4 |
| Virus | Providence virus | 4 | 4 | RNA | 1 |
| Virus | Providencia phage Redjac | 41 | 1 | DNA | NA |
| Virus | Prune dwarf virus | 4 | 4 | RNA | 1 |
| Virus | Prunus necrotic ringspot virus | 4 | 4 | RNA | 1 |
| Virus | Pseudaletia unipuncta granulovirus | 183 | 1 | DNA | NA |
| Virus | Pseudoalteromonas phage H105/1 | 52 | 1 | DNA | 4 |
| Virus | Pseudoalteromonas phage PM2 | 22 | 1 | DNA | NA |
| Virus | Pseudocowpox virus | 134 | 1 | DNA | 5 |
| Virus | Pseudomonas phage 119X | 53 | 1 | DNA | 4 |
| Virus | Pseudomonas phage 14-1 | 90 | 1 | DNA | 4 |
| Virus | Pseudomonas phage 201phi2-1 | 461 | 1 | DNA | 4 |
| Virus | Pseudomonas phage 73 | 52 | 1 | DNA | 4 |
| Virus | Pseudomonas phage B3 | 59 | 1 | DNA | 4 |
| Virus | Pseudomonas phage Bf7 | 46 | 1 | DNA | 4 |
| Virus | Pseudomonas phage D3 | 95 | 1 | DNA | 4 |
| Virus | Pseudomonas phage D3112 | 55 | 1 | DNA | 4 |
| Virus | Pseudomonas phage DMS3 | 52 | 1 | DNA | 4 |
| Virus | Pseudomonas phage EL | 201 | 1 | DNA | 4 |
| Virus | Pseudomonas phage F10 | 63 | 1 | DNA | 4 |
| Virus | Pseudomonas phage F116 | 70 | 1 | DNA | 4 |
| Virus | Pseudomonas phage F8 | 91 | 1 | DNA | 4 |
| Virus | Pseudomonas phage gh-1 | 42 | 1 | DNA | 4 |
| Virus | Pseudomonas phage KPP10 | 146 | 1 | DNA | 4 |
| Virus | Pseudomonas phage LBL3 | 88 | 1 | DNA | 4 |
| Virus | Pseudomonas phage LIT1 | 90 | 1 | DNA | 4 |
| Virus | Pseudomonas phage LKD16 | 53 | 1 | DNA | 4 |
| Virus | Pseudomonas phage LMA2 | 94 | 1 | DNA | 4 |
| Virus | Pseudomonas phage Lu11 | 391 | 1 | DNA | 4 |
| Virus | Pseudomonas phage LUZ19 | 54 | 1 | DNA | 4 |
| Virus | Pseudomonas phage LUZ24 | 68 | 1 | DNA | 4 |
| Virus | Pseudomonas phage LUZ7 | 115 | 1 | DNA | 4 |
| Virus | Pseudomonas phage M6 | 85 | 1 | DNA | 4 |
| Virus | Pseudomonas phage MP1412 | 77 | 1 | DNA | 4 |
| Virus | Pseudomonas phage MP29 | 51 | 1 | DNA | 4 |
| Virus | Pseudomonas phage MP38 | 51 | 1 | DNA | 4 |
| Virus | Pseudomonas phage MP42 | 53 | 1 | DNA | 4 |
| Virus | Pseudomonas phage OBP | 309 | 1 | DNA | 4 |
| Virus | Pseudomonas phage PA11 | 70 | 1 | DNA | NA |
| Virus | Pseudomonas phage PAJU2 | 79 | 1 | DNA | 4 |
| Virus | Pseudomonas phage PAK_P1 | 158 | 1 | DNA | 4 |
| Virus | Pseudomonas phage PaP2 | 58 | 1 | DNA | 4 |
| Virus | Pseudomonas phage PaP3 | 71 | 1 | DNA | 4 |
| Virus | Pseudomonas phage PB1 | 93 | 1 | DNA | 4 |
| Virus | Pseudomonas phage Pf1 | 15 | 2 | DNA | NA |
| Virus | Pseudomonas phage Pf3 | 9 | 2 | DNA | NA |
| Virus | Pseudomonas phage phi-2 | 43 | 1 | DNA | 4 |
| Virus | Pseudomonas phage phi12 | 15 | 3 | RNA | NA |
| Virus | Pseudomonas phage phi13 | 13 | 3 | RNA | NA |
| Virus | Pseudomonas phage phi15 | 50 | 1 | DNA | 4 |
| Virus | Pseudomonas phage phi2954 | 14 | 3 | RNA | NA |
| Virus | Pseudomonas phage phi297 | 69 | 1 | DNA | 4 |
| Virus | Pseudomonas phage phi6 | 13 | 3 | RNA | NA |
| Virus | Pseudomonas phage phi8 | 19 | 3 | RNA | NA |
| Virus | Pseudomonas phage phiCTX | 47 | 1 | DNA | 4 |
| Virus | Pseudomonas phage phiIBB-PF7A | 52 | 1 | DNA | 4 |
| Virus | Pseudomonas phage phikF77 | 53 | 1 | DNA | 4 |
| Virus | Pseudomonas phage phiKMV | 49 | 1 | DNA | 4 |
| Virus | Pseudomonas phage phiKZ | 306 | 1 | DNA | 4 |

| Type | Name | Number of genes | Baltimore class | Nucleic Acid | Monophyletic group |
|---|---|---|---|---|---|
| Virus | Pseudomonas phage PP7 | 4 | 4 | RNA | 1 |
| Virus | Pseudomonas phage PRR1 | 4 | 4 | RNA | 1 |
| Virus | Pseudomonas phage PT2 | 54 | 1 | DNA | 4 |
| Virus | Pseudomonas phage PT5 | 52 | 1 | DNA | 4 |
| Virus | Pseudomonas phage SN | 92 | 1 | DNA | 4 |
| Virus | Pseudomonas phage tf | 72 | 1 | DNA | 4 |
| Virus | Pseudomonas phage UFV-P2 | 41 | 1 | DNA | 4 |
| Virus | Pseudomonas phage vB_Pae-Kakheti25 | 58 | 1 | DNA | 4 |
| Virus | Pseudomonas phage vB_Pae-TbilisiM32 | 51 | 1 | DNA | 4 |
| Virus | Pseudomonas phage vB_PaeS_PMG1 | 96 | 1 | DNA | 4 |
| Virus | Pseudomonas phage YuA | 77 | 1 | DNA | 4 |
| Virus | Psittacid herpesvirus 1 | 77 | 1 | DNA | NA |
| Virus | Pumpkin yellow mosaic Malaysia virus | 6 | 2 | DNA | 3 |
| Virus | Puumala virus | 3 | 5 | RNA | NA |
| Virus | Pyrobaculum spherical virus | 48 | 1 | DNA | NA |
| Virus | Pyrococcus abyssi virus 1 | 25 | 1 | DNA | NA |
| Virus | Quail picornavirus QPV1/HUN/2010 | 1 | 4 | RNA | 1 |
| Virus | Quang Binh virus | 3 | 4 | RNA | 1 |
| Virus | Rabbit calicivirus Australia 1 MIC-07 | 2 | 4 | RNA | 1 |
| Virus | Rabbit coronavirus HKU14 | 11 | 4 | RNA | NA |
| Virus | Rabbit fibroma virus | 165 | 1 | DNA | 5 |
| Virus | Rabbit hemorrhagic disease virus | 10 | 4 | RNA | 1 |
| Virus | Rabbit vesivirus | 3 | 4 | RNA | 1 |
| Virus | Rabies virus | 5 | 5 | RNA | NA |
| Virus | Rachiplusia ou MNPV | 149 | 1 | DNA | NA |
| Virus | Radish leaf curl betasatellite | 1 | 2 | DNA | NA |
| Virus | Radish leaf curl virus | 7 | 2 | DNA | 3 |
| Virus | Radish mosaic virus | 2 | 4 | RNA | 1 |
| Virus | Ralstonia phage p12J | 9 | 2 | DNA | NA |
| Virus | Ralstonia phage PE226 | 9 | 2 | DNA | NA |
| Virus | Ralstonia phage phiRSA1 | 51 | 1 | DNA | 4 |
| Virus | Ralstonia phage RSB1 | 47 | 1 | DNA | 4 |
| Virus | Ralstonia phage RSL1 | 343 | 1 | DNA | 4 |
| Virus | Ralstonia phage RSM1 | 15 | 2 | DNA | NA |
| Virus | Ralstonia phage RSM3 | 14 | 2 | DNA | NA |
| Virus | Ralstonia phage RSS1 | 12 | 2 | DNA | NA |
| Virus | Ramie mosaic virus | 8 | 2 | DNA | 3 |
| Virus | Ranid herpesvirus 1 | 132 | 1 | DNA | NA |
| Virus | Ranid herpesvirus 2 | 147 | 1 | DNA | NA |
| Virus | Raphanus sativus cryptic virus 1 | 2 | 3 | RNA | 1 |
| Virus | Raphanus sativus cryptic virus 2 | 3 | 3 | RNA | 1 |
| Virus | Raphanus sativus cryptic virus 3 | 2 | 3 | RNA | 1 |
| Virus | Raptor adenovirus A | 23 | 1 | DNA | NA |
| Virus | Raspberry bushy dwarf virus | 3 | 4 | RNA | 1 |
| Virus | Raspberry latent virus | 12 | 3 | RNA | NA |
| Virus | Raspberry leaf mottle virus | 10 | 4 | RNA | 1 |
| Virus | Raspberry ringspot virus | 4 | 4 | RNA | 1 |
| Virus | Rat coronavirus Parker | 10 | 4 | RNA | NA |
| Virus | Rattail cactus necrosis associated virus | 4 | 4 | RNA | 1 |
| Virus | Rattus norvegicus papillomavirus 1 EES-2009 | 6 | 1 | DNA | 3 |
| Virus | Rauscher murine leukemia virus | 3 | 6 | RNA | 2 |
| Virus | Raven circovirus | 2 | 2 | DNA | 3 |
| Virus | RD114 retrovirus | 2 | 6 | RNA | 2 |
| Virus | Red clover mottle virus | 10 | 4 | RNA | 1 |
| Virus | Red clover necrotic mosaic virus | 4 | 4 | RNA | 1 |
| Virus | Red clover vein mosaic virus | 6 | 4 | RNA | 1 |
| Virus | Redspotted grouper nervous necrosis virus | 3 | 4 | RNA | 1 |
| Virus | Rehmannia mosaic virus | 4 | 4 | RNA | 1 |
| Virus | Respiratory syncytial virus | 10 | 5 | RNA | NA |
| Virus | Reston ebolavirus | 8 | 5 | RNA | NA |
| Virus | Reticuloendotheliosis virus | 3 | 6 | RNA | 2 |
| Virus | Rhizobium phage 16-3 | 110 | 1 | DNA | 4 |
| Virus | Rhizoctonia solani virus 717 | 2 | 3 | RNA | 1 |
| Virus | Rhizosolenia setigera RNA virus 01 | 2 | 4 | RNA | 1 |
| Virus | Rhodobacter phage RcapMu | 58 | 1 | DNA | 4 |
| Virus | Rhodococcus phage REQ1 | 85 | 1 | DNA | 4 |
| Virus | Rhodococcus phage REQ2 | 82 | 1 | DNA | 4 |
| Virus | Rhodococcus phage REQ3 | 60 | NA | NA | NA |
| Virus | Rhodococcus phage RER2 | 66 | 1 | DNA | 4 |
| Virus | Rhodococcus phage RGL3 | 66 | 1 | DNA | 4 |
| Virus | Rhodococcus phage RRH1 | 20 | 1 | DNA | 4 |
| Virus | Rhododendron virus A | 2 | 3 | RNA | NA |
| Virus | Rhodothermus phage RM378 | 146 | 1 | DNA | 4 |
| Virus | Rhopalosiphum padi virus | 2 | 4 | RNA | 1 |
| Virus | Rhynchosai mild mosaic virus | 6 | 2 | DNA | 3 |
| Virus | Rhynchosia golden mosaic virus | 7 | 2 | DNA | 3 |
| Virus | Rhynchosia golden mosaic Yucatan virus | 6 | 2 | DNA | 3 |
| Virus | Rhynchosia yellow mosaic India virus | 8 | 2 | DNA | 3 |
| Virus | Ribgrass mosaic virus | 4 | 4 | RNA | 1 |
| Virus | Rice black streaked dwarf virus | 13 | 3 | RNA | NA |

| Type | Name | Number of genes | Baltimore class | Nucleic Acid | Monophyletic group |
|---|---|---|---|---|---|
| Virus | Rice dwarf virus | 13 | 3 | RNA | NA |
| Virus | Rice gall dwarf virus | 12 | 3 | RNA | NA |
| Virus | Rice grassy stunt virus | 12 | 5 | RNA | NA |
| Virus | Rice ragged stunt virus | 13 | 3 | RNA | NA |
| Virus | Rice stripe virus | 7 | 4 | RNA | 1 |
| Virus | Rice tungro bacilliform virus | 4 | 7 | DNA | 2 |
| Virus | Rice tungro spherical virus | 8 | 4 | RNA | 1 |
| Virus | Rice yellow mottle virus | 7 | 4 | RNA | 1 |
| Virus | Rice yellow stunt virus | 7 | 5 | RNA | NA |
| Virus | Rift Valley fever virus | 4 | 5 | RNA | NA |
| Virus | Rinderpest virus (strain Kabete O) | 7 | 5 | RNA | NA |
| Virus | Rio Bravo virus | 14 | 4 | RNA | 1 |
| Virus | Rodent herpesvirus Peru | 82 | 1 | DNA | NA |
| Virus | Rose cryptic virus 1 | 3 | 3 | RNA | 1 |
| Virus | Rose rosette virus | 4 | 4 | RNA | 1 |
| Virus | Rose spring dwarf-associated virus | 8 | 4 | RNA | 1 |
| Virus | Rose yellow mosaic virus | 10 | 4 | RNA | 1 |
| Virus | Rosellinia necatrix megabirnavirus 1/W779 | 4 | NA | RNA | NA |
| Virus | Rosellinia necatrix partitivirus 1-W8 | 2 | 3 | RNA | 1 |
| Virus | Rosellinia necatrix quadrivirus 1 | 4 | 3 | RNA | NA |
| Virus | Roseobacter phage RDJL Phi 1 | 87 | 1 | DNA | 4 |
| Virus | Roseobacter phage SIO1 | 34 | 1 | DNA | 4 |
| Virus | Roseophage DSS3P2 | 81 | 1 | DNA | NA |
| Virus | Roseophage EE36P1 | 79 | 1 | DNA | NA |
| Virus | Ross River virus | 13 | 4 | RNA | 1 |
| Virus | Ross's goose hepatitis B virus | 3 | 7 | DNA | 2 |
| Virus | Rotavirus A | 12 | 3 | RNA | NA |
| Virus | Rotavirus C | 11 | 3 | RNA | NA |
| Virus | Rotavirus D chicken/05V0049/DEU/2005 | 12 | 3 | RNA | NA |
| Virus | Rous sarcoma virus | 14 | 6 | RNA | 2 |
| Virus | Rousettus aegyptiacus papillomavirus type 1 | 7 | 1 | DNA | 3 |
| Virus | Rousettus bat coronavirus HKU10 | 9 | 4 | RNA | NA |
| Virus | Rubella virus | 7 | 4 | RNA | 1 |
| Virus | Rubus canadensis virus 1 | 5 | 4 | RNA | NA |
| Virus | Rubus chlorotic mottle virus | 4 | 4 | RNA | NA |
| Virus | Rudbeckia flower distortion virus | 7 | 7 | DNA | 2 |
| Virus | Rupestris stem pitting-associated virus | 5 | 4 | RNA | NA |
| Virus | Ryegrass mosaic virus | 12 | 4 | RNA | 1 |
| Virus | Ryegrass mottle virus | 7 | 4 | RNA | 1 |
| Virus | Sabia virus | 4 | 5 | RNA | NA |
| Virus | Sacbrood virus | 1 | 4 | RNA | 1 |
| Virus | Saccharomyces 20S RNA narnavirus | 1 | 4 | RNA | 1 |
| Virus | Saccharomyces 23S RNA narnavirus | 1 | 4 | RNA | 1 |
| Virus | Saccharomyces cerevisiae killer virus M1 | 1 | 3 | RNA | NA |
| Virus | Saccharomyces cerevisiae virus L-A | 3 | 3 | RNA | NA |
| Virus | Saccharomyces cerevisiae virus L-BC (La) | 2 | 3 | RNA | NA |
| Virus | Saccharum streak virus | 3 | 2 | DNA | 3 |
| Virus | Saffold virus | 13 | 4 | RNA | 1 |
| Virus | Saguaro cactus virus | 8 | 4 | RNA | 1 |
| Virus | Saimiriine herpesvirus 1 | 70 | 1 | DNA | NA |
| Virus | Saimiriine herpesvirus 2 | 76 | 1 | DNA | NA |
| Virus | Saimiriine herpesvirus 3 | 142 | 1 | DNA | NA |
| Virus | Salisaeta icosahedral phage 1 | 57 | 1 | DNA | NA |
| Virus | Salivirus NG-J1 | 12 | 4 | RNA | 1 |
| Virus | Salmon pancreas disease virus | 13 | 4 | RNA | 1 |
| Virus | Salmonella enterica bacteriophage SE1 | 67 | 1 | DNA | 4 |
| Virus | Salmonella phage 7-11 | 151 | 1 | DNA | 4 |
| Virus | Salmonella phage c341 | 68 | 1 | DNA | NA |
| Virus | Salmonella phage E1 | 51 | 1 | DNA | NA |
| Virus | Salmonella phage epsilon34 | 73 | 1 | DNA | 4 |
| Virus | Salmonella phage Felix01 | 131 | 1 | DNA | 4 |
| Virus | Salmonella phage Fels-1 | 52 | NA | NA | NA |
| Virus | Salmonella phage Fels-2 | 47 | 1 | DNA | 4 |
| Virus | Salmonella phage phiSG-JL2 | 55 | 1 | DNA | 4 |
| Virus | Salmonella phage PVP-SE1 | 244 | 1 | DNA | 4 |
| Virus | Salmonella phage SE2 | 61 | 1 | DNA | 4 |
| Virus | Salmonella phage SETP3 | 53 | 1 | DNA | 4 |
| Virus | Salmonella phage SFP10 | 201 | 1 | DNA | 4 |
| Virus | Salmonella phage SPN19 | 72 | 1 | DNA | 4 |
| Virus | Salmonella phage SPN1S | 52 | 1 | DNA | 4 |
| Virus | Salmonella phage SPN9CC | 62 | 1 | DNA | 4 |
| Virus | Salmonella phage SS3e | 58 | 1 | DNA | 4 |
| Virus | Salmonella phage SSU5 | 130 | 1 | DNA | 4 |
| Virus | Salmonella phage ST160 | 63 | 1 | DNA | 4 |
| Virus | Salmonella phage ST64B | 56 | 1 | DNA | NA |
| Virus | Salmonella phage vB_SemP_Emek | 70 | 1 | DNA | 4 |
| Virus | Salmonella phage vB_SosS_Oslo | 79 | 1 | DNA | 4 |
| Virus | Salmonella phage Vi01 | 208 | 1 | DNA | 4 |
| Virus | Salmonella phage Vi06 | 47 | 1 | DNA | 4 |
| Virus | Sandfly fever Naples virus | 4 | 5 | RNA | NA |

| Type | Name | Number of genes | Baltimore class | Nucleic Acid | Monophyletic group |
|---|---|---|---|---|---|
| Virus | Sandfly fever Turkey virus | 4 | 5 | RNA | NA |
| Virus | Santeuil nodavirus | 3 | 4 | RNA | 1 |
| Virus | Sapovirus C12 | 3 | 4 | RNA | 1 |
| Virus | Sapovirus Hu/Dresden/pJG-Sap01/DE | 3 | 4 | RNA | 1 |
| Virus | Sapovirus Mc10 | 3 | 4 | RNA | 1 |
| Virus | SARS coronavirus | 30 | 4 | RNA | NA |
| Virus | Satellite virus of maize white line mosaic virus | 1 | NA | NA | NA |
| Virus | Sathuperi virus | 4 | 5 | RNA | NA |
| Virus | Satsuma dwarf virus | 8 | 4 | RNA | 1 |
| Virus | Scallion mosaic virus | 12 | 4 | RNA | 1 |
| Virus | Scallion virus X | 5 | 4 | RNA | 1 |
| Virus | Schizochytrium single-stranded RNA virus | 2 | 4 | RNA | 1 |
| Virus | Schlumbergera virus X | 5 | 4 | RNA | 1 |
| Virus | Sclerophthora macrospora virus A | 3 | 4 | RNA | NA |
| Virus | Sclerophthora macrospora virus B | 2 | 4 | RNA | NA |
| Virus | Sclerotinia sclerotiorum debilitation-associated RNA virus | 1 | 4 | RNA | 1 |
| Virus | Sclerotinia sclerotiorum dsRNA mycovirus-L | 2 | 3 | RNA | NA |
| Virus | Sclerotinia sclerotiorum hypovirulence associated DNA virus 1 | 2 | 2 | DNA | NA |
| Virus | Sclerotinia sclerotiorum hypovirus 1 | 1 | 3 | RNA | NA |
| Virus | Sclerotinia sclerotiorum partitivirus S | 2 | 3 | RNA | 1 |
| Virus | Scotophilus bat coronavirus 512 | 6 | 4 | RNA | NA |
| Virus | Scrophularia mottle virus | 3 | 4 | RNA | 1 |
| Virus | Sea turtle tornovirus 1 | 3 | 2 | DNA | NA |
| Virus | Seal anellovirus TFFN/USA/2006 | 3 | 2 | DNA | NA |
| Virus | Seal picornavirus type 1 | 12 | 4 | RNA | 1 |
| Virus | Semliki forest virus | 14 | 4 | RNA | 1 |
| Virus | Sendai virus | 10 | 5 | RNA | NA |
| Virus | Seneca valley virus | 1 | 4 | RNA | 1 |
| Virus | Senecio yellow mosaic virus | 6 | 2 | DNA | 3 |
| Virus | Seoul virus | 3 | 5 | RNA | NA |
| Virus | Sepik virus | 1 | 4 | RNA | 1 |
| Virus | Sesbania mosaic virus | 8 | 4 | RNA | 1 |
| Virus | SFTS virus HB29 | 6 | 5 | RNA | NA |
| Virus | Shallot virus X | 6 | 4 | RNA | 1 |
| Virus | Shallot yellow stripe virus | 13 | 4 | RNA | 1 |
| Virus | Shamonda virus | 4 | 5 | RNA | NA |
| Virus | Sheeppox virus | 148 | 1 | DNA | 5 |
| Virus | Sheldgoose hepatitis B virus | 5 | 7 | DNA | 2 |
| Virus | Shigella phage EP23 | 57 | 1 | DNA | 4 |
| Virus | Shigella phage phiSboM-AG3 | 216 | 1 | DNA | 4 |
| Virus | Shigella phage Shfl1 | 80 | 1 | DNA | 4 |
| Virus | Shigella phage Shfl2 | 265 | 1 | DNA | 4 |
| Virus | Shigella phage SP18 | 284 | 1 | DNA | 4 |
| Virus | Shrimp white spot syndrome virus | 531 | 1 | DNA | NA |
| Virus | Sibine fusca densovirus | 3 | 2 | DNA | 3 |
| Virus | Sida golden mosaic Costa Rica virus | 7 | 2 | DNA | 3 |
| Virus | Sida golden mosaic Florida virus | 7 | 2 | DNA | 3 |
| Virus | Sida golden mosaic Florida virus-Malvastrum | 7 | 2 | DNA | 3 |
| Virus | Sida golden mosaic Honduras virus | 7 | 2 | DNA | 3 |
| Virus | Sida golden mosaic virus | 7 | 2 | DNA | 3 |
| Virus | Sida golden mottle virus | 7 | 2 | DNA | 3 |
| Virus | Sida leaf curl virus | 6 | 2 | DNA | 3 |
| Virus | Sida leaf curl virus satellite DNA beta | 1 | 2 | DNA | 3 |
| Virus | Sida leaf curl virus-associated DNA 1 | 1 | 2 | DNA | 3 |
| Virus | Sida leaf curl virus-associated DNA beta | 1 | 2 | DNA | 3 |
| Virus | Sida micrantha mosaic virus | 7 | 2 | DNA | 3 |
| Virus | Sida mosaic Alagoas virus | 7 | 2 | DNA | 3 |
| Virus | Sida mosaic Bolivia virus 1 | 7 | 2 | DNA | 3 |
| Virus | Sida mosaic Bolivia virus 2 | 7 | 2 | DNA | 3 |
| Virus | Sida mosaic Sinaloa virus | 7 | 2 | DNA | 3 |
| Virus | Sida mottle virus | 6 | 2 | DNA | 3 |
| Virus | Sida yellow mosaic virus | 6 | 2 | DNA | 3 |
| Virus | Sida yellow mosaic Yucatan virus | 6 | 2 | DNA | 3 |
| Virus | Sida yellow mottle virus | 7 | 2 | DNA | 3 |
| Virus | Sida yellow vein disease associated DNA 1 | 1 | 2 | DNA | NA |
| Virus | Sida yellow vein Madurai virus | 6 | 2 | DNA | 3 |
| Virus | Sida yellow vein Vietnam alphasatellite | 1 | 2 | DNA | NA |
| Virus | Sida yellow vein Vietnam virus | 6 | 2 | DNA | 3 |
| Virus | Sida yellow vein Vietnam virus satellite DNA beta | 1 | 2 | DNA | 3 |
| Virus | Sida yellow vein virus | 7 | 2 | DNA | 3 |
| Virus | Sida yellow vein virus satellite DNA beta | 1 | 2 | DNA | NA |
| Virus | Siegesbeckia yellow vein Guangxi virus | 6 | 2 | DNA | 3 |
| Virus | Siegesbeckia yellow vein virus | 6 | 2 | DNA | 3 |
| Virus | Siegesbeckia yellow vein virus-associated DNA beta | 1 | 2 | DNA | NA |
| Virus | Simbu virus | 4 | 5 | RNA | NA |
| Virus | Simian adenovirus 1 | 35 | 1 | DNA | NA |
| Virus | Simian adenovirus 21 | 38 | 1 | DNA | NA |
| Virus | Simian adenovirus 25 | 38 | 1 | DNA | NA |
| Virus | Simian adenovirus 3 | 34 | 1 | DNA | NA |
| Virus | Simian adenovirus 49 | 32 | 1 | DNA | NA |

| Type | Name | Number of genes | Baltimore class | Nucleic Acid | Monophyletic group |
|---|---|---|---|---|---|
| Virus | Simian agent 12 | 6 | 1 | DNA | 3 |
| Virus | Simian enterovirus A | 12 | 4 | RNA | 1 |
| Virus | Simian enterovirus SV19 | 12 | 4 | RNA | 1 |
| Virus | Simian enterovirus SV43 | 12 | 4 | RNA | 1 |
| Virus | Simian enterovirus SV6 | 12 | 4 | RNA | 1 |
| Virus | Simian foamy virus | 6 | 6 | RNA | 2 |
| Virus | Simian hemorrhagic fever virus | 15 | 4 | RNA | NA |
| Virus | Simian immunodeficiency virus | 6 | 6 | RNA | 2 |
| Virus | Simian immunodeficiency virus SIV-mnd 2 | 8 | 6 | RNA | 2 |
| Virus | Simian picornavirus 17 | 12 | 4 | RNA | 1 |
| Virus | Simian picornavirus strain N125 | 12 | 4 | RNA | 1 |
| Virus | Simian picornavirus strain N203 | 12 | 4 | RNA | 1 |
| Virus | Simian retrovirus 4 | 4 | 6 | RNA | 2 |
| Virus | Simian sapelovirus 1 | 13 | 4 | RNA | 1 |
| Virus | Simian T-cell lymphotropic virus 6 | 6 | 6 | RNA | 2 |
| Virus | Simian T-lymphotropic virus 1 | 4 | 6 | RNA | 2 |
| Virus | Simian T-lymphotropic virus 2 | 12 | 6 | RNA | 2 |
| Virus | Simian T-lymphotropic virus 3 | 5 | 6 | RNA | 2 |
| Virus | Simian virus 12 | 6 | 1 | DNA | 3 |
| Virus | Simian virus 40 | 7 | 1 | DNA | 3 |
| Virus | Simian virus 41 | 7 | 5 | RNA | NA |
| Virus | Simian-Human immunodeficiency virus | 10 | 6 | RNA | 2 |
| Virus | Sin Nombre virus | 4 | 5 | RNA | NA |
| Virus | Sindbis virus | 14 | 4 | RNA | 1 |
| Virus | Singapore grouper iridovirus | 162 | 1 | DNA | 5 |
| Virus | Siniperca chuatsi rhabdovirus | 6 | 5 | RNA | NA |
| Virus | Sinorhizobium phage PBC5 | 83 | 1 | DNA | NA |
| Virus | Sleeping disease virus | 13 | 4 | RNA | 1 |
| Virus | Slow bee paralysis virus | 1 | 4 | RNA | 1 |
| Virus | Small anellovirus 1 | 3 | 2 | DNA | NA |
| Virus | Small anellovirus 2 | 5 | 2 | DNA | NA |
| Virus | Snake adenovirus 1 | 28 | 1 | DNA | NA |
| Virus | Snake parvovirus 1 | 2 | 2 | DNA | 3 |
| Virus | Snakehead retrovirus | 7 | 6 | RNA | 2 |
| Virus | Snakehead virus | 6 | 5 | RNA | NA |
| Virus | Snow goose hepatitis B virus | 5 | 7 | DNA | 2 |
| Virus | Sodalis phage phiSG1 | 47 | NA | NA | NA |
| Virus | Sodalis phage SO-1 | 59 | 1 | DNA | 4 |
| Virus | Soil-borne cereal mosaic virus | 6 | 4 | RNA | 1 |
| Virus | Soil-borne wheat mosaic virus | 6 | 4 | RNA | 1 |
| Virus | Solenopsis invicta virus 2 | 4 | 4 | RNA | NA |
| Virus | Solenopsis invicta virus 3 | 2 | 4 | RNA | NA |
| Virus | Solenopsis invicta virus-1 | 2 | 4 | RNA | 1 |
| Virus | Sonchus yellow net virus | 6 | 5 | RNA | NA |
| Virus | Sorghum chlorotic spot virus | 7 | 4 | RNA | 1 |
| Virus | Sorghum mosaic virus | 12 | 4 | RNA | 1 |
| Virus | South African cassava mosaic virus | 8 | 2 | DNA | 3 |
| Virus | South polar skua adenovirus-1 | 23 | 1 | DNA | NA |
| Virus | Southern bean mosaic virus | 7 | 4 | RNA | 1 |
| Virus | Southern cowpea mosaic virus | 7 | 4 | RNA | 1 |
| Virus | Southern elephant seal virus | 4 | 4 | RNA | 1 |
| Virus | Southern rice black-streaked dwarf virus | 13 | 3 | RNA | NA |
| Virus | Southern tomato virus | 2 | 3 | RNA | NA |
| Virus | Sowbane mosaic virus | 4 | 4 | RNA | 1 |
| Virus | Soybean chlorotic blotch virus | 9 | 2 | DNA | 3 |
| Virus | Soybean chlorotic mottle virus | 9 | 7 | DNA | 2 |
| Virus | Soybean chlorotic spot virus | 6 | 2 | DNA | 3 |
| Virus | Soybean crinkle leaf virus | 8 | 2 | DNA | 3 |
| Virus | Soybean dwarf virus | 5 | 4 | RNA | 1 |
| Virus | Soybean mild mottle virus | 6 | 2 | DNA | 3 |
| Virus | Soybean mosaic virus | 12 | 4 | RNA | 1 |
| Virus | Soybean Putnam virus | 6 | 7 | DNA | 2 |
| Virus | Soybean yellow common mosaic virus | 4 | 4 | RNA | NA |
| Virus | Soybean yellow mottle mosaic virus | 5 | 4 | RNA | 1 |
| Virus | Sparrow coronavirus HKU17 | 8 | 4 | RNA | NA |
| Virus | Sphaeropsis sapinea RNA virus 1 | 2 | 3 | RNA | NA |
| Virus | Sphaeropsis sapinea RNA virus 2 | 2 | 3 | RNA | NA |
| Virus | Spilanthes yellow vein virus | 6 | 2 | DNA | 3 |
| Virus | Spinach curly top Arizona virus | 5 | 2 | DNA | 3 |
| Virus | Spinach curly top virus | 7 | 2 | DNA | 3 |
| Virus | Spinach latent virus | 5 | 4 | RNA | 1 |
| Virus | Spinach severe curly top virus | 6 | 2 | DNA | 3 |
| Virus | Spiroplasma kunkelii virus SkV1_CR2-3x | 13 | 2 | DNA | NA |
| Virus | Spiroplasma phage 1-C74 | 13 | 2 | DNA | NA |
| Virus | Spiroplasma phage 1-R8A2B | 12 | 2 | DNA | NA |
| Virus | Spiroplasma phage 4 | 9 | 2 | DNA | NA |
| Virus | Spiroplasma phage SVTS2 | 13 | 2 | DNA | NA |
| Virus | Spissistilus festinus reovirus | 10 | 3 | RNA | NA |
| Virus | Spissistilus festinus virus 1 | 2 | 3 | RNA | NA |
| Virus | Spleen focus-forming virus | 4 | 6 | RNA | 2 |

| Type | Name | Number of genes | Baltimore class | Nucleic Acid | Monophyletic group |
|---|---|---|---|---|---|
| Virus | Spodoptera exigua iflavirus 1 | 3 | 4 | RNA | 1 |
| Virus | Spodoptera exigua MNPV | 139 | 1 | DNA | NA |
| Virus | Spodoptera frugiperda ascovirus 1a | 123 | 1 | DNA | 5 |
| Virus | Spodoptera frugiperda MNPV | 143 | 1 | DNA | NA |
| Virus | Spodoptera litura granulovirus | 136 | 1 | DNA | NA |
| Virus | Spodoptera litura NPV | 141 | 1 | DNA | NA |
| Virus | Spodoptera litura nucleopolyhedrovirus II | 147 | 1 | DNA | NA |
| Virus | Sporobolus striate mosaic virus 1 | 4 | 2 | DNA | 3 |
| Virus | Sporobolus striate mosaic virus 2 | 4 | 2 | DNA | 3 |
| Virus | Spring beauty latent virus | 4 | 4 | RNA | 1 |
| Virus | Spring viraemia of carp virus | 5 | 5 | RNA | NA |
| Virus | Sputnik virophage | 21 | 1 | DNA | Unconsidered |
| Virus | Sputnik2_proteome_aa | 20 | 1 | DNA | Unconsidered |
| Virus | Sputnik3_proteome_aa | 20 | 1 | DNA | Unconsidered |
| Virus | Squash leaf curl China virus | 2 | 2 | DNA | 3 |
| Virus | Squash leaf curl Philippines virus | 7 | 2 | DNA | 3 |
| Virus | Squash leaf curl virus | 6 | 2 | DNA | 3 |
| Virus | Squash leaf curl Yunnan virus | 6 | 2 | DNA | 3 |
| Virus | Squash mild leaf curl virus | 6 | 2 | DNA | 3 |
| Virus | Squash mosaic virus | 10 | 4 | RNA | 1 |
| Virus | Squash vein yellowing virus | 12 | 4 | RNA | 1 |
| Virus | Squash yellow mild mottle virus | 6 | 2 | DNA | 3 |
| Virus | Squirrel monkey polyomavirus | 6 | 1 | DNA | 3 |
| Virus | Squirrel monkey retrovirus | 6 | 6 | RNA | 2 |
| Virus | Sri Lankan cassava mosaic virus | 8 | 2 | DNA | 3 |
| Virus | St Croix River virus | 10 | 3 | RNA | NA |
| Virus | St. Augustine decline satellite virus | 1 | NA | NA | NA |
| Virus | St. Louis encephalitis virus | 1 | 4 | RNA | 1 |
| Virus | Stachytarpheta leaf curl virus | 6 | 2 | DNA | 3 |
| Virus | Staphylococcus aureus phage P68 | 22 | 1 | DNA | NA |
| Virus | Staphylococcus phage 11 | 53 | 1 | DNA | 4 |
| Virus | Staphylococcus phage 187 | 77 | 1 | DNA | 4 |
| Virus | Staphylococcus phage 2638A | 57 | 1 | DNA | 4 |
| Virus | Staphylococcus phage 29 | 67 | 1 | DNA | 4 |
| Virus | Staphylococcus phage 37 | 70 | 1 | DNA | 4 |
| Virus | Staphylococcus phage 3A | 67 | 1 | DNA | 4 |
| Virus | Staphylococcus phage 42E | 79 | 1 | DNA | 4 |
| Virus | Staphylococcus phage 44AHJD | 21 | 1 | DNA | 4 |
| Virus | Staphylococcus phage 47 | 65 | 1 | DNA | 4 |
| Virus | Staphylococcus phage 52A | 60 | 1 | DNA | 4 |
| Virus | Staphylococcus phage 53 | 74 | 1 | DNA | 4 |
| Virus | Staphylococcus phage 55 | 77 | 1 | DNA | 4 |
| Virus | Staphylococcus phage 66 | 27 | 1 | DNA | 4 |
| Virus | Staphylococcus phage 69 | 69 | 1 | DNA | 4 |
| Virus | Staphylococcus phage 71 | 67 | 1 | DNA | 4 |
| Virus | Staphylococcus phage 77 | 69 | 1 | DNA | 4 |
| Virus | Staphylococcus phage 80alpha | 73 | 1 | DNA | 4 |
| Virus | Staphylococcus phage 85 | 71 | 1 | DNA | 4 |
| Virus | Staphylococcus phage 88 | 66 | 1 | DNA | 4 |
| Virus | Staphylococcus phage 92 | 64 | 1 | DNA | 4 |
| Virus | Staphylococcus phage 96 | 74 | 1 | DNA | 4 |
| Virus | Staphylococcus phage CNPH82 | 65 | 1 | DNA | 4 |
| Virus | Staphylococcus phage EW | 77 | 1 | DNA | 4 |
| Virus | Staphylococcus phage G1 | 214 | 1 | DNA | 4 |
| Virus | Staphylococcus phage K | 115 | 1 | DNA | 4 |
| Virus | Staphylococcus phage P954 | 69 | 1 | DNA | NA |
| Virus | Staphylococcus phage PH15 | 68 | 1 | DNA | 4 |
| Virus | Staphylococcus phage phi 12 | 49 | 1 | DNA | 4 |
| Virus | Staphylococcus phage phi13 | 49 | 1 | DNA | 4 |
| Virus | Staphylococcus phage phi2958PVL | 60 | NA | NA | NA |
| Virus | Staphylococcus phage phiETA | 66 | 1 | DNA | 4 |
| Virus | Staphylococcus phage phiETA2 | 69 | 1 | DNA | 4 |
| Virus | Staphylococcus phage phiETA3 | 68 | 1 | DNA | 4 |
| Virus | Staphylococcus phage phiMR11 | 67 | 1 | DNA | 4 |
| Virus | Staphylococcus phage phiMR25 | 70 | 1 | DNA | 4 |
| Virus | Staphylococcus phage phiN315 | 65 | 1 | DNA | 4 |
| Virus | Staphylococcus phage phiNM | 64 | 1 | DNA | 4 |
| Virus | Staphylococcus phage phiNM3 | 65 | 1 | DNA | 4 |
| Virus | Staphylococcus phage phiPVL-CN125 | 65 | 1 | DNA | NA |
| Virus | Staphylococcus phage phiPVL108 | 59 | 1 | DNA | 4 |
| Virus | Staphylococcus phage phiSauS-IPLA35 | 62 | 1 | DNA | 4 |
| Virus | Staphylococcus phage phiSauS-IPLA88 | 60 | 1 | DNA | 4 |
| Virus | Staphylococcus phage phiSLT | 61 | 1 | DNA | 4 |
| Virus | Staphylococcus phage PT1028 | 22 | 1 | DNA | NA |
| Virus | Staphylococcus phage PVL | 62 | 1 | DNA | 4 |
| Virus | Staphylococcus phage ROSA | 74 | 1 | DNA | NA |
| Virus | Staphylococcus phage S24-1 | 21 | 1 | DNA | 4 |
| Virus | Staphylococcus phage SAP-2 | 20 | 1 | DNA | 4 |
| Virus | Staphylococcus phage SAP-26 | 63 | 1 | DNA | 4 |
| Virus | Staphylococcus phage SpaA1 | 63 | NA | NA | NA |

| Type | Name | Number of genes | Baltimore class | Nucleic Acid | Monophyletic group |
|---|---|---|---|---|---|
| Virus | Staphylococcus phage TEM123 | 43 | 1 | DNA | 4 |
| Virus | Staphylococcus phage tp310-1 | 59 | NA | NA | NA |
| Virus | Staphylococcus phage tp310-2 | 67 | 1 | DNA | 4 |
| Virus | Staphylococcus phage tp310-3 | 58 | NA | NA | NA |
| Virus | Staphylococcus phage Twort | 195 | 1 | DNA | 4 |
| Virus | Staphylococcus phage vB_SepiS-phiIPLA5 | 66 | 1 | DNA | 4 |
| Virus | Staphylococcus phage vB_SepiS-phiIPLA7 | 59 | 1 | DNA | 4 |
| Virus | Staphylococcus phage X2 | 77 | 1 | DNA | 4 |
| Virus | Staphylococcus prophage phiPV83 | 65 | 1 | DNA | 4 |
| Virus | Starling circovirus | 3 | 2 | DNA | 3 |
| Virus | Steller sea lion vesivirus | 3 | 4 | RNA | 1 |
| Virus | Stenotrophomonas phage IME15 | 45 | 1 | DNA | 4 |
| Virus | Stenotrophomonas phage phiSHP2 | 9 | 2 | DNA | NA |
| Virus | Stenotrophomonas phage S1 | 48 | 1 | DNA | 4 |
| Virus | Strawberry chlorotic fleck-associated virus | 13 | 4 | RNA | 1 |
| Virus | Strawberry latent ringspot virus | 10 | 4 | RNA | 1 |
| Virus | Strawberry latent ringspot virus satellite RNA | 1 | NA | RNA | NA |
| Virus | Strawberry mild yellow edge virus | 6 | 4 | RNA | 1 |
| Virus | Strawberry mottle virus | 10 | 4 | RNA | 1 |
| Virus | Strawberry necrotic shock virus | 5 | 4 | RNA | 1 |
| Virus | Strawberry pallidosis-associated virus | 12 | 4 | RNA | 1 |
| Virus | Strawberry vein banding virus | 6 | 7 | DNA | 2 |
| Virus | Streptocarpus flower break virus | 4 | 4 | RNA | 1 |
| Virus | Streptococcus phage 2972 | 44 | 1 | DNA | 4 |
| Virus | Streptococcus phage 5093 | 48 | 1 | DNA | 4 |
| Virus | Streptococcus phage 7201 | 46 | 1 | DNA | 4 |
| Virus | Streptococcus phage 858 | 46 | 1 | DNA | 4 |
| Virus | Streptococcus phage Abc2 | 48 | 1 | DNA | 4 |
| Virus | Streptococcus phage ALQ13.2 | 44 | 1 | DNA | 4 |
| Virus | Streptococcus phage C1 | 20 | 1 | DNA | 4 |
| Virus | Streptococcus phage Cp-1 | 25 | 1 | DNA | 4 |
| Virus | Streptococcus phage Dp-1 | 72 | 1 | DNA | 4 |
| Virus | Streptococcus phage DT1 | 45 | 1 | DNA | 4 |
| Virus | Streptococcus phage EJ-1 | 73 | 1 | DNA | 4 |
| Virus | Streptococcus phage M102 | 41 | 1 | DNA | 4 |
| Virus | Streptococcus phage MM1 | 53 | 1 | DNA | 4 |
| Virus | Streptococcus phage O1205 | 57 | 1 | DNA | 4 |
| Virus | Streptococcus phage P9 | 53 | 1 | DNA | 4 |
| Virus | Streptococcus phage PH10 | 54 | 1 | DNA | 4 |
| Virus | Streptococcus phage PH15 | 60 | 1 | DNA | 4 |
| Virus | Streptococcus phage phi3396 | 64 | 1 | DNA | 4 |
| Virus | Streptococcus phage phiNJ2 | 55 | 1 | DNA | 4 |
| Virus | Streptococcus phage Sfi11 | 53 | 1 | DNA | 4 |
| Virus | Streptococcus phage Sfi19 | 45 | 1 | DNA | 4 |
| Virus | Streptococcus phage Sfi21 | 50 | 1 | DNA | 4 |
| Virus | Streptococcus phage SM1 | 56 | 1 | DNA | 4 |
| Virus | Streptococcus phage SMP | 48 | 1 | DNA | 4 |
| Virus | Streptococcus phage YMC-2011 | 55 | 1 | DNA | 4 |
| Virus | Streptococcus pyogenes phage 315.1 | 56 | NA | NA | NA |
| Virus | Streptococcus pyogenes phage 315.2 | 60 | 1 | DNA | NA |
| Virus | Streptococcus pyogenes phage 315.3 | 52 | NA | DNA | NA |
| Virus | Streptococcus pyogenes phage 315.4 | 64 | NA | NA | NA |
| Virus | Streptococcus pyogenes phage 315.5 | 55 | NA | NA | NA |
| Virus | Streptococcus pyogenes phage 315.6 | 51 | NA | NA | NA |
| Virus | Streptomyce phage TG1 | 54 | 1 | DNA | 4 |
| Virus | Streptomyces phage mu1/6 | 52 | 1 | DNA | 4 |
| Virus | Streptomyces phage phiBT1 | 55 | 1 | DNA | 4 |
| Virus | Streptomyces phage phiC31 | 54 | 1 | DNA | 4 |
| Virus | Streptomyces phage phiHau3 | 72 | 1 | DNA | 4 |
| Virus | Streptomyces phage phiSASD1 | 43 | 1 | DNA | 4 |
| Virus | Streptomyces phage R4 | 86 | 1 | DNA | 4 |
| Virus | Streptomyces phage SV1 | 55 | 1 | DNA | 4 |
| Virus | Streptomyces phage VWB | 61 | 1 | DNA | 4 |
| Virus | Stretch Lagoon orbivirus | 2 | 3 | RNA | NA |
| Virus | Striped Jack nervous necrosis virus | 3 | 4 | RNA | 1 |
| Virus | Stx1 converting phage | 84 | 1 | DNA | NA |
| Virus | Stx2 converting phage I | 166 | 1 | DNA | 4 |
| Virus | Stx2 converting phage II | 89 | 1 | DNA | 4 |
| Virus | Stx2-converting phage 1717 | 77 | 1 | DNA | 4 |
| Virus | Stx2-converting phage 86 | 81 | 1 | DNA | 4 |
| Virus | Suakwa aphid-borne yellows virus | 6 | 4 | RNA | 1 |
| Virus | Subterranean clover mottle virus | 7 | 4 | RNA | 1 |
| Virus | Subterranean clover stunt virus | 8 | 2 | DNA | NA |
| Virus | Sudan ebolavirus | 8 | 5 | RNA | NA |
| Virus | Sugarcane bacilliform IM virus | 3 | 7 | DNA | 2 |
| Virus | Sugarcane bacilliform Mor virus | 3 | 7 | DNA | 2 |
| Virus | Sugarcane bacilliform virus | 3 | 7 | DNA | 2 |
| Virus | Sugarcane mosaic virus | 12 | 4 | RNA | 1 |
| Virus | Sugarcane streak Egypt virus | 4 | 2 | DNA | 3 |
| Virus | Sugarcane streak mosaic virus | 11 | 4 | RNA | 1 |

| Type | Name | Number of genes | Baltimore class | Nucleic Acid | Monophyletic group |
|---|---|---|---|---|---|
| Virus | Sugarcane streak Reunion virus | 4 | 2 | DNA | 3 |
| Virus | Sugarcane streak virus | 3 | 2 | DNA | 3 |
| Virus | Sugarcane striate mosaic-associated virus | 5 | 4 | RNA | 1 |
| Virus | Sugarcane yellow leaf virus | 9 | 4 | RNA | 1 |
| Virus | Suid herpesvirus 1 | 69 | 1 | DNA | NA |
| Virus | Sulfolobales Mexican rudivirus 1 | 37 | 1 | DNA | 3 |
| Virus | Sulfolobus islandicus filamentous virus | 73 | 1 | DNA | NA |
| Virus | Sulfolobus islandicus rod-shaped virus 1 | 45 | 1 | DNA | NA |
| Virus | Sulfolobus islandicus rod-shaped virus 2 | 54 | 1 | DNA | NA |
| Virus | Sulfolobus spindle-shaped virus 4 | 34 | 1 | DNA | NA |
| Virus | Sulfolobus spindle-shaped virus 5 | 34 | 1 | DNA | NA |
| Virus | Sulfolobus spindle-shaped virus 6 | 33 | 1 | DNA | NA |
| Virus | Sulfolobus spindle-shaped virus 7 | 33 | 1 | DNA | NA |
| Virus | Sulfolobus turreted icosahedral virus | 36 | 1 | DNA | NA |
| Virus | Sulfolobus turreted icosahedral virus 2 | 34 | 1 | DNA | NA |
| Virus | Sulfolobus virus 1 | 32 | 1 | DNA | NA |
| Virus | Sulfolobus virus 2 | 34 | 1 | DNA | NA |
| Virus | Sulfolobus virus Kamchatka 1 | 31 | 1 | DNA | NA |
| Virus | Sulfolobus virus Ragged Hills | 37 | 1 | DNA | NA |
| Virus | Sulfolobus virus STSV1 | 74 | 1 | DNA | NA |
| Virus | Sunflower chlorotic mottle virus | 12 | 4 | RNA | 1 |
| Virus | Sunn hemp leaf distortion virus | 2 | 2 | DNA | 3 |
| Virus | Sus scrofa papillomavirus type 1 | 6 | 1 | DNA | 3 |
| Virus | Sweet clover necrotic mosaic virus | 4 | 4 | RNA | 1 |
| Virus | Sweet potato caulimo-like virus | 4 | 7 | DNA | 2 |
| Virus | Sweet potato chlorotic fleck virus | 6 | 4 | RNA | 1 |
| Virus | Sweet potato chlorotic stunt virus | 14 | 4 | RNA | 1 |
| Virus | Sweet potato feathery mottle virus | 12 | 4 | RNA | 1 |
| Virus | Sweet potato golden vein associated virus | 6 | 2 | DNA | 3 |
| Virus | Sweet potato leaf curl Bengal virus | 6 | 2 | DNA | 3 |
| Virus | Sweet potato leaf curl Canary virus | 6 | 2 | DNA | 3 |
| Virus | Sweet potato leaf curl Georgia virus | 6 | 2 | DNA | 3 |
| Virus | Sweet potato leaf curl Lanzarote virus | 6 | 2 | DNA | 3 |
| Virus | Sweet potato leaf curl South Carolina virus | 6 | 2 | DNA | 3 |
| Virus | Sweet potato leaf curl Spain virus | 6 | 2 | DNA | 3 |
| Virus | Sweet potato leaf curl virus | 6 | 2 | DNA | 3 |
| Virus | Sweet potato mild mottle virus | 12 | 4 | RNA | 1 |
| Virus | Sweet potato vein clearing virus | 9 | 7 | DNA | 2 |
| Virus | Sweet potato virus 2 | 1 | 4 | RNA | 1 |
| Virus | Sweet potato virus C | 11 | 4 | RNA | 1 |
| Virus | Sweet potato virus C-6 | 5 | 4 | RNA | 1 |
| Virus | Sweet potato virus G | 11 | 4 | RNA | 1 |
| Virus | Sweetpotato badnavirus A | 5 | 7 | DNA | 2 |
| Virus | Sweetpotato badnavirus B | 5 | 7 | DNA | 2 |
| Virus | Swine pasivirus 1 | 12 | 4 | RNA | 1 |
| Virus | Swinepox virus | 150 | 1 | DNA | 5 |
| Virus | Switchgrass mosaic virus | 3 | 4 | RNA | 1 |
| Virus | Synechococcus phage P60 | 80 | 1 | DNA | 4 |
| Virus | Synechococcus phage S-CBS1 | 43 | 1 | DNA | 4 |
| Virus | Synechococcus phage S-CBS2 | 102 | 1 | DNA | 4 |
| Virus | Synechococcus phage S-CBS3 | 46 | 1 | DNA | 4 |
| Virus | Synechococcus phage S-CBS4 | 105 | 1 | DNA | 4 |
| Virus | Synechococcus phage S-CRM01 | 297 | 1 | DNA | 4 |
| Virus | Synechococcus phage S-PM2 | 244 | 1 | DNA | 4 |
| Virus | Synechococcus phage S-RSM4 | 237 | 1 | DNA | 4 |
| Virus | Synechococcus phage S-ShM2 | 230 | 1 | DNA | 4 |
| Virus | Synechococcus phage S-SM1 | 234 | 1 | DNA | 4 |
| Virus | Synechococcus phage S-SM2 | 267 | 1 | DNA | 4 |
| Virus | Synechococcus phage S-SSM5 | 225 | 1 | DNA | 4 |
| Virus | Synechococcus phage S-SSM7 | 319 | 1 | DNA | 4 |
| Virus | Synechococcus phage Syn19 | 215 | 1 | DNA | 4 |
| Virus | Synechococcus phage syn9 | 226 | 1 | DNA | 4 |
| Virus | Tacaribe virus | 4 | 5 | RNA | NA |
| Virus | Tai Forest ebolavirus | 9 | 5 | RNA | NA |
| Virus | Tamana bat virus | 14 | 4 | RNA | 1 |
| Virus | Tamiami virus | 4 | 5 | RNA | NA |
| Virus | Tamus red mosaic virus | 5 | 4 | RNA | 1 |
| Virus | Tanapox virus | 156 | 1 | DNA | 5 |
| Virus | Taro bacilliform virus | 3 | 7 | DNA | 2 |
| Virus | Taro vein chlorosis virus | 6 | 5 | RNA | NA |
| Virus | Taterapox virus | 225 | 1 | DNA | 5 |
| Virus | Taura syndrome virus | 2 | 4 | RNA | 1 |
| Virus | Telosma mosaic virus | 12 | 4 | RNA | 1 |
| Virus | Tembusu virus | 1 | 4 | RNA | 1 |
| Virus | Temperate phage phiNIH1.1 | 55 | 1 | DNA | 4 |
| Virus | TGP Carmovirus 1 | 5 | 4 | RNA | 1 |
| Virus | Thalassomonas phage BA3 | 47 | 1 | DNA | 4 |
| Virus | Theilovirus | 14 | 4 | RNA | 1 |
| Virus | Thermoanaerobacterium phage THSA-485A | 57 | 1 | DNA | 4 |
| Virus | Thermococcus prieurii virus 1 | 28 | NA | DNA | NA |

| Type | Name | Number of genes | Baltimore class | Nucleic Acid | Monophyletic group |
|---|---|---|---|---|---|
| Virus | Thermoproteus tenax spherical virus 1 | 38 | 1 | DNA | NA |
| Virus | Thermus phage IN93 | 39 | 1 | DNA | NA |
| Virus | Thermus phage P23-45 | 117 | 1 | DNA | 4 |
| Virus | Thermus phage P23-77 | 37 | 1 | DNA | NA |
| Virus | Thermus phage P74-26 | 116 | 1 | DNA | 4 |
| Virus | Thermus phage phiYS40 | 170 | 1 | DNA | 4 |
| Virus | Thermus phage TMA | 168 | 1 | DNA | 4 |
| Virus | Thetapapillomavirus 1 | 6 | 1 | DNA | 3 |
| Virus | Thielaviopsis basicola mitovirus | 1 | 4 | RNA | 1 |
| Virus | Thogoto virus | 7 | 5 | RNA | NA |
| Virus | Thottapalayam virus | 3 | 5 | RNA | NA |
| Virus | Thrush coronavirus HKU12-600 | 9 | 4 | RNA | NA |
| Virus | Thunberg fritillary virus | 12 | 4 | RNA | 1 |
| Virus | Tianjin totivirus | 2 | 3 | RNA | NA |
| Virus | Tick-borne encephalitis virus | 14 | 4 | RNA | 1 |
| Virus | Tiger puffer nervous necrosis virus | 3 | 4 | RNA | 1 |
| Virus | Tioman virus | 8 | 5 | RNA | NA |
| Virus | Tobacco bushy top virus | 4 | 4 | RNA | 1 |
| Virus | Tobacco curly shoot alphasatellite | 1 | 2 | DNA | 3 |
| Virus | Tobacco curly shoot betasatellite | 1 | 2 | DNA | 3 |
| Virus | Tobacco curly shoot virus | 6 | 2 | DNA | 3 |
| Virus | Tobacco etch virus | 12 | 4 | RNA | 1 |
| Virus | Tobacco leaf chlorosis betasatellite | 1 | 2 | DNA | 3 |
| Virus | Tobacco leaf curl disease associated sequence | 1 | 2 | DNA | NA |
| Virus | Tobacco leaf curl Japan virus | 6 | 2 | DNA | 3 |
| Virus | Tobacco leaf curl Kochi virus | 6 | 2 | DNA | 3 |
| Virus | Tobacco leaf curl PUSA alphasatellite | 1 | NA | NA | NA |
| Virus | Tobacco leaf curl Pusa virus | 6 | 2 | DNA | 3 |
| Virus | Tobacco leaf curl Thailand virus | 7 | 2 | DNA | 3 |
| Virus | Tobacco leaf curl virus-associated DNA beta | 1 | 2 | DNA | NA |
| Virus | Tobacco leaf curl Yunnan virus | 6 | 2 | DNA | 3 |
| Virus | Tobacco leaf curl Yunnan virus associated DNA 1 | 1 | 2 | DNA | NA |
| Virus | Tobacco leaf curl Yunnan virus satellite DNA beta | 1 | 2 | DNA | NA |
| Virus | Tobacco leaf curl Zimbabwe virus | 6 | 2 | DNA | 3 |
| Virus | Tobacco mild green mosaic virus | 4 | 4 | RNA | 1 |
| Virus | Tobacco mosaic virus | 6 | 4 | RNA | 1 |
| Virus | Tobacco necrosis satellite virus | 1 | NA | RNA | NA |
| Virus | Tobacco necrosis virus A | 5 | 4 | RNA | 1 |
| Virus | Tobacco necrosis virus D | 6 | 4 | RNA | 1 |
| Virus | Tobacco rattle virus | 7 | 4 | RNA | 1 |
| Virus | Tobacco ringspot virus | 5 | 4 | RNA | 1 |
| Virus | Tobacco streak virus | 5 | 4 | RNA | 1 |
| Virus | Tobacco vein banding mosaic virus | 12 | 4 | RNA | 1 |
| Virus | Tobacco vein clearing virus | 4 | 7 | DNA | 2 |
| Virus | Tobacco vein distorting virus | 6 | 4 | RNA | 1 |
| Virus | Tobacco vein mottling virus | 12 | 4 | RNA | 1 |
| Virus | Tobacco yellow crinkle virus | 7 | 2 | DNA | 3 |
| Virus | Tobacco yellow dwarf virus | 4 | 2 | DNA | 3 |
| Virus | Tolypocladium cylindrosporum virus 1 | 2 | 3 | RNA | NA |
| Virus | Tomato aspermy virus | 5 | 4 | RNA | 1 |
| Virus | Tomato begomovirus satellite DNA beta | 1 | 2 | DNA | 3 |
| Virus | Tomato black ring virus | 8 | 4 | RNA | 1 |
| Virus | Tomato black ring virus satellite RNA | 1 | NA | RNA | NA |
| Virus | Tomato bushy stunt virus | 5 | 4 | RNA | 1 |
| Virus | Tomato chino La Paz virus | 5 | 2 | DNA | 3 |
| Virus | Tomato chlorosis virus | 13 | 4 | RNA | 1 |
| Virus | Tomato chlorotic mottle virus | 7 | 2 | DNA | 3 |
| Virus | Tomato chocolate spot virus | 6 | 4 | RNA | NA |
| Virus | Tomato common mosaic virus | 7 | 2 | DNA | 3 |
| Virus | Tomato curly stunt virus | 5 | 2 | DNA | 3 |
| Virus | Tomato dwarf leaf virus | 7 | 2 | DNA | 3 |
| Virus | Tomato golden mosaic virus | 7 | 2 | DNA | 3 |
| Virus | Tomato golden mottle virus | 7 | 2 | DNA | 3 |
| Virus | Tomato infectious chlorosis virus | 12 | 4 | RNA | 1 |
| Virus | Tomato leaf curl Arusha virus | 6 | 2 | DNA | 3 |
| Virus | Tomato leaf curl Bangalore virus | 6 | 2 | DNA | 3 |
| Virus | Tomato leaf curl Bangladesh betasatellite | 1 | 2 | DNA | 3 |
| Virus | Tomato leaf curl Bangladesh virus | 6 | 2 | DNA | 3 |
| Virus | Tomato leaf curl Cameroon alphasatellite | 1 | NA | NA | NA |
| Virus | Tomato leaf curl Cameroon virus | 9 | 2 | DNA | 3 |
| Virus | Tomato leaf curl Cebu virus | 6 | 2 | DNA | 3 |
| Virus | Tomato leaf curl China betasatellite | 2 | 2 | DNA | 3 |
| Virus | Tomato leaf curl China virus | 6 | 2 | DNA | 3 |
| Virus | Tomato leaf curl Cotabato virus | 6 | 2 | DNA | 3 |
| Virus | Tomato leaf curl Ghana virus | 6 | 2 | DNA | 3 |
| Virus | Tomato leaf curl Guangdong virus | 6 | 2 | DNA | 3 |
| Virus | Tomato leaf curl Guangxi virus | 6 | 2 | DNA | 3 |
| Virus | Tomato leaf curl Gujarat virus | 8 | 2 | DNA | 3 |
| Virus | Tomato leaf curl Hainan virus | 6 | 2 | DNA | 3 |
| Virus | Tomato leaf curl Hajipur betasatellite | 1 | 2 | DNA | 3 |

| Type | Name | Number of genes | Baltimore class | Nucleic Acid | Monophyletic group |
|---|---|---|---|---|---|
| Virus | Tomato leaf curl Hanoi virus | 6 | 2 | DNA | 3 |
| Virus | Tomato leaf curl Hsinchu virus | 6 | 2 | DNA | 3 |
| Virus | Tomato leaf curl Iran virus | 6 | 2 | DNA | 3 |
| Virus | Tomato leaf curl Java virus | 6 | 2 | DNA | 3 |
| Virus | Tomato leaf curl Joydebpur virus | 6 | 2 | DNA | 3 |
| Virus | Tomato leaf curl Joydebpur virus satellite DNA beta | 1 | 2 | DNA | 3 |
| Virus | Tomato leaf curl Karnataka virus | 6 | 2 | DNA | 3 |
| Virus | Tomato leaf curl Karnataka virus-associated DNA beta | 1 | 2 | DNA | 3 |
| Virus | Tomato leaf curl Kerala virus | 6 | 2 | DNA | 3 |
| Virus | Tomato leaf curl Kumasi virus | 6 | 2 | DNA | 3 |
| Virus | Tomato leaf curl Laos virus | 6 | 2 | DNA | 3 |
| Virus | Tomato leaf curl Malaysia virus | 6 | 2 | DNA | 3 |
| Virus | Tomato leaf curl Mali virus | 6 | 2 | DNA | 3 |
| Virus | Tomato leaf curl Mayotte virus | 6 | 2 | DNA | 3 |
| Virus | Tomato leaf curl Mindanao virus | 6 | 2 | DNA | 3 |
| Virus | Tomato leaf curl New Delhi betasatellite | 1 | 2 | DNA | NA |
| Virus | Tomato leaf curl New Delhi virus | 10 | 2 | DNA | 3 |
| Virus | Tomato leaf curl Oman virus | 6 | 2 | DNA | 3 |
| Virus | Tomato leaf curl Pakistan alphasatellite | 1 | 2 | DNA | NA |
| Virus | Tomato leaf curl Pakistan virus | 6 | 2 | DNA | 3 |
| Virus | Tomato leaf curl Palampur virus | 9 | 2 | DNA | 3 |
| Virus | Tomato leaf curl Patna betasatellite | 1 | 2 | DNA | 3 |
| Virus | Tomato leaf curl Patna virus | 7 | 2 | DNA | 3 |
| Virus | Tomato leaf curl Philippine betasatellite | 1 | 2 | DNA | NA |
| Virus | Tomato leaf curl Philippines virus | 6 | 2 | DNA | 3 |
| Virus | Tomato leaf curl Pune virus | 6 | 2 | DNA | 3 |
| Virus | Tomato leaf curl Ranchi virus | 7 | 2 | DNA | 3 |
| Virus | Tomato leaf curl Seychelles virus | 3 | 2 | DNA | 3 |
| Virus | Tomato leaf curl Sinaloa virus | 7 | 2 | DNA | 3 |
| Virus | Tomato leaf curl Sri Lanka virus | 6 | 2 | DNA | 3 |
| Virus | Tomato leaf curl Sudan virus | 6 | 2 | DNA | 3 |
| Virus | Tomato leaf curl Sulawesi virus | 6 | 2 | DNA | 3 |
| Virus | Tomato leaf curl Taiwan virus | 6 | 2 | DNA | 3 |
| Virus | Tomato leaf curl Vietnam virus | 6 | 2 | DNA | 3 |
| Virus | Tomato leaf curl virus | 6 | 2 | DNA | 3 |
| Virus | Tomato leaf curl virus-associated DNA beta | 1 | 2 | DNA | 3 |
| Virus | Tomato leaf curl virus-Pune-associated DNA beta | 1 | 2 | DNA | 3 |
| Virus | Tomato leaf curl Yemen betasatellite | 1 | 2 | DNA | 3 |
| Virus | Tomato leaf deformation virus | 11 | 2 | DNA | 3 |
| Virus | Tomato marchitez virus | 8 | 4 | RNA | 1 |
| Virus | Tomato mild mosaic virus | 7 | 2 | DNA | 3 |
| Virus | Tomato mosaic Havana virus | 7 | 2 | DNA | 3 |
| Virus | Tomato mosaic leaf curl virus | 7 | 2 | DNA | 3 |
| Virus | Tomato mosaic virus | 4 | 4 | RNA | 1 |
| Virus | Tomato mottle leaf curl Zulia virus | 4 | 2 | DNA | 3 |
| Virus | Tomato mottle Taino virus | 6 | 2 | DNA | 3 |
| Virus | Tomato mottle virus | 6 | 2 | DNA | 3 |
| Virus | Tomato necrotic stunt virus | 12 | 4 | RNA | 1 |
| Virus | Tomato pseudo-curly top virus | 6 | 2 | DNA | 3 |
| Virus | Tomato ringspot virus | 10 | 4 | RNA | 1 |
| Virus | Tomato rugose mosaic virus | 7 | 2 | DNA | 3 |
| Virus | Tomato severe leaf curl virus | 4 | 2 | DNA | 3 |
| Virus | Tomato severe rugose virus | 7 | 2 | DNA | 3 |
| Virus | Tomato spotted wilt virus | 5 | 5 | RNA | NA |
| Virus | Tomato torrado virus | 3 | 4 | RNA | 1 |
| Virus | Tomato yellow leaf curl China alphasatellite | 1 | 2 | DNA | NA |
| Virus | Tomato yellow leaf curl China virus | 6 | 2 | DNA | 3 |
| Virus | Tomato yellow leaf curl Guangdong virus | 6 | 2 | DNA | 3 |
| Virus | Tomato yellow leaf curl Kanchanaburi virus | 8 | 2 | DNA | 3 |
| Virus | Tomato yellow leaf curl Malaga virus | 6 | 2 | DNA | 3 |
| Virus | Tomato yellow leaf curl Mali virus-associated DNA beta | 1 | 2 | DNA | 3 |
| Virus | Tomato yellow leaf curl Sardinia virus | 6 | 2 | DNA | 3 |
| Virus | Tomato yellow leaf curl Thailand betasatellite | 2 | 2 | DNA | 3 |
| Virus | Tomato yellow leaf curl Thailand virus | 10 | 2 | DNA | 3 |
| Virus | Tomato yellow leaf curl Thailand virus associated DNA 1 | 1 | 2 | DNA | NA |
| Virus | Tomato yellow leaf curl Vietnam virus | 6 | 2 | DNA | 3 |
| Virus | Tomato yellow leaf curl Vietnam virus satellite DNA beta | 1 | 2 | DNA | 3 |
| Virus | Tomato yellow leaf curl virus | 6 | 2 | DNA | 3 |
| Virus | Tomato yellow leaf curl virus-associated DNA beta | 1 | 2 | DNA | NA |
| Virus | Tomato yellow leaf distortion virus | 7 | 2 | DNA | 3 |
| Virus | Tomato yellow margin leaf curl virus | 7 | 2 | DNA | 3 |
| Virus | Tomato yellow spot virus | 6 | 2 | DNA | 3 |
| Virus | Tomato yellow vein streak virus | 7 | 2 | DNA | 3 |
| Virus | Tomato zonate spot virus | 5 | 5 | RNA | NA |
| Virus | Torque teno canis virus | 3 | 2 | DNA | NA |
| Virus | Torque teno douroucouli virus | 3 | 2 | DNA | NA |
| Virus | Torque teno felis virus | 3 | 2 | DNA | NA |
| Virus | Torque teno midi virus 1 | 4 | 2 | DNA | NA |
| Virus | Torque teno midi virus 2 | 4 | 2 | DNA | NA |
| Virus | Torque teno mini virus 1 | 3 | 2 | DNA | NA |

| Type | Name | Number of genes | Baltimore class | Nucleic Acid | Monophyletic group |
|---|---|---|---|---|---|
| Virus | Torque teno mini virus 2 | 3 | 2 | DNA | NA |
| Virus | Torque teno mini virus 3 | 3 | 2 | DNA | NA |
| Virus | Torque teno mini virus 4 | 2 | 2 | DNA | NA |
| Virus | Torque teno mini virus 5 | 3 | 2 | DNA | NA |
| Virus | Torque teno mini virus 6 | 3 | 2 | DNA | NA |
| Virus | Torque teno mini virus 7 | 2 | 2 | DNA | NA |
| Virus | Torque teno mini virus 8 | 2 | 2 | DNA | NA |
| Virus | Torque teno mini virus 9 | 3 | 2 | DNA | NA |
| Virus | Torque teno sus virus 1a | 3 | 2 | DNA | NA |
| Virus | Torque teno sus virus 1b | 3 | 2 | DNA | NA |
| Virus | Torque teno tamarin virus | 4 | 2 | DNA | NA |
| Virus | Torque teno virus 1 | 3 | 2 | DNA | NA |
| Virus | Torque teno virus 10 | 4 | 2 | DNA | NA |
| Virus | Torque teno virus 12 | 4 | 2 | DNA | NA |
| Virus | Torque teno virus 14 | 2 | 2 | DNA | NA |
| Virus | Torque teno virus 15 | 2 | 2 | DNA | NA |
| Virus | Torque teno virus 16 | 2 | 2 | DNA | NA |
| Virus | Torque teno virus 19 | 2 | 2 | DNA | NA |
| Virus | Torque teno virus 2 | 2 | 2 | DNA | NA |
| Virus | Torque teno virus 25 | 3 | 2 | DNA | NA |
| Virus | Torque teno virus 26 | 3 | 2 | DNA | NA |
| Virus | Torque teno virus 27 | 4 | 2 | DNA | NA |
| Virus | Torque teno virus 28 | 4 | 2 | DNA | NA |
| Virus | Torque teno virus 3 | 6 | 2 | DNA | NA |
| Virus | Torque teno virus 4 | 2 | 2 | DNA | NA |
| Virus | Torque teno virus 6 | 4 | 2 | DNA | NA |
| Virus | Torque teno virus 7 | 3 | 2 | DNA | NA |
| Virus | Torque teno virus 8 | 4 | 2 | DNA | NA |
| Virus | Tree shrew adenovirus A | 36 | 1 | DNA | NA |
| Virus | Triatoma virus | 2 | 4 | RNA | 1 |
| Virus | Trichechus manatus latirostris papillomavirus 2 | 7 | 1 | DNA | 3 |
| Virus | Trichechus manatus papillomavirus 1 | 7 | 1 | DNA | 3 |
| Virus | Trichodysplasia spinulosa-associated polyomavirus | 5 | 1 | DNA | 3 |
| Virus | Trichomonas vaginalis virus | 2 | 3 | RNA | NA |
| Virus | Trichomonas vaginalis virus 3 | 2 | 3 | RNA | NA |
| Virus | Trichomonas vaginalis virus II | 2 | 3 | RNA | NA |
| Virus | Trichoplusia ni ascovirus 2c | 164 | 1 | DNA | 5 |
| Virus | Trichoplusia ni cypovirus 15 | 1 | 3 | RNA | NA |
| Virus | Trichoplusia ni SNPV | 145 | 1 | DNA | NA |
| Virus | Triticum mosaic virus | 11 | 4 | RNA | 1 |
| Virus | Tsukamurella phage TPA2 | 78 | 1 | DNA | 4 |
| Virus | Tuber aestivum endornavirus | 1 | 3 | RNA | NA |
| Virus | Tuber aestivum mitovirus | 1 | 4 | RNA | 1 |
| Virus | Tula virus | 4 | 5 | RNA | NA |
| Virus | Tulare apple mosaic virus | 5 | 4 | RNA | 1 |
| Virus | Tulip virus X | 5 | 4 | RNA | 1 |
| Virus | Tupaia paramyxovirus | 8 | 5 | RNA | NA |
| Virus | Tupaia virus | 7 | 5 | RNA | NA |
| Virus | Tupaiid herpesvirus 1 | 158 | 1 | DNA | NA |
| Virus | Turdivirus 1 | 12 | 4 | RNA | 1 |
| Virus | Turdivirus 2 | 12 | 4 | RNA | 1 |
| Virus | Turdivirus 3 | 12 | 4 | RNA | 1 |
| Virus | Turkey adenovirus 1 | 37 | 1 | DNA | NA |
| Virus | Turkey adenovirus A | 47 | 1 | DNA | NA |
| Virus | Turkey astrovirus | 3 | 4 | RNA | 1 |
| Virus | Turkey astrovirus 2 | 3 | 4 | RNA | 1 |
| Virus | Turkey coronavirus | 26 | 4 | RNA | NA |
| Virus | Turkey gallivirus | 1 | 4 | RNA | 1 |
| Virus | Turnip crinkle virus | 4 | 4 | RNA | 1 |
| Virus | Turnip curly top virus | 6 | 2 | DNA | 3 |
| Virus | Turnip mosaic virus | 12 | 4 | RNA | 1 |
| Virus | Turnip ringspot virus | 2 | 4 | RNA | 1 |
| Virus | Turnip rosette virus | 8 | 4 | RNA | 1 |
| Virus | Turnip vein-clearing virus | 4 | 4 | RNA | 1 |
| Virus | Turnip yellow mosaic virus | 5 | 4 | RNA | 1 |
| Virus | Turnip yellows virus | 6 | 4 | RNA | 1 |
| Virus | Tursiops truncatus papillomavirus 1 | 7 | 1 | DNA | 3 |
| Virus | Tursiops truncatus papillomavirus 2 | 7 | 1 | DNA | 3 |
| Virus | UR2 sarcoma virus | 2 | 6 | RNA | 2 |
| Virus | Urochloa streak virus | 3 | 2 | DNA | 3 |
| Virus | Ursus maritimus papillomavirus 1 | 5 | 1 | DNA | 3 |
| Virus | Ustilago maydis virus H1 | 1 | 3 | RNA | NA |
| Virus | Usutu virus | 16 | 4 | RNA | 1 |
| Virus | Uukuniemi virus | 6 | 5 | RNA | NA |
| Virus | Vaccinia virus | 223 | 1 | DNA | 5 |
| Virus | Vallota speciosa virus | 11 | 4 | RNA | 1 |
| Virus | Valsa ceratosperma hypovirus 1 | 1 | 3 | RNA | NA |
| Virus | Variola virus | 197 | 1 | DNA | 5 |
| Virus | Varroa destructor virus-1 | 1 | 4 | RNA | 1 |
| Virus | Velvet bean severe mosaic virus | 9 | 2 | DNA | 3 |

| Type | Name | Number of genes | Baltimore class | Nucleic Acid | Monophyletic group |
|---|---|---|---|---|---|
| Virus | Velvet tobacco mottle virus | 4 | 4 | RNA | 1 |
| Virus | Venezuelan equine encephalitis virus | 14 | 4 | RNA | 1 |
| Virus | Verbena virus Y | 12 | 4 | RNA | 1 |
| Virus | Vernonia yellow vein betasatellite | 1 | 2 | DNA | NA |
| Virus | Vernonia yellow vein Fujian virus alphasatellite | 1 | NA | NA | NA |
| Virus | Vernonia yellow vein Fujian virus betasatellite | 1 | 2 | DNA | 3 |
| Virus | Vernonia yellow vein virus | 7 | 2 | DNA | 3 |
| Virus | Vesicular exanthema of swine virus | 12 | 4 | RNA | 1 |
| Virus | Vesicular stomatitis Indiana virus | 7 | 5 | RNA | NA |
| Virus | Vibrio phage CTX | 13 | 2 | DNA | NA |
| Virus | Vibrio phage fs1 | 15 | 2 | DNA | NA |
| Virus | Vibrio phage fs2 | 9 | 2 | DNA | NA |
| Virus | Vibrio phage ICP1 | 232 | 1 | DNA | 4 |
| Virus | Vibrio phage ICP2 | 72 | 1 | DNA | 4 |
| Virus | Vibrio phage ICP3 | 54 | 1 | DNA | 4 |
| Virus | Vibrio phage K139 | 44 | 1 | DNA | 4 |
| Virus | Vibrio phage kappa | 45 | 1 | DNA | 4 |
| Virus | Vibrio phage KSF-1phi | 12 | 2 | DNA | NA |
| Virus | Vibrio phage KVP40 | 381 | 1 | DNA | 4 |
| Virus | Vibrio phage N4 | 47 | 1 | DNA | 4 |
| Virus | Vibrio phage SIO-2 | 115 | 1 | DNA | 4 |
| Virus | Vibrio phage VCY-phi | 11 | 2 | DNA | 3 |
| Virus | Vibrio phage VEJphi | 11 | 2 | DNA | NA |
| Virus | Vibrio phage Vf12 | 7 | 2 | DNA | NA |
| Virus | Vibrio phage Vf33 | 7 | 2 | DNA | NA |
| Virus | Vibrio phage VfO3K6 | 10 | 2 | DNA | NA |
| Virus | Vibrio phage VfO4K68 | 8 | 2 | DNA | NA |
| Virus | Vibrio phage VGJphi | 13 | 2 | DNA | NA |
| Virus | Vibrio phage VHML | 57 | 1 | DNA | 4 |
| Virus | Vibrio phage VP2 | 47 | 1 | DNA | 4 |
| Virus | Vibrio phage VP5 | 48 | 1 | DNA | 4 |
| Virus | Vibrio phage VP882 | 71 | 1 | DNA | 4 |
| Virus | Vibrio phage VP93 | 44 | 1 | DNA | 4 |
| Virus | Vibrio phage VpV262 | 67 | 1 | DNA | 4 |
| Virus | Vibrio phage VSK | 14 | 2 | DNA | NA |
| Virus | Vibriophage VP4 | 31 | 1 | DNA | 4 |
| Virus | Vicia cryptic virus | 2 | 3 | RNA | 1 |
| Virus | Vicia faba endornavirus | 1 | 3 | RNA | NA |
| Virus | Viral hemorrhagic septicemia virus Fil3 | 6 | 5 | RNA | NA |
| Virus | Visna/Maedi virus | 6 | 6 | RNA | 2 |
| Virus | Walleye dermal sarcoma virus | 6 | 6 | RNA | 2 |
| Virus | Walrus calicivirus | 12 | 4 | RNA | 1 |
| Virus | Wasabi mottle virus | 4 | 4 | RNA | 1 |
| Virus | Watermelon chlorotic stunt virus | 8 | 2 | DNA | 3 |
| Virus | Watermelon mosaic virus | 12 | 4 | RNA | 1 |
| Virus | Watermelon silver mottle virus | 5 | 5 | RNA | NA |
| Virus | Weissella phage phiYS61 | 48 | 1 | DNA | 4 |
| Virus | Wesselsbron virus | 1 | 4 | RNA | 1 |
| Virus | West Nile virus | 29 | 4 | RNA | 1 |
| Virus | Western equine encephalomyelitis virus | 14 | 4 | RNA | 1 |
| Virus | Whataroa virus | 4 | 4 | RNA | 1 |
| Virus | Wheat dwarf India virus | 4 | 2 | DNA | 3 |
| Virus | Wheat dwarf virus | 4 | 2 | DNA | 3 |
| Virus | Wheat eqlid mosaic virus | 11 | 4 | RNA | 1 |
| Virus | Wheat streak mosaic virus | 12 | 4 | RNA | 1 |
| Virus | Wheat yellow dwarf virus-GPV | 6 | 4 | RNA | 1 |
| Virus | Wheat yellow mosaic virus | 13 | 4 | RNA | 1 |
| Virus | White ash mosaic virus | 5 | 4 | RNA | NA |
| Virus | White bream virus | 5 | 4 | RNA | NA |
| Virus | White clover cryptic virus 1 | 2 | 3 | RNA | 1 |
| Virus | White clover mosaic virus | 5 | 4 | RNA | 1 |
| Virus | White-eye coronavirus HKU16 | 8 | 4 | RNA | NA |
| Virus | Whitewater Arroyo virus | 4 | 5 | RNA | NA |
| Virus | Wigeon coronavirus HKU20 | 10 | 4 | RNA | NA |
| Virus | Wild potato mosaic virus | 11 | 4 | RNA | 1 |
| Virus | Wild tomato mosaic virus | 12 | 4 | RNA | 1 |
| Virus | Wiseana iridescent virus | 193 | 1 | DNA | 5 |
| Virus | Wissadula golden mosaic virus | 7 | 2 | DNA | 3 |
| Virus | Wisteria vein mosaic virus | 12 | 4 | RNA | 1 |
| Virus | Wongabel virus | 10 | 5 | RNA | NA |
| Virus | Woodchuck hepatitis virus | 5 | 7 | DNA | 2 |
| Virus | Woolly monkey sarcoma virus | 10 | 6 | RNA | 2 |
| Virus | WU Polyomavirus | 5 | 1 | DNA | 3 |
| Virus | Xanthomonas phage Cf1c | 9 | 2 | DNA | NA |
| Virus | Xanthomonas phage OP1 | 59 | 1 | DNA | 4 |
| Virus | Xanthomonas phage OP2 | 62 | 1 | DNA | 4 |
| Virus | Xanthomonas phage phiL7 | 58 | 1 | DNA | 4 |
| Virus | Xanthomonas phage vB_XveM_DIBBI | 81 | 1 | DNA | 4 |
| Virus | Xanthomonas phage Xop411 | 58 | 1 | DNA | 4 |
| Virus | Xanthomonas phage Xp10 | 60 | 1 | DNA | 4 |

| Type | Name | Number of genes | Baltimore class | Nucleic Acid | Monophyletic group |
|---|---|---|---|---|---|
| Virus | Xanthomonas phage Xp15 | 84 | 1 | DNA | NA |
| Virus | Xenopus laevis endogenous retrovirus Xen1 | 2 | 6 | RNA | 2 |
| Virus | Xestia c-nigrum granulovirus | 181 | 1 | DNA | NA |
| Virus | Xipapillomavirus 1 | 8 | 1 | DNA | 3 |
| Virus | Xylella phage Xfas53 | 45 | 1 | DNA | 4 |
| Virus | Y73 sarcoma virus | 1 | 6 | RNA | 2 |
| Virus | Yaba monkey tumor virus | 140 | 1 | DNA | 5 |
| Virus | Yaba-like disease virus | 152 | 1 | DNA | 5 |
| Virus | Yam bean mosaic virus | 12 | 4 | RNA | 1 |
| Virus | Yam mild mosaic virus | 12 | 4 | RNA | 1 |
| Virus | Yam mosaic virus | 12 | 4 | RNA | 1 |
| Virus | Yellow fever virus | 14 | 4 | RNA | 1 |
| Virus | Yellowtail ascites virus | 3 | 3 | RNA | NA |
| Virus | Yersinia phage Berlin | 45 | 1 | DNA | 4 |
| Virus | Yersinia phage L-413C | 40 | 1 | DNA | 4 |
| Virus | Yersinia phage phiA1122 | 50 | 1 | DNA | 4 |
| Virus | Yersinia phage phiR1-37 | 367 | 1 | DNA | 4 |
| Virus | Yersinia phage phiYeO3-12 | 59 | 1 | DNA | 4 |
| Virus | Yersinia phage PY54 | 67 | 1 | DNA | 4 |
| Virus | Yersinia phage Yepe2 | 46 | 1 | DNA | 4 |
| Virus | Yoka poxvirus | 186 | 1 | DNA | 5 |
| Virus | Yokose virus | 1 | 4 | RNA | 1 |
| Virus | Youcai mosaic virus | 3 | 4 | RNA | 1 |
| Virus | Yunnan orbivirus | 11 | 3 | RNA | NA |
| Virus | Zalophus californianus papillomavirus 1 | 7 | 1 | DNA | 3 |
| Virus | Zantedeschia mild mosaic virus | 12 | 4 | RNA | 1 |
| Virus | Zika virus | 1 | 4 | RNA | 1 |
| Virus | Zinnia leaf curl disease associated sequence | 1 | 2 | DNA | NA |
| Virus | Zinnia leaf curl virus-associated DNA beta | 1 | 2 | DNA | NA |
| Virus | Zucchini green mottle mosaic virus | 4 | 4 | RNA | 1 |
| Virus | Zucchini yellow mosaic virus | 12 | 4 | RNA | 1 |
| Virus | Zygocactus virus X | 5 | 4 | RNA | 1 |
| Virus | Zygosaccharomyces bailii virus Z | 2 | 3 | RNA | NA |
