# Supplementary figures and images for: Extensive Gene Remodeling in the Viral World: New Evidence for Nongradual Evolution in the Mobilome Network
Source: Genome Biol Evol. 2014 Aug 7;6(9):2195–205. doi: 10.1093/gbe/evu168 (PMC4202312; doi:10.1093/gbe/evu168)

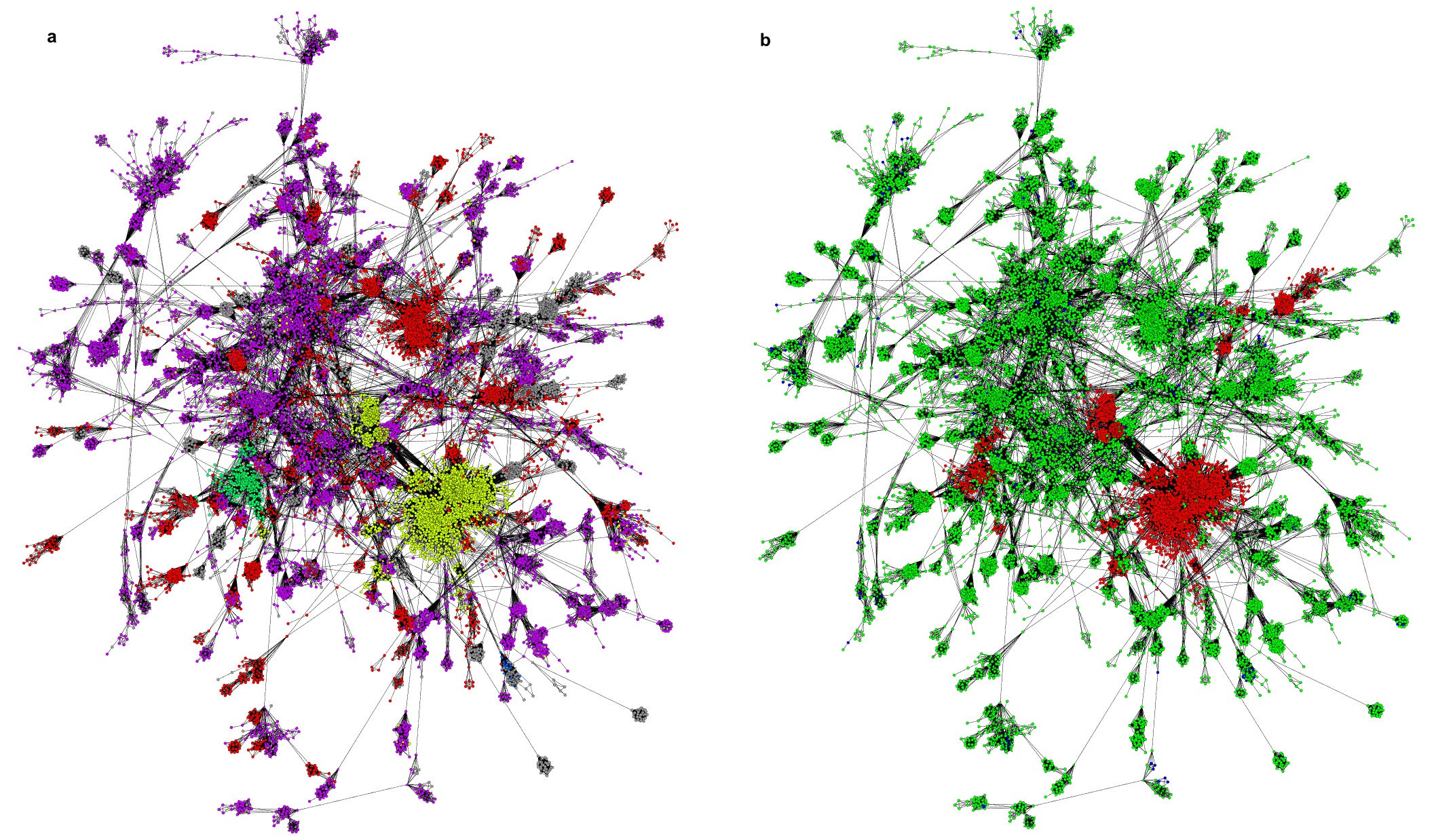

Supplement: Supplementary Data [file supp_evu168_bapteste_ED_fig2.jpg]
