# Supplementary material for: Extensive Gene Remodeling in the Viral World: New Evidence for Nongradual Evolution in the Mobilome Network
Source: Genome Biol Evol. 2014 Aug 7;6(9):2195–205. doi: 10.1093/gbe/evu168 (PMC4202312; doi:10.1093/gbe/evu168)
Supplement: Supplementary Data [file supp_evu168_SI_gbe_revised_notrackchanges.docx]

Extensive gene remodeling in the viral world : new evidence for non-gradual evolution in the mobilome network

PA Jachiet, P Colson, P Lopez, E Bapteste

**Supplementary Information**

Figures S1-S6

Tables S1-S2

*Table S3 is 53 pages long and submitted separately.*

Fig. S1: **Distributions of hits and sequences lengths**

Frequency histograms represents the lengths of : (i) BLAST hits between any pair of sequences in the network (purple); (ii) BLAST hits between each unique pair of composite sequences and component sequences (red); (iii) non annotated (in a COG or KOG search with an E-value < 10-5) viral sequences (orange); (iv) annotated viral sequences (blue); (v) annotated viral sequences belonging to COG or KOG categories enriched in viruses with respect to cellular organisms (black), (vi) annotated viral sequences belonging to COG or KOG categories non enriched in viruses with respect to cellular organisms (green). All distributions were truncated at length >= 1000 amino acids. Corresponding statistics for the hits lengths distributions are: for (i), a minimal/median/maximal length of 19/238/7180 aa, respectively; for (ii) a minimal/median/maximal length of 25/288/5793 aa, respectively. Corresponding statistics for the sequences lengths distributions are: for (iii) a minimal/median/maximal length of 7/144/7312 aa, respectively; for (iv) a minimal/median/maximal length of 28/342/7182 aa, respectively; for (v) a minimal/median/maximal length of 43/346,5/7182 aa, respectively; for (vi) a minimal/median/maximal length of 28/311/4815 aa, respectively. Distributions for (i) and (ii), for (iii) and (iv) and for (v) and (vi) are significantly different according to two-sided KS tests (p-values < 2,2 e^-16^).


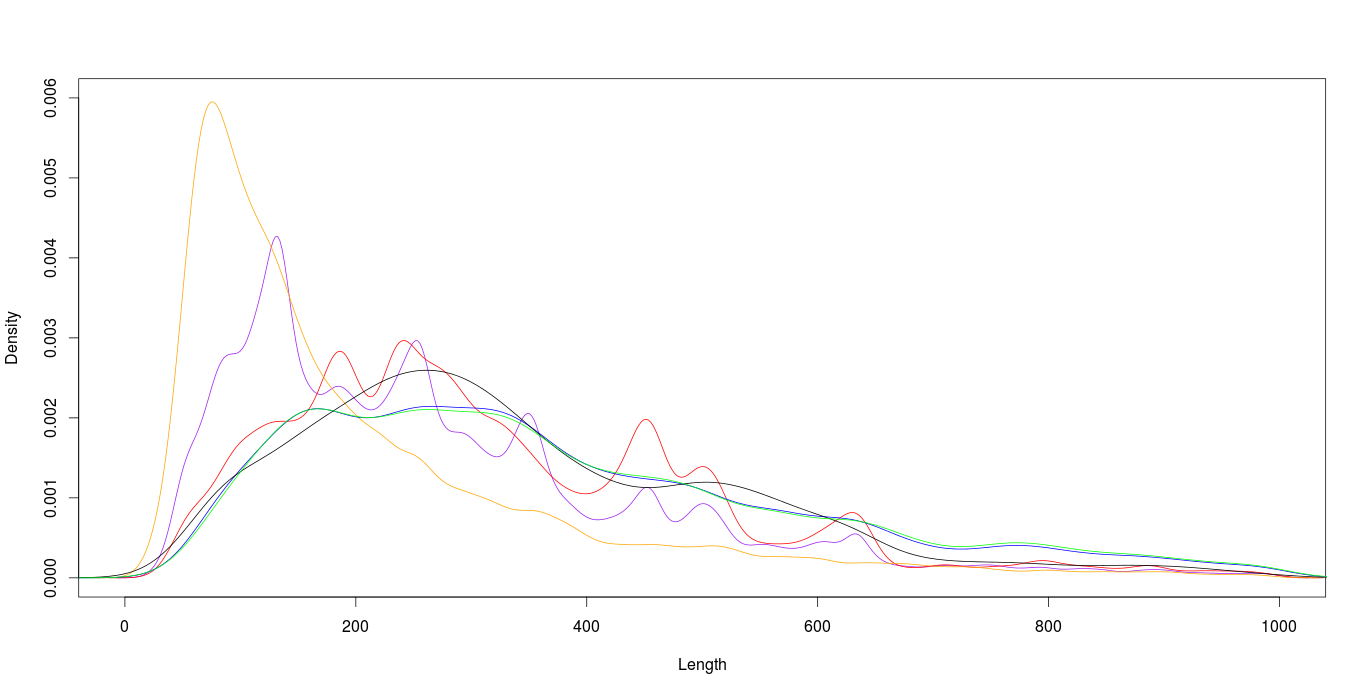


Fig. S2: **Distributions of E-values for hits in the network**

Frequency histograms showing the distribution of -log10(E-value) for (i) all hits present in the network (green), and for (ii) hits between composite and component sequences (blue). Values are thresholded at 150. Distributions are significantly different according to a two sided KS test (p-value < 2,2 e^-16^).


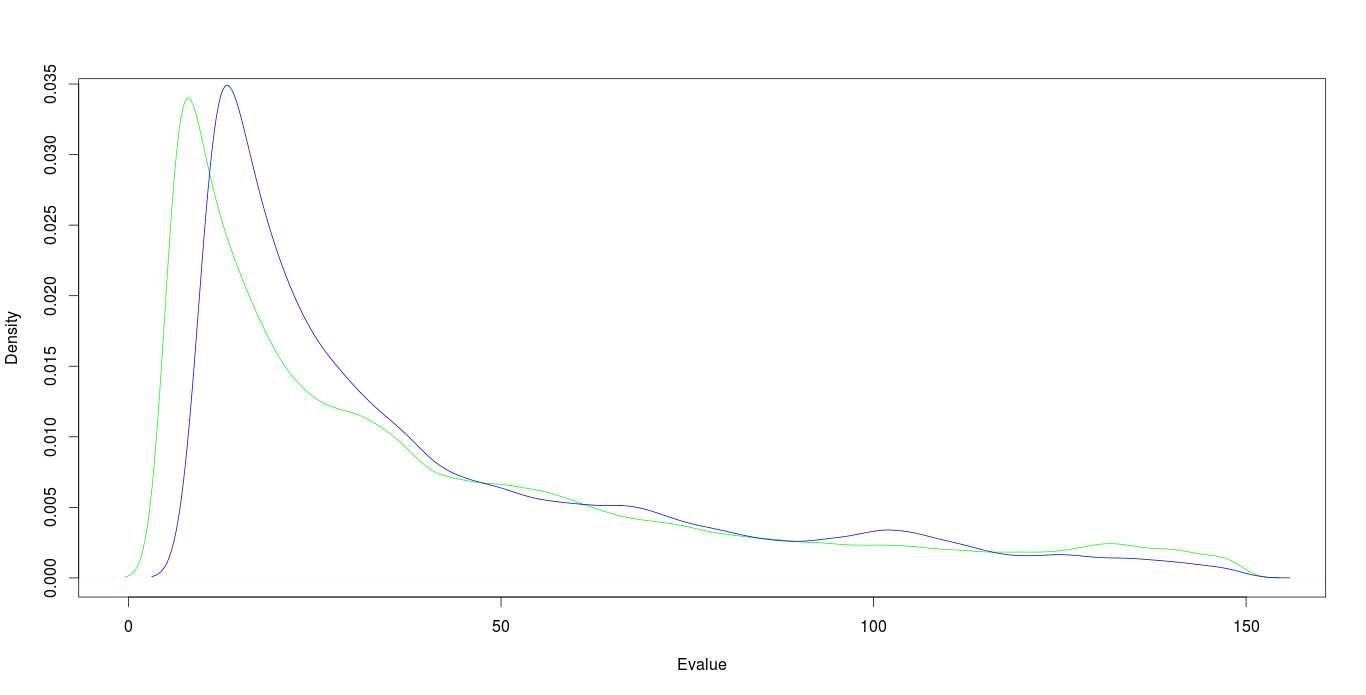


Fig. S3: **Functional distribution of cellular, viral, viral composite genes and ‘safest’ viral composite genes**


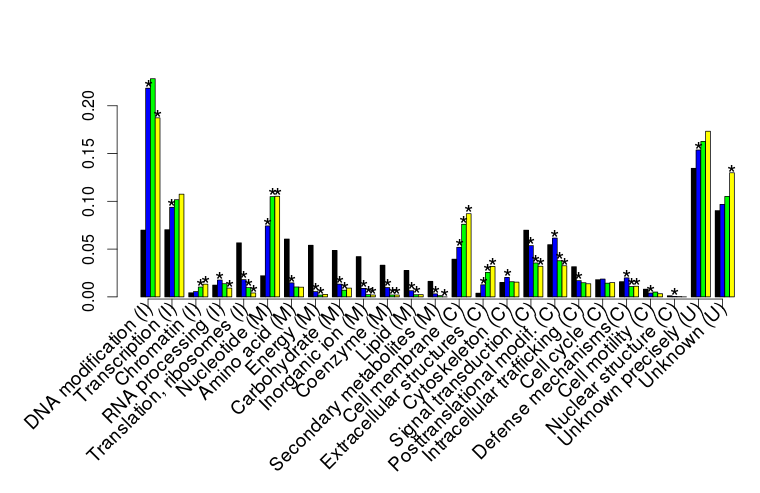
A stringent subset of composite genes was defined as indicated in the main text, to avoid artifactual composite arising from annotations errors. The proportion of genes in each functional category was plotted for the reference “cellular” dataset (black), the viral dataset (blue), the viral composite subset (green), and the ‘safest’ viral composite genes (yellow). Genes assigned to multiple categories were redistributed evenly into each of the concerned categories. Unannotated genes were not considered. Functional categories significantly depleted or enriched in the viral dataset with respect to the cellular dataset (respectively in viral and ‘safest’ viral composite genes with respect to viral dataset) were highlighted with a star (Fisher test, overall significance level of 0.05). Broad functional categories are indicated with a letter, (I): Information storage and processing, (C): Cellular processes and signaling, (M): Metabolism, (U): Poorly characterized.

Fig. S4: **Giant connected component of the viral gene similarity network**

Nodes are individual sequences, edges represent similarity of BLAST E-value < 1e-5. **(A)** Node colors correspond to major monophyletic classes of viruses (1: yellow, 2: green, 3: blue, 4: purple, 5: red). **(B)** Node colors correspond to types of nucleic acids (DNA: green, RNA: red).


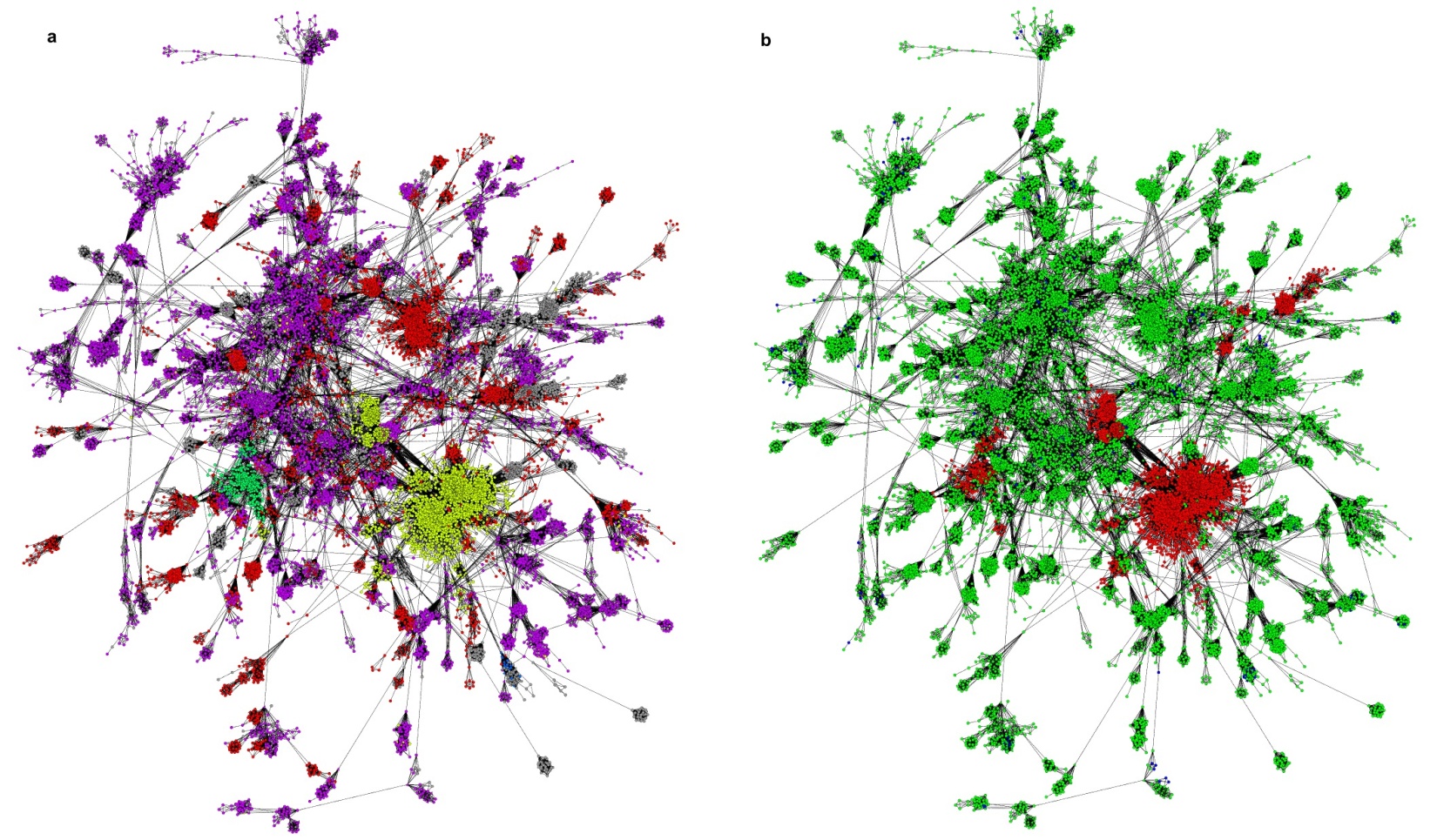


Fig. S5: **Gene similarity across viral classes outside of the giant connected component**Regions of the sequence similarity graph, outside of the giant connected component, presenting similarity between genes carried from virus of different Baltimore classes. Colors correspond to Baltimore classes (dsDNA: yellow, ssDNA: light green, dsRNA: bright green, +ssRNA: light blue, -ssRNA: dark blue, +ssRNA with DNA intermediate: purple, dsDNA with RNA intermediate: red). Shape indicate genes detected as composite (triangle), as multi-composite (diamond) or not detected as such (circle). COG or KOG functional category is indicated below when known.
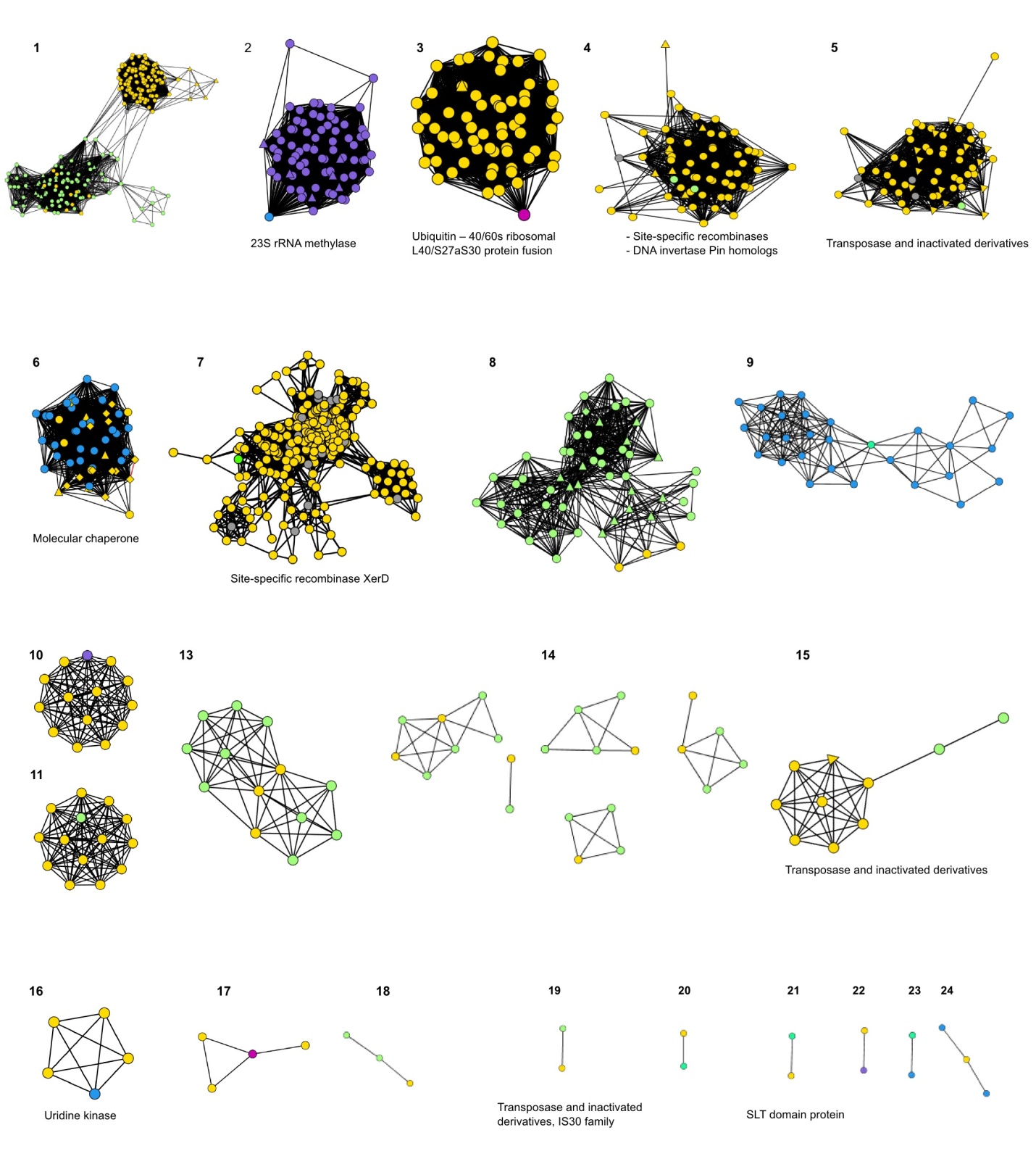


Fig. S6: **Gene similarity across viral classes inside the giant connected component**Regions of the sequence similarity graph, inside of the giant connected component, presenting similarity between genes carried from virus of different Baltimore classes. Colors correspond to Baltimore classes (dsDNA: yellow, ssDNA: light green, dsRNA: bright green, +ssRNA: light blue, -ssRNA: dark blue, +ssRNA with DNA intermediate: purple, dsDNA with RNA intermediate: red). Shape indicate genes detected as composite (triangle), as multi-composite (diamond) or not detected as such (circle). COG or KOG functional category is indicated below when known.
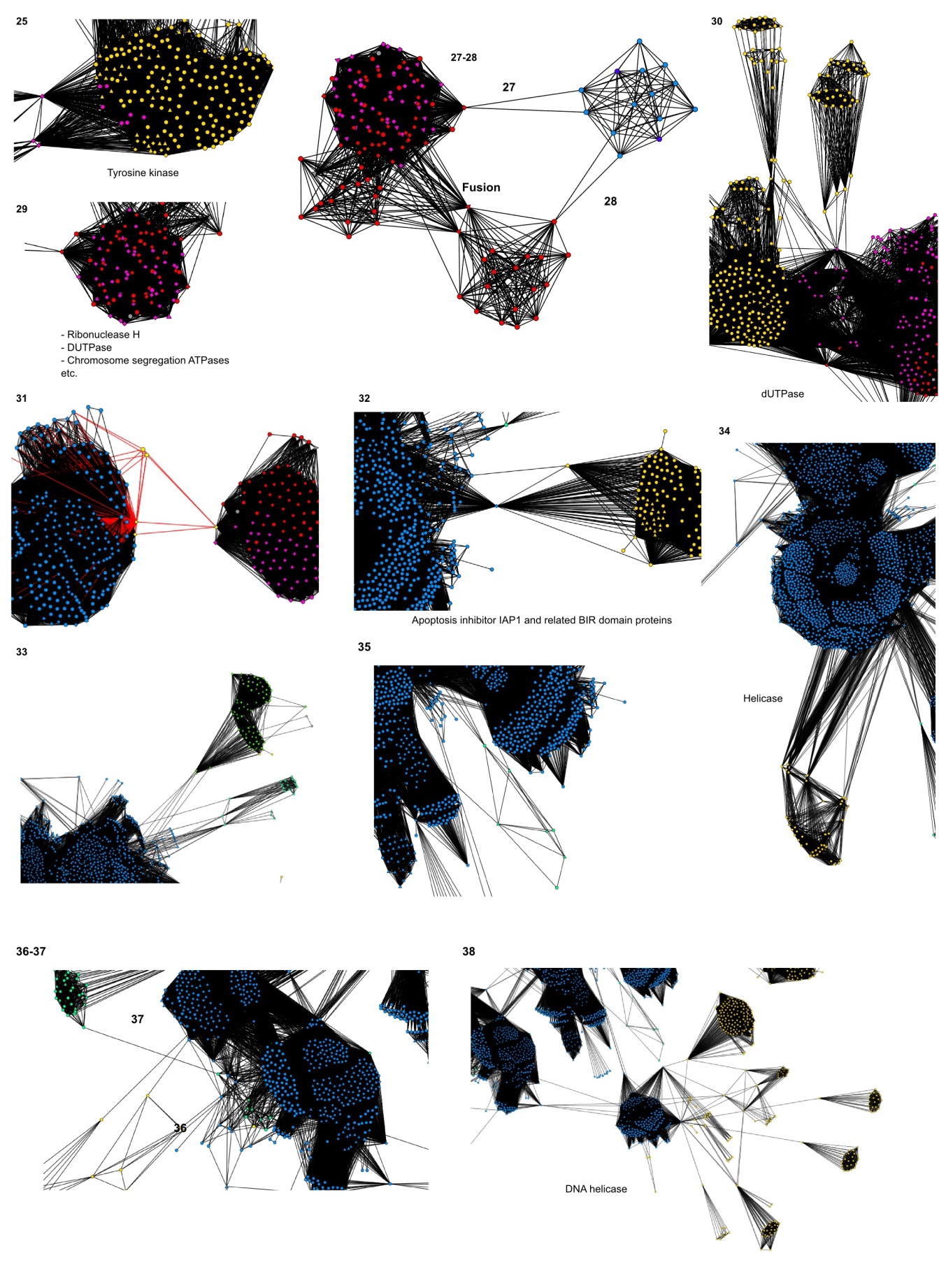


Table S2: **Overview of the dataset**

Statistics on the cellular, plasmidic and viral sequences analyzed to identify important viral versus cellular functions.


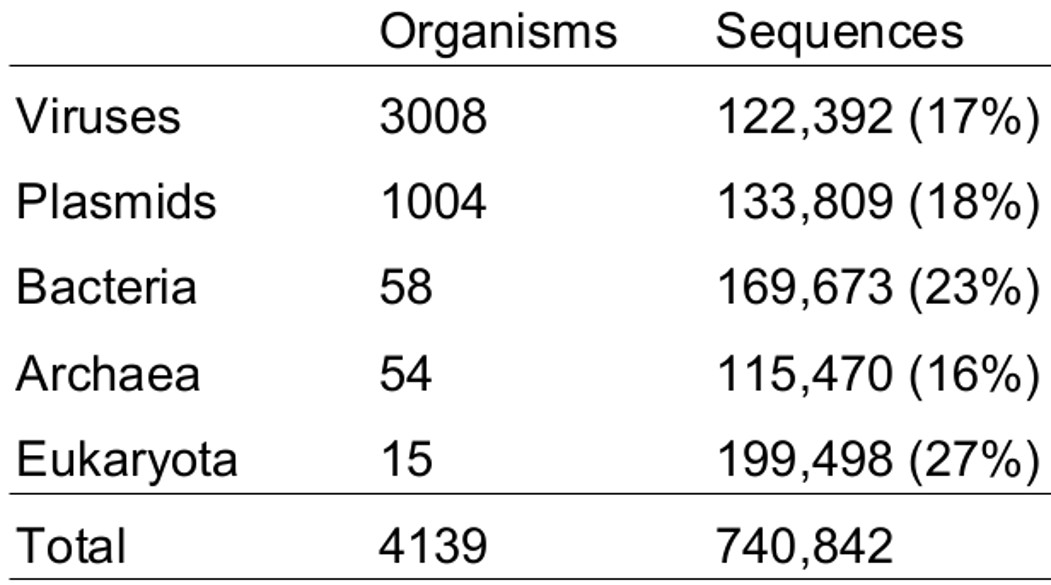


Table S3: **Detailed composition of the dataset and viral annotations**

Names and number of genes of each genome analyzed in the viral and in the extended comparative dataset. Classification of all viral genomes according to their DNA or RNA nature, and into Baltimore and major monophyletic classes as described by Koonin et al. (*41*).

*Table S3 is 53 pages long and submitted separately.*
